# ^183^W NMR Spectroscopy Guides the Search for Tungsten Alkylidyne Catalysts for Alkyne Metathesis

**DOI:** 10.1002/anie.202009975

**Published:** 2020-09-24

**Authors:** Julius Hillenbrand, Markus Leutzsch, Christopher P. Gordon, Christophe Copéret, Alois Fürstner

**Affiliations:** ^1^ Max-Planck-Institut für Kohlenforschung 45470 Mülheim/Ruhr Germany; ^2^ Department of Chemistry and Applied Biosciences ETH Zürich Vladimir-Prelog-Weg 1–5 8093 Zürich Switzerland

**Keywords:** ligand design, metathesis, NMR spectroscopy, structure elucidation, tungsten

## Abstract

Triarylsilanolates are privileged ancillary ligands for molybdenum alkylidyne catalysts for alkyne metathesis but lead to disappointing results and poor stability in the tungsten series. ^1^H,^183^W heteronuclear multiple bond correlation spectroscopy, exploiting a favorable ^5^ *J*‐coupling between the ^183^W center and the peripheral protons on the alkylidyne cap, revealed that these ligands upregulate the Lewis acidity to an extent that the tungstenacyclobutadiene formed in the initial [2+2] cycloaddition step is over‐stabilized and the catalytic turnover brought to a halt. Guided by the ^183^W NMR shifts as a proxy for the Lewis acidity of the central atom and by an accompanying chemical shift tensor analysis of the alkylidyne unit, the ligand design was revisited and a more strongly π‐donating all‐alkoxide ligand prepared. The new expanded chelate complex has a tempered Lewis acidity and outperforms the classical Schrock catalyst, carrying monodentate *tert*‐butoxy ligands, in terms of rate and functional‐group compatibility.

## Graphical abstract

Silanolate ligands excel with molybdenum alkylidynes but are found to be inadequate for tungsten in that they impart undue Lewis acidity. This effect is manifested in the ^183^W NMR shifts, which are surprisingly easy to record by ^1^H,^183^W HMBC experiments. As a consequence, the ligand scaffold was redesigned and an unprecedented chelate alkoxide complex, which is a new lead in the quest for competent tungsten‐based alkyne metathesis catalysts, was developed.

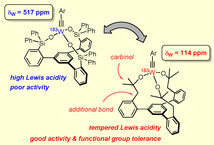

## Introduction

**Figure 1:**
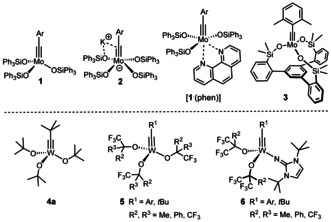
Selected alkylidyne catalysts for alkyne metathesis.

Molybdenum alkylidyne complexes endowed with triarylsilanolate ligands such as **1**, the corresponding ate‐complex **2** and the derived bench‐stable phenanthroline adduct [**1**⋅(phen)] set the standards in the field of alkyne metathesis (Figure 1).[^1^, ^2^, ^3^, ^4^, ^5^] Their functional group compatibility is largely unrivaled;[^6^, ^7^, ^8^, ^9^, ^10^] it has recently been further improved by the development of a second catalyst generation distinguished by a tripodal silanolate ligand framework.[^11^, ^12^] Specifically, the “canopy complex” **3** and relatives maintain the virtues of the parent complex **1**, yet allow the chelate effect to be harnessed in form of an improved stability towards protic sites; in conjunction with the well‐balanced electrophilic character and proper steric protection of the operative Mo≡CR unit, this results in an excellent overall application profile.^[11]^

From a historic perspective, however, tungsten alkylidyne complexes had taken the lead:^[13]^ Complex **4 a** developed by Schrock and co‐workers was the first molecularly well‐defined catalyst for alkyne metathesis;^[14]^ it played a quintessential role in deciphering the mechanism of this transformation^[15]^ and empowered early applications,^[16]^ even though these examples also witnessed that the functional group tolerance is limited.[^16^, ^17^, ^18^, ^19^] Related tungsten alkylidynes, most notably complexes of types **5** and **6** carrying more electron‐withdrawing (partly) fluorinated alkoxide ligands, have also been thoroughly studied from the organometallic viewpoint,[^20^, ^21^, ^22^, ^23^] but the relatively few available reactivity data do not convincingly prove that their scope is significantly broader than that of **4 a**.^[24]^

## Results and Discussion

### Tungsten Alkylidynes with a Tripodal Silanolate Ligand Architecture

**Scheme 1:**
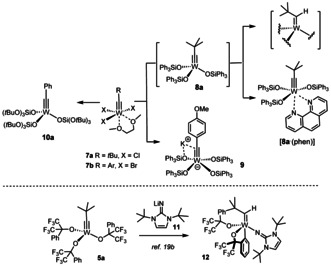
Known tungsten alkylidynes with silicon‐based ligands; decomposition by arene C−H activation with concomitant formation of an alkylidene unit.

When seen against this backdrop, it is perhaps surprising that tungsten alkylidynes with silanolates as ancillary ligands are conspicuously underrepresented in the literature (Scheme 1). The neopentylidyne complex **8 a** is accessible from **7 a** by salt metathesis but was found to degrade rapidly in solution as well as in the solid state; the derived phenanthroline adduct [**8 a**⋅(phen)] is stable but kinetically inert and hence no adequate precatalyst either.[^25^, ^26^] Unligated **8 a** succumbs to a C−H activation process that converts the alkylidyne into an alkylidene unit: while this transformation is clearly manifested in the NMR data,^[27]^ the resulting complex could not be obtained in crystalline form and hence remains structurally undefined.^[25]^ In this context, reference is made to a similar C−H activation observed for **5 a** on treatment with **11**.^[22]^ This decomposition pathway may be the reason why yet another complex carries siloxide ligands devoid of aromatic substituents in the periphery; once again, the fairly small number of applications that **10 a** has found so far, mostly to conjugated 1,3‐diyne substrates, make an accurate assessment difficult.[^28^, ^29^]

On top of this stability issue comes a practical problem: other than the trichloro alkylidyne **7 a**, the much more readily available tribromo complexes of type **7 b** ^[30]^ with an aryl substituent on the alkylidyne invariably led to formation of the corresponding ate‐complexes such as **9** on treatment with Ph_3_SiOK in toluene.[^1a^, ^31^] For the intrinsically high Lewis‐acidity of tungsten, they do not lose the fourth silanolate to vacate the necessary coordination site for the incoming substrate under conditions that work well in the molybdenum series.^[1]^ Although **9** exhibits modest catalytic activity at high temperature in the homo‐metathesis of 1‐phenyl‐1‐propyne, it cannot compete at all with its molybdenum counterpart **2**.[^1^, ^25^] Interestingly, however, **9** and relatives cleave the triple bond of aryldiazonium salts [Ar−N≡N]^+^ with surprising ease at a rate that outcompetes loss of dinitrogen.^[32]^

**Scheme 2:**
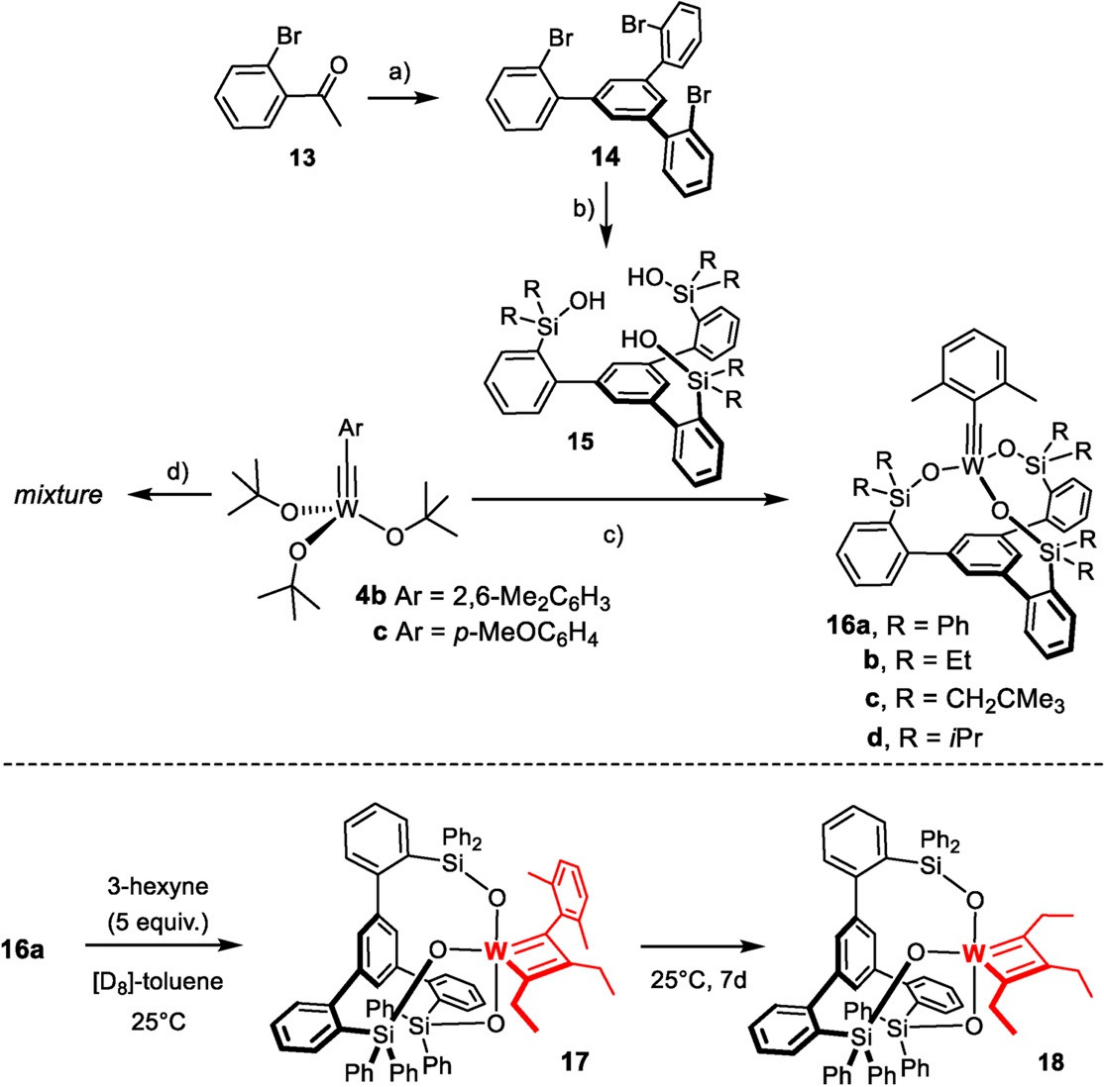
a) SiCl_4_, EtOH, 0 °C → RT, 90 % (X=H, 20 g scale); b) see ref. [11]; c) **4 b**, **15**, toluene, 79 % (**16 a**), 98 % (**16 b**), quant. (**16 c**), 99 % (**16 d**); d) **4 c**, **15** (R=Ph), toluene.

These issues notwithstanding, we conjectured that the tripodal silanolate ligand framework of the new “canopy catalysts” such as **3** might provide an opportunity for the development of truly competent tungsten catalysts. The phenyl ring forming the basal plane blocks the coordination site *trans* to the alkylidyne and should hence prevent detrimental C−H activation as observed for **5 a** and **8 a** from occurring. Moreover, the problem with competing ate‐complex formation can likely be solved by a new synthesis route that avoids salt metathesis (Scheme 2):^[11]^ Specifically, it was found that **3** is best prepared on treatment of [(*t*BuO)_3_Mo≡CAr] with trisilanole **15**; the ligand exchange is entropically driven and benefits from the different acidity of alcohols and silanols. Since deprotonation of the ligand is unnecessary, irreversible ate‐complex formation is effectively prevented.

**Figure 2:**
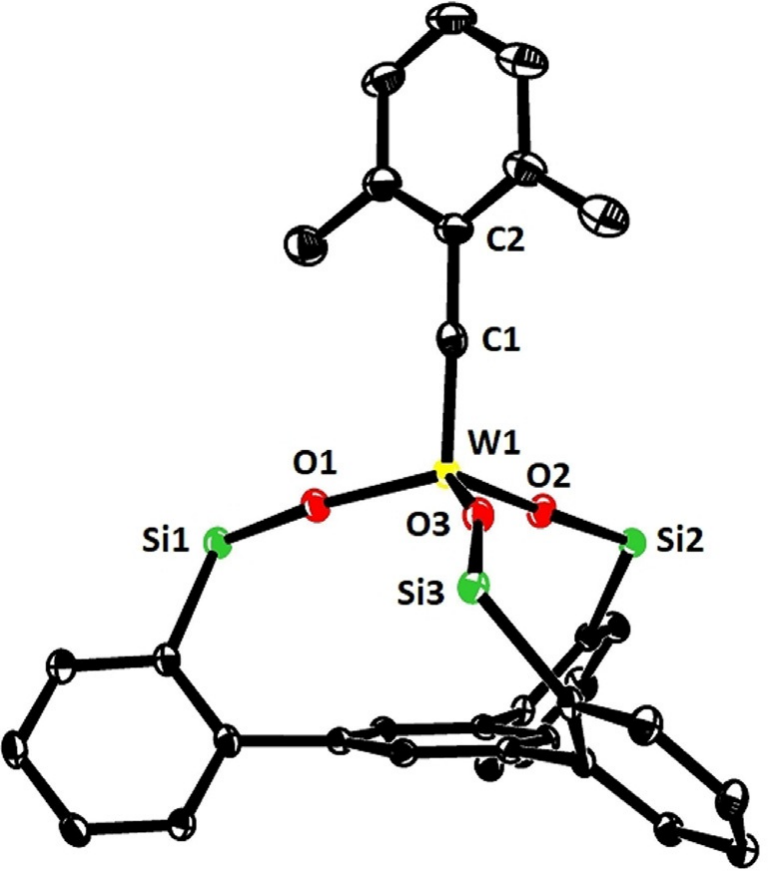
Representation of the truncated structure of the tungsten alkylidyne complex **16 a** with a tripodal ligand framework in the solid state, in which the lateral phenyl rings on the silicon atoms were removed for clarity (for the full structure, see the SI); likewise, co‐crystallized Et_2_O and all H‐atoms are not shown.^[75]^ Selected bond lengths [Å] and angles [°]: W1–C1 1.759(3), C1–C2 1.453(5), W1–O1 1.880(2), W1–O2 1.884(2), W1–O3 1.873(2); W1‐C1‐C2 176.5(3), Si1‐O1‐W1 171.4 (2), Si2‐O2‐W1 159.1(2), Si3‐O3‐W1 167.0(2).

In consideration thereof, the analogous complex **4 b** ^[33]^ was chosen as the starting point for the preparation of a series of new tungsten alkylidynes; it differs from the classical Schrock catalyst **4 a** ^[14]^ only in the substituent of the alkylidyne but is easier to make on scale. In line with our expectation, reaction of **4 b** with **15 a** (R=Ph) in toluene afforded **16 a** in 79 % yield (Scheme 2),^[34]^ whereas **4 c** lacking the 2,6‐dimethyl substitution on the benzylidyne furnished a complex mixture. The structure of **16 a** in the solid state (Figure 2) shows great resemblance to the analogous molybdenum canopy complexes,^[11]^ even though the short W−O bonds bear testimony to the higher Lewis‐acidity of tungsten. The catalytic activity, however, was disappointing and the likely reason readily identified. Addition of 3‐hexyne (5 equiv) to a solution of **16 a** in [D_8_]toluene caused an instant color change from yellow to deep red: Upon cooling to −20 °C, a sharp signal set was recorded that allowed the new species to be identified as the *mixed* metallacyclobutadiene complex **17** formed in the initial [2+2] cycloaddition step (Scheme 2).^[35]^ Although 3‐hexyne was present in excess, it took one week at ambient temperature for **17** to convert into metallacyclobutadiene **18** carrying three ethyl substituents by reaction with a second equivalent. The striking inertia of **17** as the “snapshot” of the “initiation” step of the catalytic cycle implies that cycloreversion is not only rate‐determining, as expected,^[36]^ but is much too slow for a competent catalyst. Such hyper‐stabilization of the metallacycle is well known to be the result of an overly Lewis‐acidic metal fragment.[^20^, ^37^, ^38^, ^39^] In this context, it needs to be emphasized that treatment of an analogous molybdenum canopy complex with 3‐hexyne unexpectedly furnished a metallatetrahedrane complex;[^11^, ^12^] such disparate behavior of tungsten and molybdenum alkylidynes with the same ancillary ligand sphere is striking and unprecedented.

**Figure 3:**
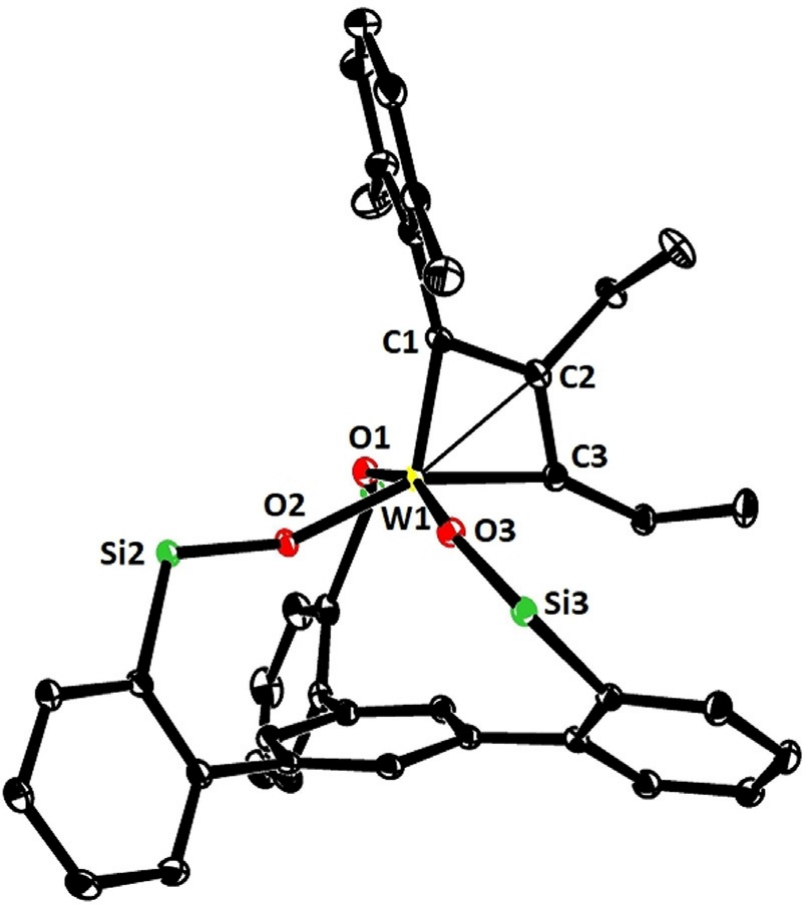
Representation of the truncated structure of the metallacyclobutadiene complex **17** with a tripodal ligand framework in the solid state, in which the lateral phenyl rings on the silicon bridges and all H‐atoms were removed for clarity.^[75]^ Selected bond lengths [Å] and angles [°]: W1–C1 1.877(2), W1–C3 1.965(2), W1–C2 2.152(2), W1–O1 1.988(1), W1–O2 1.903(1), W1–O3 1.952(1); W1‐C1‐C2 78.2(1), W1‐C3‐C2 77.2(1), C1‐C2‐C3 121.5(1), Si1‐O1‐W1 153.9(1), Si2‐O2‐W1 155.3(1), Si3‐O3‐W1 149.8(1).

**Figure 4:**
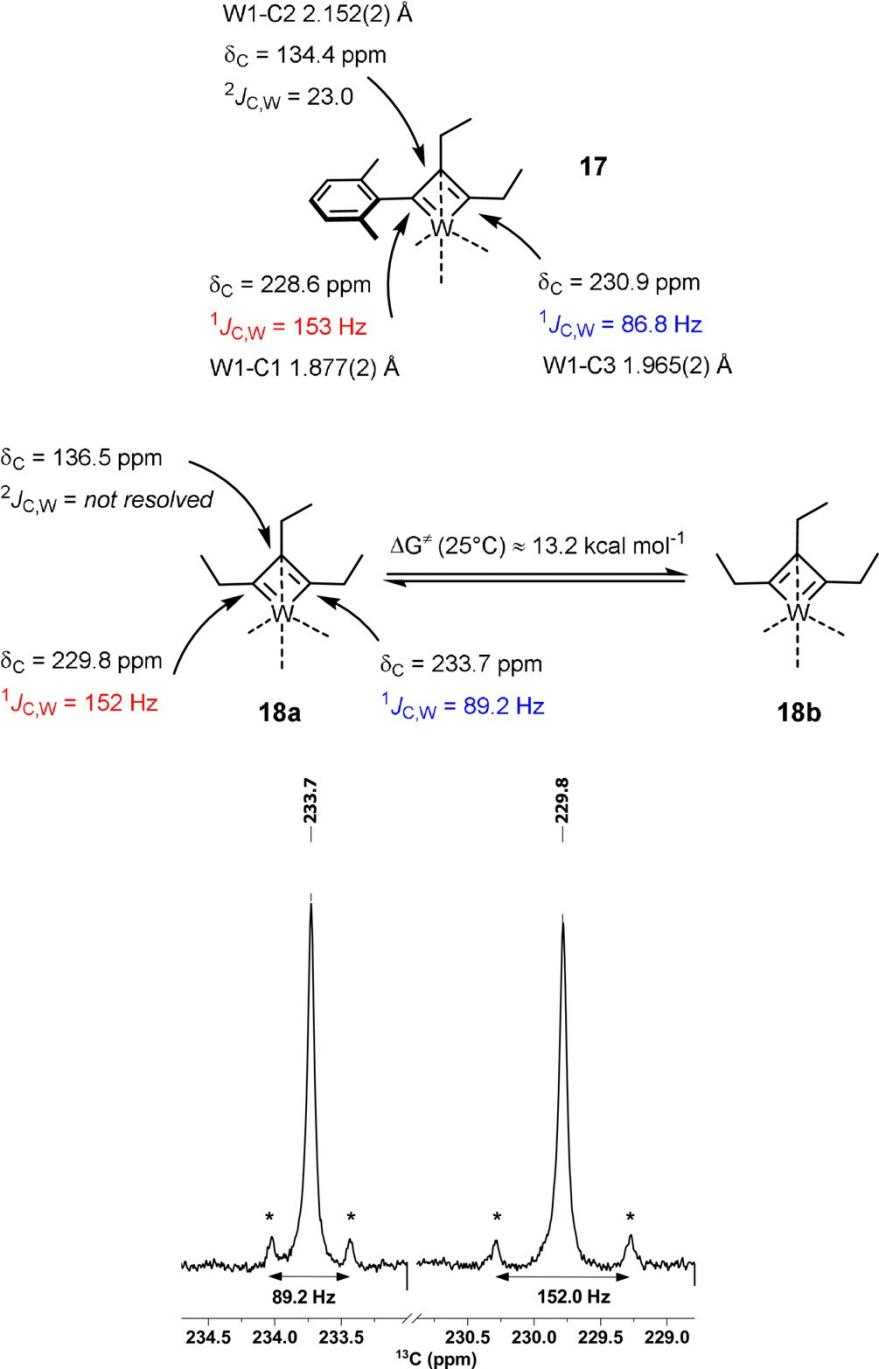
Selected spectral and crystallographic data of the metallacyclobutadiene subunits of **17** and **18**; the insert shows the satellite peaks for the C_α_/C_α_′ atoms of **18**.

The coordination geometry about the tungsten center of **17** (Figure 3) is much closer to square pyramidal (*τ*_5_≈0.14) than to the trigonal bipyramidal arrangement, which basically all other known tungstenacyclobutadiene complexes adopt;[^14b^, ^20^, ^23^, ^24b^, ^37^, ^38^, ^39^, ^40^] this difference is likely a consequence of the chelate ligand framework and may contribute to the low reactivity of **17**.^[41]^ The equatorial siloxide serves as a notably better π‐electron donor to tungsten, since the W1‐O2 bond (1.903(1) Å), which is essentially coplanar with the metallacycle, is significantly shorter than the orthogonal W1‐O1 (1.988(1) Å) and W1‐O3 (1.952(1) Å) bonds. The metallacyclobutadiene ring itself is highly distorted, in that all bond lengths and angles are uneven. This finding is in accord with the spectroscopic data which show that the C_α_/C_α′_ atoms of the metallacycle in **17** are inequivalent (Figure 4). Most notable are the different ^1^ *J*_C,W_ coupling constants: although a detailed interpretation is difficult,^[42]^ we note that the shorter W1‐C1 “double” bond features the much larger ^1^ *J*_C,W_ coupling constant. In the derived complex **18**, each ethyl substituent gives rise to distinct ^1^H and ^13^C NMR signals. Importantly, ROESY‐NMR experiments show a dynamic process that mutually interconverts the C_α_/C_α′_ positions and their ethyl substituents on the NMR timescale,^[43]^ which is interpreted as interconversion of the two tautomers **18 a** and **18 b**.^[44]^ The observation of two different valence isomers implies that the metallacyclobutadiene formed by [2+2] cycloaddition and the metallacyclobutadiene releasing the product in the [2+2] cycloreversion step are *discrete* entities that must not be mistaken for a mesomeric resonance form.^[45]^

### Ligand Design Revisited: Guidance by ^183^W NMR Spectroscopy

**Figure 5:**
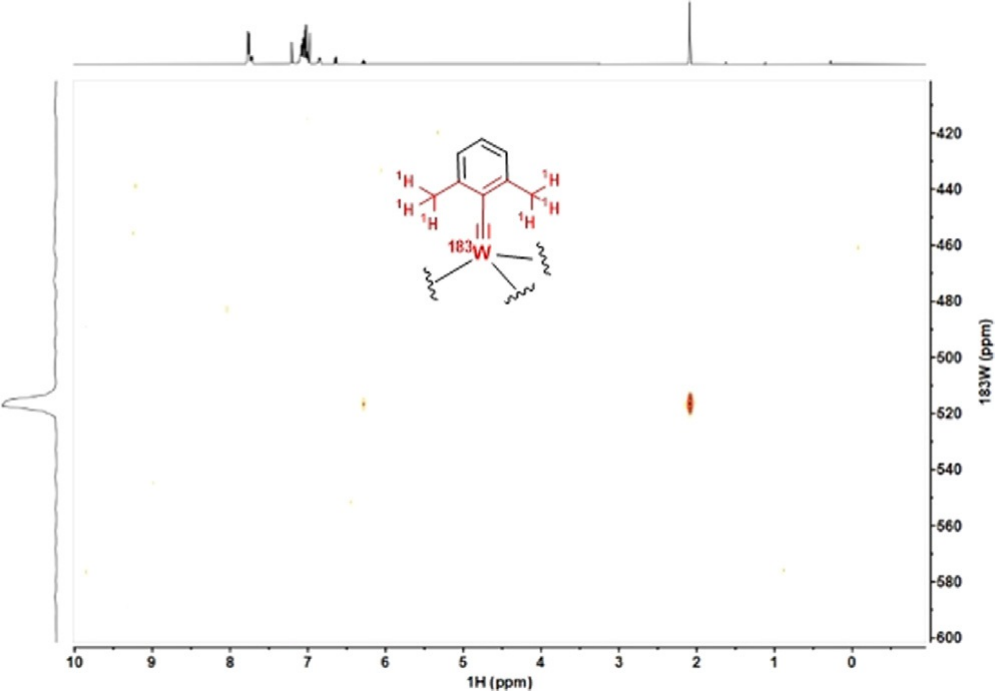
^1^H,^183^W HMBC spectrum of complex **16 a**.

The exceptional kinetic stability of **17** and **18** shows that a simple extrapolation of the successful ligand design from molybdenum to tungsten is to no avail. We had previously recognized that a direct look at the central metal via ^95^Mo NMR spectroscopy was tremendously useful for catalyst design.^[11]^ Therefore it was hoped that similar advantage could be taken from ^183^W NMR in our quest for truly competent tungsten alkylidynes; the very large spectral range of this spin‐1/2 nucleus and the pleasingly narrow signal line widths suggest so. However, the dramatically poor receptivity is a serious handicap in that the low‐*γ* ^183^W isotope is ≈49 times less sensitive than the already insensitive ^95^Mo nucleus.^[46]^ This issue had previously been addressed in various ways, of which 2D shift correlation spectroscopy is arguably the most successful approach.^[47]^ Unfortunately, many tungsten complexes show very small scalar couplings to protons (hydrides), even when directly bound.^[48]^ It is hence truly remarkable that the ^183^W NMR signal of **16 a** (δ_W_=517 ppm) can be detected with ease by ^1^H,^183^W HMBC measurements using long‐range couplings (^5^ *J*) between the tungsten center and the protons on the *ortho*‐methyl groups of the benzylidyne unit (Figure 5).^[49]^ The fact that such ^5^ *J*‐coupling does exist is testament to an efficient orbital overlap between the alkylidyne and its aryl cap.

We are aware of a single prior study into tungsten alkylidynes based on 1D ^183^W NMR spectroscopy.^[50]^ Surprisingly though, the reported ^183^W NMR shift of [(*t*BuO)_3_W≡*CPh*] (δ_W_=2526 ppm) is drastically different from what we record by the ^1^H,^183^W HMBC pulse sequence for the closely related complex **4 b** (δ_W_=166 ppm) carrying two remote methyl substituents on the phenyl ring. Struck by the discrepancy, we sought to rigorously confirm our data point by a number of control experiments. To this end, spectra were recorded with different offset and sweep widths to exclude possible folding of the signals.^[51]^ In addition, a 1D ^183^W NMR spectrum of **4 b** was recorded using an INEPT magnetization transfer, which proved that the inversely detected and the directly recorded δ_W_ are identical within ±0.2 ppm.

**Figure 6:**
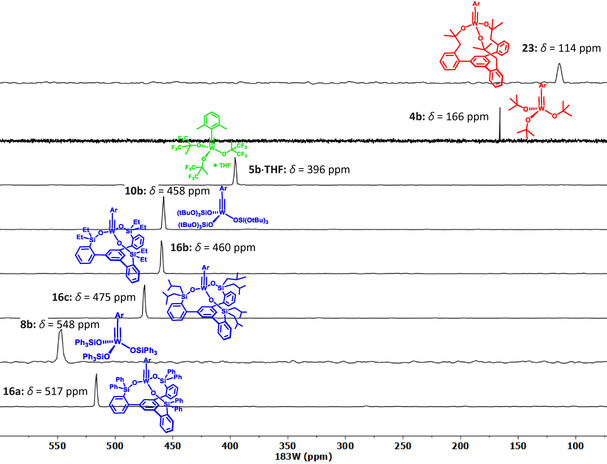
Comparison of the ^183^W NMR shifts ([D_8_]toluene) of different tungsten alkylidyne complexes, as determined by ^1^H,^183^W HMBC experiments (for **4 b**, the INEPT‐based 1D ^183^W NMR spectrum referred to in the text is shown). Siloxide containing complexes are color‐coded in blue, alkoxide complexes in red, and fluoroalkoxide in green; Ar=2,6‐dimethylphenyl.

**Figure 7:**
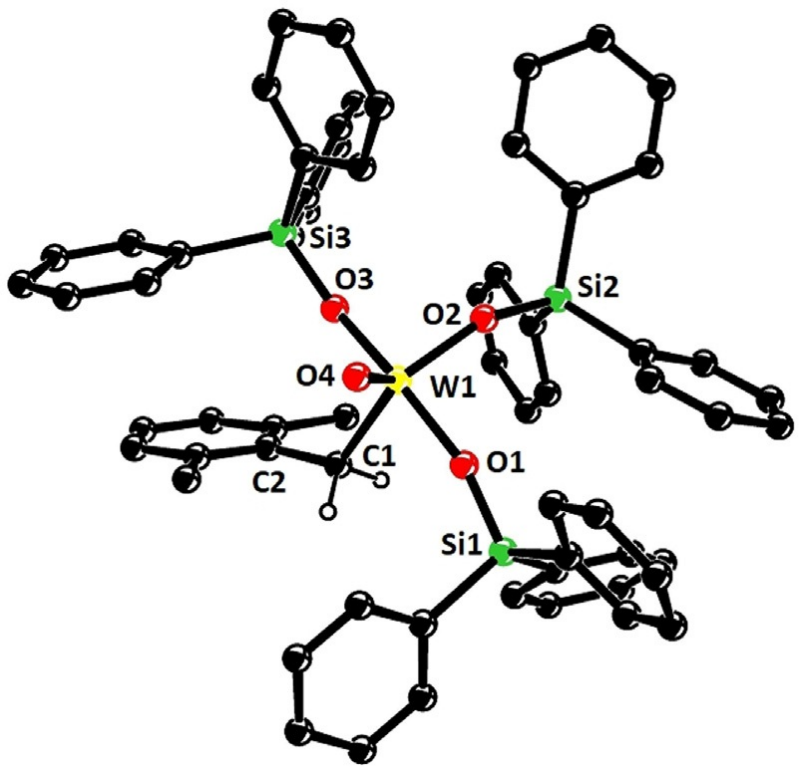
Structure of the unusual benzyl tungsten‐oxo complex **19** in the solid state; all hydrogen atoms except for the benzylic ones were removed for clarity.^[75]^ Selected bond length [Å] and angles [°]: W1–C1 2.180(4), W1–O4 1.703(3), W1–O1 1.877(3), W1–O2 1.889(3), W1–O3 1.886(3); W1‐C1‐C2 122.7(3), Si1‐O1‐W1 159.9(2), Si2‐O2‐W1 150.5(2), Si3‐O3‐W1 165.8(2).

**Scheme 3:**
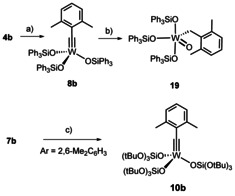
a) Ph_3_SiOH, toluene, 41 %; b) H_2_O, toluene, 50 %; c) (*t*BuO)_3_SiOLi, CH_2_Cl_2_, quant.

After this validation, ^1^H,^183^W HMBC experiments were used to record spectra with an excellent signal‐to‐noise ratio in less than 20 min on a Bruker AVIIIHD 400 MHz NMR spectrometer. Figure 6 shows that the catalytically poorly active podand complex **16 a** is much more deshielded than the traditional Schrock‐type complex **4 b** from which it is derived. Since the over‐stabilization of the metallacyclobutadienes **17** and **18** had shown that the metal center in **16 a** is too Lewis‐acidic, it is tempting to take the deshielding of the ^183^W NMR signal as a proxy.^[52]^ To test this hypothesis more closely, additional tungsten alkylidyne complexes were prepared by the standard methods (Schemes 2 and 3). It is of note that solvolysis of **4 b** by Ph_3_SiOH furnished complex **8 b** bearing three monodentate Ph_3_SiO‐ ligands, which had been beyond reach of salt metathesis because of instant ate‐complex formation. Since the ^183^W NMR signal of **8 b** (δ_W_=548 ppm) is even more deshielded than that of **16 a**, this complex is no promising candidate for catalytic alkyne metathesis.^[53]^ Crystals suitable for X‐ray diffraction could not be grown but repeated attempts furnished a different crop that turned out to be the tungsten d^0^ oxo alkyl complex **19** (Figure 7), inadvertently formed by addition of moisture to the alkylidyne unit of **8 b**. Indeed, this complex can be deliberately made from **8 b** and one equivalent of water in toluene; the strong deshielding of the tungsten center (δ_W_=758.1 ppm, see the SI) is well befitting a W^VI^=O unit. To the best of our knowledge, **19** is only the second example for the formation of a alkyl ligand by controlled double‐protonation of an alkylidyne and the first reported case in which a monomeric oxo‐complex is obtained in this way.[^54^, ^55^, ^56^, ^57^]

Next, the phenyl groups on the silicon bridges of the tripodal framework were replaced by more electron‐donating alkyl substituents (note that complex **3** as the currently best canopy catalyst of the molybdenum series carries methyl rather than phenyl groups).^[11]^ As expected, the spectral response is a shift of the ^183^W signal to higher frequencies, even though complexes **16 b** (R=Et) and **16 c** (R=CH_2_CHMe_2_) are still much more deshielded than the parent complex **4 b** (Figure 6). The same is true for **10 b** ^[33]^ carrying permethylated siloxide rather than silanolate ligands, which differs from complex **10 a** ^[28]^ as the only previously disclosed tungsten alkylidyne with a silicon‐based ancillary ligand sphere solely in the substitution pattern of the aryl cap on the alkylidyne. The expectation that the almost identical ^183^W NMR shifts of **10 b**, **16 b** and **16 c** might translate into similar performance proved correct: all of them effect the homo‐metathesis of 1‐methoxy‐4‐(prop‐1‐yn‐1‐yl)benzene at ambient temperature in toluene,^[58]^ but the mass balance was invariably poor because of substantial competing polymerization, independent of whether MS 5 Å was added or not.^[59]^

To complete the picture, Figure 6 also includes the ^183^W NMR spectrum of [ArC≡W{OC(CH_3_)(CF_3_)_2_}_3_]⋅THF (**5 b**, Ar=2,6‐dimethylphenyl) for comparison. Despite the presence of THF as an additional neutral donor ligand, the observed signal at δ_W_=396 ppm speaks for a highly Lewis‐acidic alkylidyne. This conclusion fits well to the previously reported finding that tungsten alkylidynes with hexafluoroalkoxide ligands lead to (over)stabilized metallacycles and are hardly adequate for alkyne metathesis because they lack activity[^38^, ^60^] or cause competing polymerization.[^20^, ^61^] We hence conclude that silanolates (and highly fluorinated alkoxides) as poor π‐ and σ‐donors synergize exceedingly well with molybdenum alkylidynes, but the rather disappointing results obtained with **8 b**, **10 b** and **16 a**–**c** in concord with their strongly deshielded ^183^W NMR signals indicate a fundamental mismatch for the tungsten series that cannot be rectified by peripheral modifications.

**Scheme 4:**
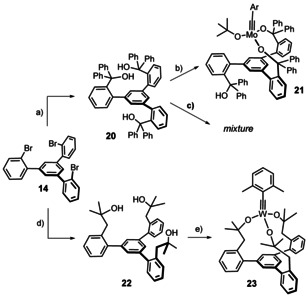
a) *t*BuLi, Et_2_O, benzophenone, −125 °C → RT, 78 %; b) see ref. [11], 79 % (Ar=*p*‐MeOC_6_H_4_); c) **4 b**, toluene; d) *t*BuLi, Et_2_O, isobutylene oxide, −125 °C → RT, 61 % (2.6 g scale); e) **4 b**, toluene, quant. (1.6 g scale).

Based on these data, it became increasingly clear that the Si‐linker strategy had to be abandoned altogether and the search re‐directed toward a ligand with *stronger* (rather than weaker) donor properties than *t*BuO‐ that the inherently more electropositive W^VI^ seems to mandate; if one overshoots, however, catalytic activity will also get lost as a certain Lewis‐acidity is necessary for the [2+2] cycloaddition to proceed.[^3^, ^4^, ^24b^, ^24c^, ^36^] Once again, we reasoned that a chelate structure might provide an adequate balance: the W‐O‐C angles are expected to be more obtuse and the O‐atoms therefore more sp‐hybridized than those of monodentate *t*BuO‐, which should gently upregulate their π‐donor character; an improved stability of a chelate architecture comes on top. However, triol **20** as the carbinol variant of trisilanole **15** is inadequate for this purpose because it is incapable of engaging all three ‐OH groups with a single Mo^VI^ or W^VI^ atom (Scheme 4).[^11^, ^62^] As a consequence, the ligand design was revisited and the expanded *C*_3_‐symmetric triol **22** prepared from **14** in two high‐yielding steps. Despite the additional rotatable bond and the resulting higher conformational freedom, this ligand adopts a favorable “all‐up” conformation in the solid state (see the SI); treatment with **4 b** furnished the targeted chelate complex **23** in almost quantitative yield (Scheme 4).^[63]^

**Figure 8:**
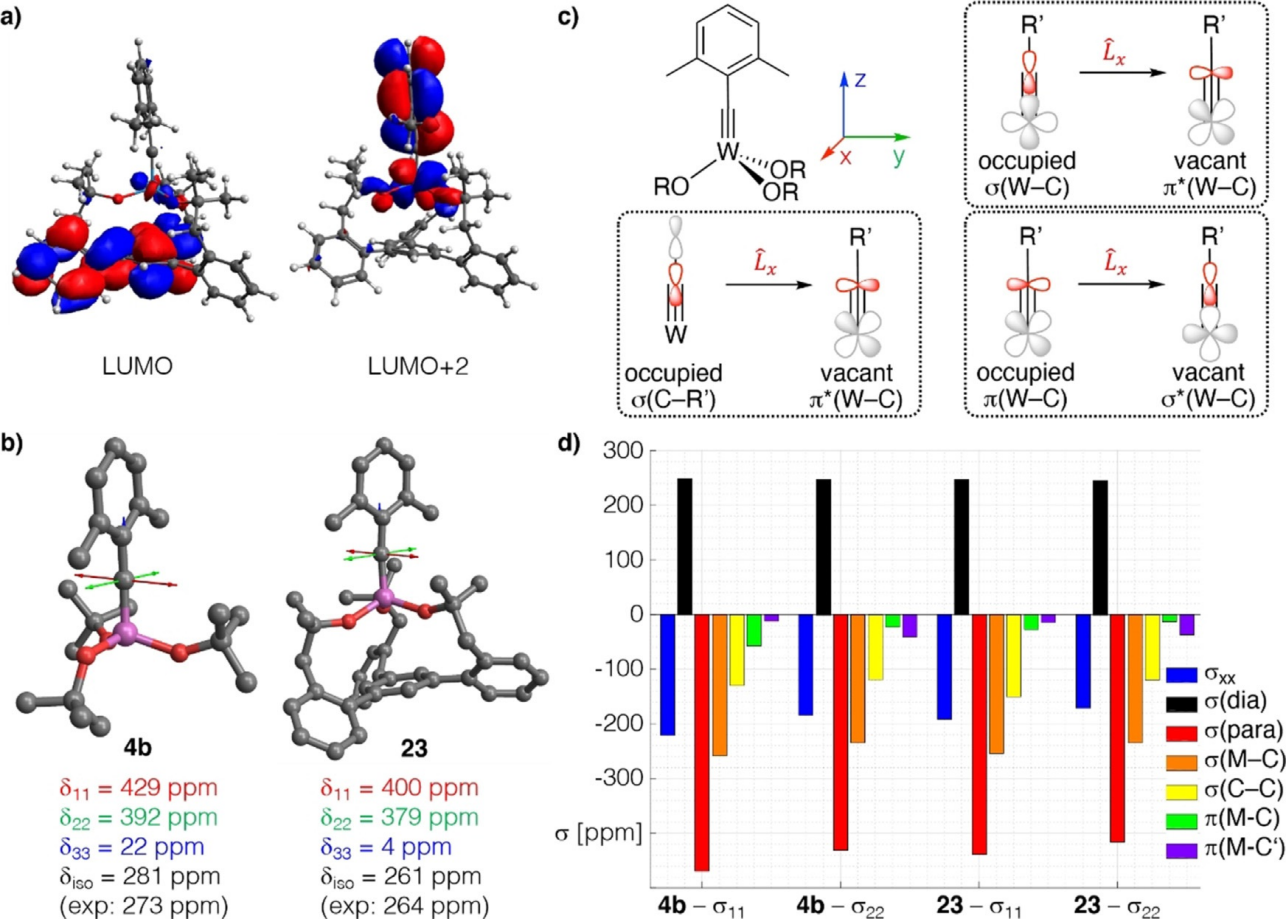
a) Canonical LUMO and LUMO+2 of the tripodal chelate complex **23**. b) Graphical representation of the shielding tensors of the Schrock complex **4 b** and **23**, which is more shielded in all three principle components. c) The three most relevant orbital couplings of a metal alkylidyne. d) Chemical Shift Tensor (CST) analysis for the alkylidyne C‐atom of both complexes based on Natural Localized Molecular Orbitals (NLMOs).

Using the ^1^H,^183^W HMBC technique, a ^183^W resonance at δ_W_=114 ppm was recorded for **23**, which is *upfield* of the signal of the parent complex **4 b** (δ_W_=166 ppm). In line with our expectation that a chelate carbinol ligand is a somewhat better donor for the geometrically enforced substantial widening of the W‐O‐C angles,^[64]^ this data point suggests that the tungsten center of **23** is indeed *less* Lewis‐acidic than that of the classical Schrock‐type complex **4 b**, even though the first coordination sphere is formally made up of (substituted) “*tert*‐butoxy” groups in either case. This view is corroborated by computational data. Specifically, the calculated energies of the canonical MO's are notably different: the tripodal ligand as the better donor raises the energy of the π‐symmetric orbitals. This effect is slightly manifested in the π(W‐C) orbitals (HOMO, HOMO‐1) but particularly notable in the energy of the π*(W‐C) orbitals (for details, see the SI). An important consequence of this uplifting of the π*(W‐C) levels is the fact that the canonical LUMO of **23** is almost entirely ligand‐based, with the largest lobes on the phenyl group that forms the basal plane (Figure 8).

A detailed analysis of the ^13^C NMR data by 2‐component ZORA DFT calculations^[65]^ confirms the conclusions (for full computational details, see the SI): the alkylidyne C‐atom of **23** is clearly more shielded than that of **4 b** (Table 1). Because the computed isotropic shifts (δ_iso_) and ^1^ *J*_W,C_ and ^2^ *J*_W,C_ coupling constants nicely reproduce the experimental values,^[42]^ one can confidently break the data down to the individual components δ_ii_ of the chemical shift tensor (δ_iso_=(δ_11_ + δ_22_ + δ_33_)/3), which themselves are linked to the computed shielding tensor σ (δ_ii_≈σ_iso,ref_−σ_ii_). Details of chemical shift tensor (CST) analysis apart,^[66]^ it has been shown that magnetic shielding reflects an increasing energy gap between high‐lying occupied and the low‐lying vacant orbitals. For metal alkylidynes, the key contributions come from the pairs π(M‐C)→σ*(M‐C), σ(M‐C)→π*(M‐C), and σ(C‐Ar)→π*(M‐C) (Figure 8 c).^[67]^

**Table 1:** Tabulation of recorded and computed NMR data ([D_8_]toluene) of the alkylidyne unit of different tungsten 2,6‐dimethylbenzylidyne complexes. 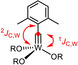

Complex	*δ*_W_ [ppm]	δ_C_ [ppm]		^1^ *J*_W,C_ [Hz]		^2^ *J*_W,C_ [Hz]	
		exp.	computed	exp.	computed	exp.	computed
**16 a**	517	287	285	270	282	37.1	37.3
**8 b**	548	283	274	273	289	30.1	38.9
**10 b**	458	281	280	274	n.d.	38.7	n.d.
**4 b**	166	273	281	297	310	44.8	43.4
**23**	114	264	262	293	304	44.4	43.4

n.d.=not determined

The computed data shown in Figure 8 and the results of a Natural Chemical Shielding (NCS) analysis of all complexes compiled in Table 1 (see also the Supporting Information Table S‐13 and Figure S‐35) can be summarized as follows: The contributions arising from π(M‐C) orbitals (and to a lesser extent those of the σ(M‐C) orbitals) decrease upon going from **4 b** to **23**. This reflects the higher‐lying π*(M‐C) orbitals that render **23** less electrophilic, as well as the equally higher‐lying σ*(M‐C) orbital induced by the tripodal ligand. It is also interesting to note that the lower‐lying of the π*(M‐C) orbitals in **4 b** and **23** is associated with the less‐deshielding σ_22_ component. This presumably results from the delocalization of the π(M‐C) system over the dimethylphenyl moiety, which decreases the efficiency of the orbital coupling. The component oriented along the M‐C axis (σ_33_) is particularly shielded in **23** and points to a highly cylindrical electron density distribution around this M−C bond, as is observed for acetylene and molecules with a C_∞_ axis,^[68]^ making it difficult to draw a direct relation to the HOMO–LUMO gap for this component.

One can therefore safely conclude that complex **23** must not be mistaken for just a tethered version of the classical Schrock catalyst; rather, the tripodal ligand topology itself constitutes an intrinsic electronic determinant. With the canonical LUMO delocalized on the ligand framework and a relatively high lying metal‐centered π*(W‐C) orbital (LUMO+2), over‐stabilization of a derived metallacycle is improbable; a good level of activity and selectivity may therefore be expected.

### Benchmarking

**Figure 9:**
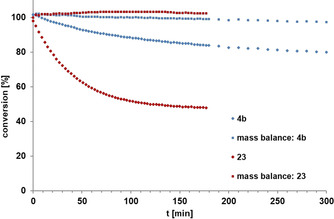
Comparison of the intrinsic reactivity of the tripodal complex **23** with that of the ordinary Schrock catalyst **4 b** with monomeric *tert*‐butoxy ligands in the homo‐metathesis of 1‐methoxy‐4‐(prop‐1‐yn‐1‐yl)benzene to 4,4′‐dimethoxytolane ([D_8_]toluene, 0.1 M, 27 °C, 5 mol % catalyst loading).

**Scheme 5:**
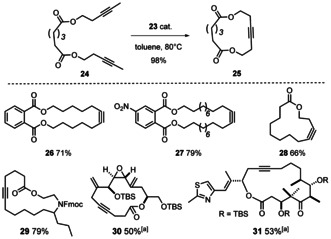
Representative RCAM reactions. Unless stated otherwise, all reactions were performed with 5 mol % of catalyst. [a] With 30 mol % catalyst.

Indeed, the direct comparison shows that **23** outperforms the Schrock catalyst **4 b** in terms of rate; moreover, polymerization has essentially ceased, as evident from the mass balance data (Figure 9).^[69]^ When performed in the presence of silanized MS 5 Å to sequester the released 2‐butyne,^[70]^ the reaction furnished the desired homo‐metathesis product in 85 % yield (NMR). Good results were also achieved in a number of demanding ring closing alkyne metathesis (RCAM) reactions (Scheme 5). Since acid‐sensitive epoxides and aldol subunits as well as many heteroatom donor sites are known to be incompatible with the parent Schrock catalyst **4 a**,[^16^, ^17^, ^71^, ^72^] it is notable that complex **23** opens new opportunities. The successful formation of cycloalkyne **30** bears witness of this notion, since this compound comprises a very acid‐ and base‐sensitive vinylepoxide subunit, a labile allylic ether as well as a skipped and hence non‐thermodynamic array of double and triple bonds.^[73]^ The formation of cycloalkyne **31** is equally indicative: for the presence of a highly elimination‐prone aldol subunit and the additional donor site of the thiazole ring in the side chain, this particular compound had previously been reported to lay beyond the reach of the classical Schrock catalyst **4 a**.^[74]^

## Conclusion

Although the new prototype complex **23** does not yet rival the best molybdenum alkylidynes known to date, it constitutes a new lead in the tungsten series. From the conceptual viewpoint, we are inclined to believe that future ligand design must try to better balance rather than simply increase the Lewis acidity of the operative tungsten alkylidyne unit; this conclusion, however, is very much at odds with much of what has been practiced during the last decades. Any such endeavor will likely benefit from reverse 2D ^1^H,^183^W spectroscopy exploiting long‐range coupling, which helped us to uncover the reasons why the poorly π‐ and σ‐donating silanol ligands that excel in the molybdenum series are by and large inadequate for tungsten. This insight then resulted in the development of a new catalyst endowed with an expanded tripodal alkoxide ligand, which outperforms the classical Schrock catalyst. We plan to take further advantage of ^183^W NMR as a particularly relevant analytical technique in our ongoing quest for evermore competent catalysts.

## Conflict of interest

The authors declare no conflict of interest.

## Supporting information

As a service to our authors and readers, this journal provides supporting information supplied by the authors. Such materials are peer reviewed and may be re‐organized for online delivery, but are not copy‐edited or typeset. Technical support issues arising from supporting information (other than missing files) should be addressed to the authors.

Supplementary
