# Supplementary material for: 183W NMR Spectroscopy Guides the Search for Tungsten Alkylidyne Catalysts for Alkyne Metathesis
Source: Angew Chem Int Ed Engl. 2020 Sep 24;59(48):21758–68. doi: 10.1002/anie.202009975 (PMC7756321; doi:10.1002/anie.202009975)
Supplement: Supplementary file 1 — Supplementary [file ANIE-59-21758-s001.pdf]

Angewandte Chemie
Eine Zeitschrift der Gesellschaft Deutscher Chemiker

Supporting Information

^{183}W NMR Spectroscopy Guides the Search for Tungsten Alkylidyne Catalysts for Alkyne Metathesis

*Julius Hillenbrand, Markus Leutzsch, Christopher P. Gordon, Christophe Copéret, and Alois Fürstner**

anie_202009975_sm_miscellaneous_information.pdf

SUPPORTING INFORMATION

COPIES OF SPECTRA

^{1}H NMR of (5'-(2-(diethylsilyl)phenyl)-[1,1':3',1''-terphenyl]-2,2''-diyl)bis(diethylsilane) (S1), 400 MHz, $CDCl_3$, 25ºC

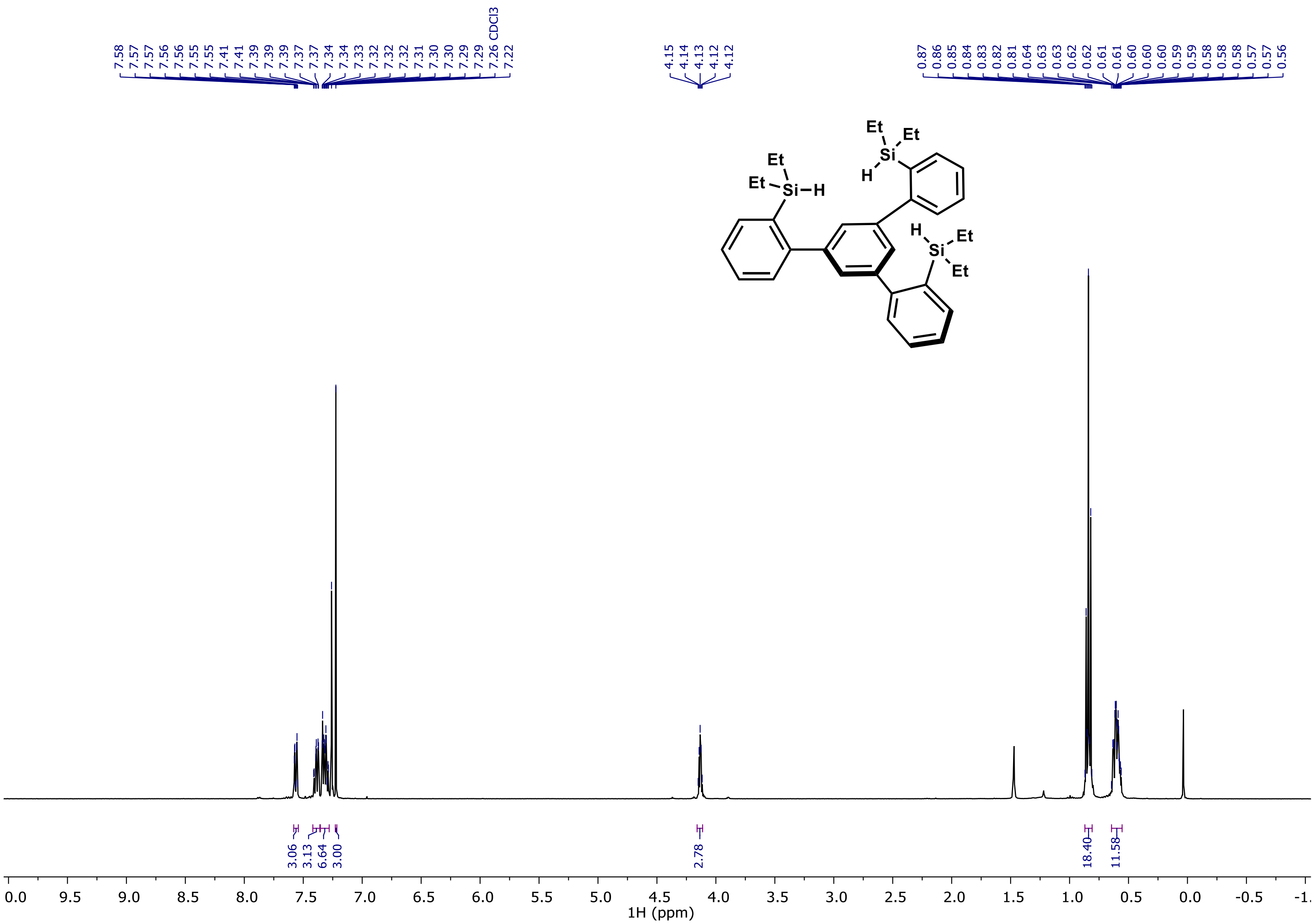

^{13}C NMR of (5'-(2-(diethylsilyl)phenyl)-[1,1':3',1''-terphenyl]-2,2''-diyl)bis(diethylsilane) (S1), 101 MHz, $CDCl_3$, 25ºC

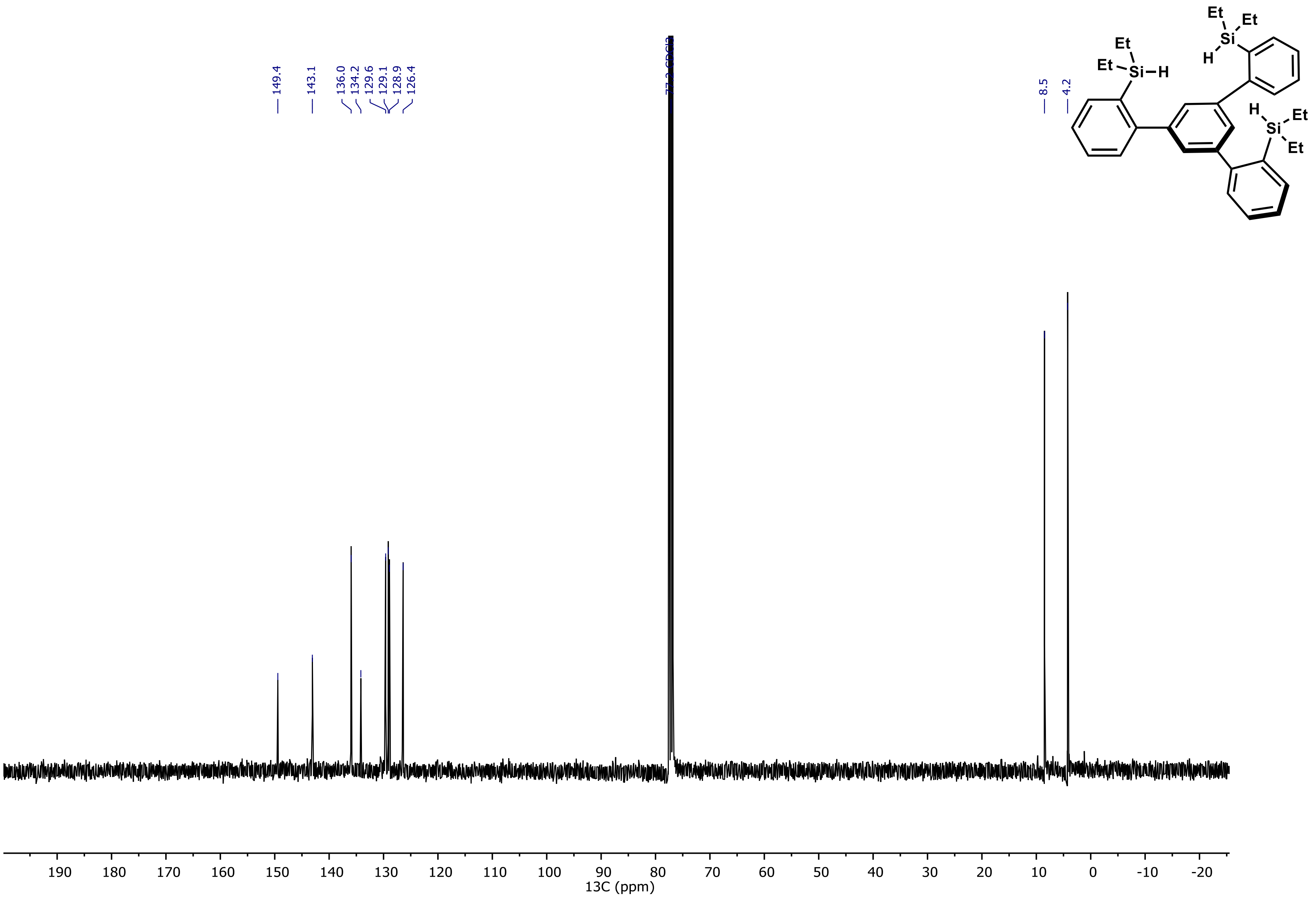

^{1}H NMR of Ligand 15b, 400 MHz, $CDCl_3$, 25ºC

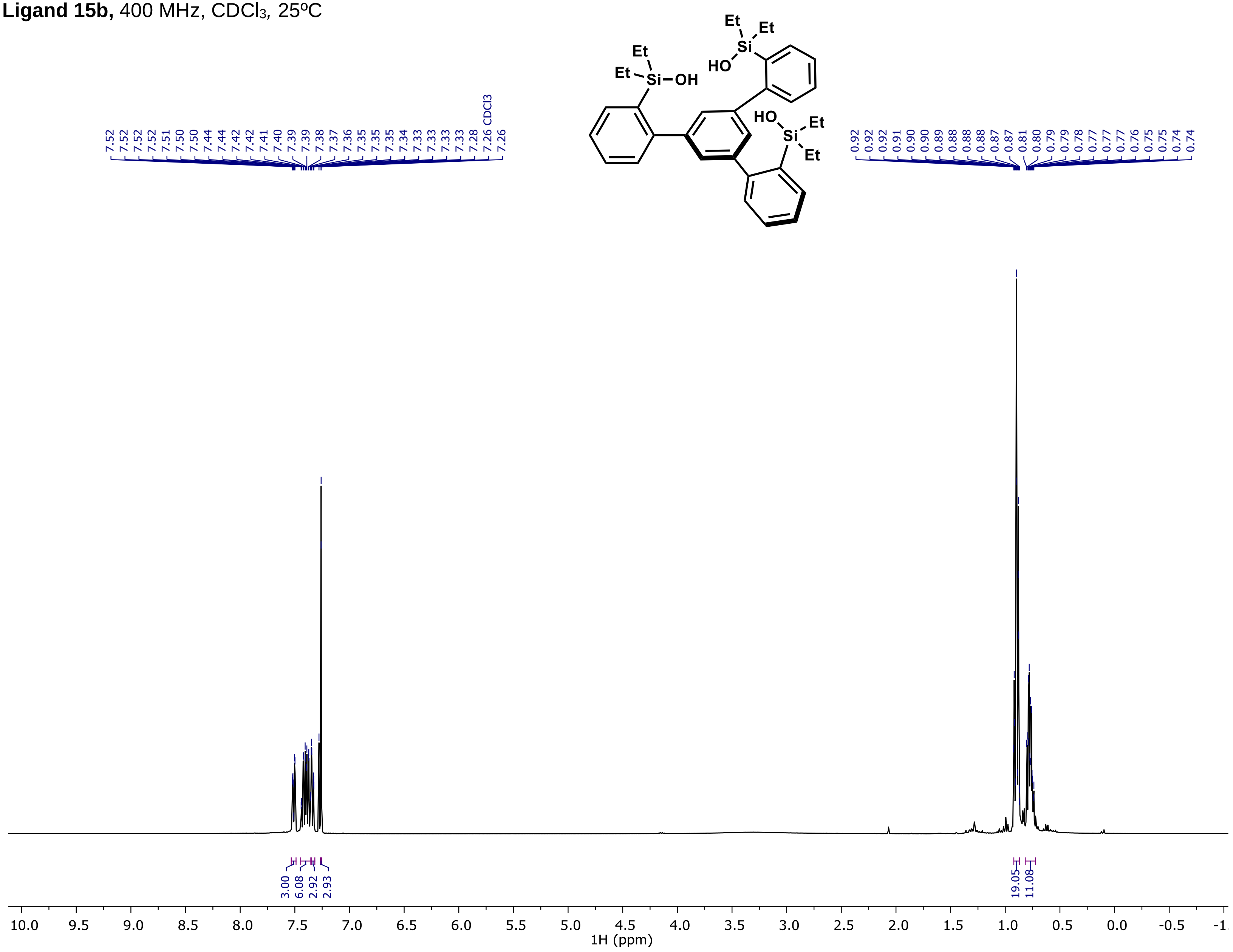

^{13}C NMR of Ligand 15b, 101 MHz, $CDCl_3$, 25ºC

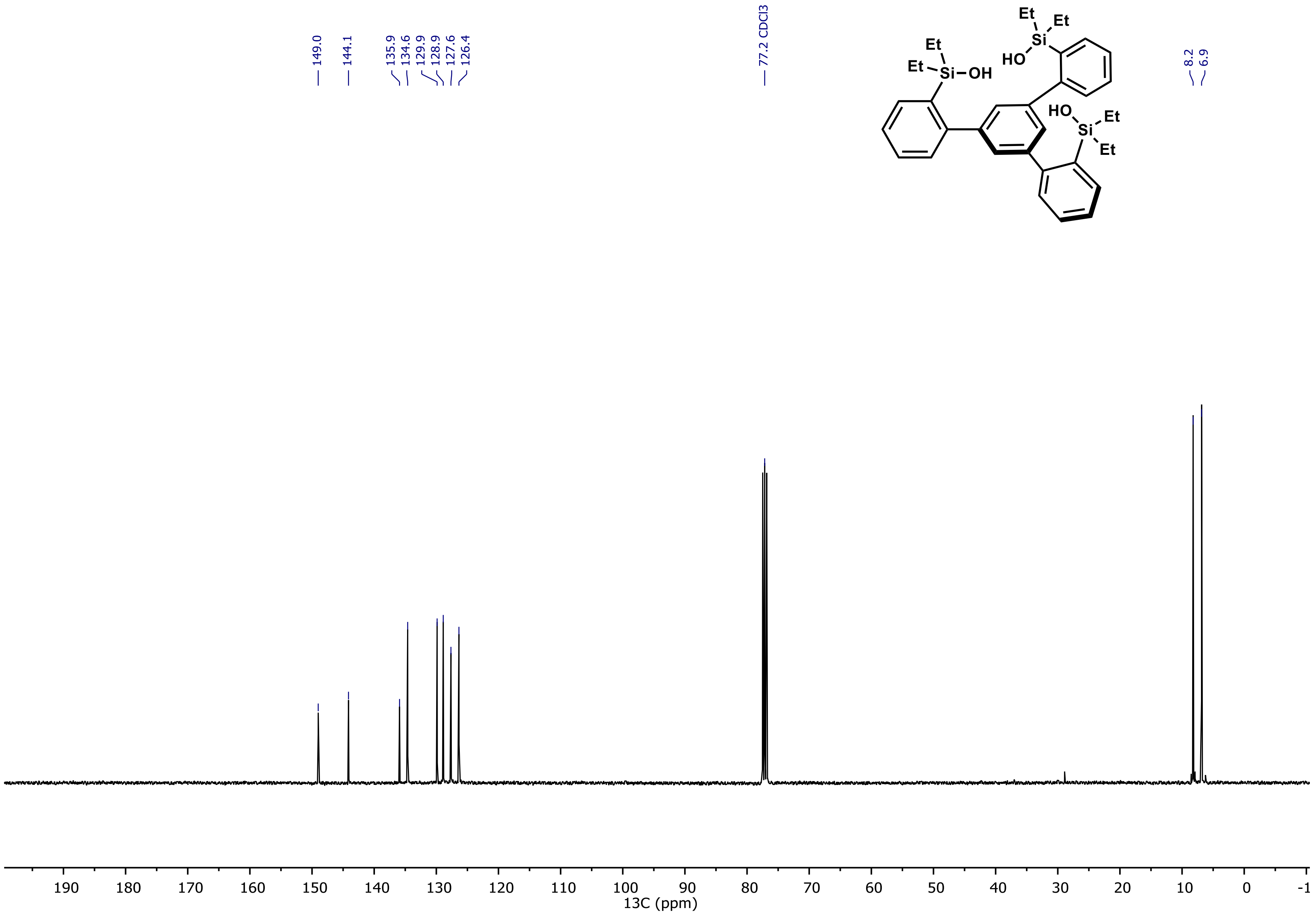

^{29}Si NMR of Ligand 15b, 119 MHz, $CDCl_3$, 25ºC

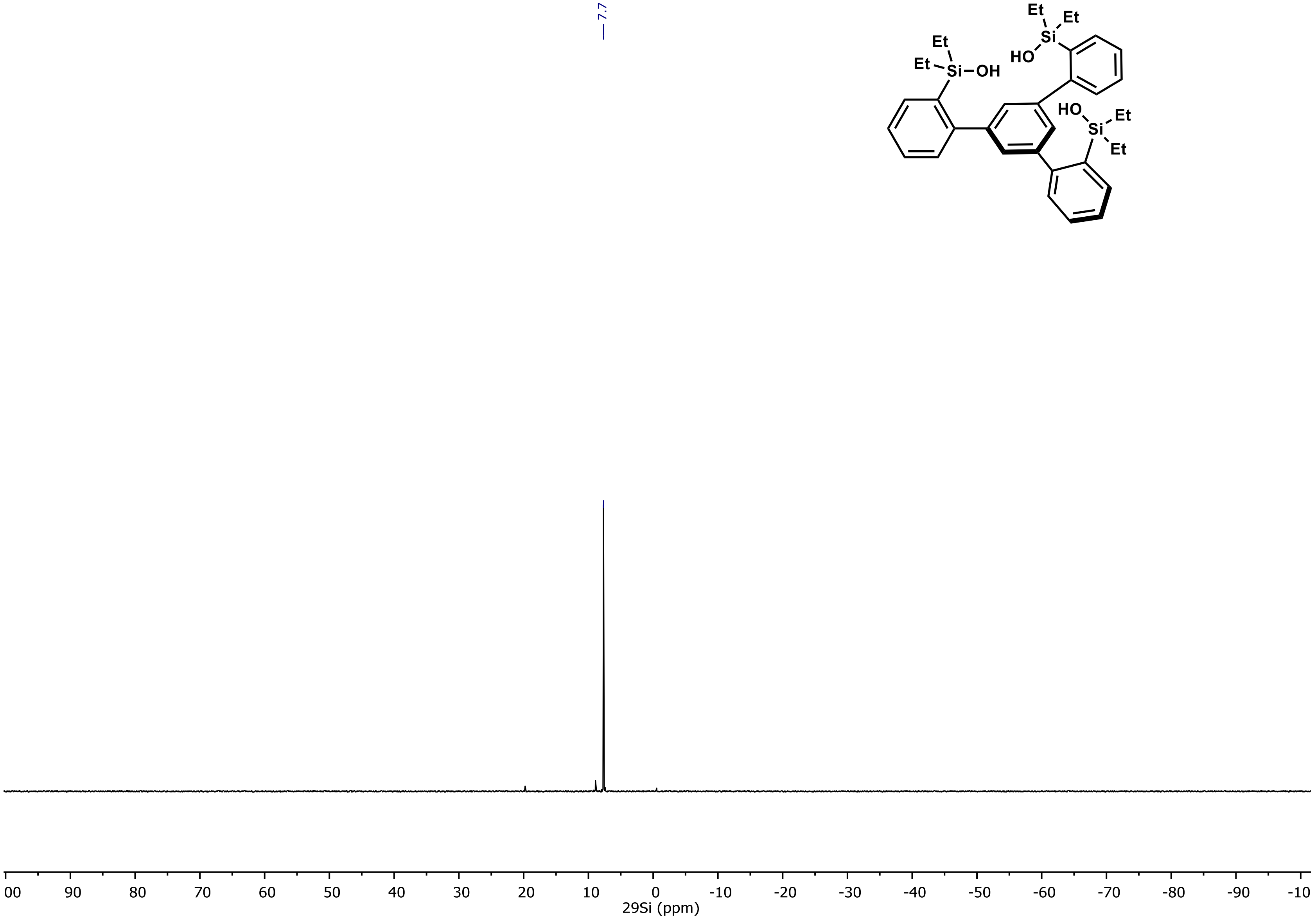

^{1}H NMR of (5'-(2-(diisobutylsilyl)phenyl)-[1,1':3',1''-terphenyl]-2,2''-diyl)bis(diisobutylsilane) (S2), 600 MHz, $CDCl_3$, 25ºC

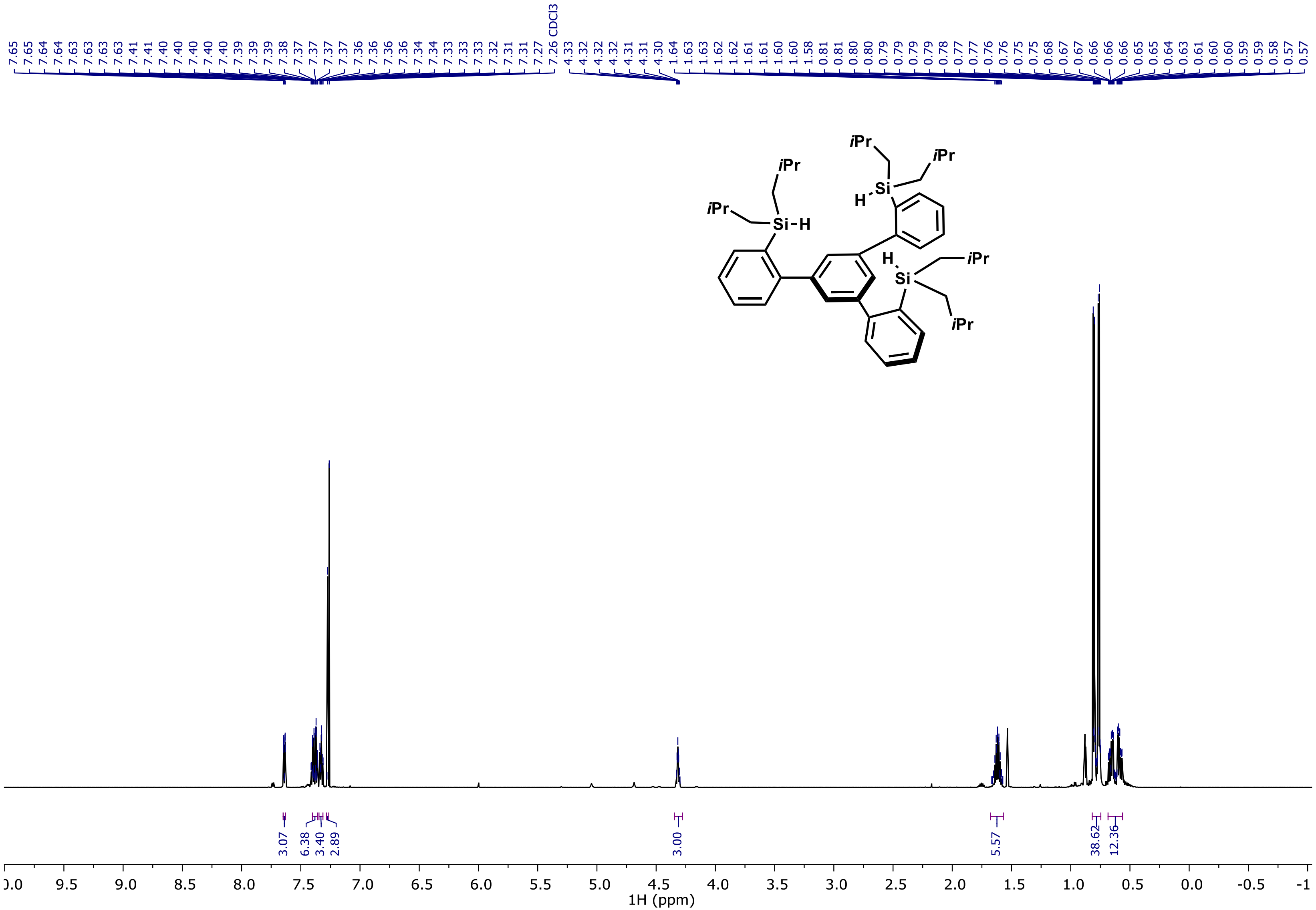

^{13}C NMR of (5'-(2-(diisobutylsilyl)phenyl)-[1,1':3',1''-terphenyl]-2,2''-diyl)bis(diisobutylsilane) (S2), 151 MHz, $CDCl_3$, 25ºC

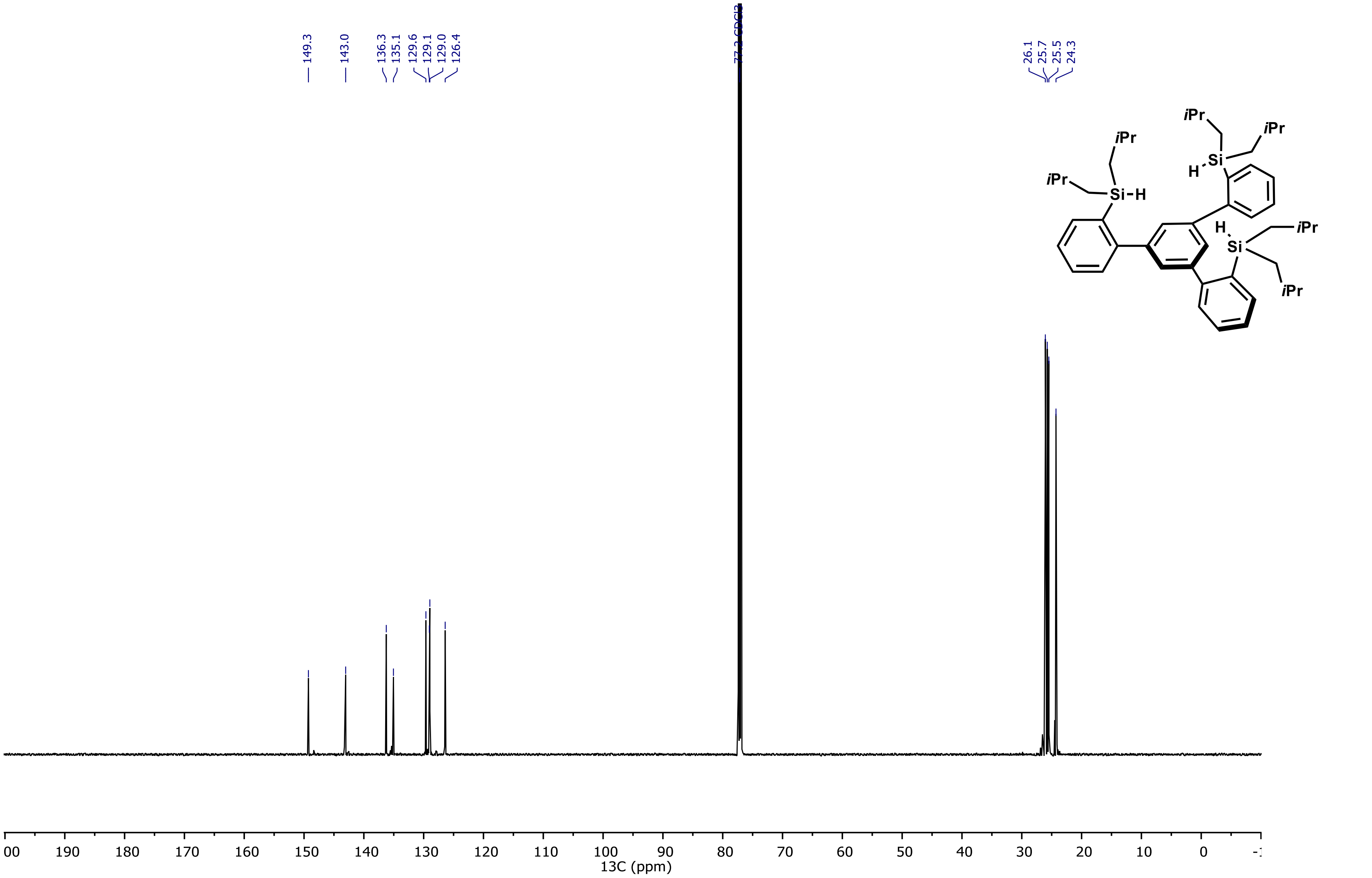

^{29}Si NMR of (5'-(2-(diisobutylsilyl)phenyl)-[1,1':3',1''-terphenyl]-2,2''-diyl)bis(diisobutylsilane) (S2), 119 MHz, $CDCl_3$, 25ºC

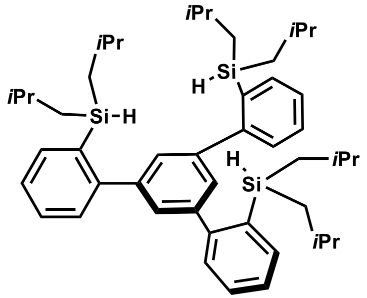

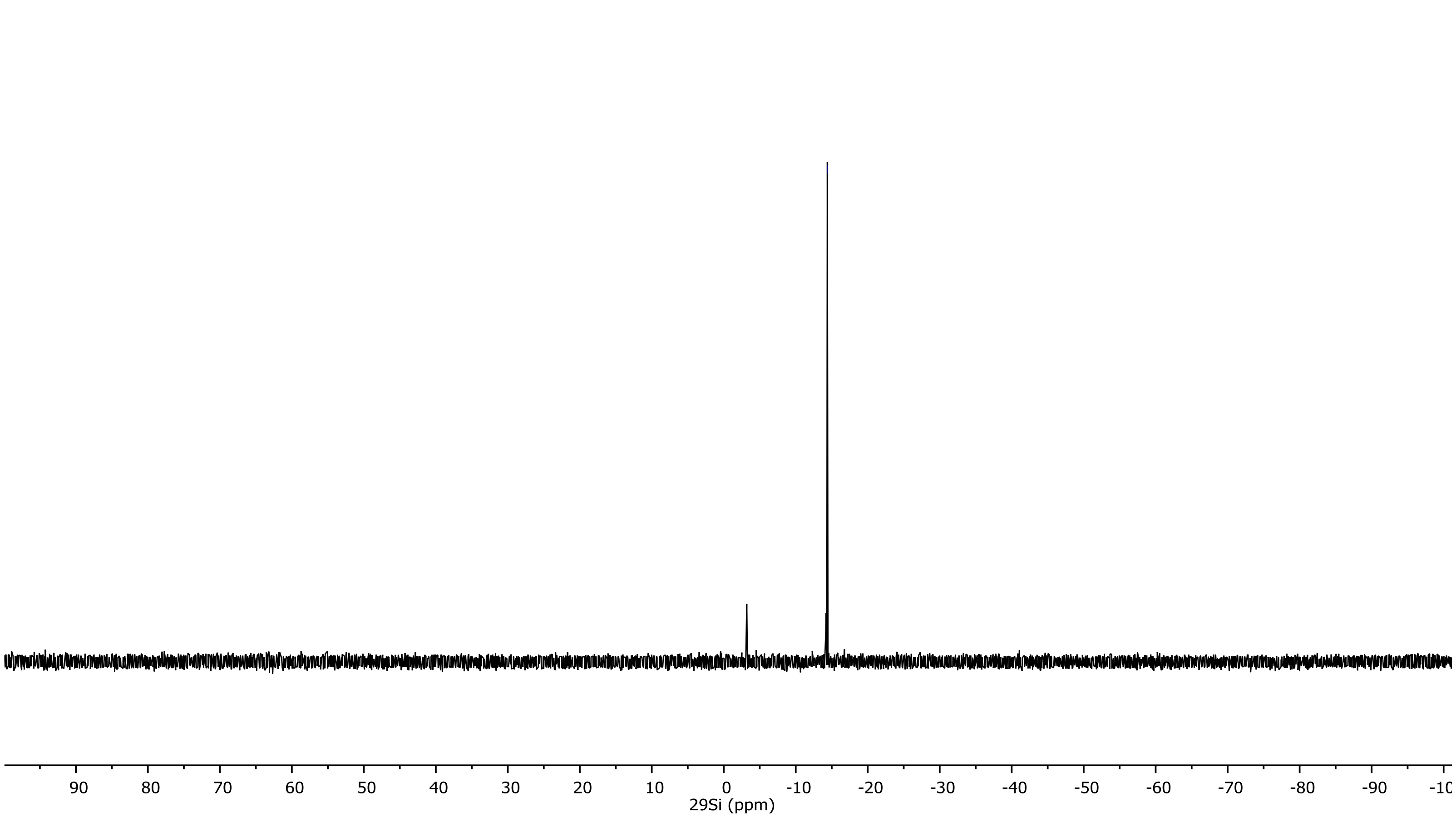

^{1}H NMR of Ligand 15c, 600 MHz, $CDCl_3$, 25ºC

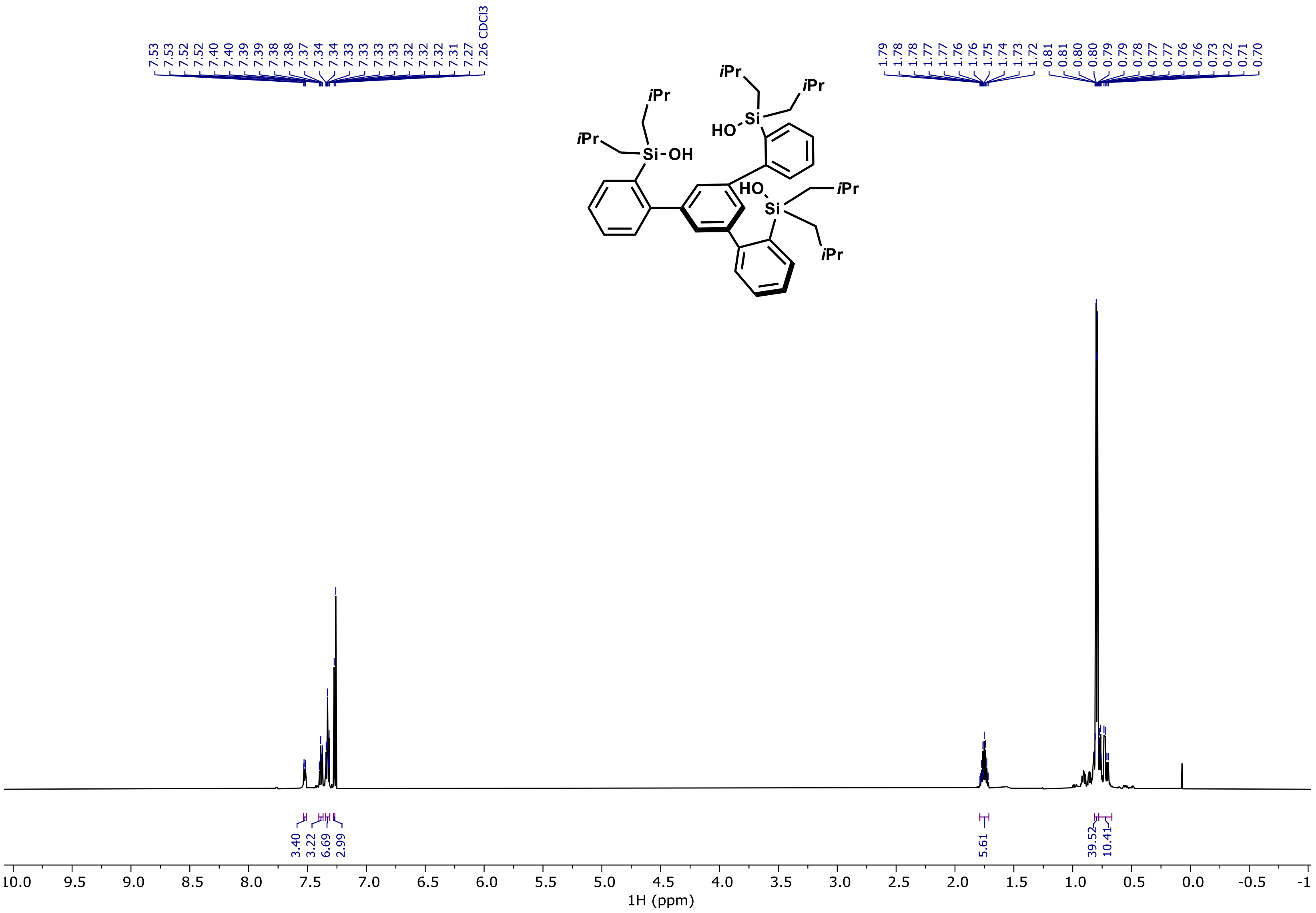

^{13}C NMR of Ligand 15c, 151 MHz, $CDCl_3$, 25ºC

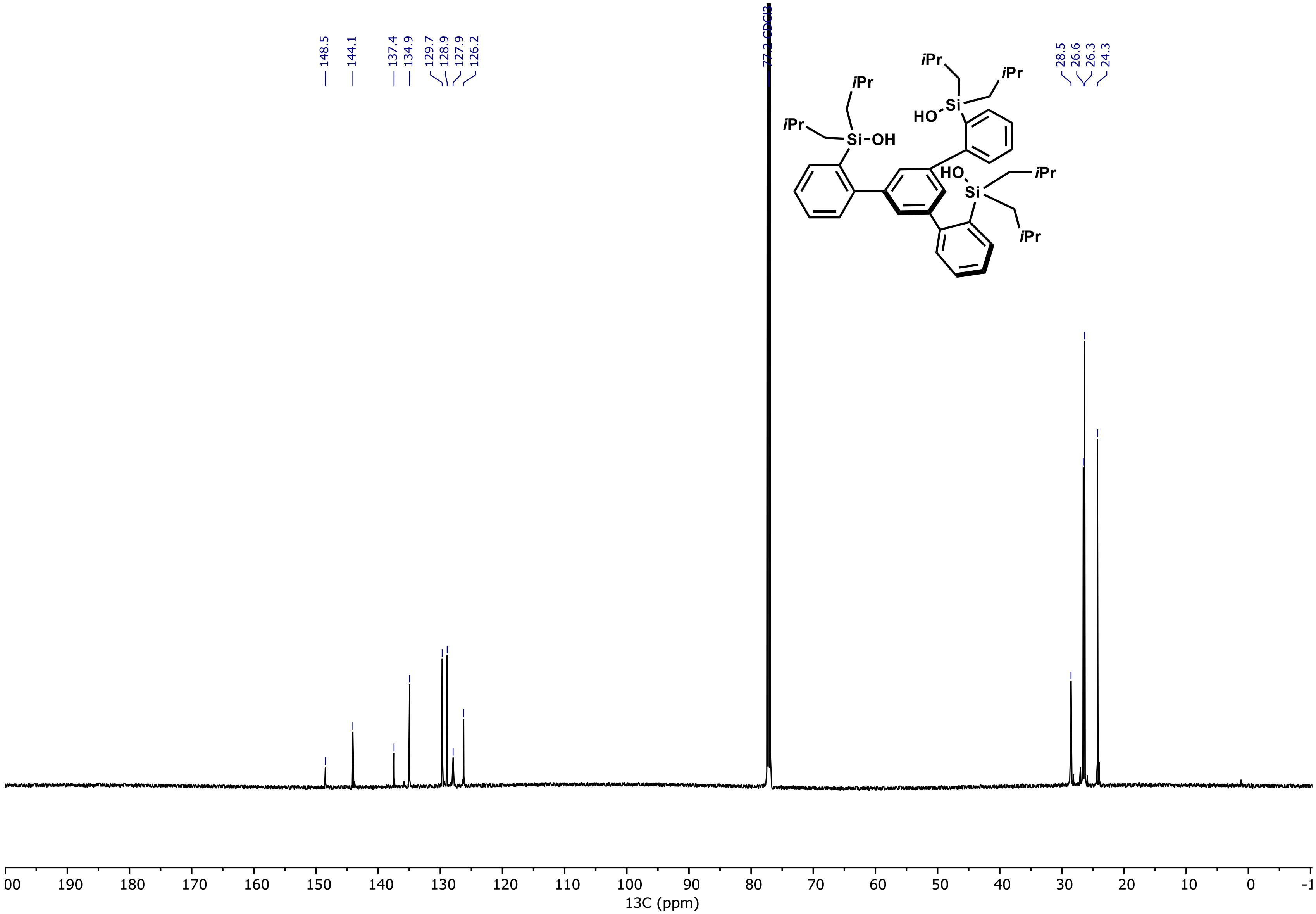

^{29}Si NMR of Ligand 15c, 119 MHz, $CDCl_3$, 25ºC

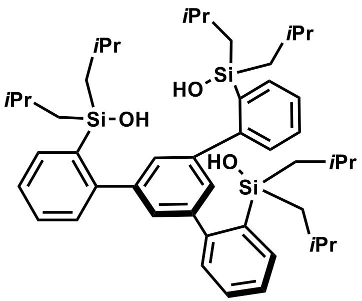

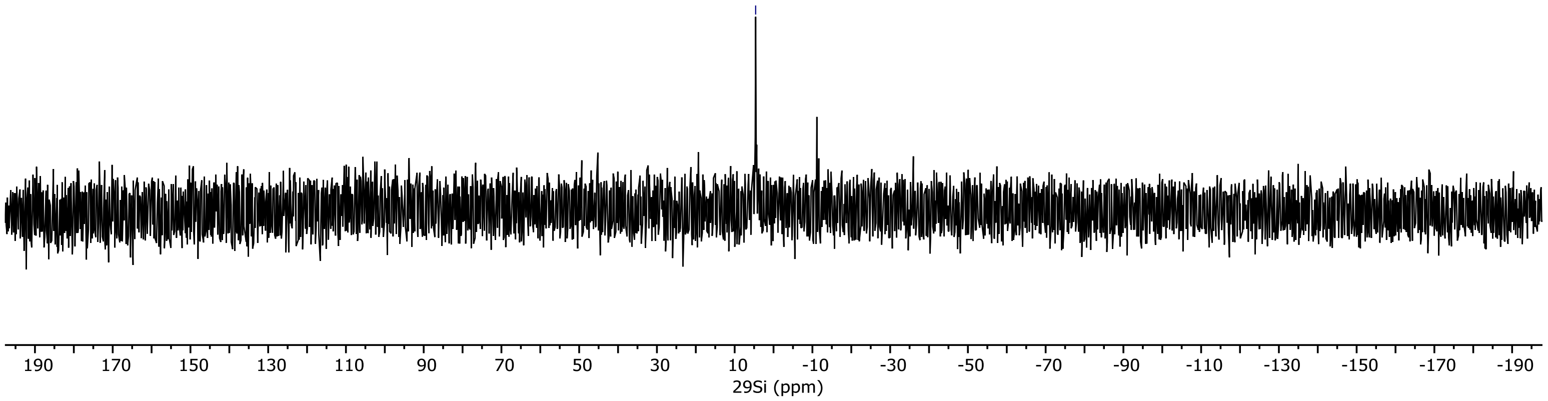

^{1}H NMR of Ligand 22, 400 MHz, $CDCl_3$, 25ºC

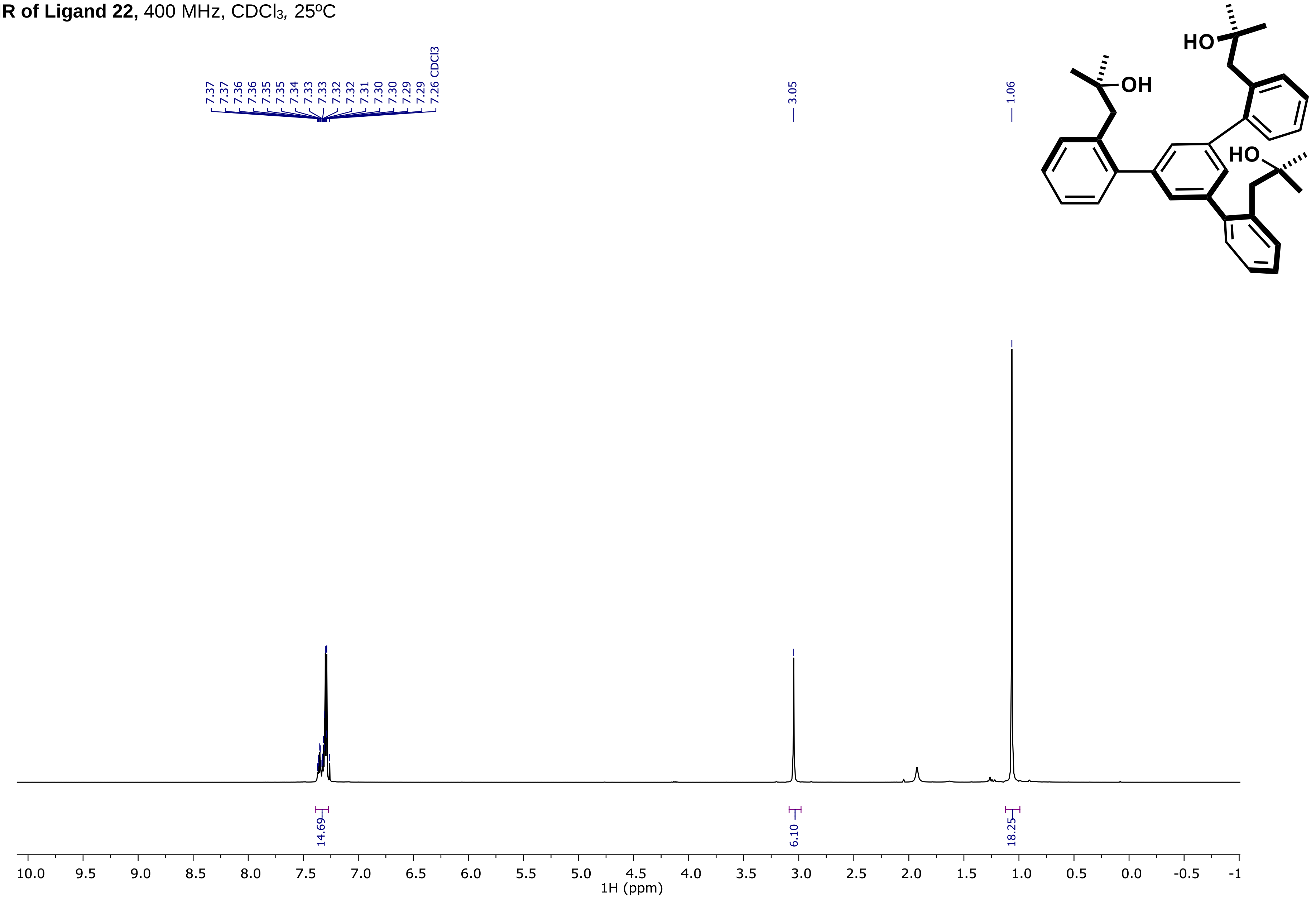

^{13}C NMR of Ligand 22, 101 MHz, $CDCl_3$, 25ºC

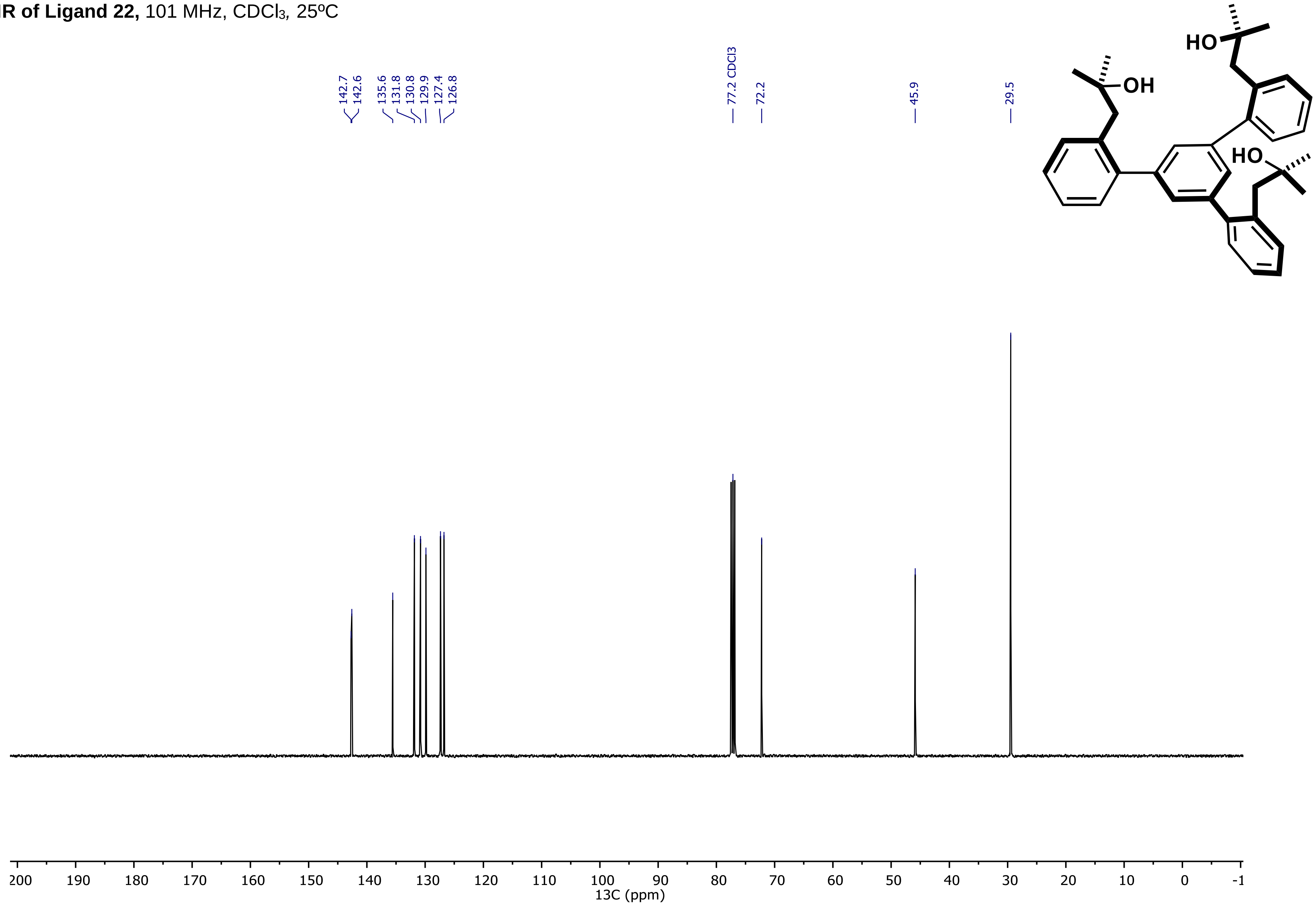

^{1}H NMR of Complex S4, 400 MHz, C_6D_6, 25ºC

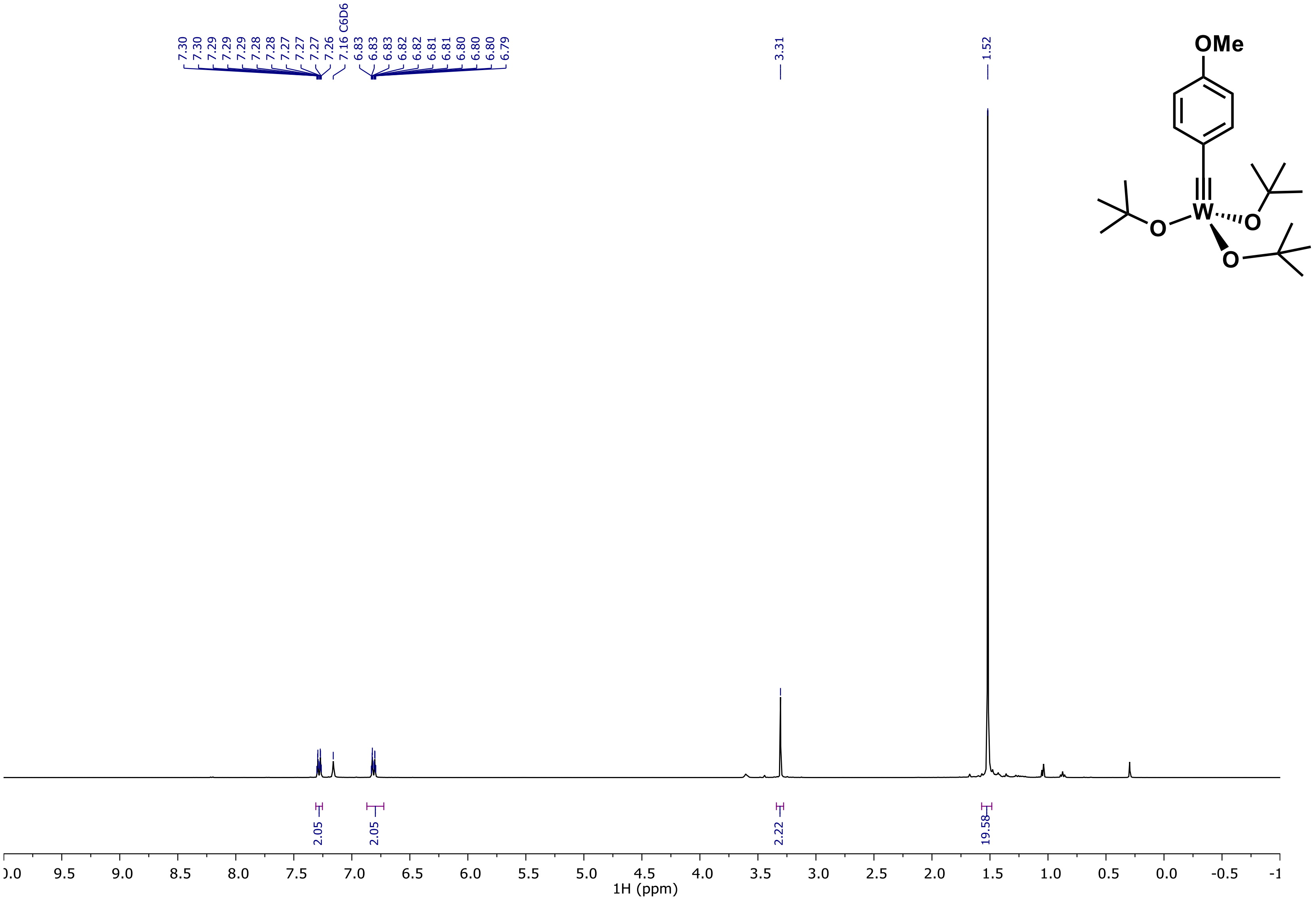

^{13}C NMR of Complex S4 , 101 MHz, C_6D_6, 25ºC

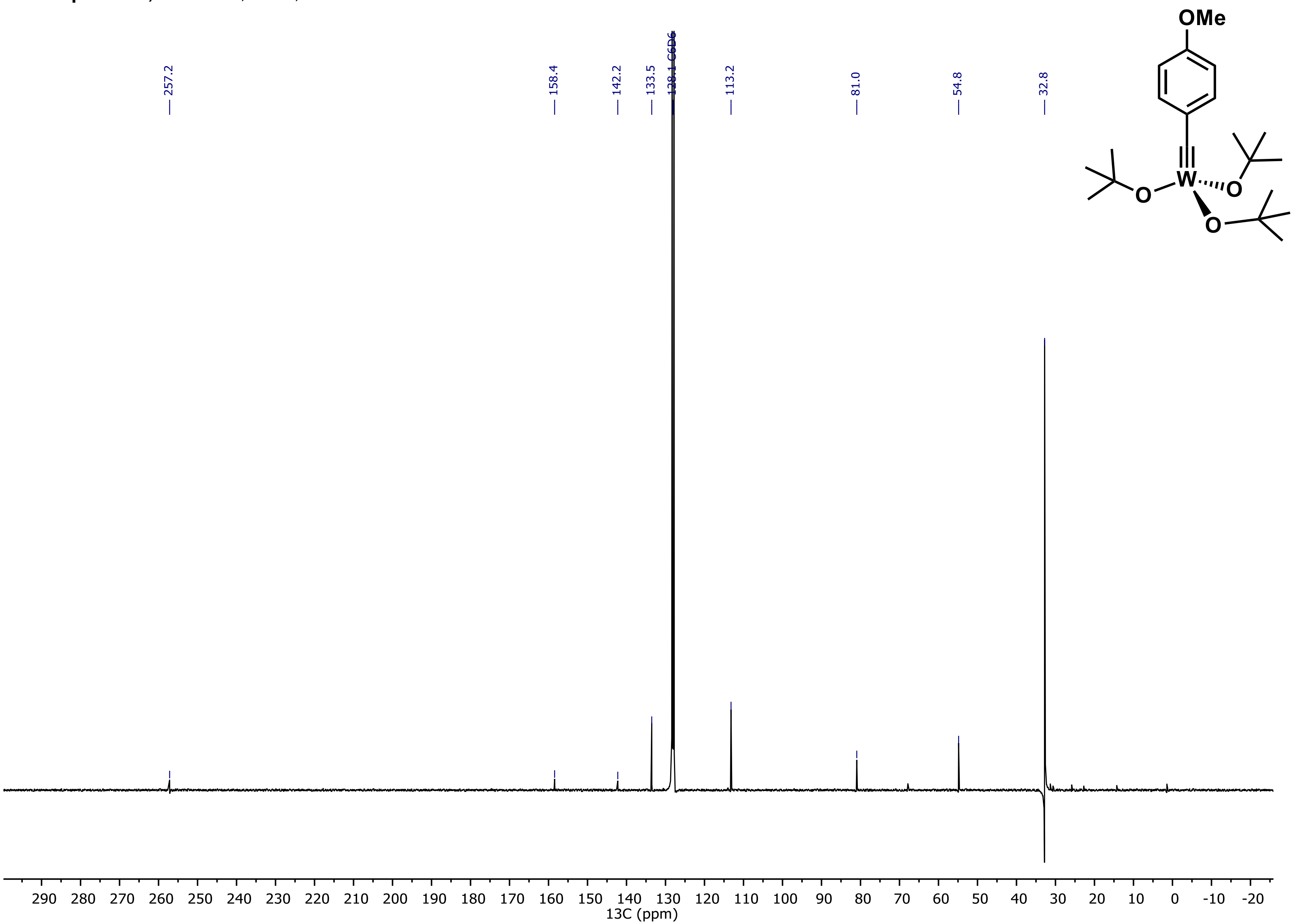

^{1}H NMR of [NMe_4]W$(CO)_5$(COAr) (Ar= 2,6-dimethlyphenyl) S5, 600 MHz, CCl_2D_2, 25ºC

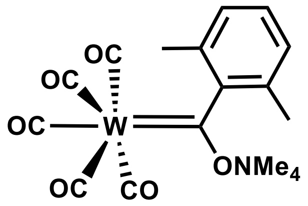

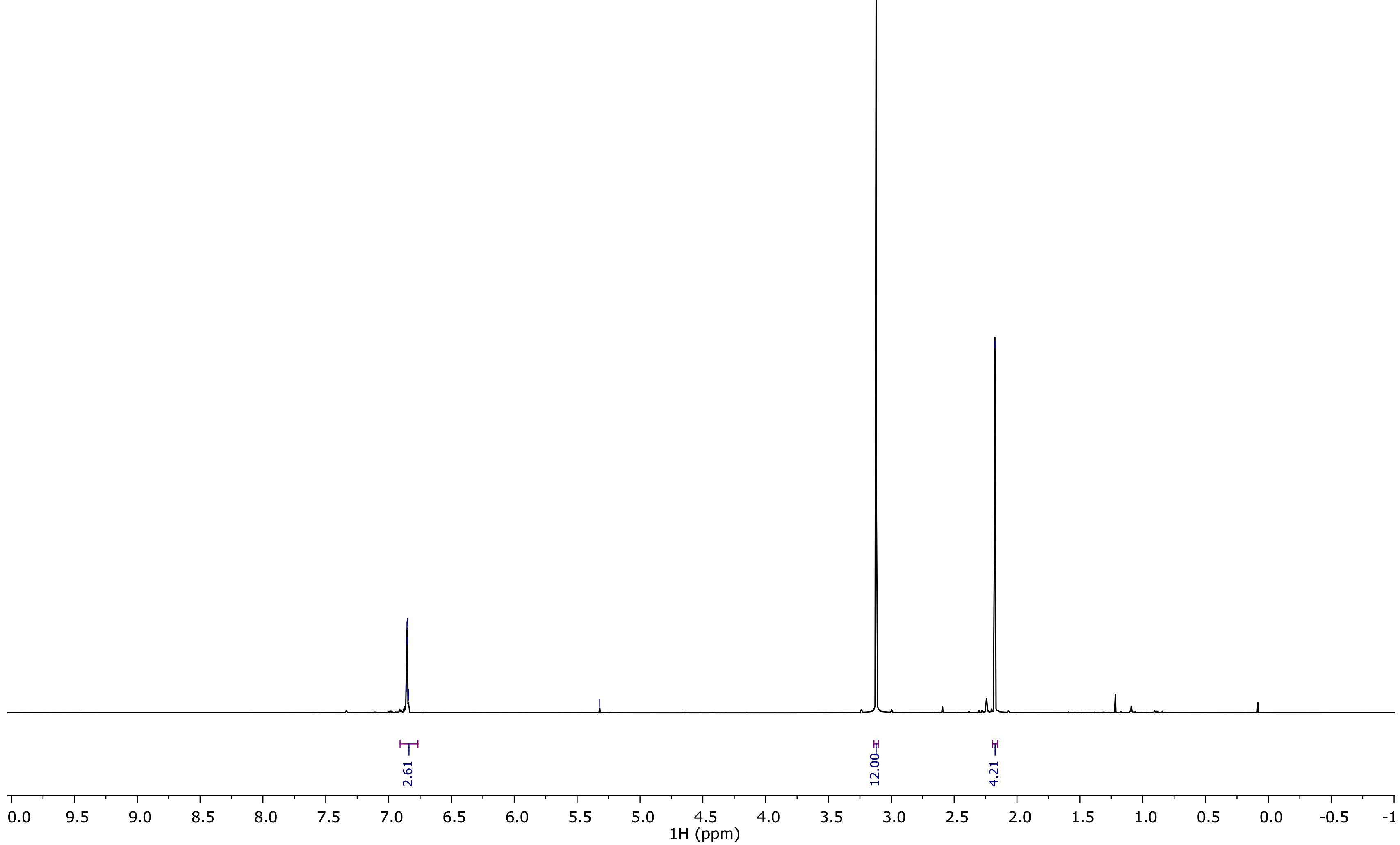

^{13}C NMR of [NMe$_4$]W(CO)$_5$(COAr) (Ar= 2,6-dimethlyphenyl) S5, 151 MHz, CCl_2D_2, 25ºC

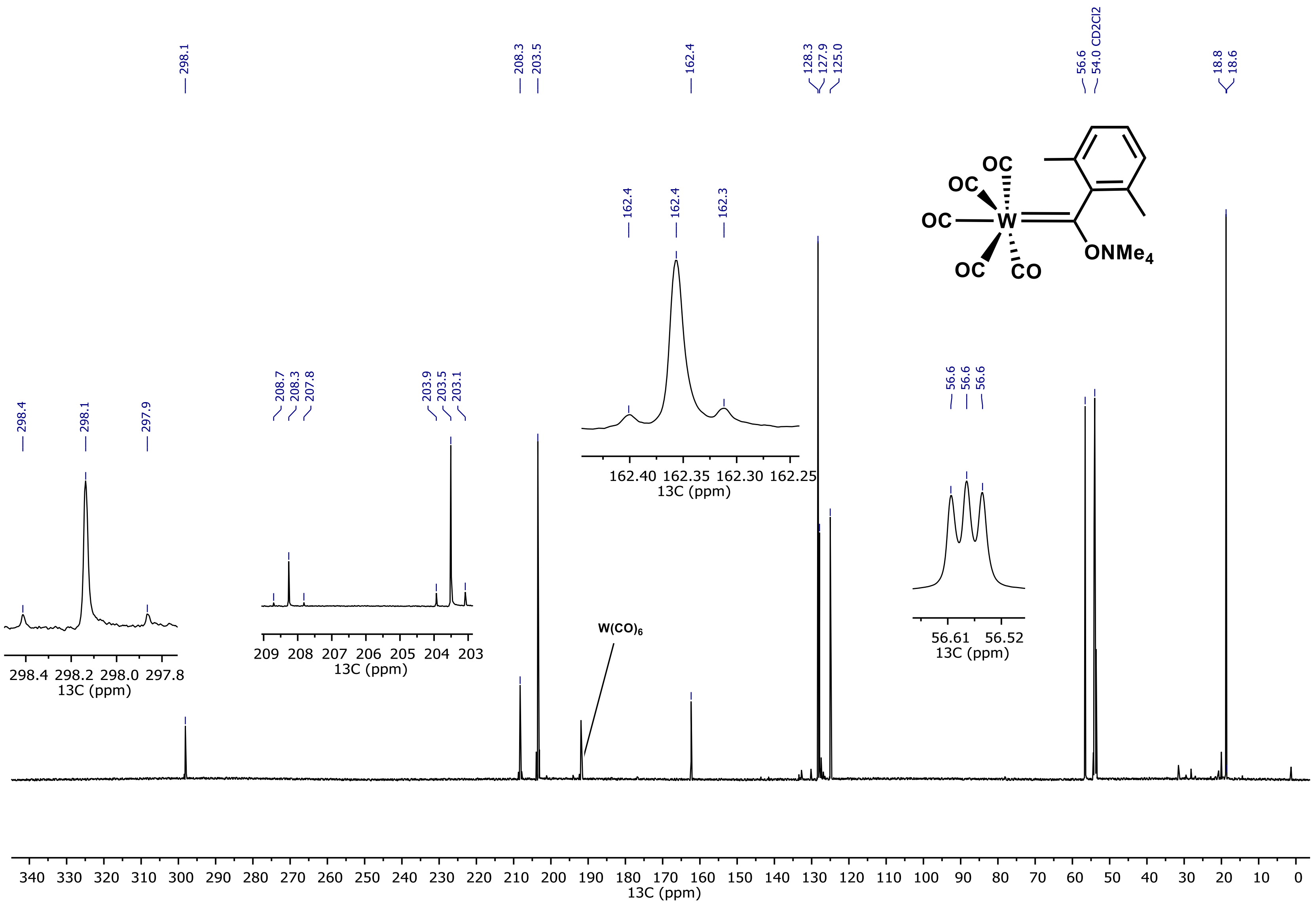

^{1}H NMR of Complex [W(≡CAr)Br_3(dme)] (Ar= 2,6-dimethlyphenyl) S6, 600 MHz, C_6D_6, 25ºC

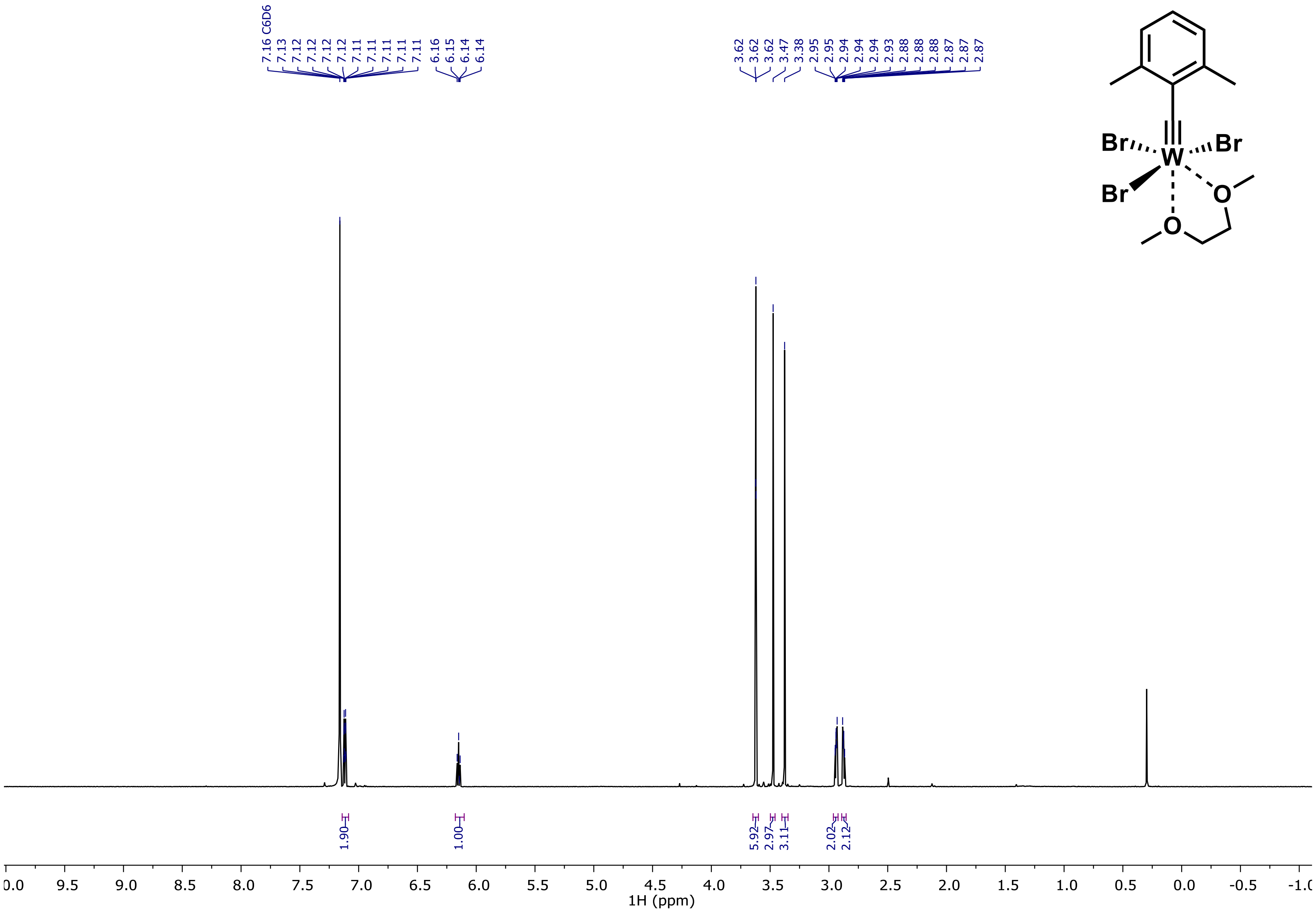

^{13}C NMR of Complex [W(≡CAr)Br$_3$(dme)] (Ar= 2,6-dimethlyphenyl) S6, 151 MHz, C_6D_6, 25ºC

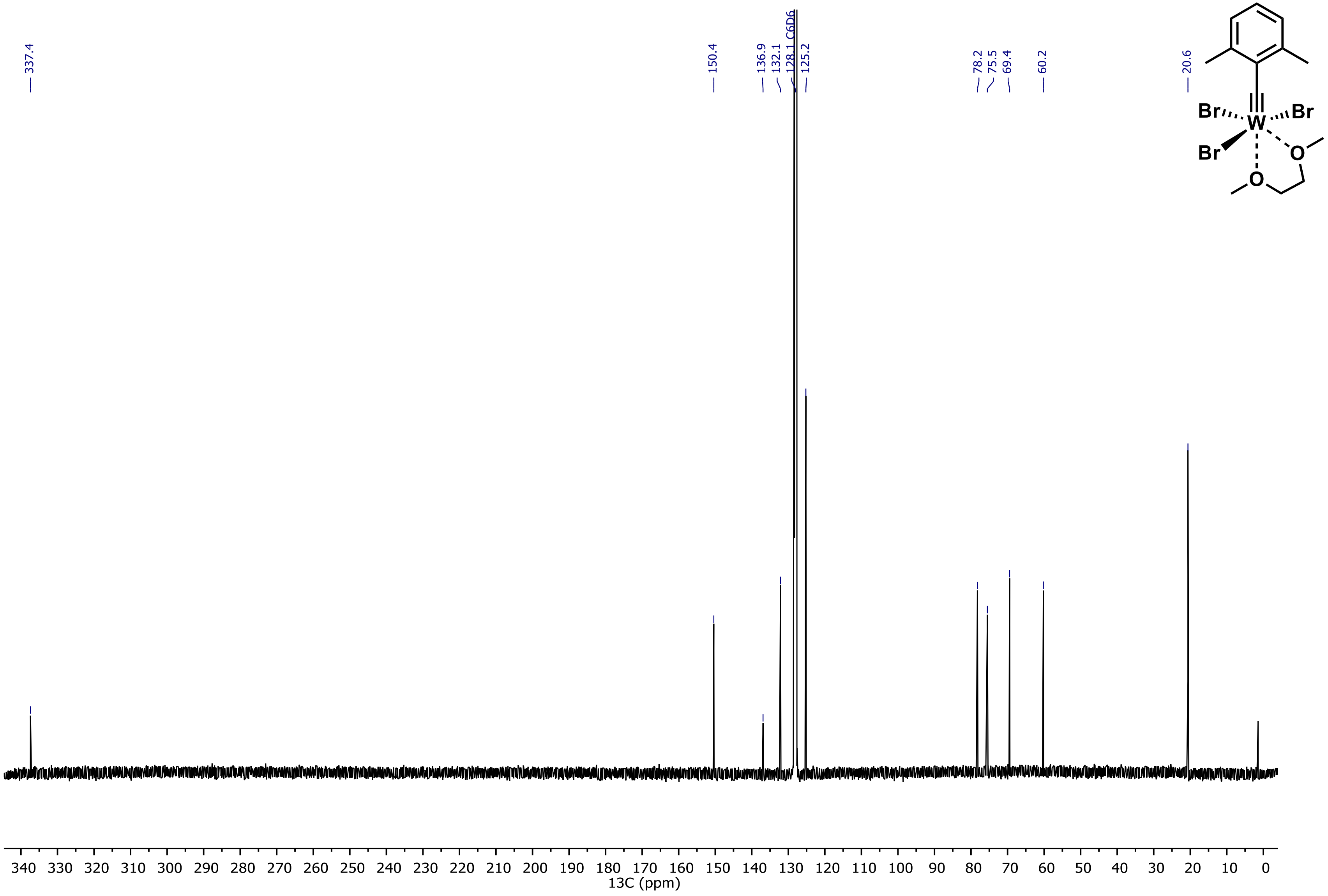

^{1}H NMR of Complex 4b, 600 MHz, $C_6D_5CD_3$, 25ºC

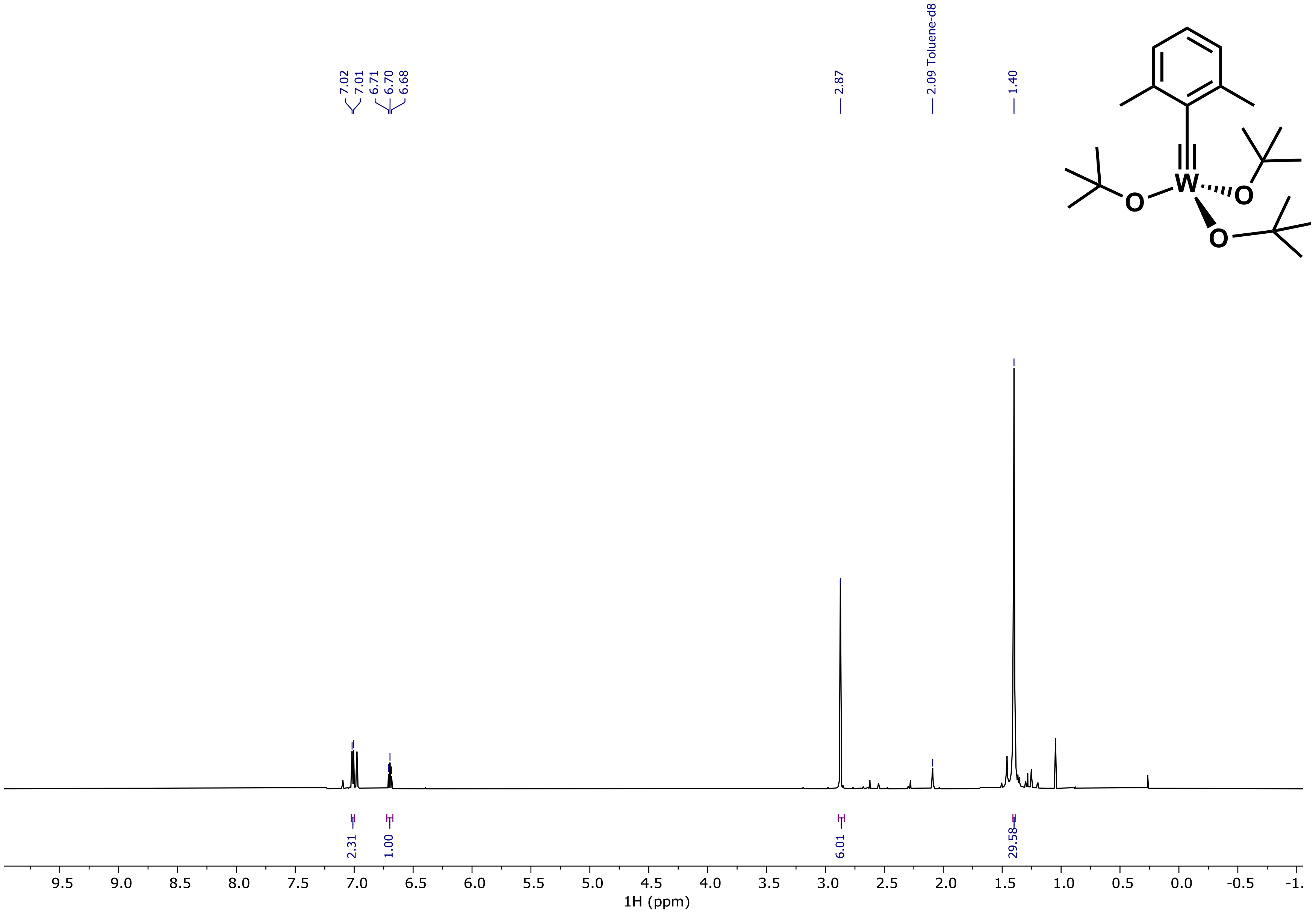

^{13}C NMR of Complex 4b, 151 MHz, $C_6D_5CD_3$, 25ºC

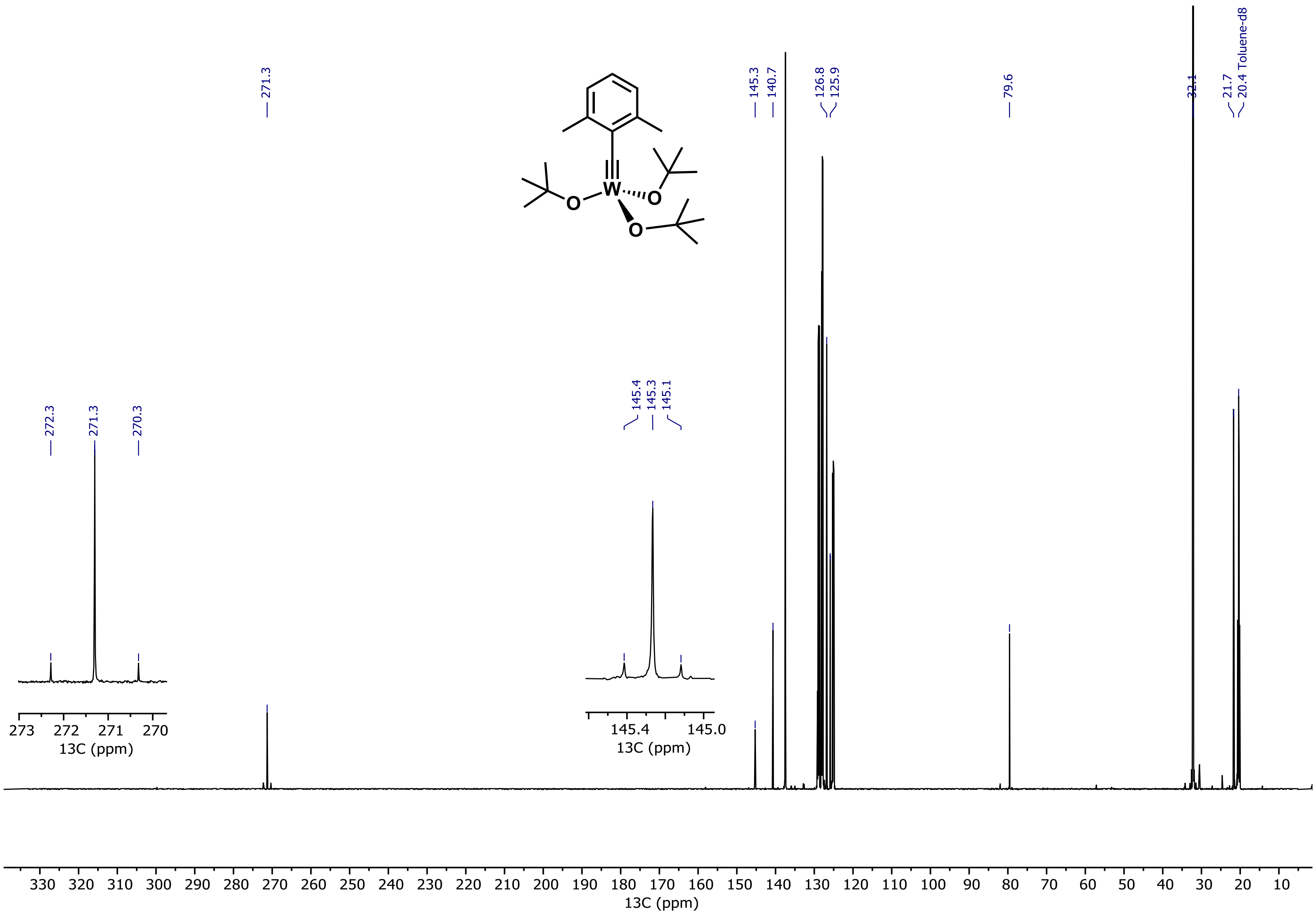

1D ^{183}W NMR of Complex 4b, 151 MHz, $C_6D_5CD_3$, 25ºC

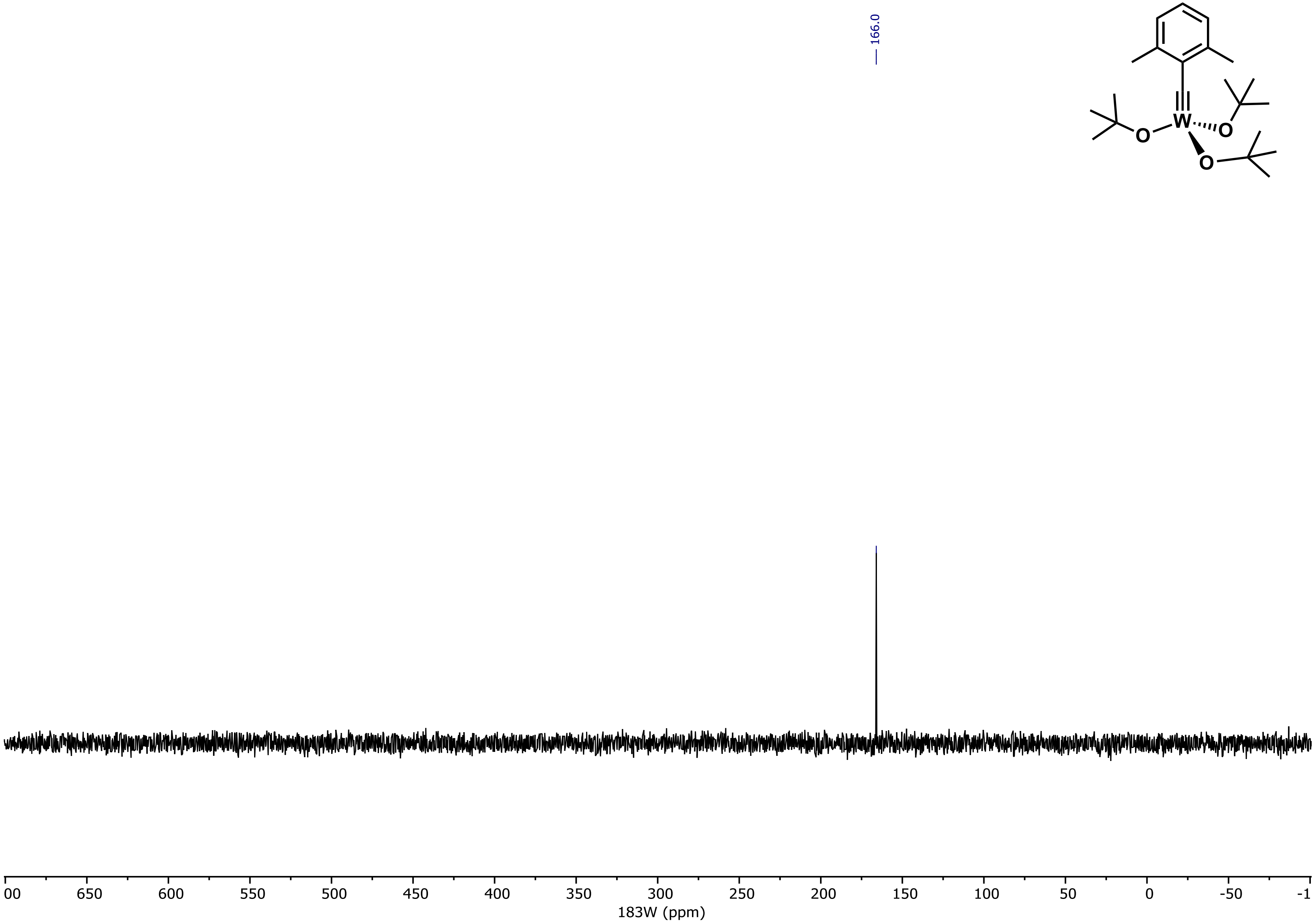

^{1}H NMR of Complex 16a, 600 MHz, $C_6D_5CD_3$, 25ºC

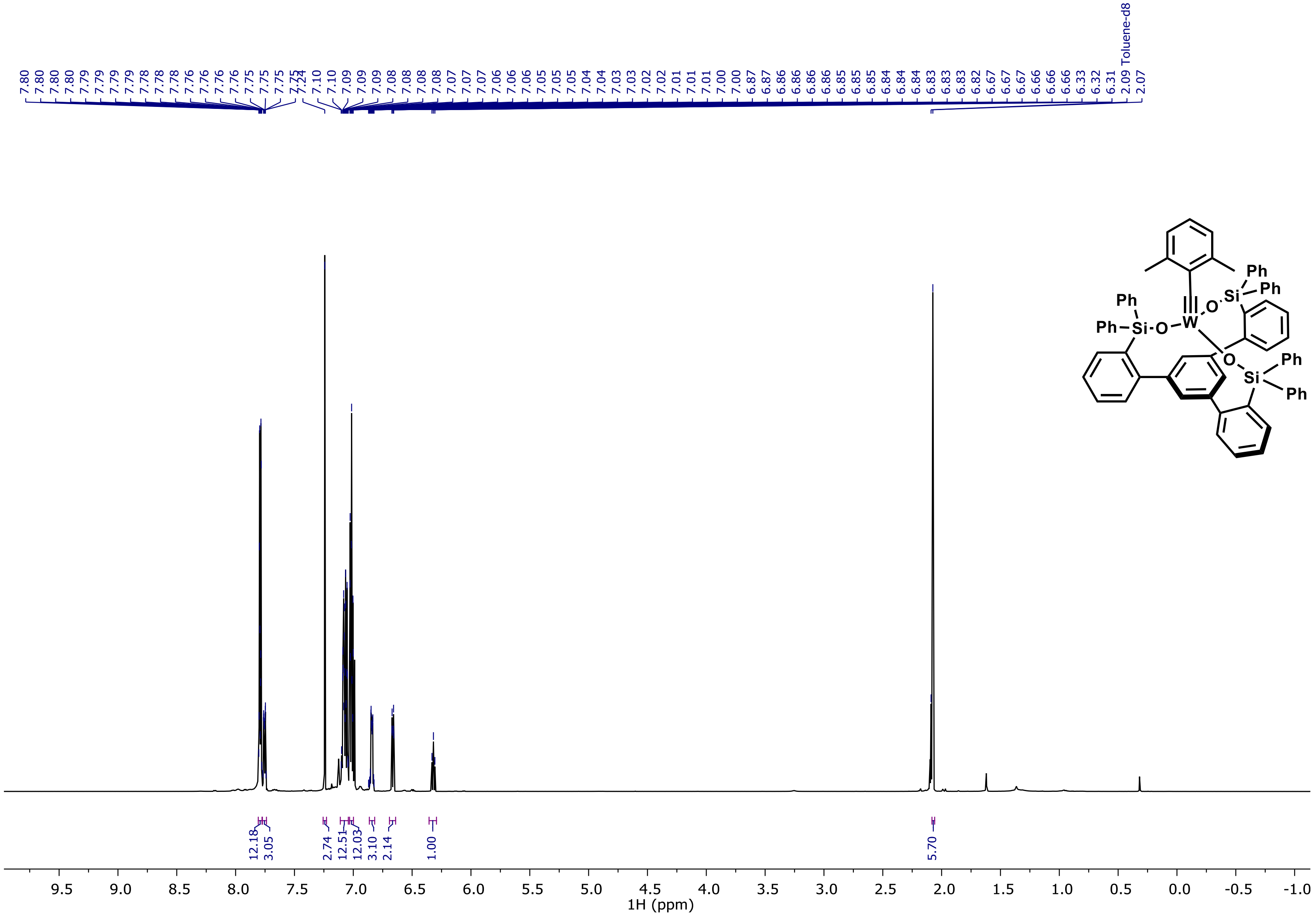

^{13}C NMR of Complex 16a, 151 MHz, $C_6D_5CD_3$, 25ºC

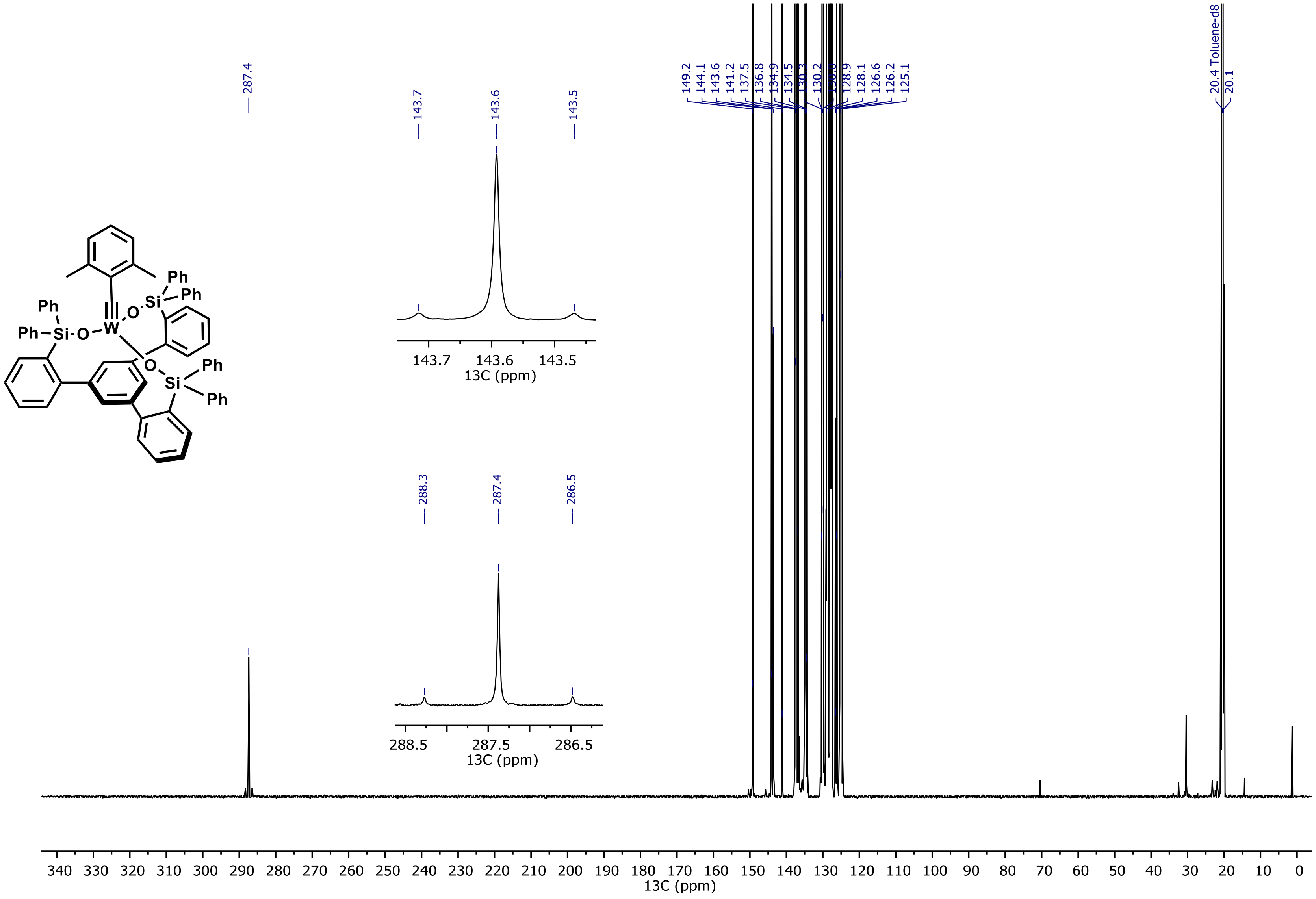

^{29}Si NMR of Complex 16a, 119 MHz, $C_6D_5CD_3$, 25ºC

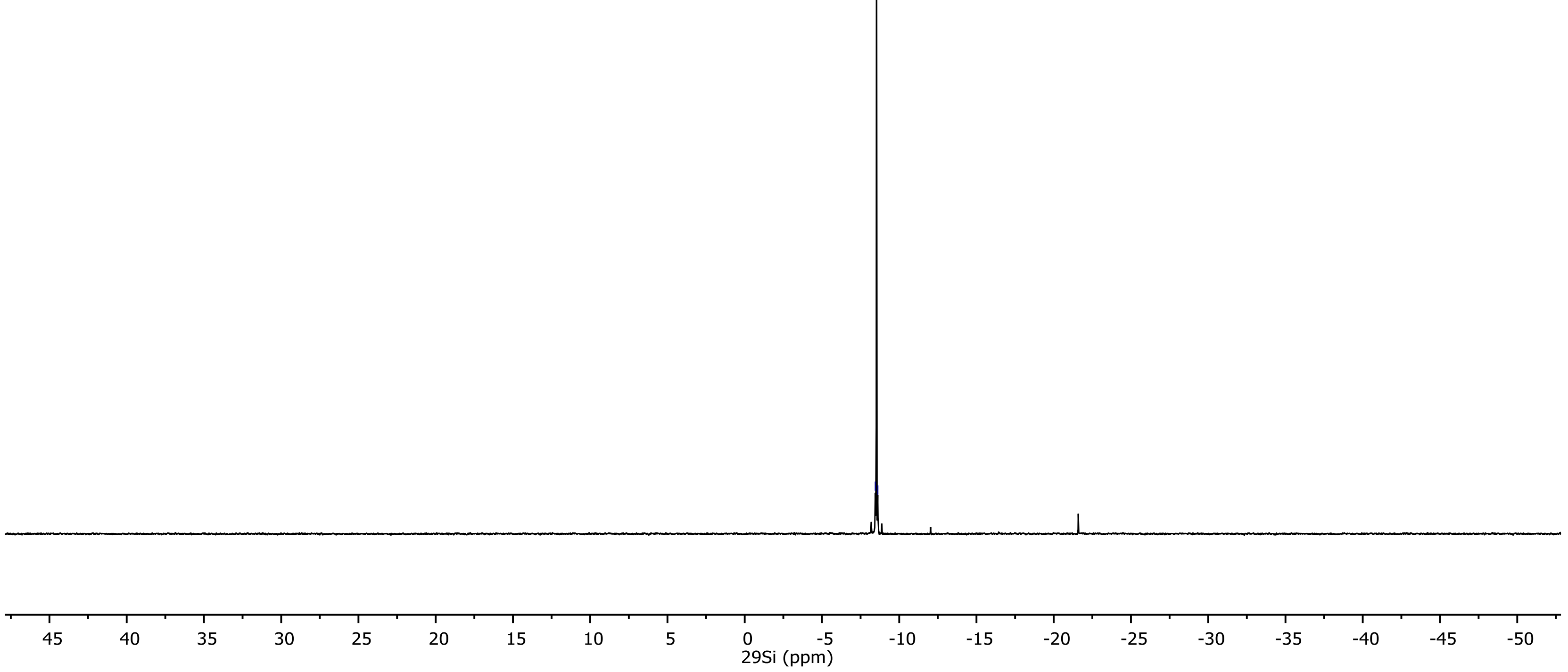

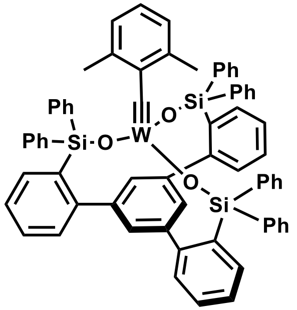

Folding Screening: Comparison of different offsets with two different sweep width: ^{1}H-^{183}W HMBC NMR of Complex 16a, 400 MHz, $C_6D_5CD_3$, 25ºC

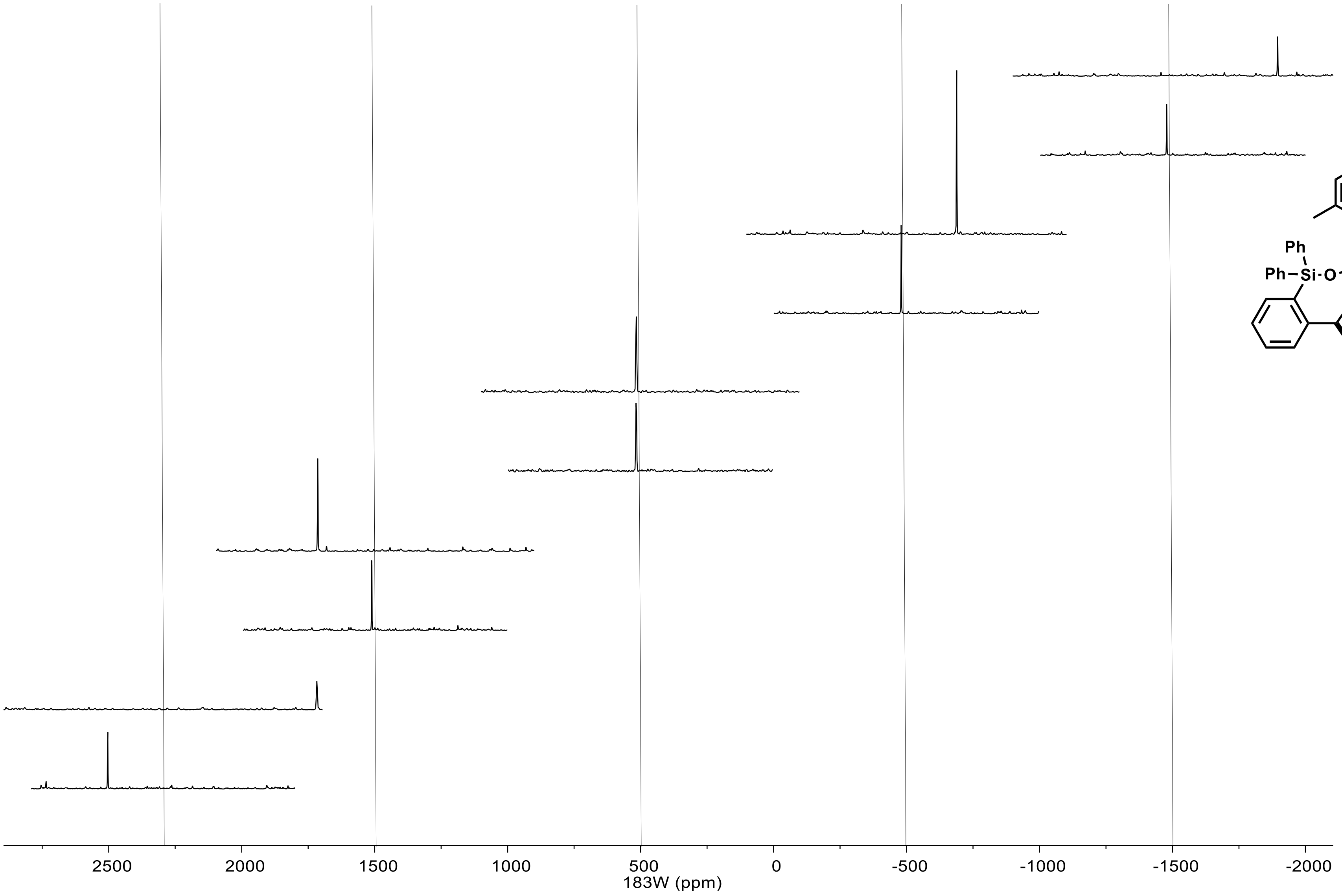

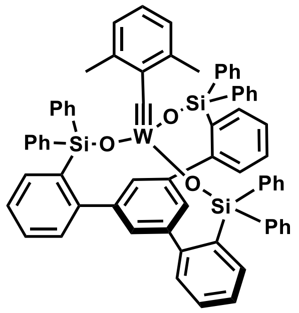

^{1}H-^{183}W HMBC NMR of Complex 16a, 400 MHz, $C_6D_5CD_3$, 25ºC

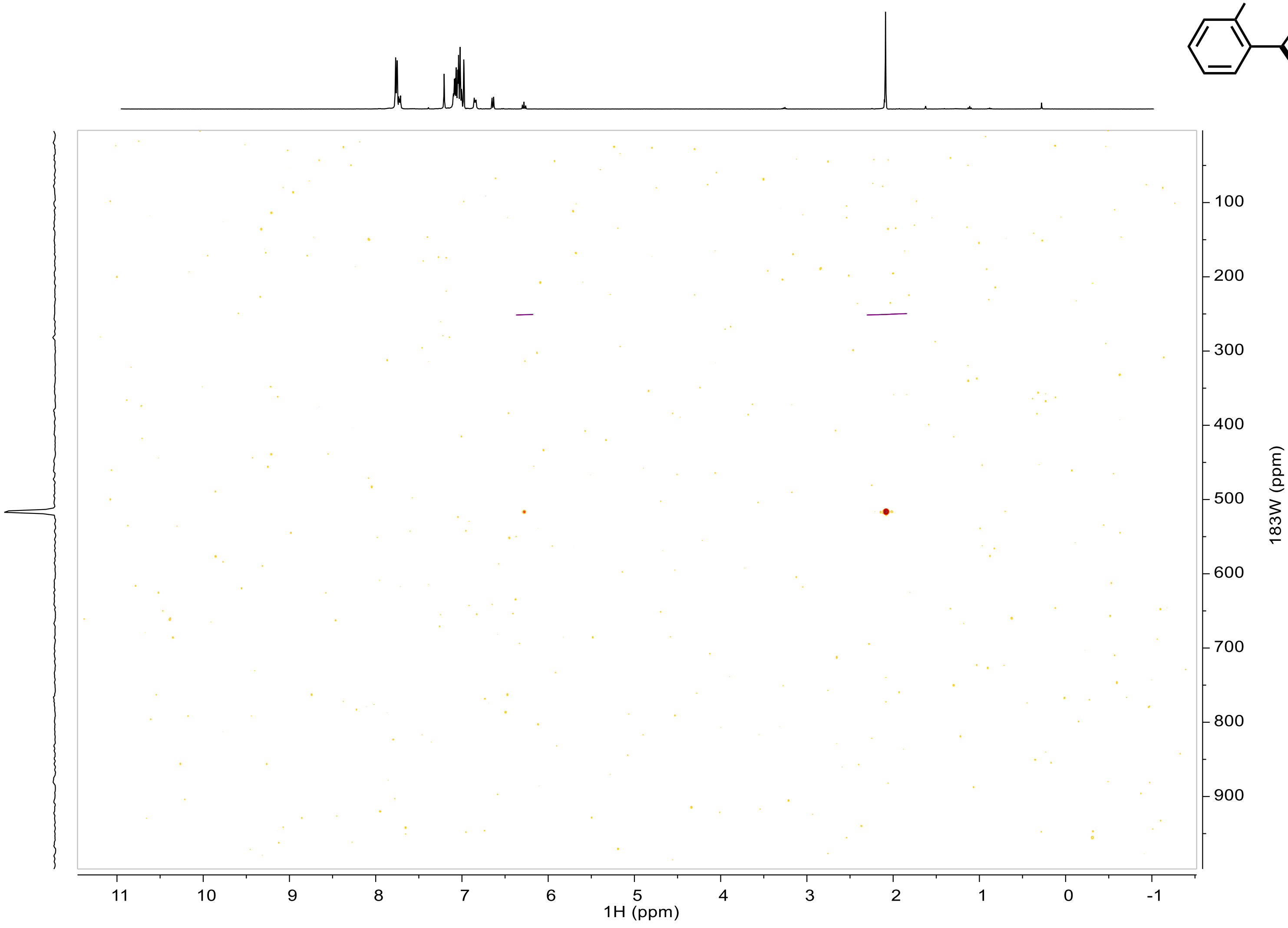

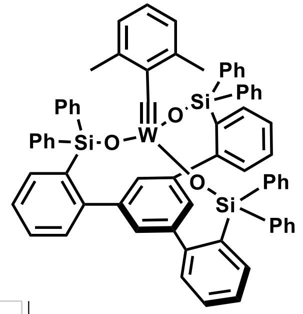

^{183}W NMR Projection created from 2D-HMBC Experiment of Complex 16a, 17 MHz, $C_6D_5CD_3$, 25ºC

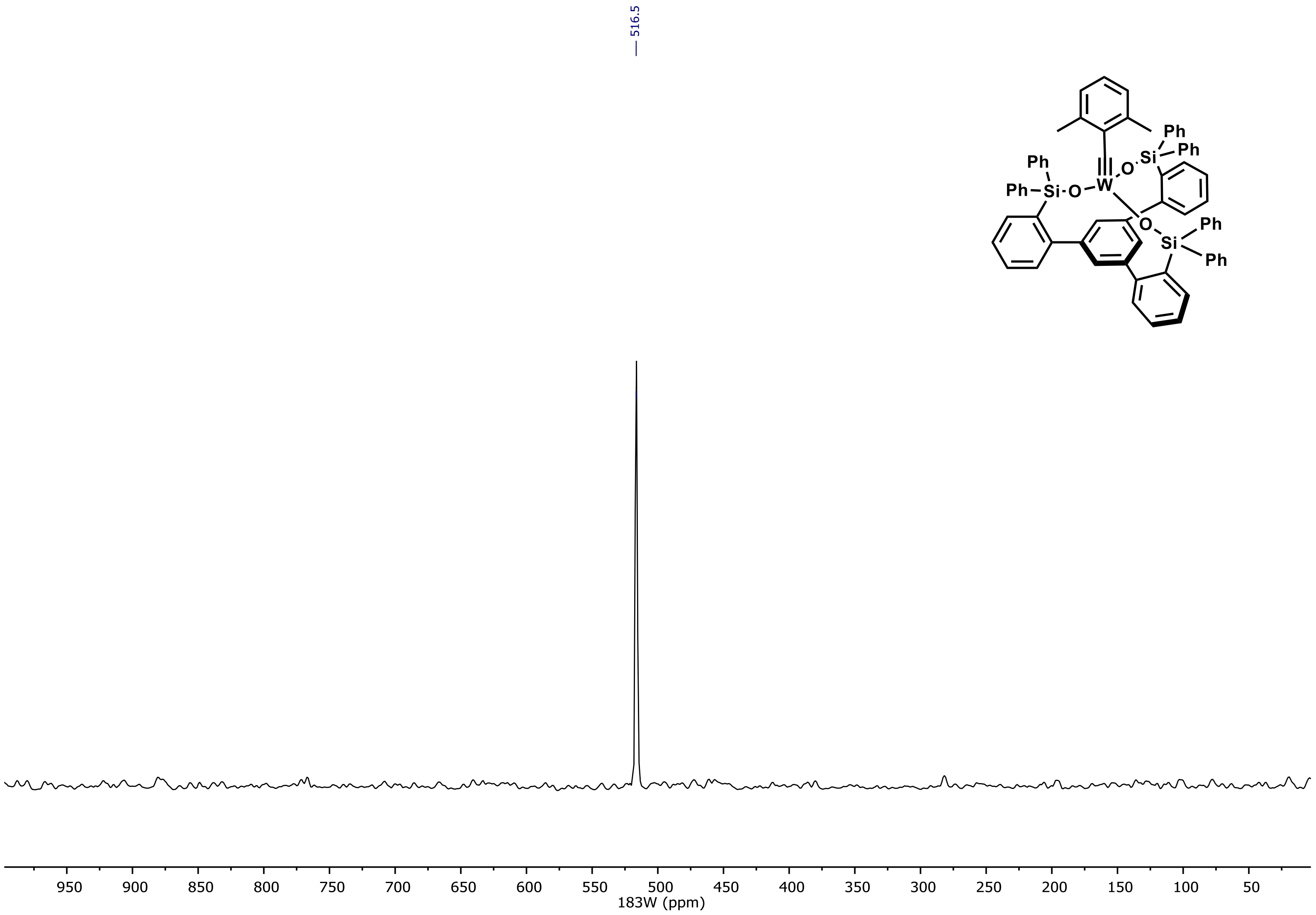

^{1}H NMR of Complex 16b, 600 MHz, $C_6D_5CD_3$, 25ºC

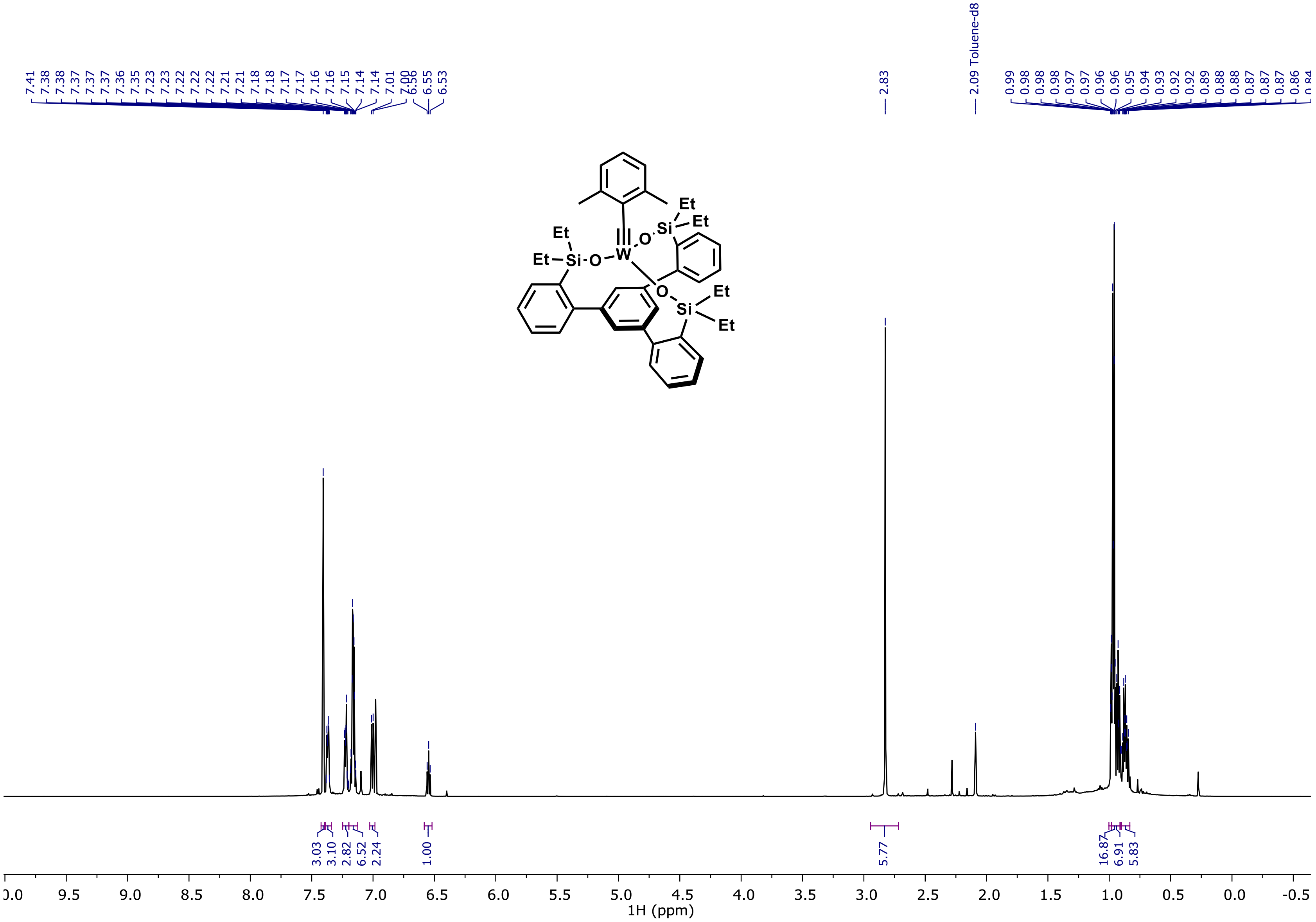

^{13}C NMR of Complex 16b, 151 MHz, $C_6D_5CD_3$, 25ºC

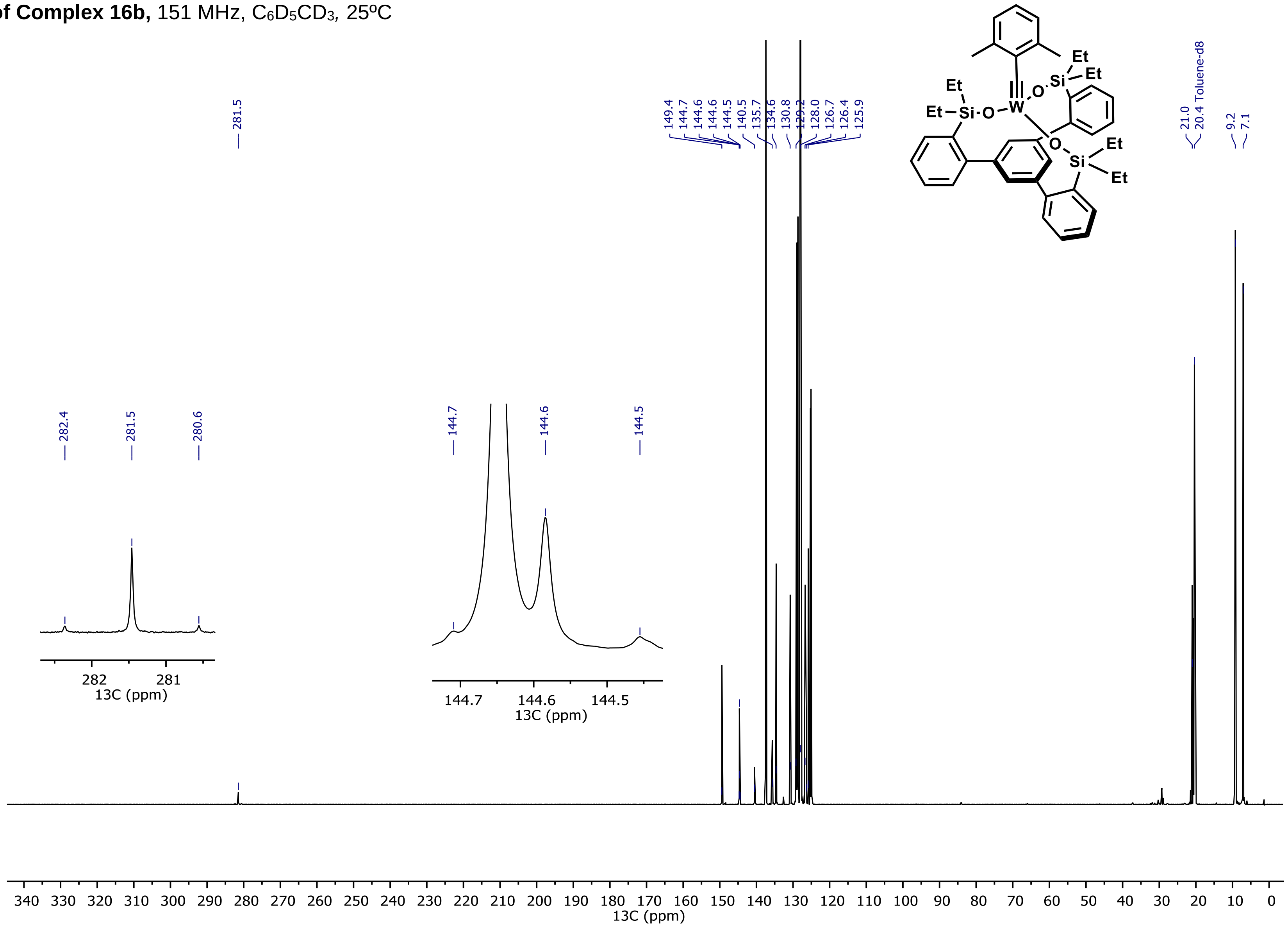

^{29}Si NMR of Complex 16b , 119 MHz, $C_6D_5CD_3$, 25ºC

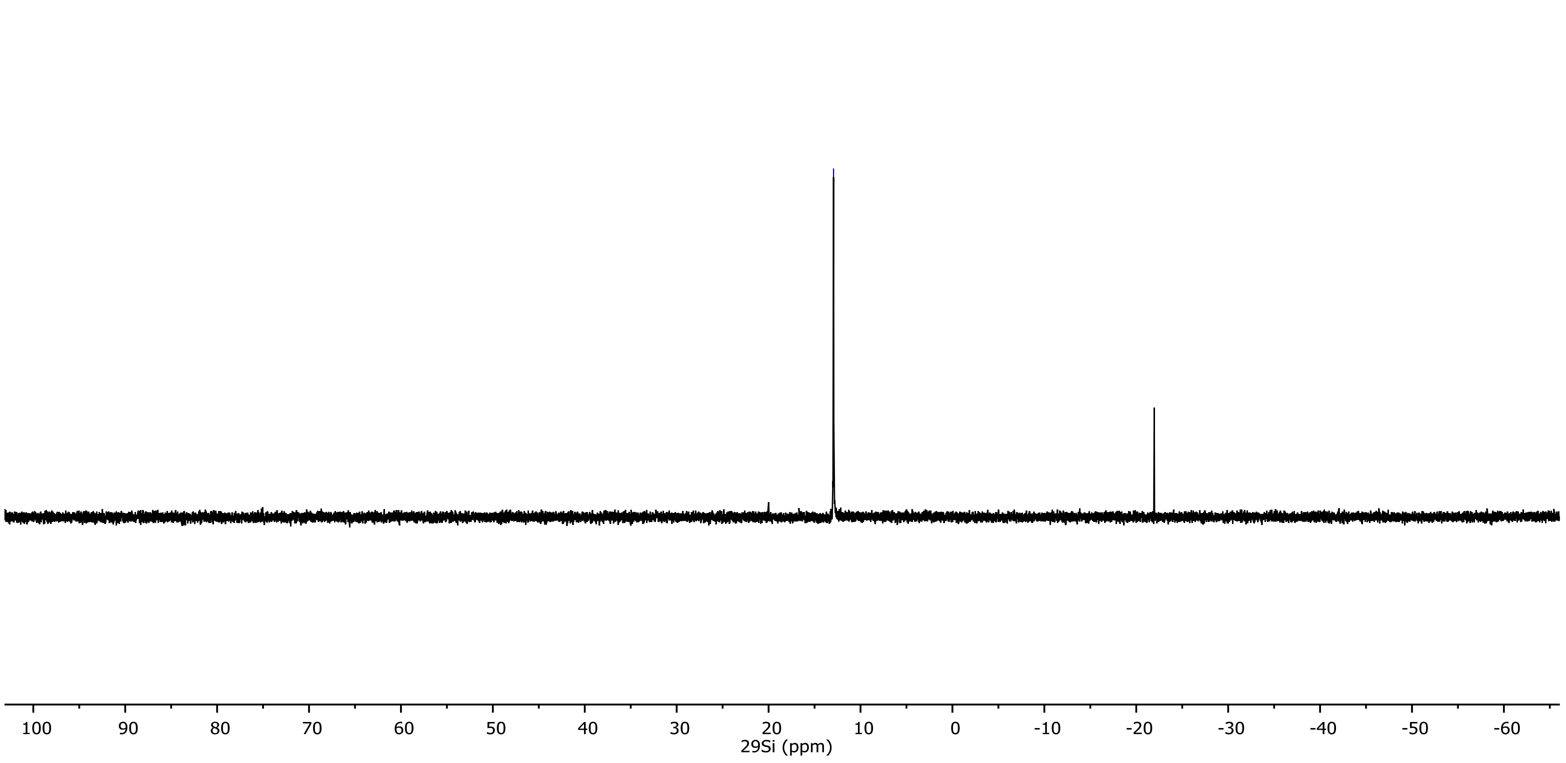

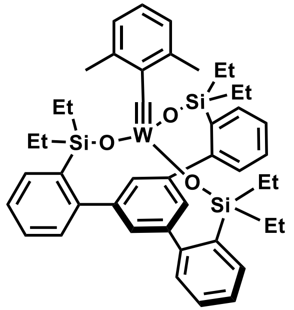

^{1}H-^{183}W HMBC NMR of Complex 16b, 400 MHz, $C_6D_5CD_3$, 25ºC

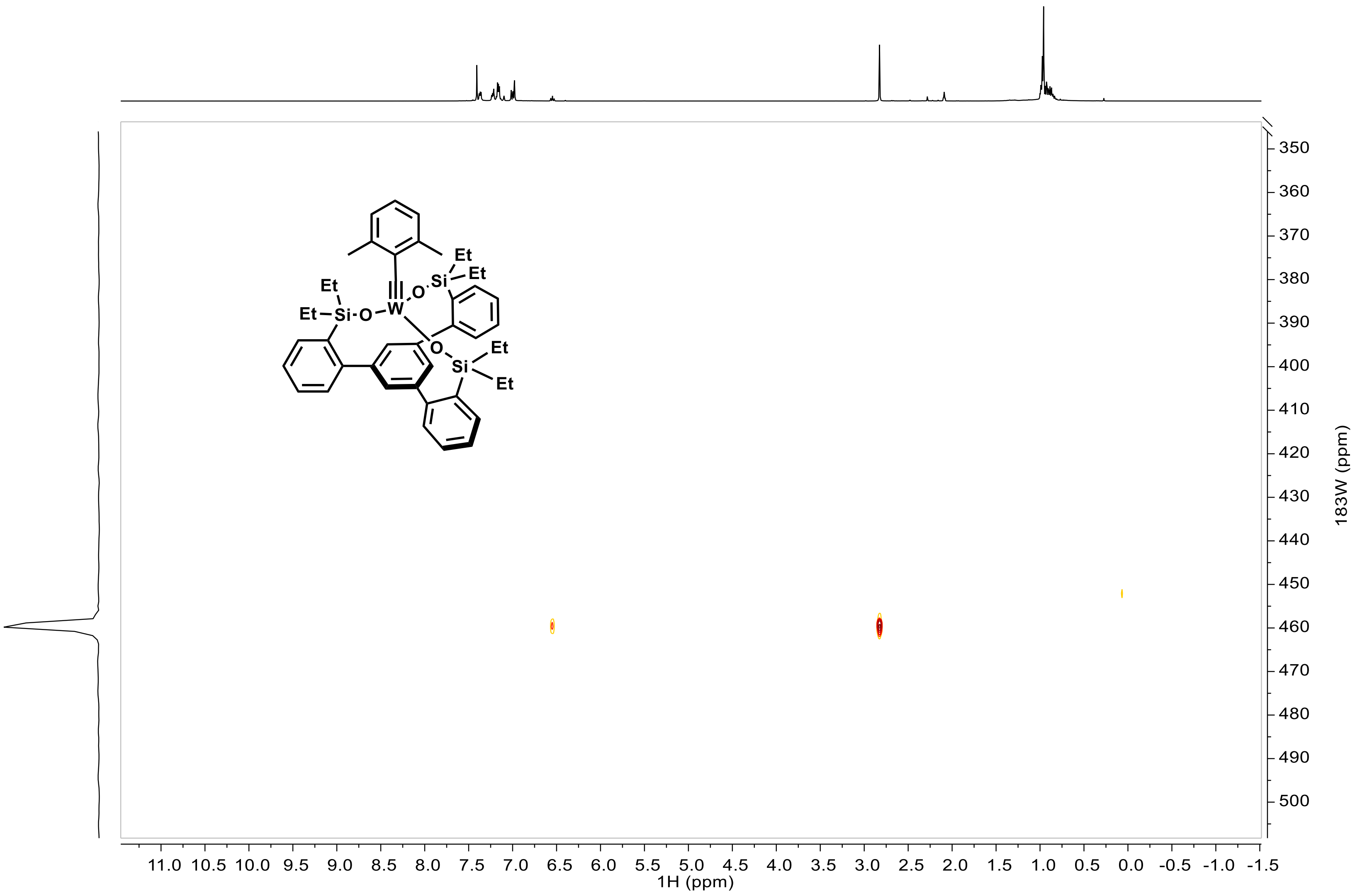

^{183}W NMR Projection created from 2D-HMBC Experiment of Complex 16b, 17 MHz, $C_6D_5CD_3$, 25ºC

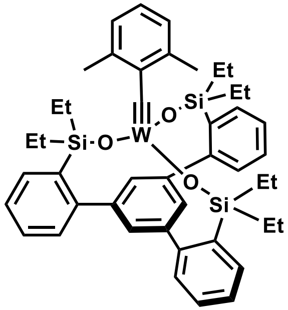

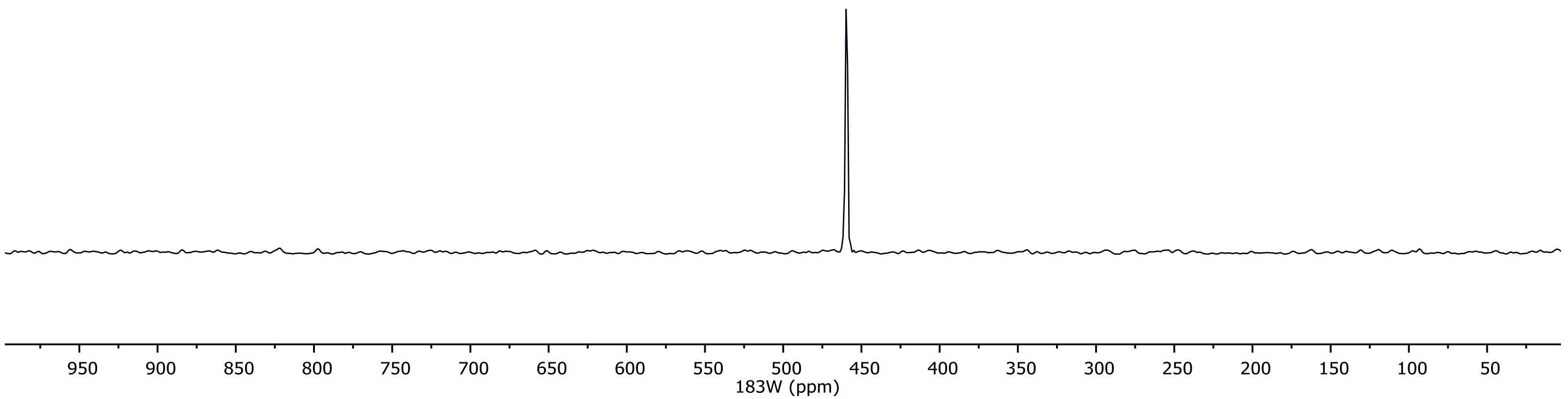

^{1}H NMR of Complex 16c, 600 MHz, $C_6D_5CD_3$, 25ºC

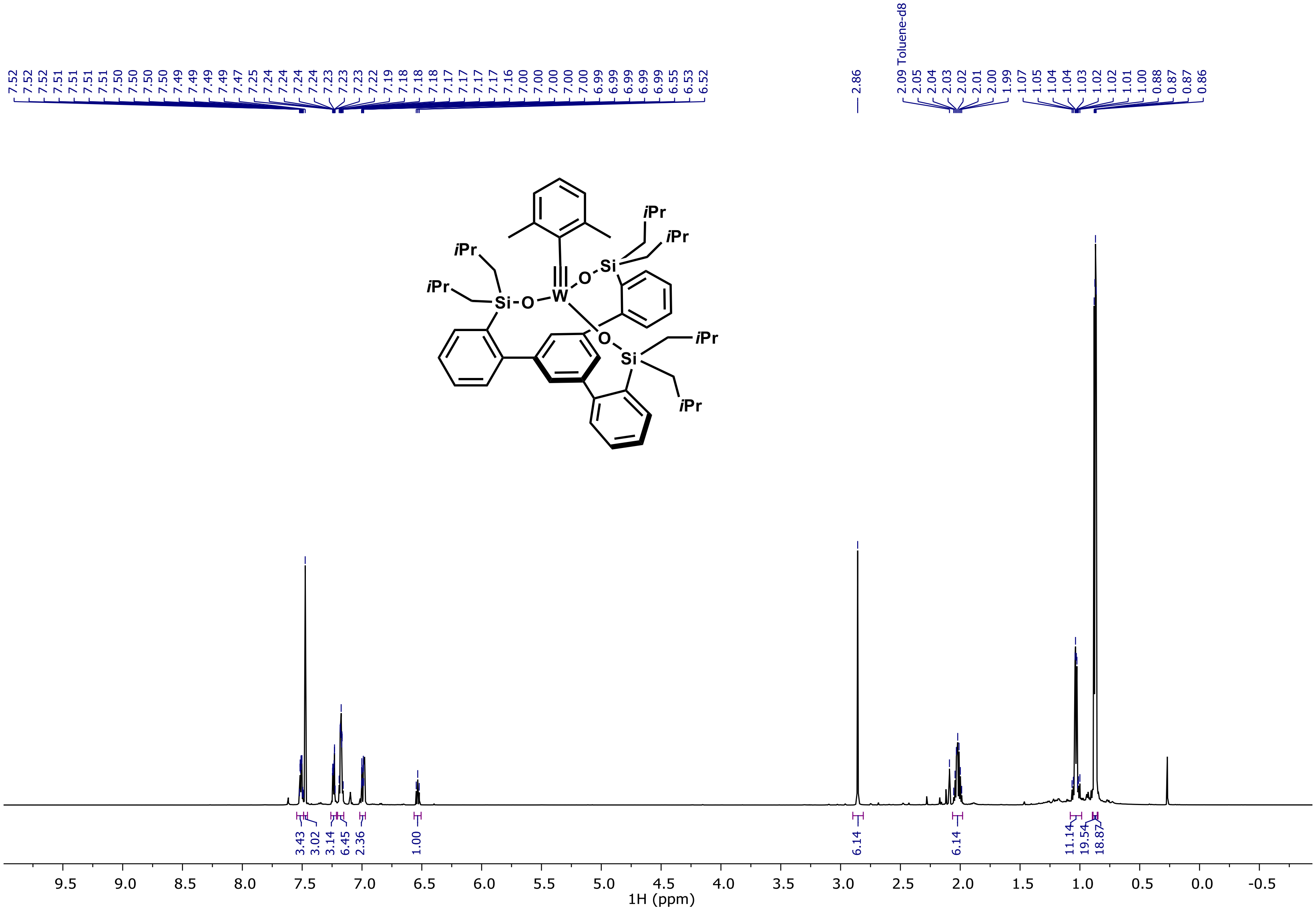

^{13}C NMR of Complex 16c, 151 MHz, $C_6D_5CD_3$, 25ºC

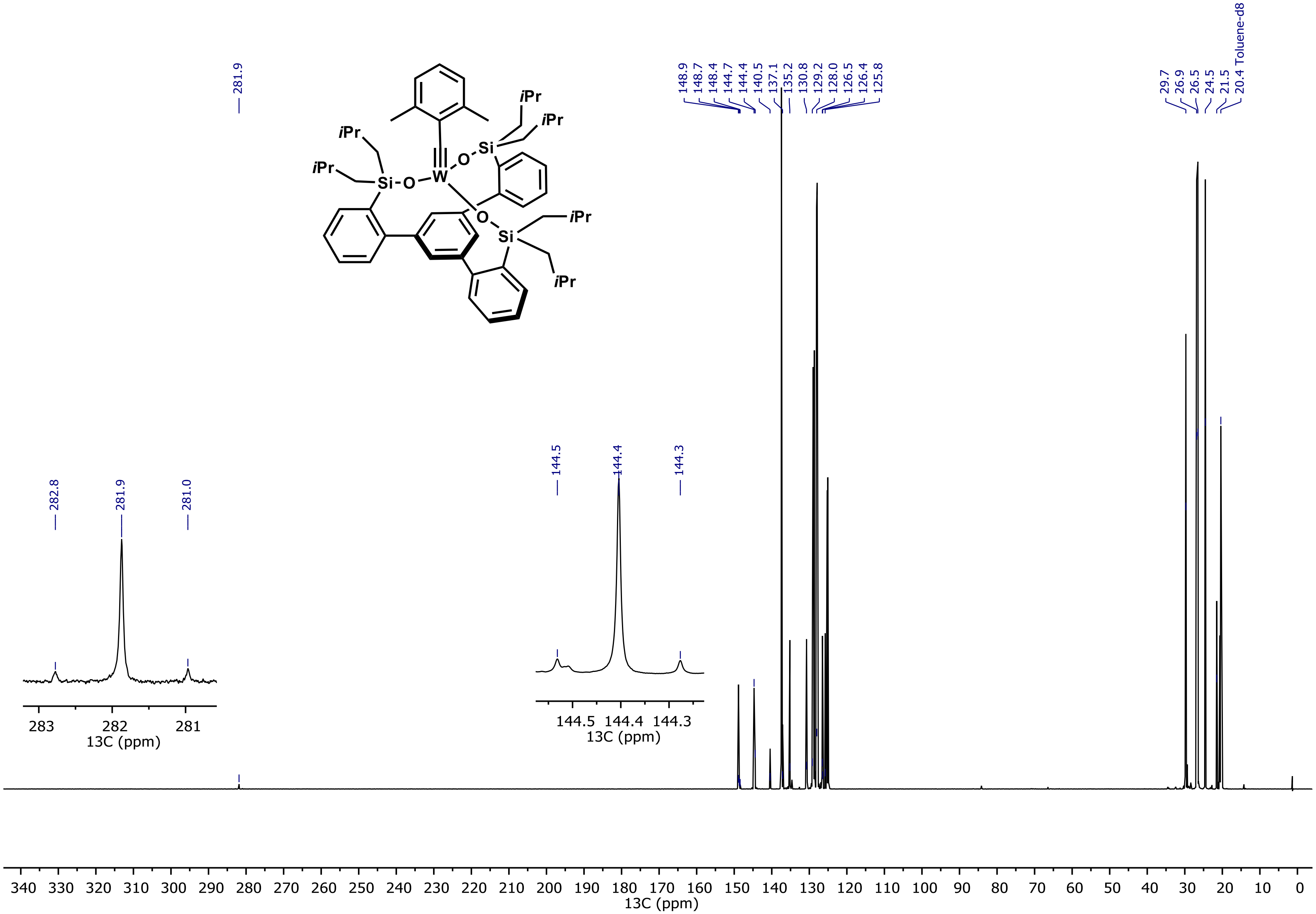

^{29}Si NMR of Complex 16c, 119 MHz, $C_6D_5CD_3$, 25ºC

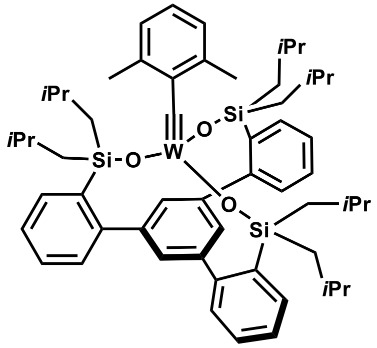

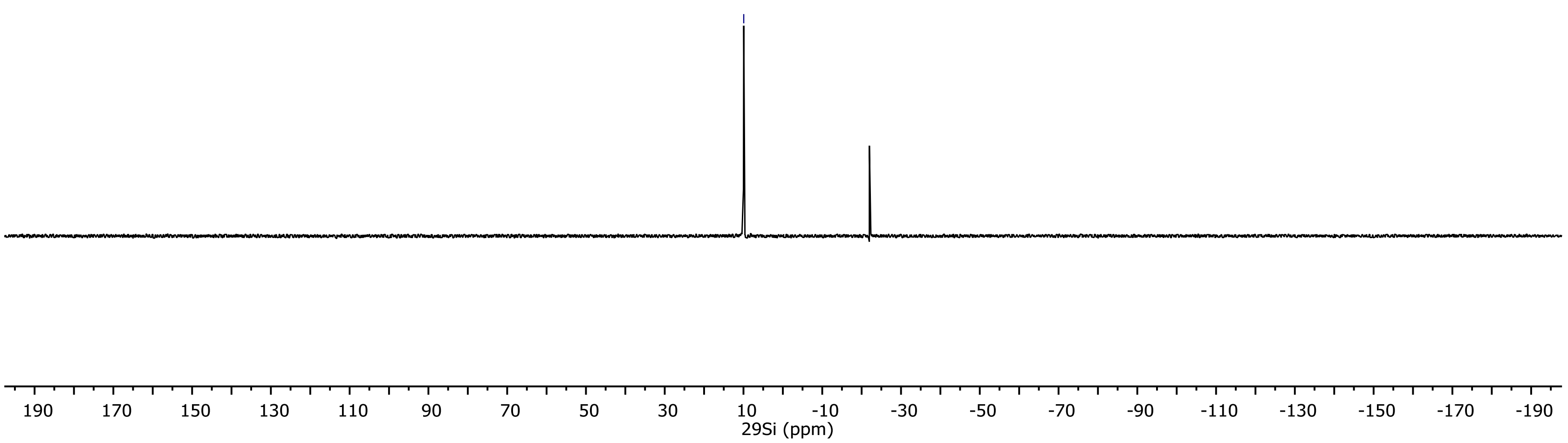

^{1}H-^{183}W HMBC NMR of Complex 16c, 400 MHz, $C_6D_5CD_3$, 25ºC

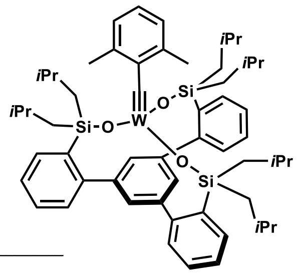

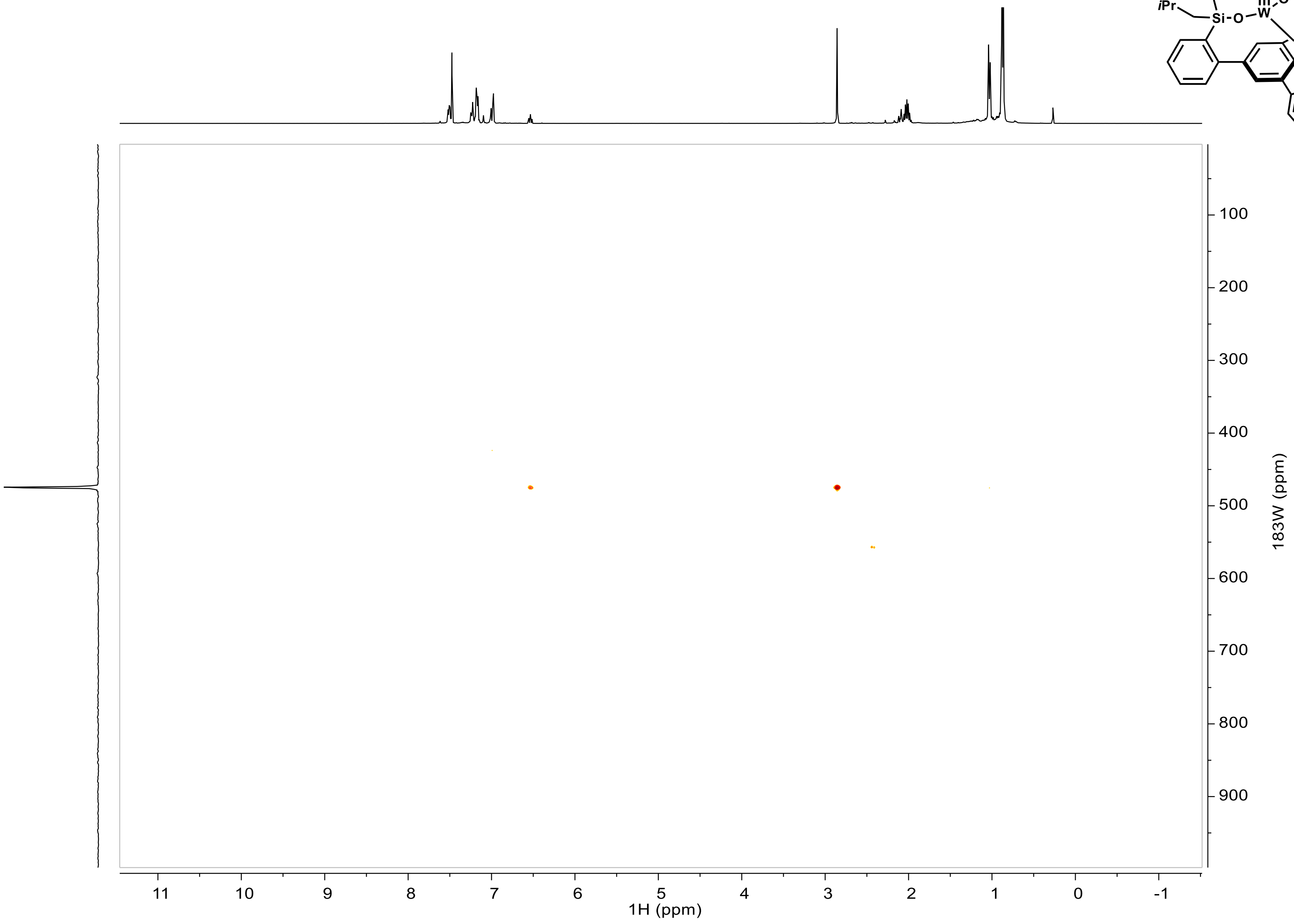

^{183}W NMR Projection created from 2D-HMBC Experiment of Complex 16c, 17 MHz, $C_6D_5CD_3$, 25ºC

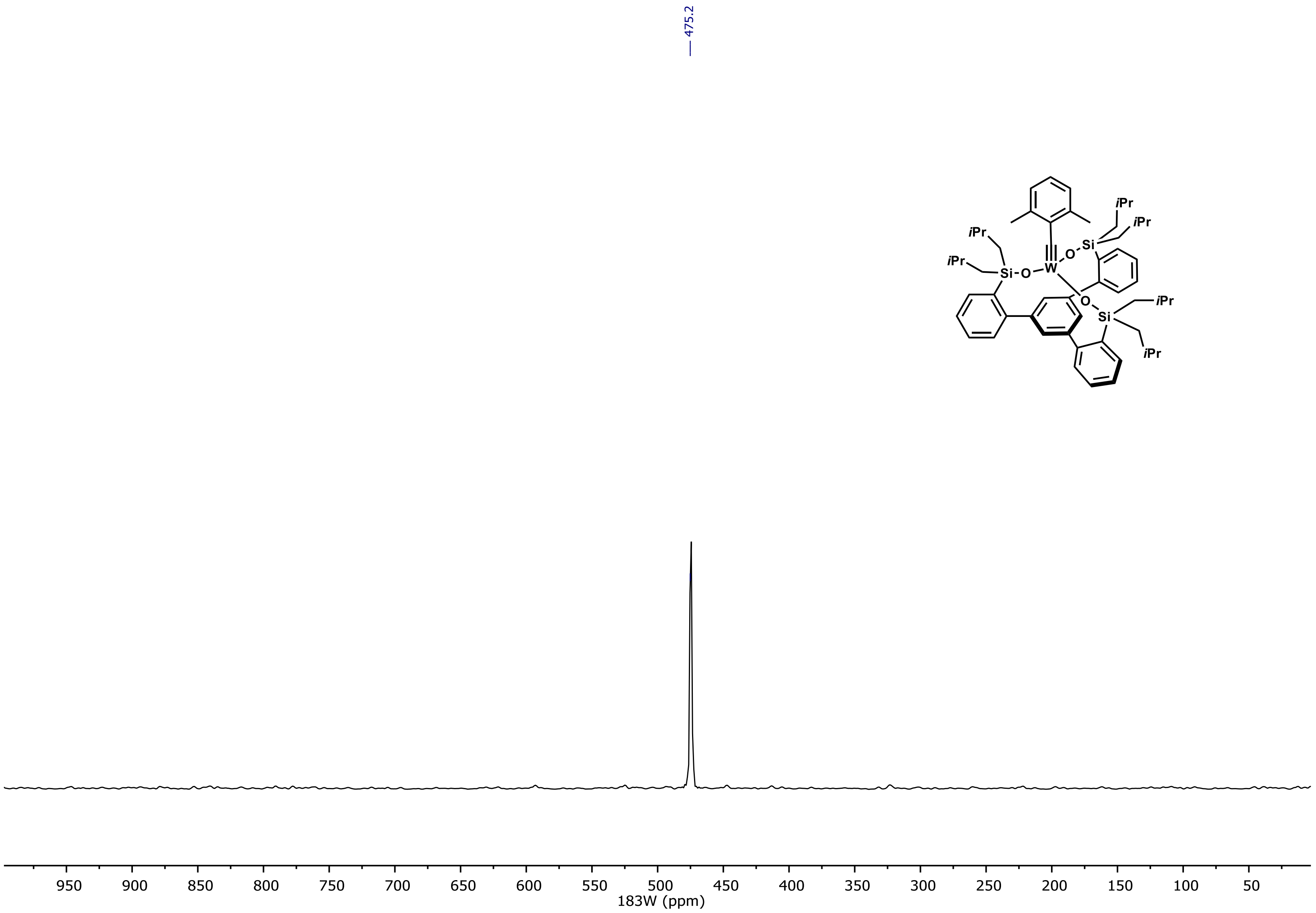

^{1}H NMR of Complex 16d, 600 MHz, $C_6D_5CD_3$, 25ºC

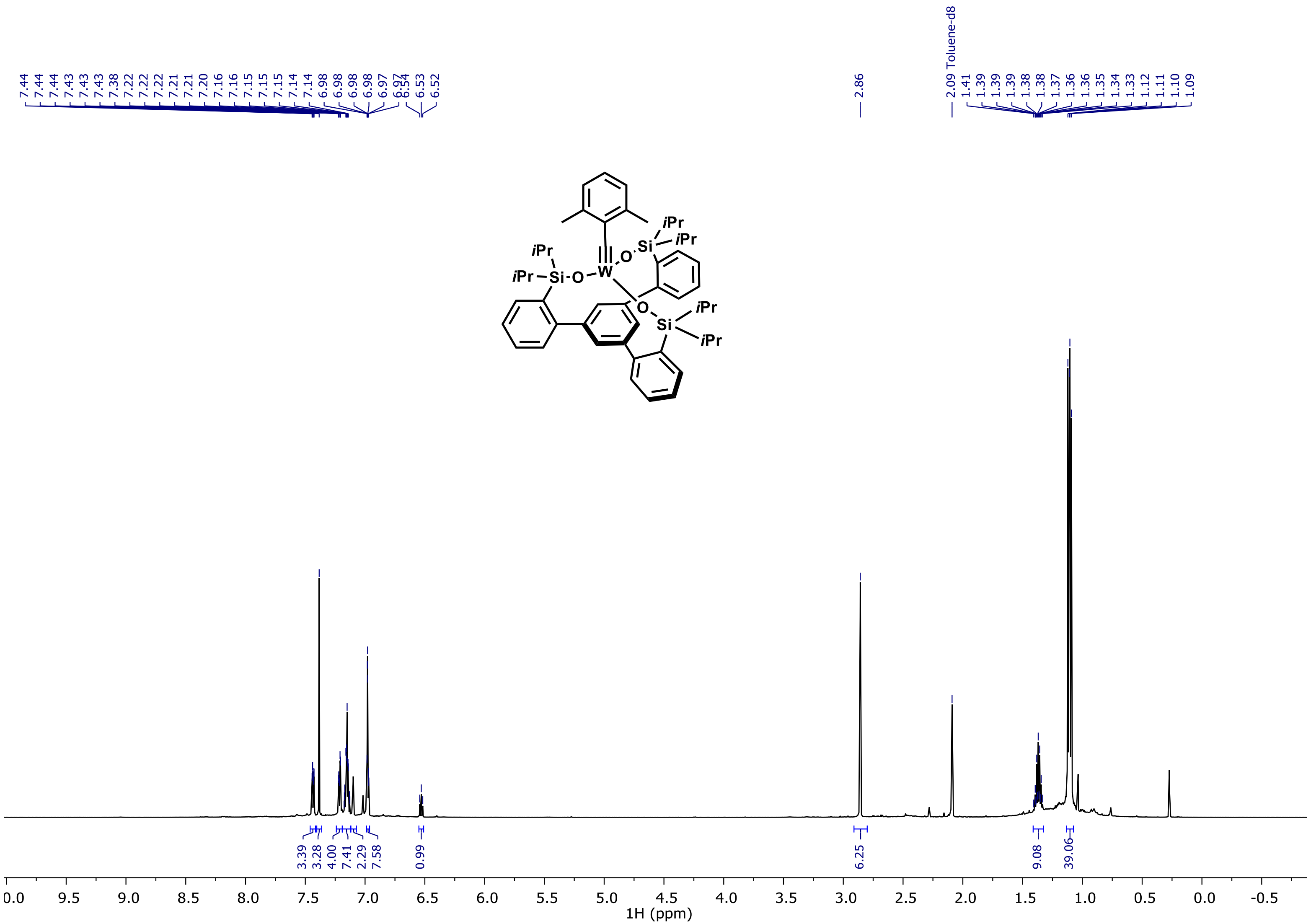

^{13}C NMR of Complex 16d, 151 MHz, $C_6D_5CD_3$, 25ºC

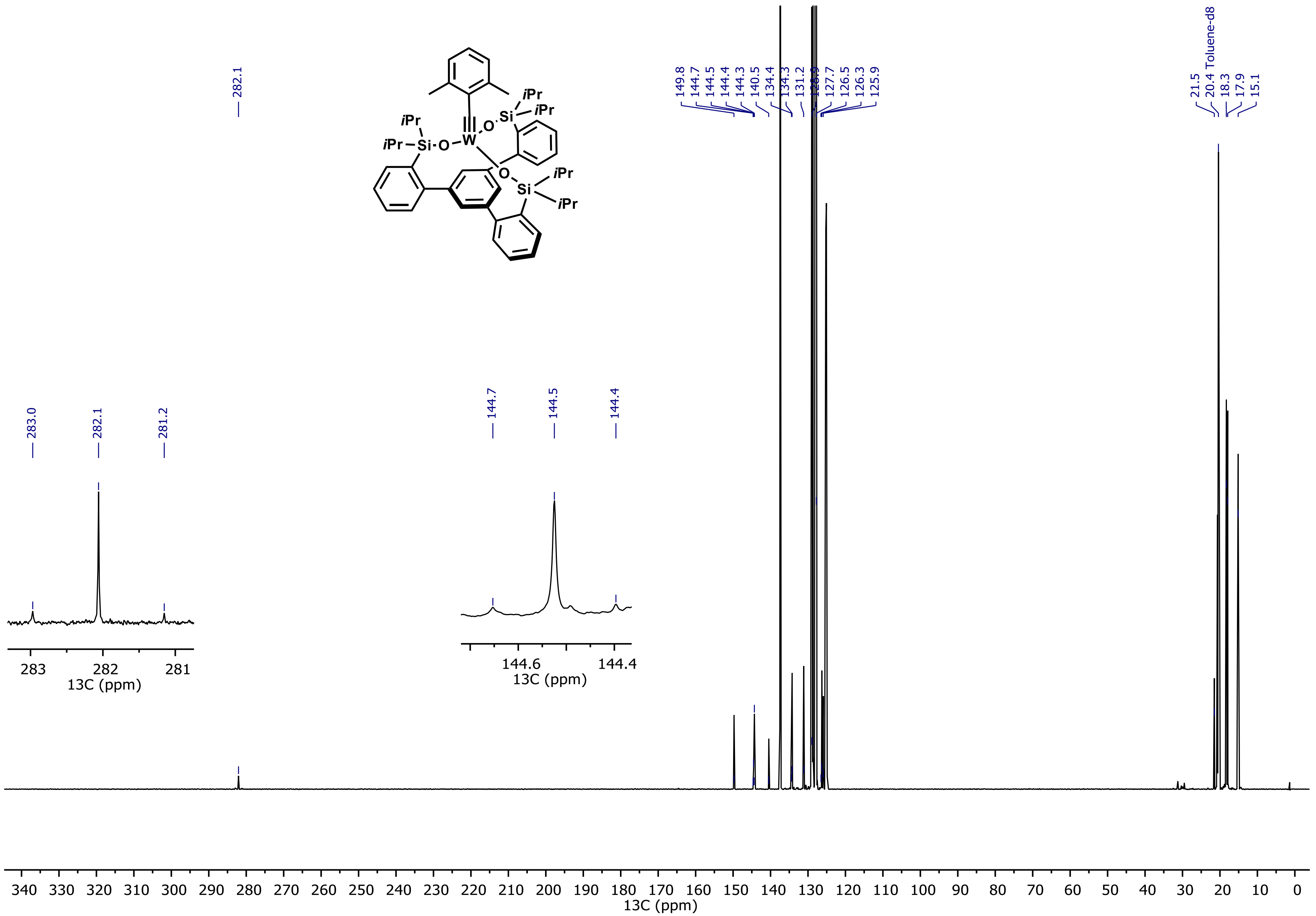

^{29}Si NMR of Complex 16d, 119 MHz, $C_6D_5CD_3$, 25ºC

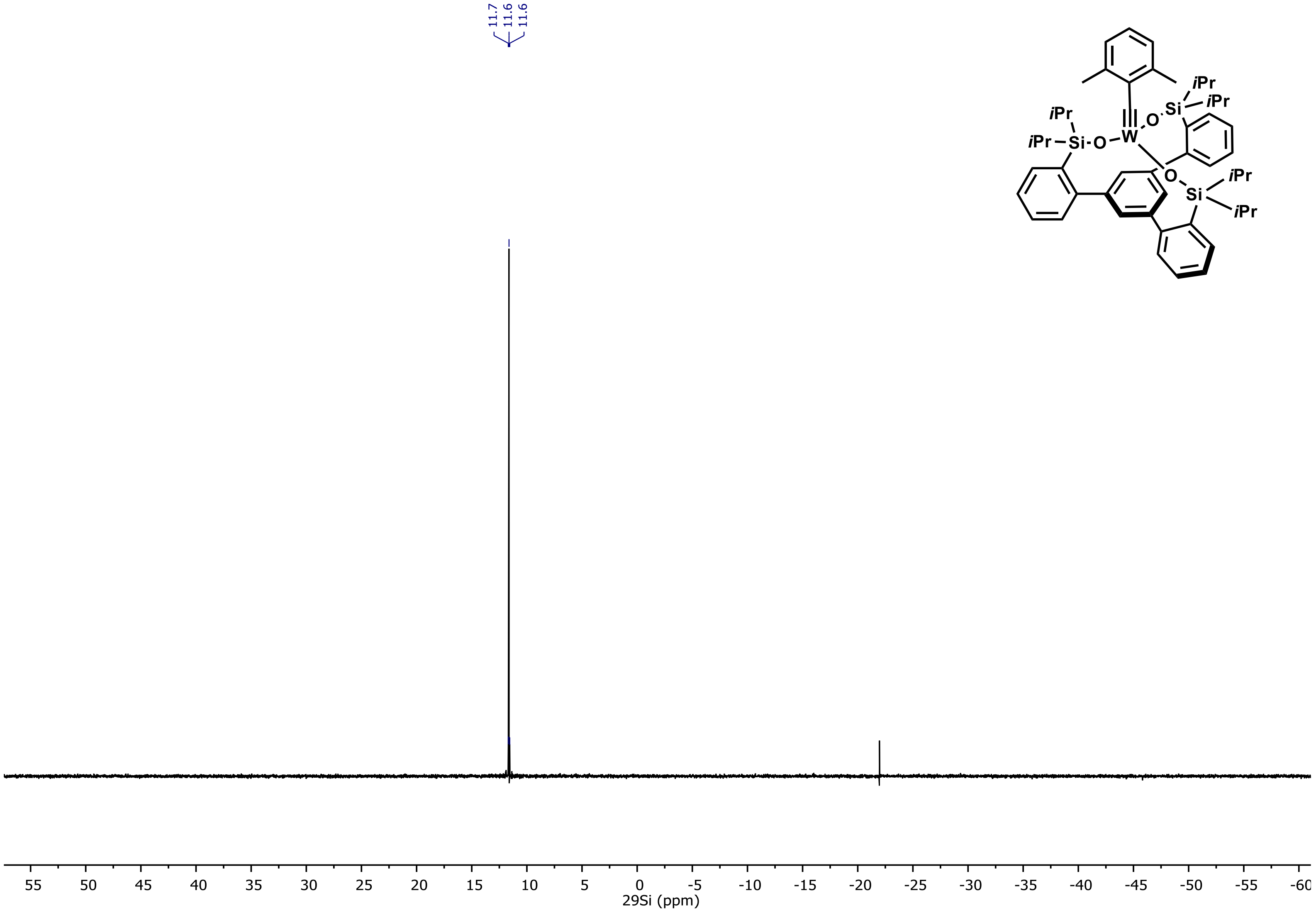

^{1}H-^{183}W HMBC NMR of Complex 16d, 400 MHz, $C_6D_5CD_3$, 25ºC

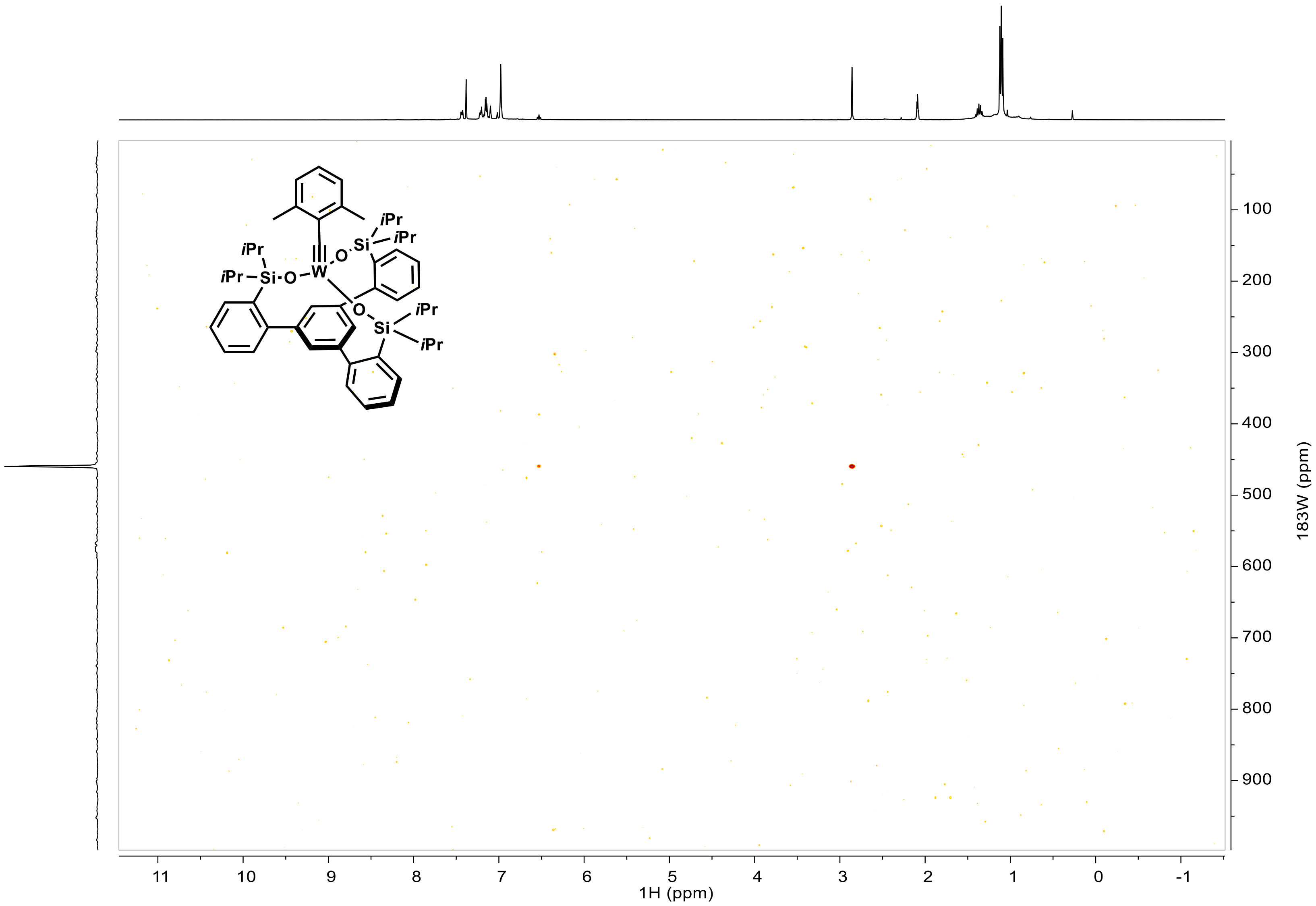

^{183}W NMR Projection created from 2D-HMBC Experiment of Complex 16d, 17 MHz, $C_6D_5CD_3$, 25ºC

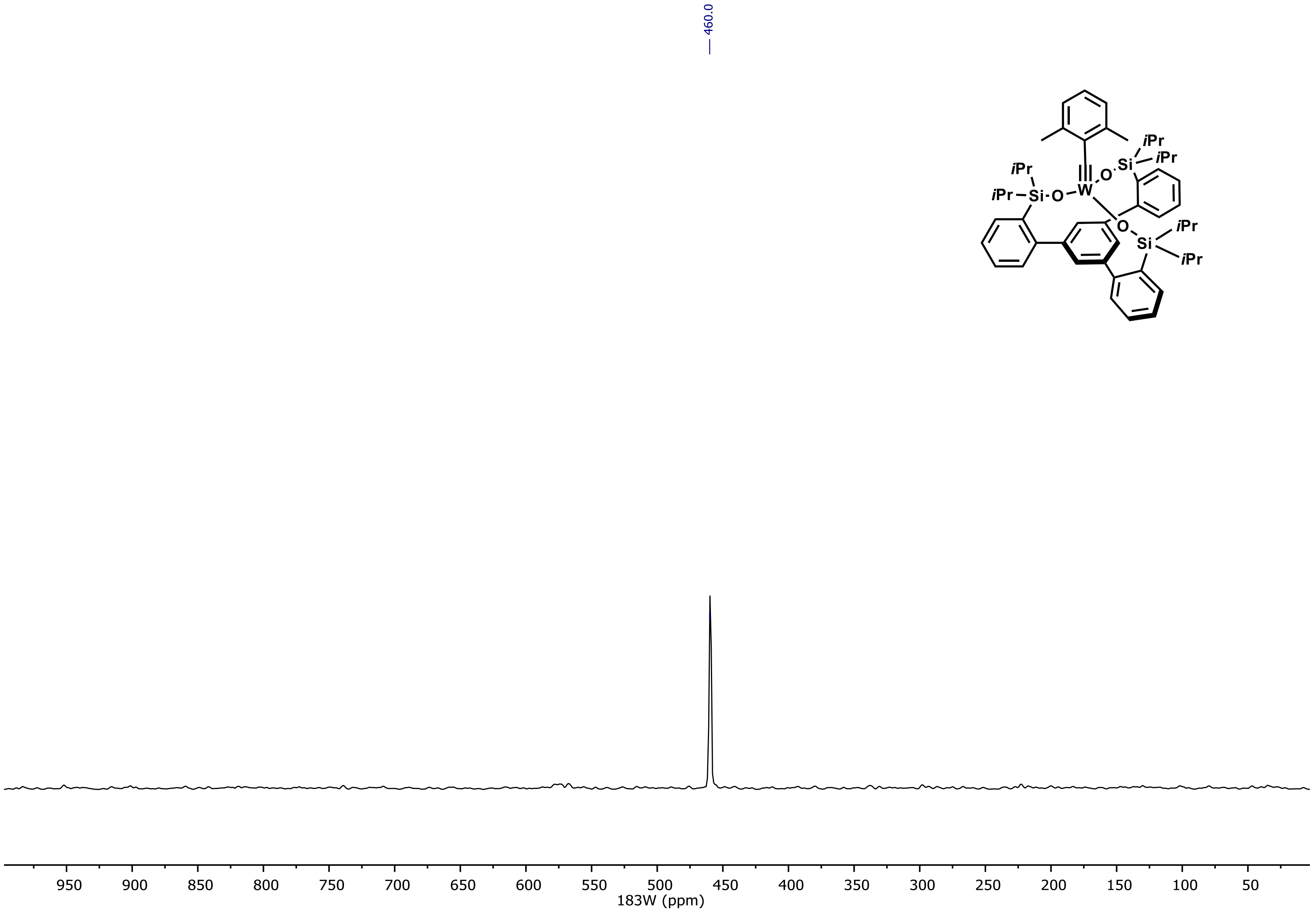

VT NMR studies of mixed metallacyclobutadiene complex 17, 600 MHz, $C_6D_5CD_3$, 25°C to −40ºC

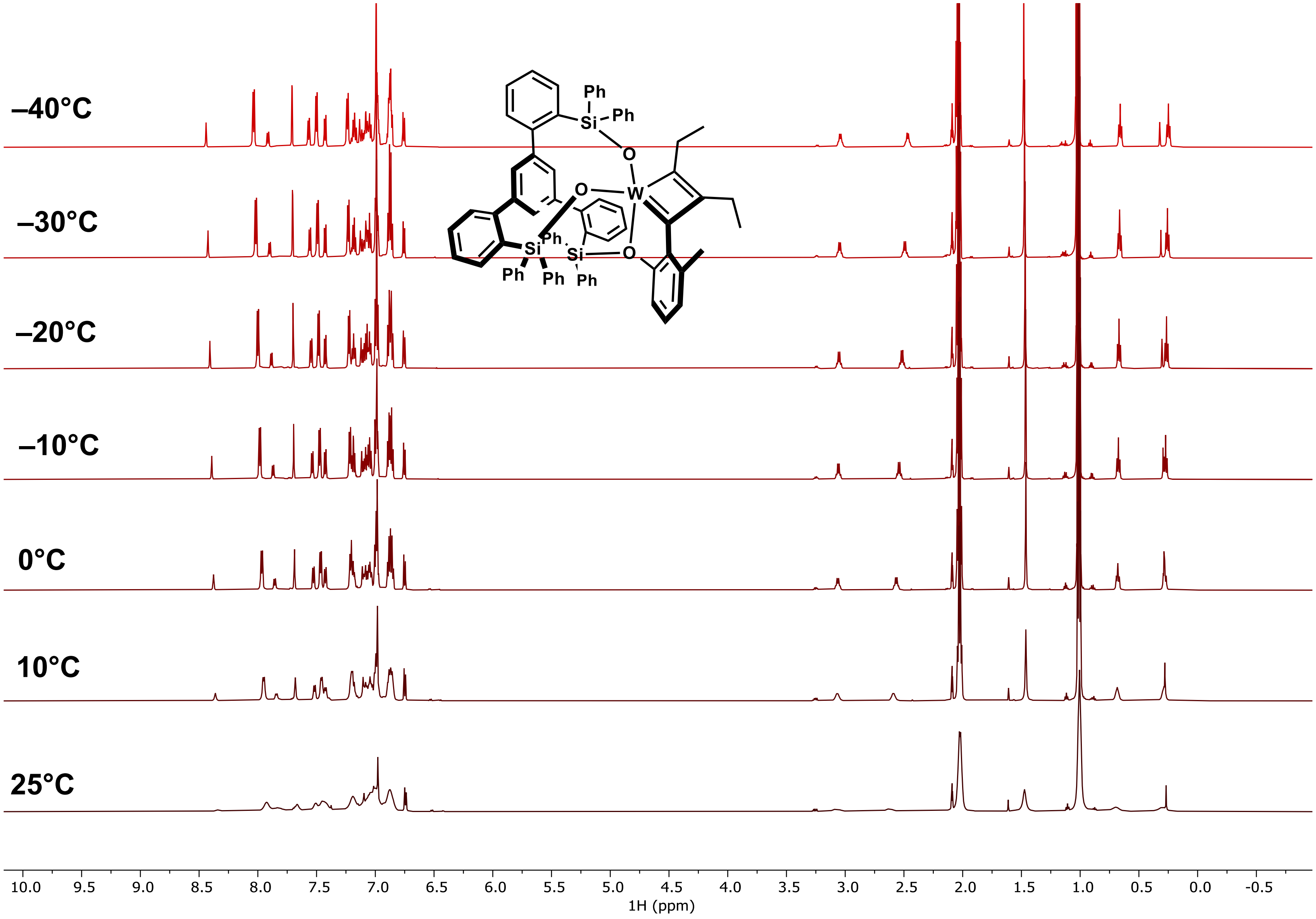

^{1}H NMR of Complex 17, 600 MHz, $C_6D_5CD_3$, −40ºC

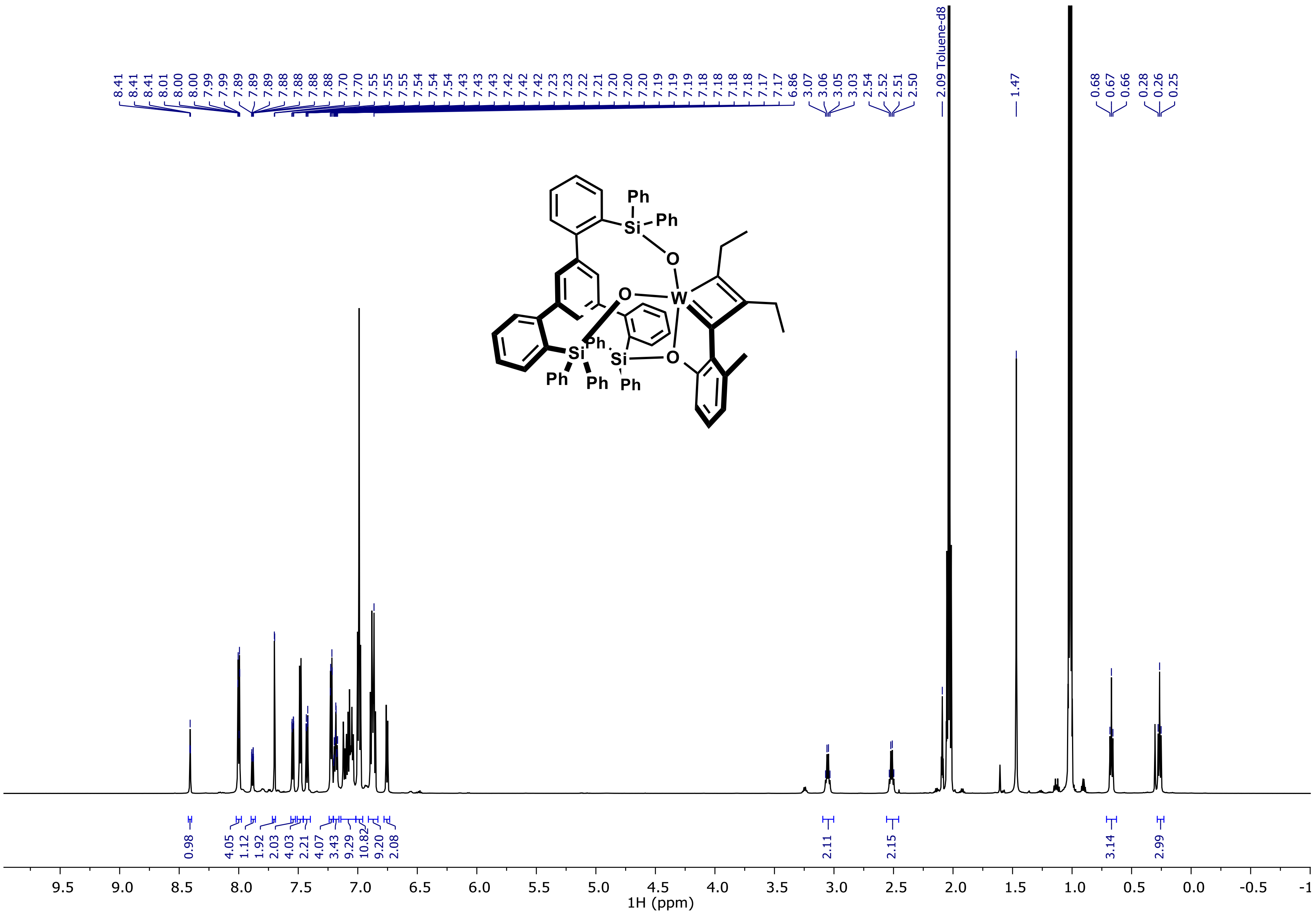

^{13}C NMR of Complex 17, 151 MHz, $C_6D_5CD_3$, −40ºC

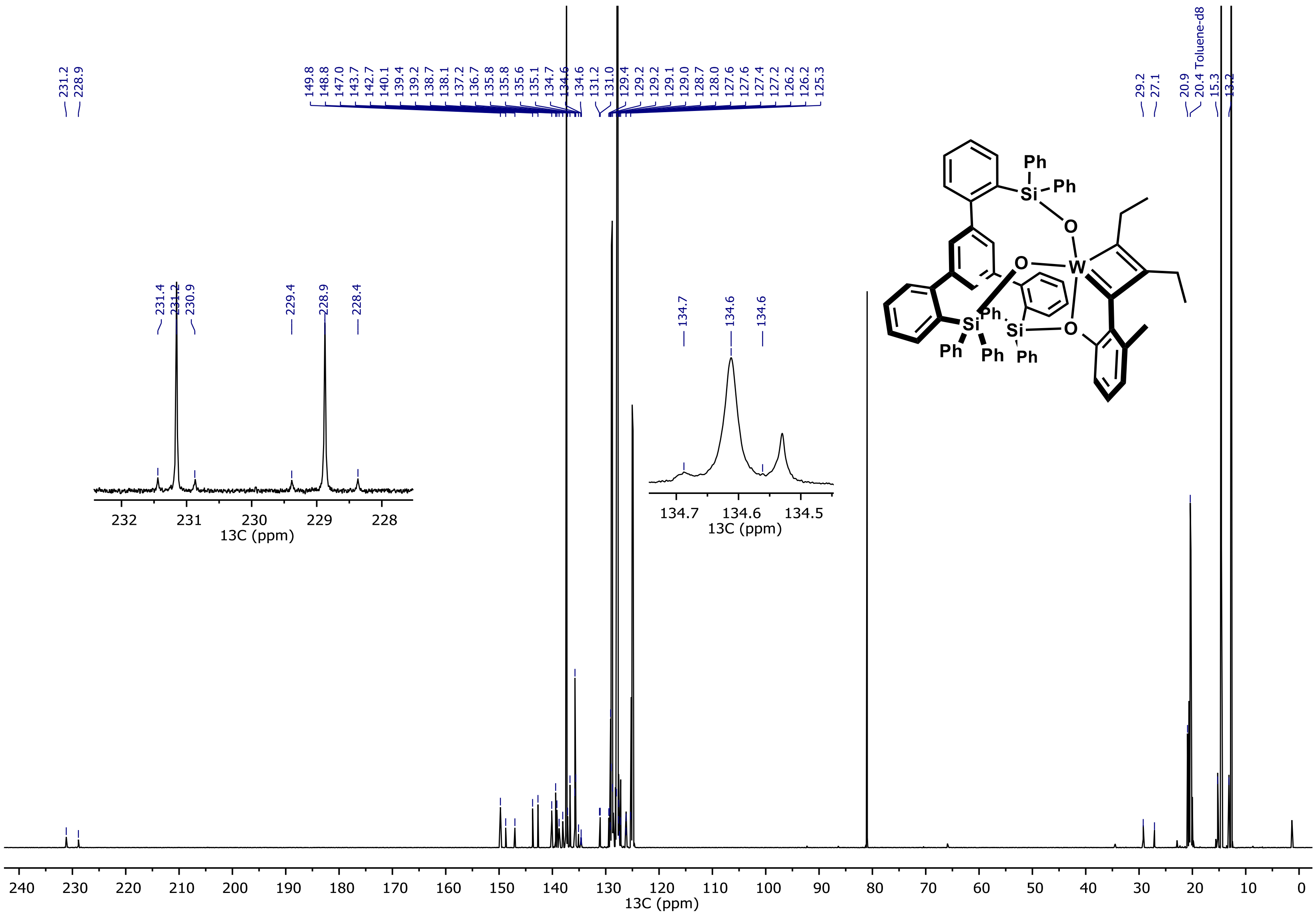

^{29}Si NMR of Complex 17, 119 MHz, $C_6D_5CD_3$, −40ºC

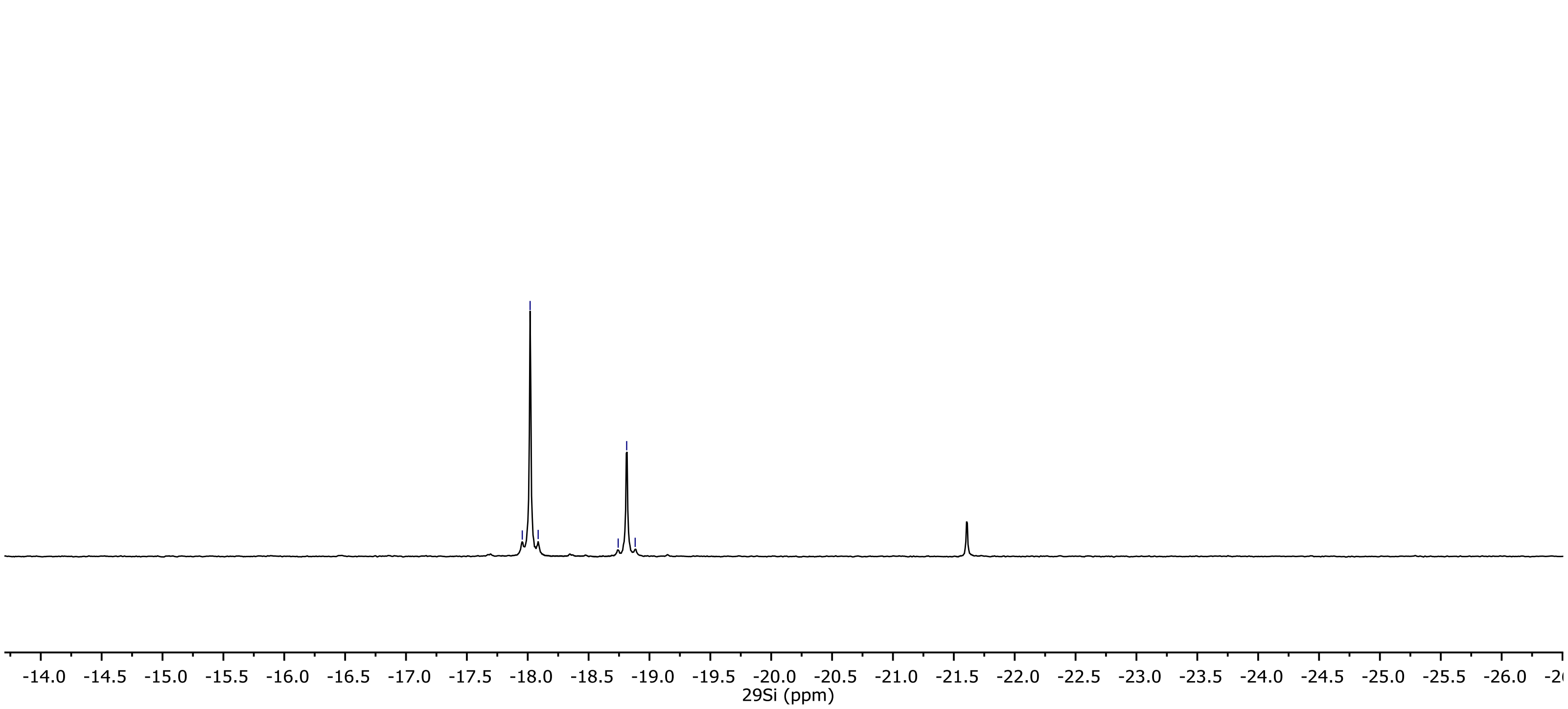

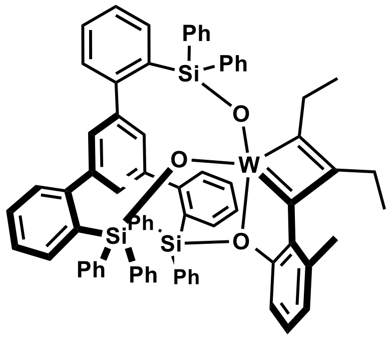

^{1}H EASY ROESY NMR of complex 17, $C_6D_5CD_3$, -40°C

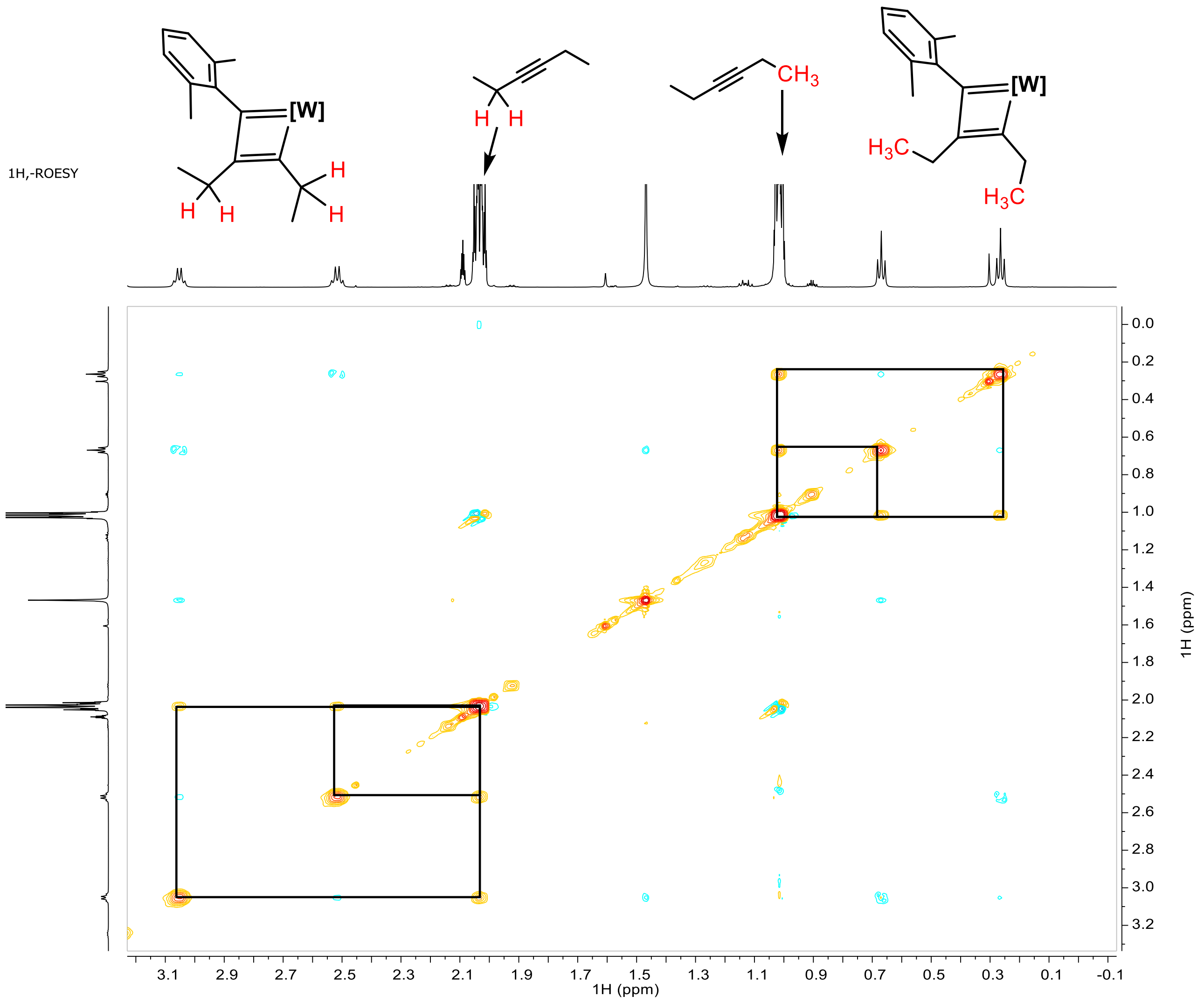

^{1}H EASY ROESY NMR of complex 17, $C_6D_5CD_3$, -40°C

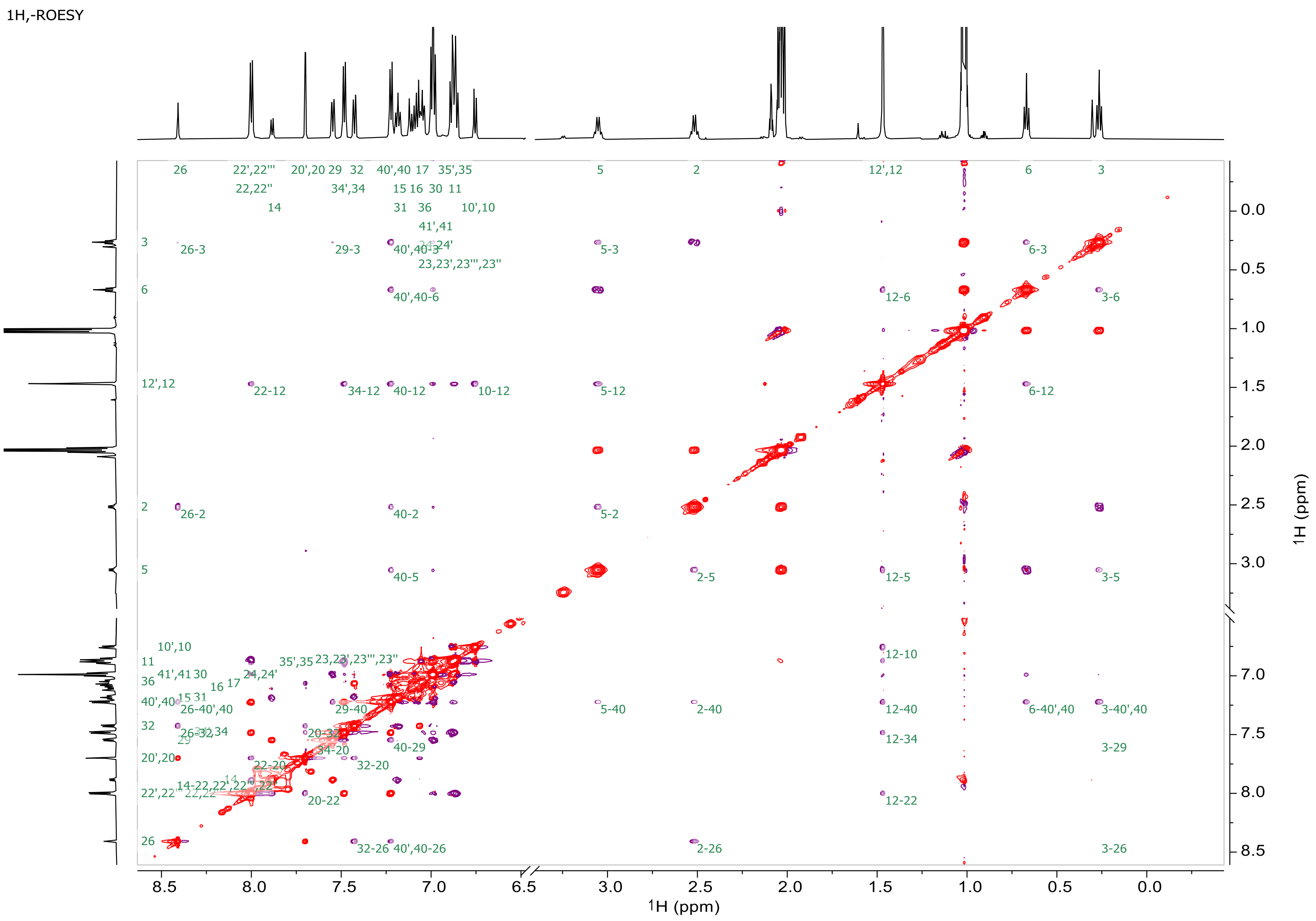

^{1}H COSY NMR of complex 17, $C_6D_5CD_3$, -40°C

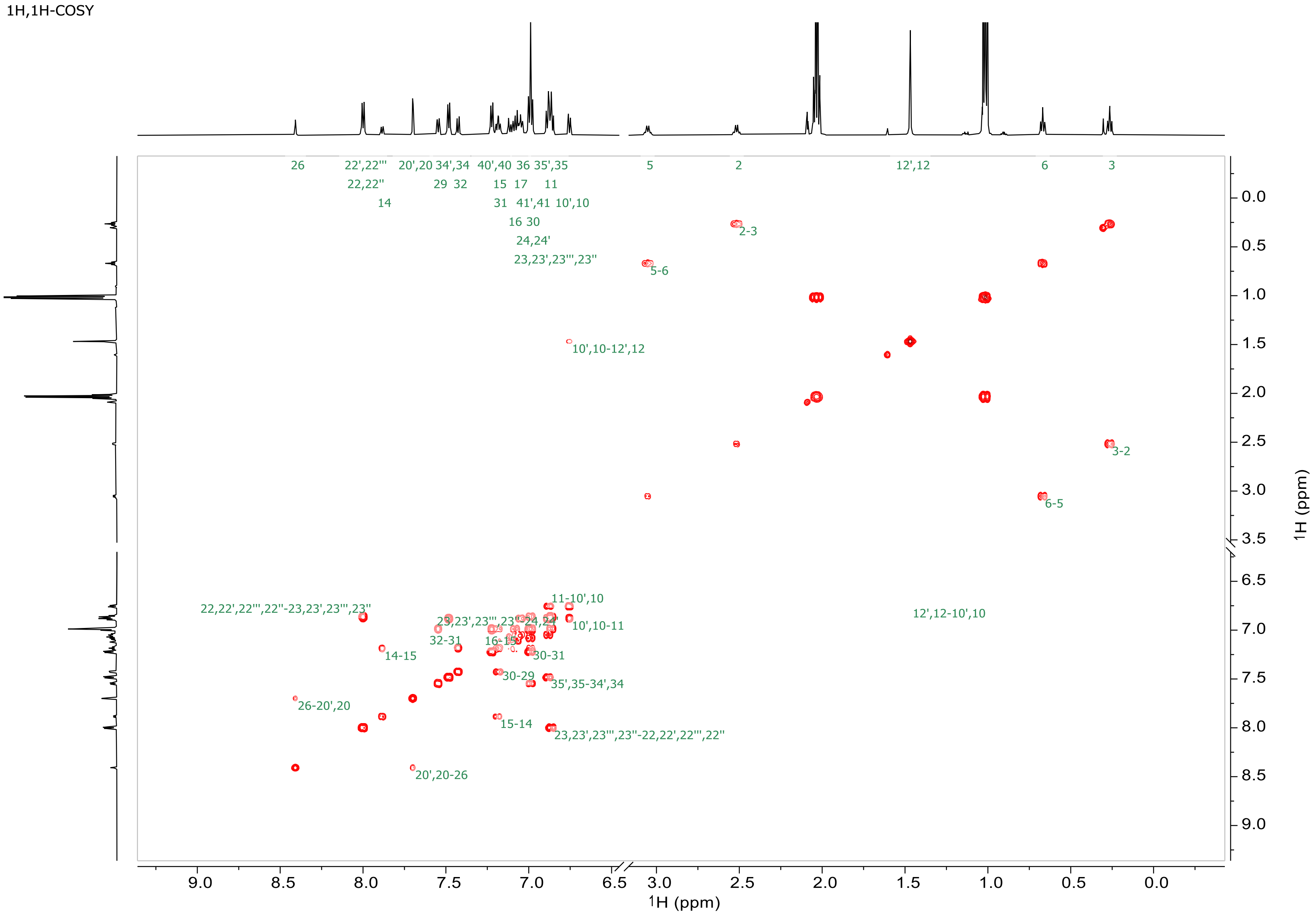

^{1}H ^{29}Si HMBC NMR of complex 17, $C_6D_5CD_3$, -40°C

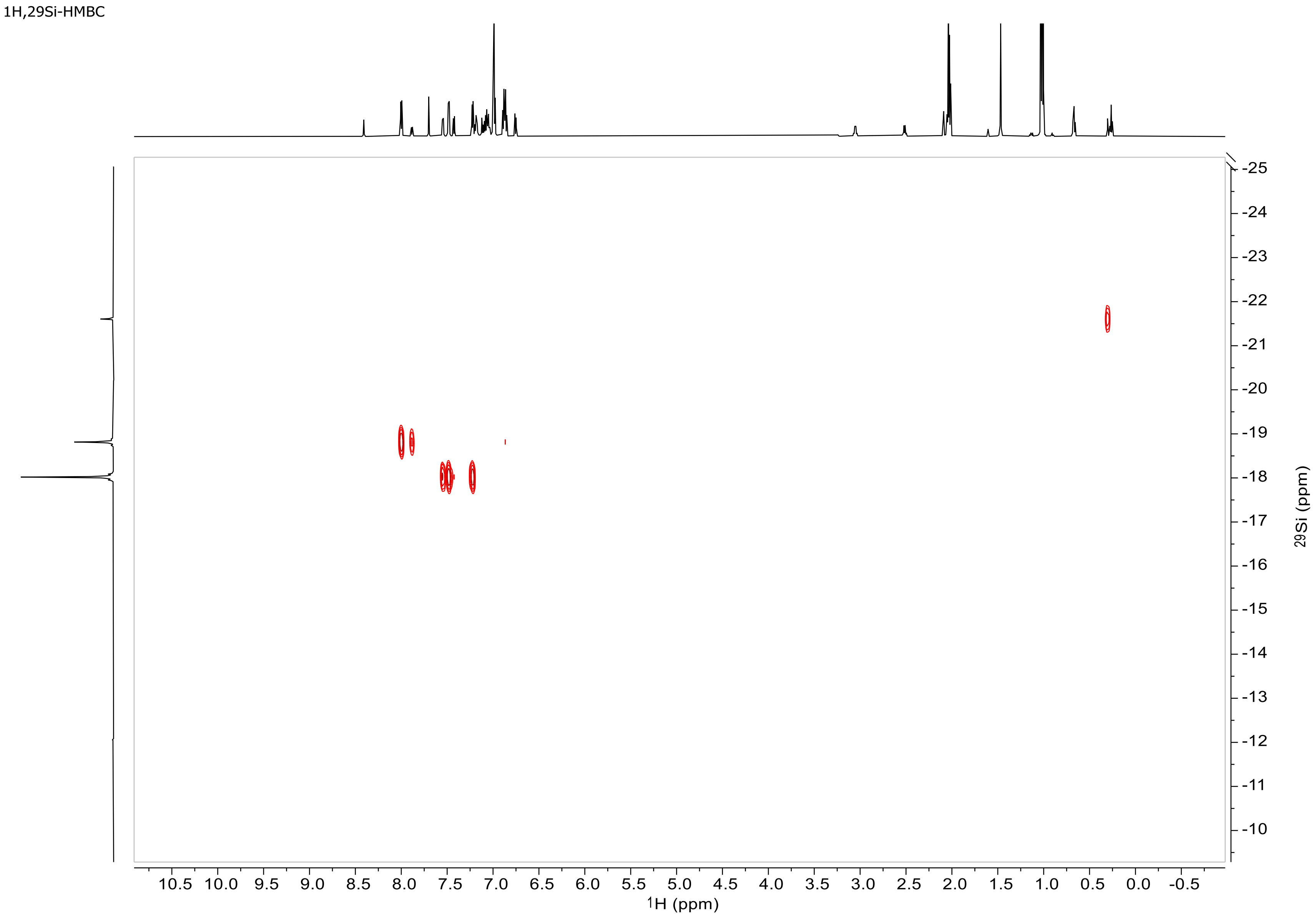

^{1}H HSQC NMR of complex 17, $C_6D_5CD_3$, -40°C

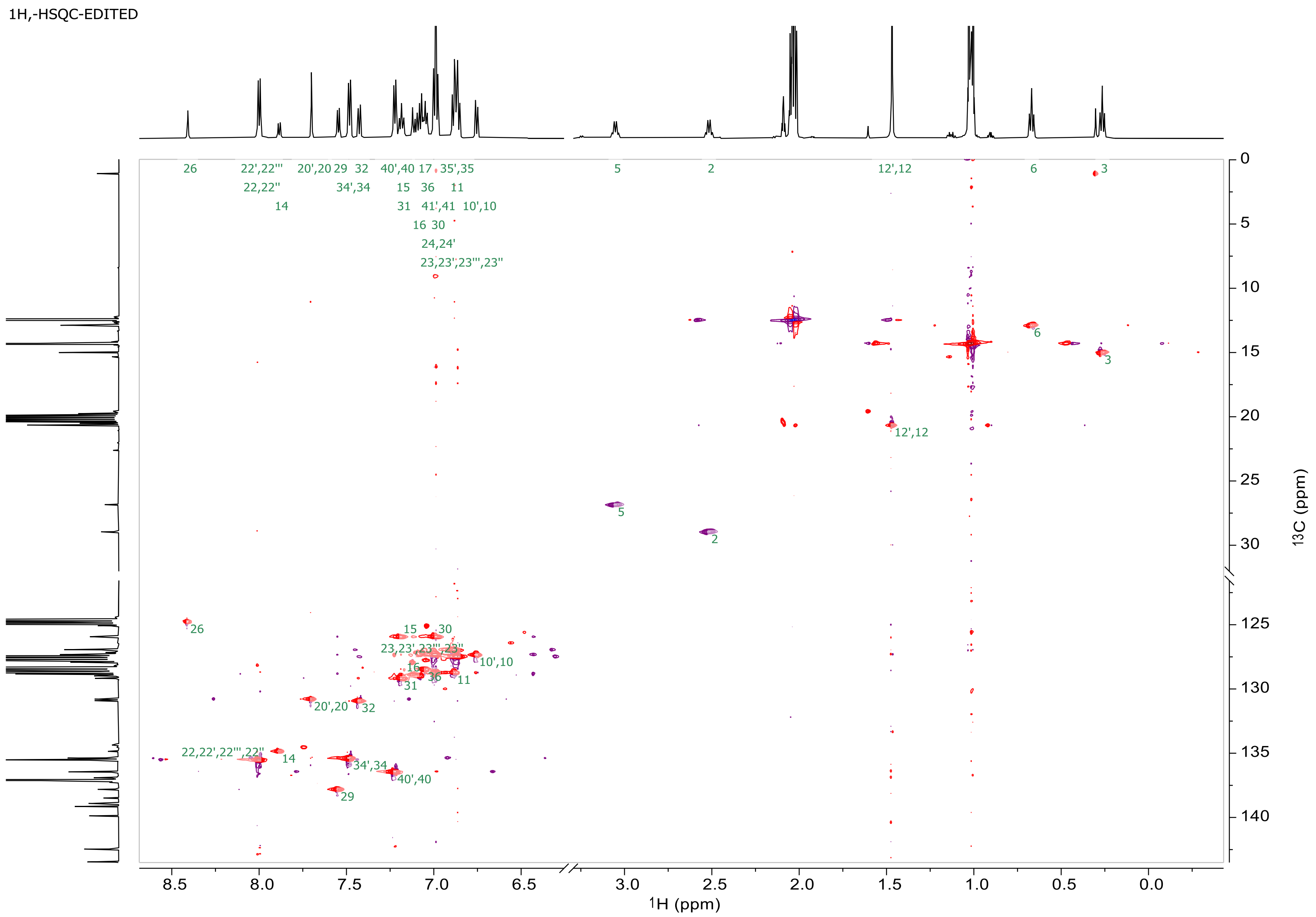

^{1}H HMBC NMR of complex 17, $C_6D_5CD_3$, -40°C

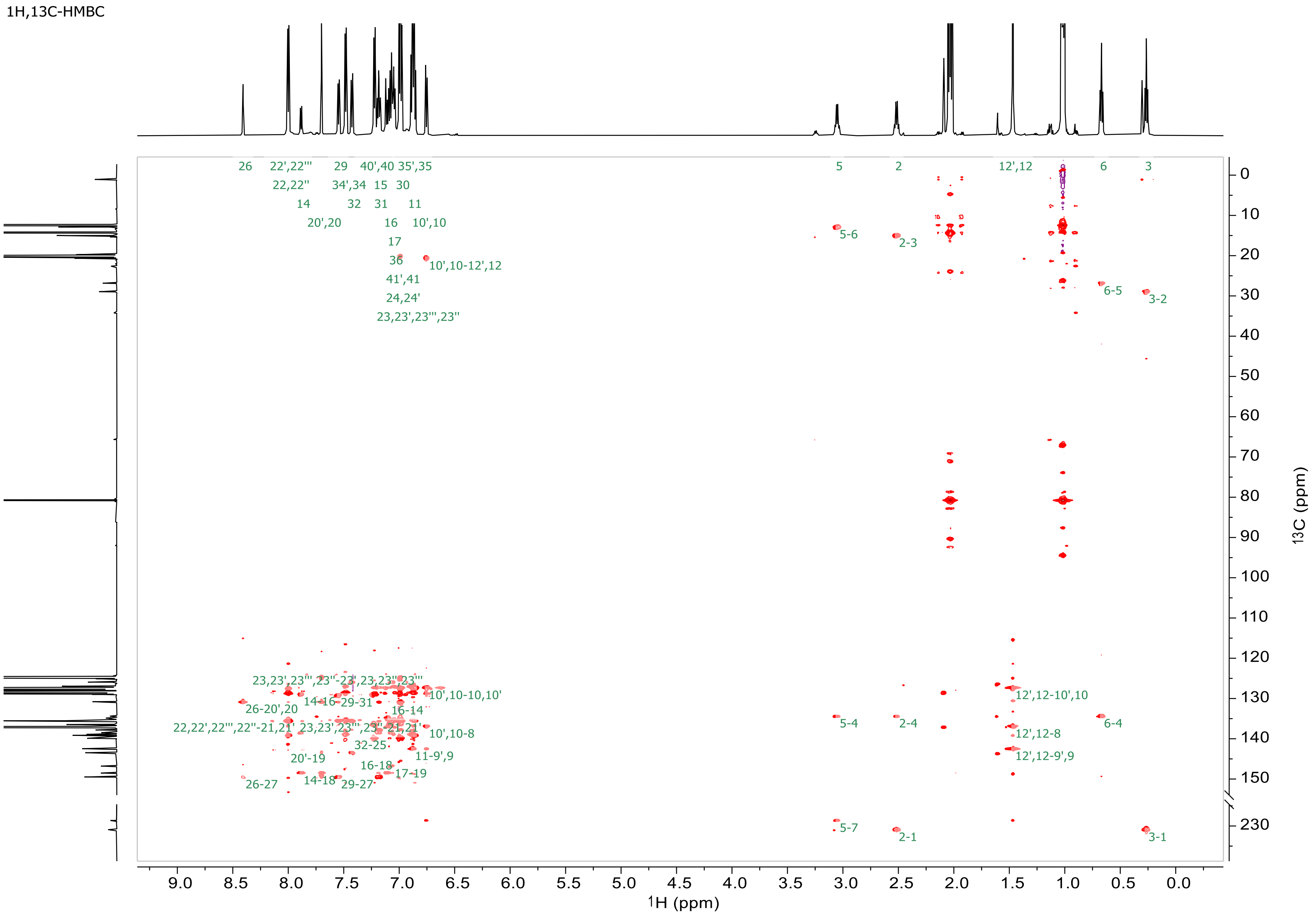

NMR assignments of complex 17, $C_6D_5CD_3$, -20°C

Atom	δ (ppm)	J	COSY	HSQC	HMBC	NOESY
1 C	231.18	86.80(200)			2, 3, 5	
2 C	29.22			2	3	
H2	2.52		3	2	1, 3, 4	5, 26, 40
3 C	15.26			3	2	
H3	0.26		2	3	1, 2	5, 6, 26, 29, 40, 40'
4 C	134.63	23.00(200)			2, 5, 6	
5 C	27.11			5	6	
H2	3.05		6	5	1, 4, 6, 7	2, 3, 12, 40
6 C	13.15			6	5	
H3	0.67		5	6	4, 5	3, 12, 40, 40'
7 C	228.90	156.00(200)			5	
8 C	137.17				10, 10', 12, 12'	
9 C	142.72				11, 12	
9' C	142.72				11, 12'	
10 C	127.59			10	10', 12	
H	6.75		11, 12	10	8, 10', 12	12
10' C	127.59			10'	10, 12'	
H	6.75		11, 12'	10'	8, 10, 12'	
11 C	128.98			11		
H	6.88		10, 10'	11	9, 9'	
12 C	20.91			12	10	
H3	1.47		10	12	8, 9, 10	5, 6, 10, 22, 34, 40
12' C	20.91			12'	10'	
H3	1.47		10'	12'	8, 9', 10'	
13 C	138.76				14, 15, 17	
14 C	135.12			14	16	
H	7.88		15	14	13, 16, 18	22, 22', 22'', 22'''

Atom	δ (ppm)	J	COSY	HSQC	HMBC	NOESY
15 C	126.20			15	17	
H	7.19		14, 16	15	13, 17	
16 C	129.17			16	14, 20'	
H	7.10		15, 17	16	14, 18	
17 C	127.43			17	15	
H	7.06		16, 17	17	13, 15, 19	
18 C	148.77				14, 16, 20	
19 C	147.05				17, 20'	
20 C	131.05			20	20', 26	
H	7.70		26	20	18, 20', 26, 27	22, 32, 34
20' C	131.05			20'	20, 26	
H	7.70		26	20'	16, 19, 20	
21 C	139.41				22, 22', 23, 23'	
21' C	139.41				22'', 22''', 23'', 23'''	
22 C	135.78			22	22', 24	
H	8.00		23	22	21, 22', 24	12, 14, 20
22' C	135.78			22'	22, 24	
H	8.00		23'	22'	21, 22, 24	14
22'' C	135.78			22''	22''', 24'	
H	8.00		23''	22''	21', 22''', 24'	14
22''' C	135.78			22'''	22'', 24'	
H	8.00		23'''	22'''	21', 22'', 24'	14
23 C	127.71			23	23'	
H	6.86		22, 24	23	21, 23'	
23' C	127.71			23'	23	
H	6.86		22', 24	23'	21, 23	

Atom	δ (ppm)	J	COSY	HSQC	HMBC	NOESY
23'' C	127.71			23''	23'''	
H	6.86		22'', 24'	23''	21', 23'''	
23''' C	127.71			23'''	23''	
H	6.86		22''', 24'	23'''	21', 23''	
24 C	128.99			24	22, 22'	
H	6.99		23, 23'	24	22, 22'	
24' C	128.99			24'	22'', 22'''	
H	6.99		23'', 23'''	24'	22'', 22'''	
25 C	143.73				32	
25' C	143.73					
26 C	125.03			26	20	
H	8.41		20, 20'	26	20, 20', 27	2, 3, 32, 40, 40'
27 C	149.77				20, 26, 29, 31	
28 C	135.80				29, 30, 32	
29 C	138.07			29	31	
H	7.55		30	29	27, 28, 31	3, 40
30 C	126.19			30	32	
H	6.99		29, 31	30	28, 32	
31 C	129.43			31	29	
H	7.18		30, 32	31	27, 29	
32 C	131.18			32	30	
H	7.43		31	32	25, 28, 30	20, 26
33 C	139.17				34, 34', 35, 35'	
34 C	135.64			34	34', 36	
H	7.48		35	34	33, 34', 36	12, 20
34' C	135.64			34'	34, 36	
H	7.48		35'	34'	33, 34, 36	

Atom	δ (ppm)	J	COSY	HSQC	HMBC	NOESY
35 C	127.20			35	35'	
H	6.88		34, 36	35	33, 35'	
35' C	127.20			35'	35	
H	6.88		34', 36	35'	33, 35	
36 C	128.74			36	34, 34'	
H	7.05		35, 35'	36	34, 34'	
39 C	140.14				40, 40', 41	
40 C	136.72			40	40', 42	
H	7.22		41	40	39, 40', 42	2, 3, 5, 6, 12, 26, 29
40' C	136.72			40'	40, 42	
H	7.22		41'	40'	39, 40, 42	3, 6, 26
41 C	127.61			41		
H	6.99		40, 42	41	39, 41'	
41' C	127.61			41'	41	
H	6.99		40', 42	41'		
42 C	129.24			42	40, 40'	
H			41, 41'	42	40, 40'	
100 Si	-18.81					
101 Si	-18.02					
101' Si	-18.02					
200 W		156.00(7), 86.80(1), 23.00(4)				

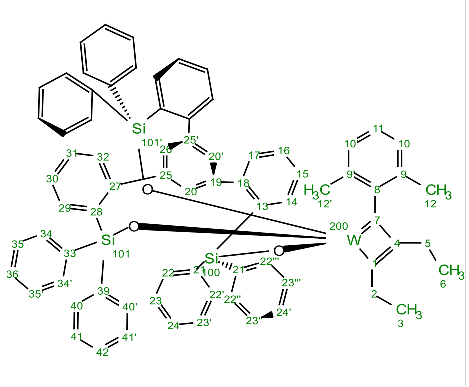

Remarks:
The chem. shift of C16 was extracted from 2D HSQC due to the overlap with solvent signals. 1H shifts of complex multiplets were extracted trom the 2D data.

^{1}H NMR of complex 18, 600 MHz, $C_6D_5CD_3$, −20ºC

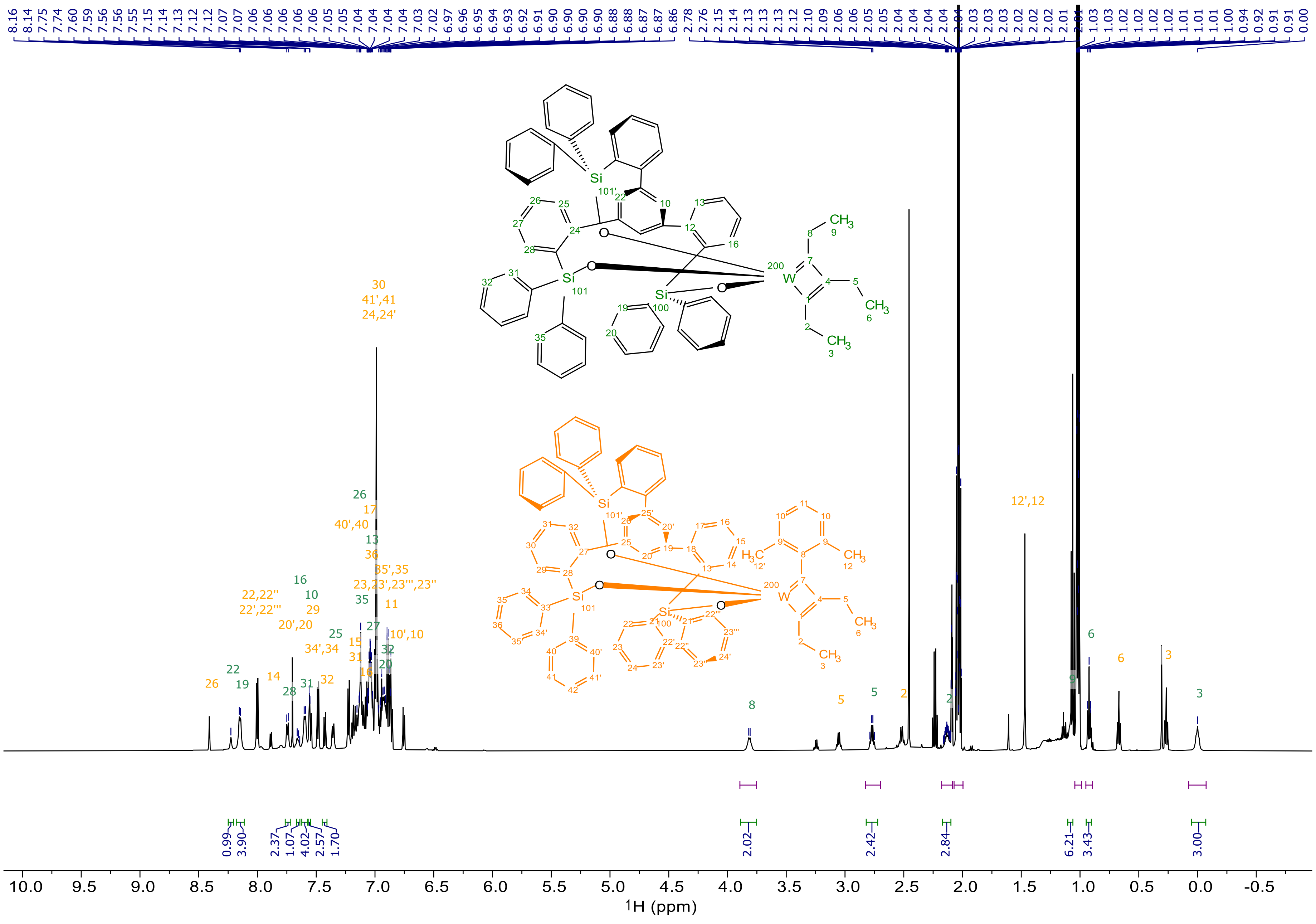

VT NMR studies of mixed metallacyclobutadiene complex 18, 600 MHz, $C_6D_5CD_3$, 25°C to −40ºC

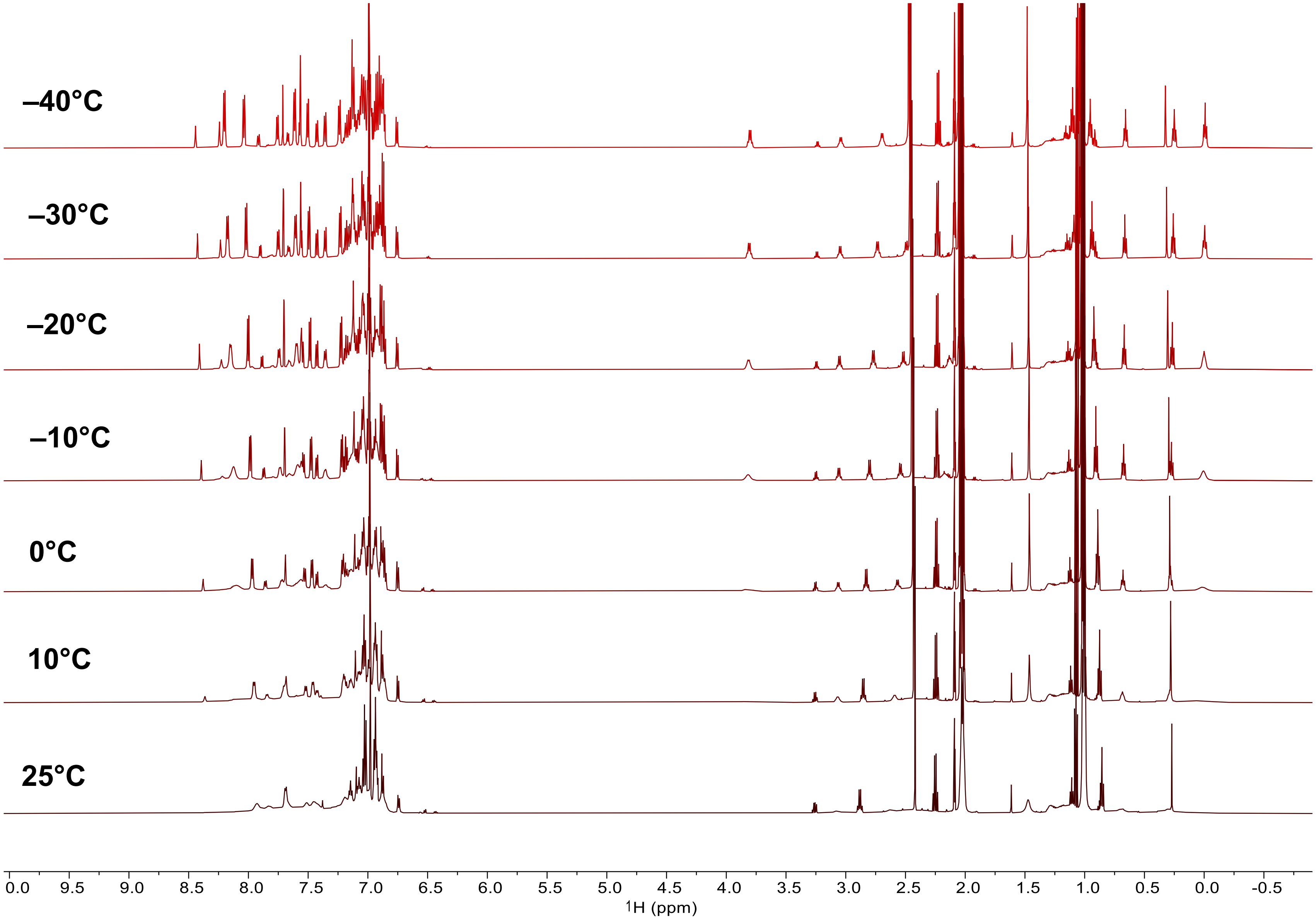

^{29}Si NMR of complex 18, 119 MHz, $C_6D_5CD_3$, −20ºC

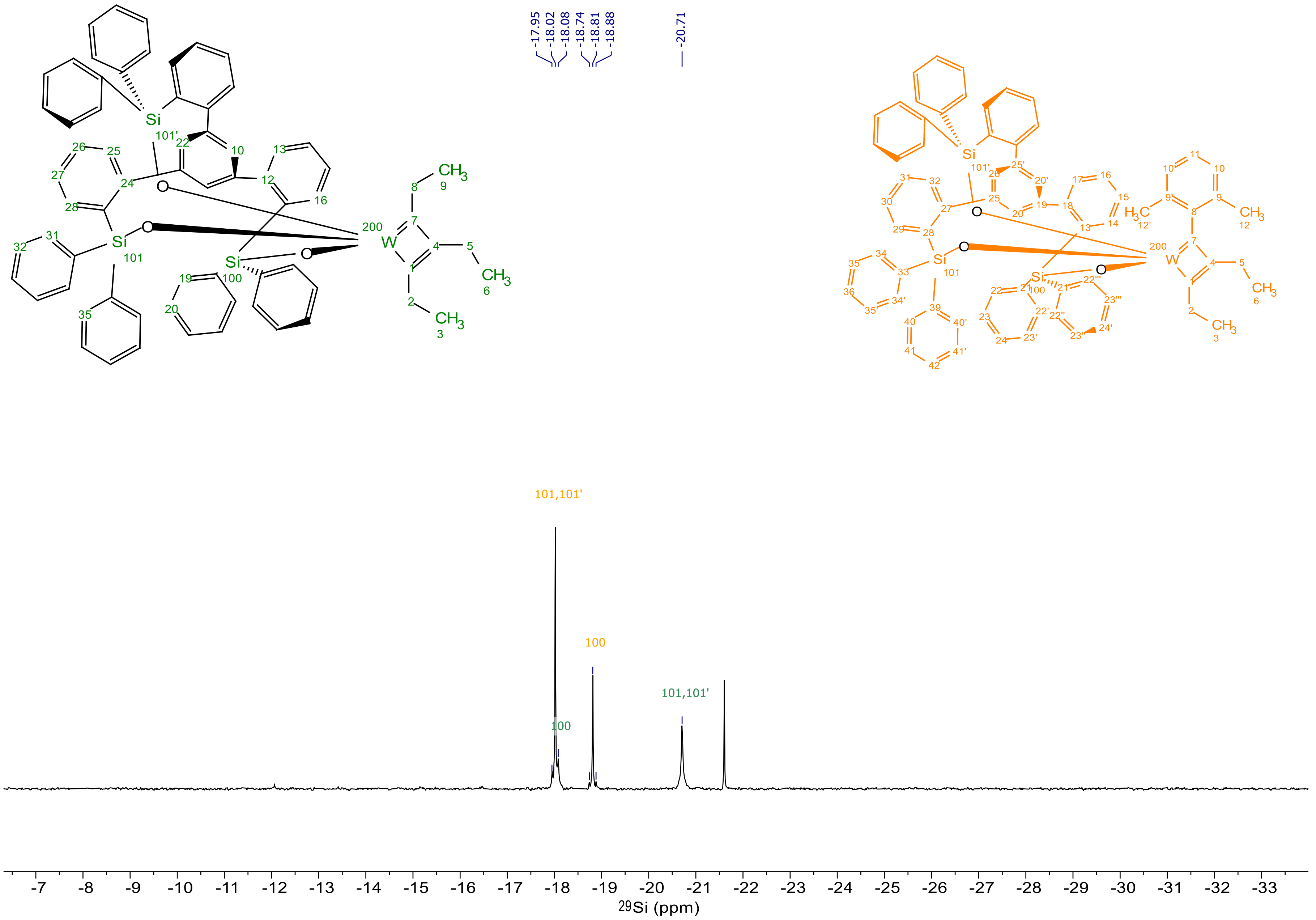

^{13}C NMR of complex 18, 151 MHz, $C_6D_5CD_3$, −20ºC

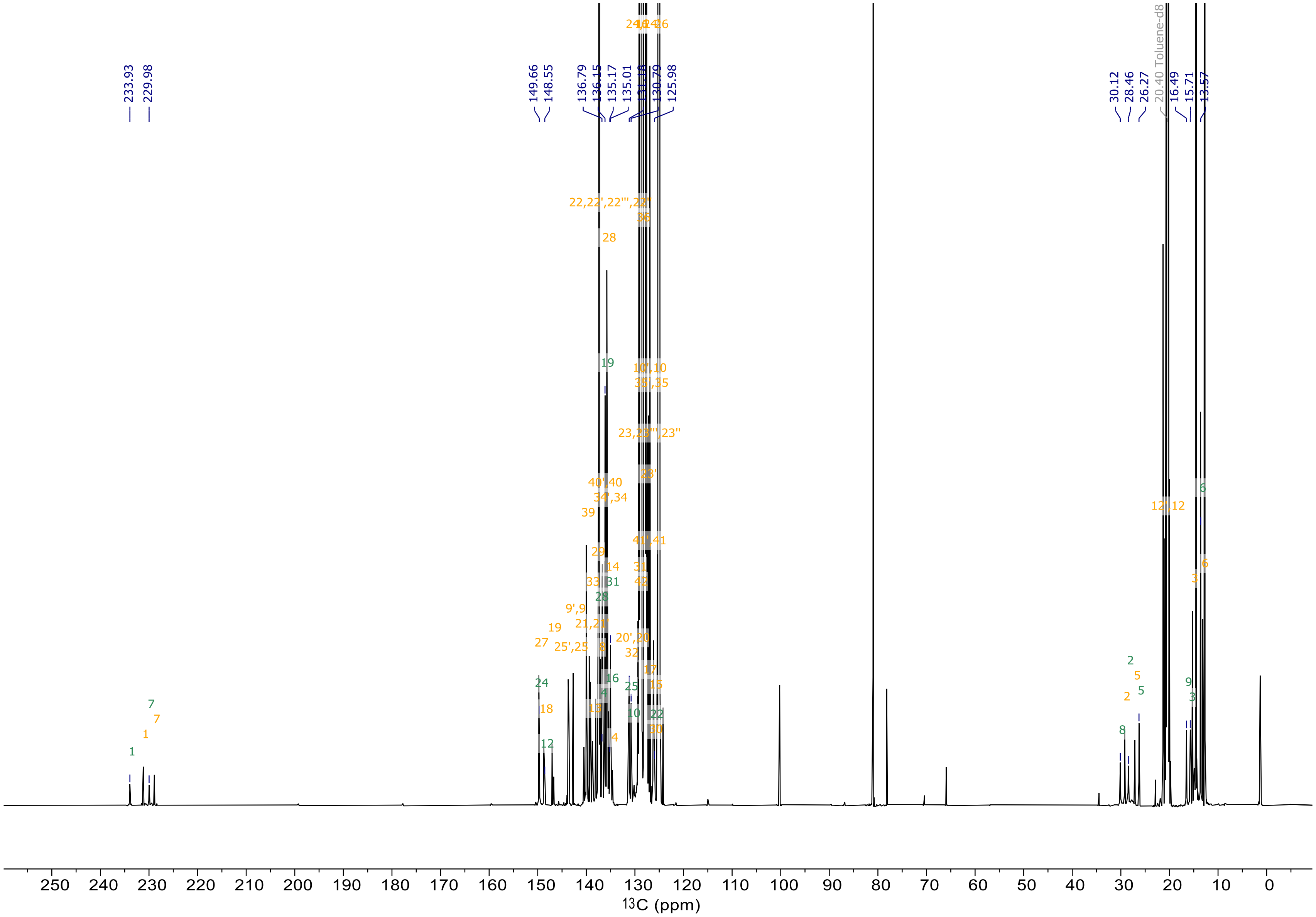

^{13}C NMR of complex 18, 151 MHz, $C_6D_5CD_3$, −20ºC

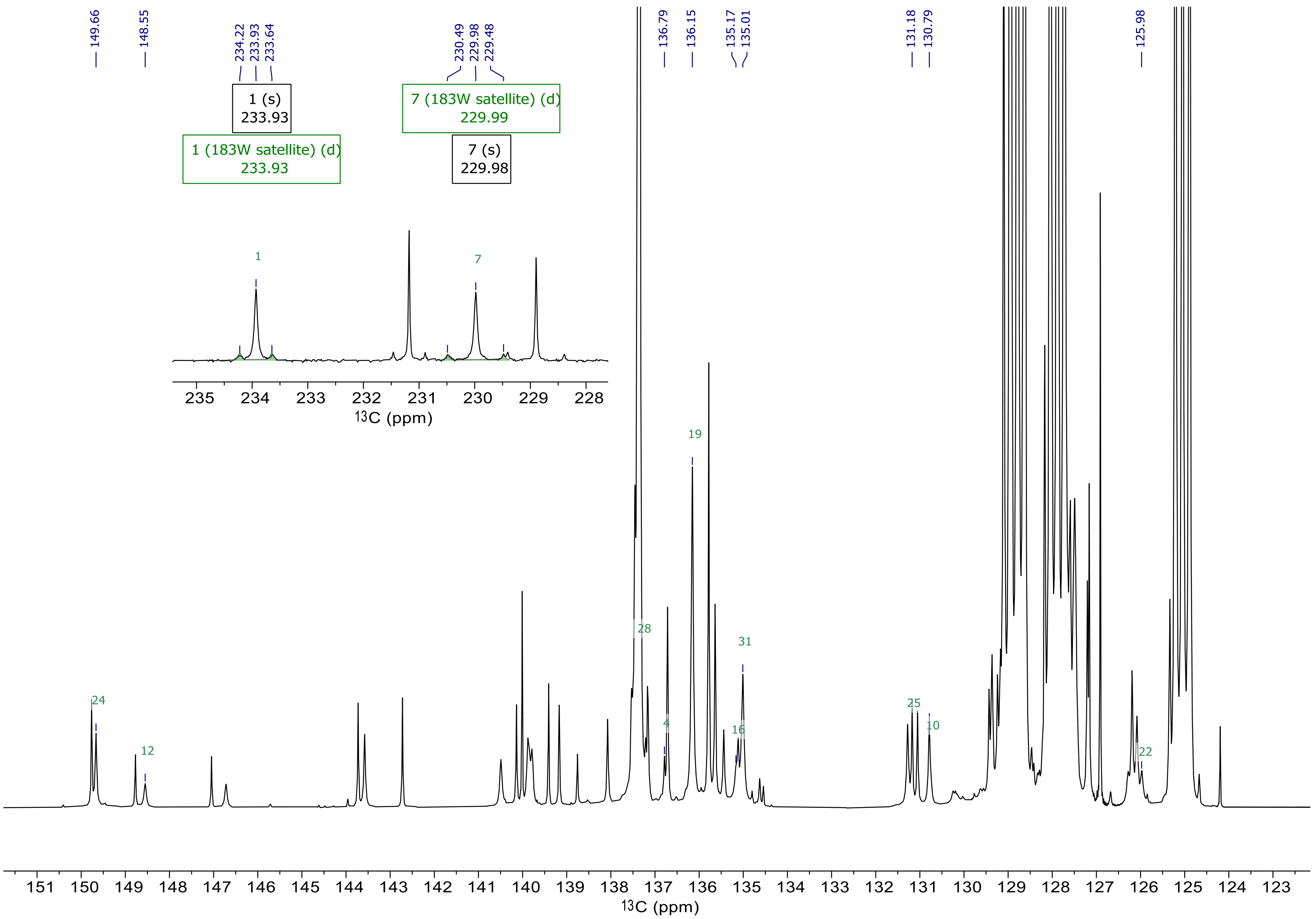

^{13}C NMR of complex 18, 151 MHz, $C_6D_5CD_3$, −20ºC

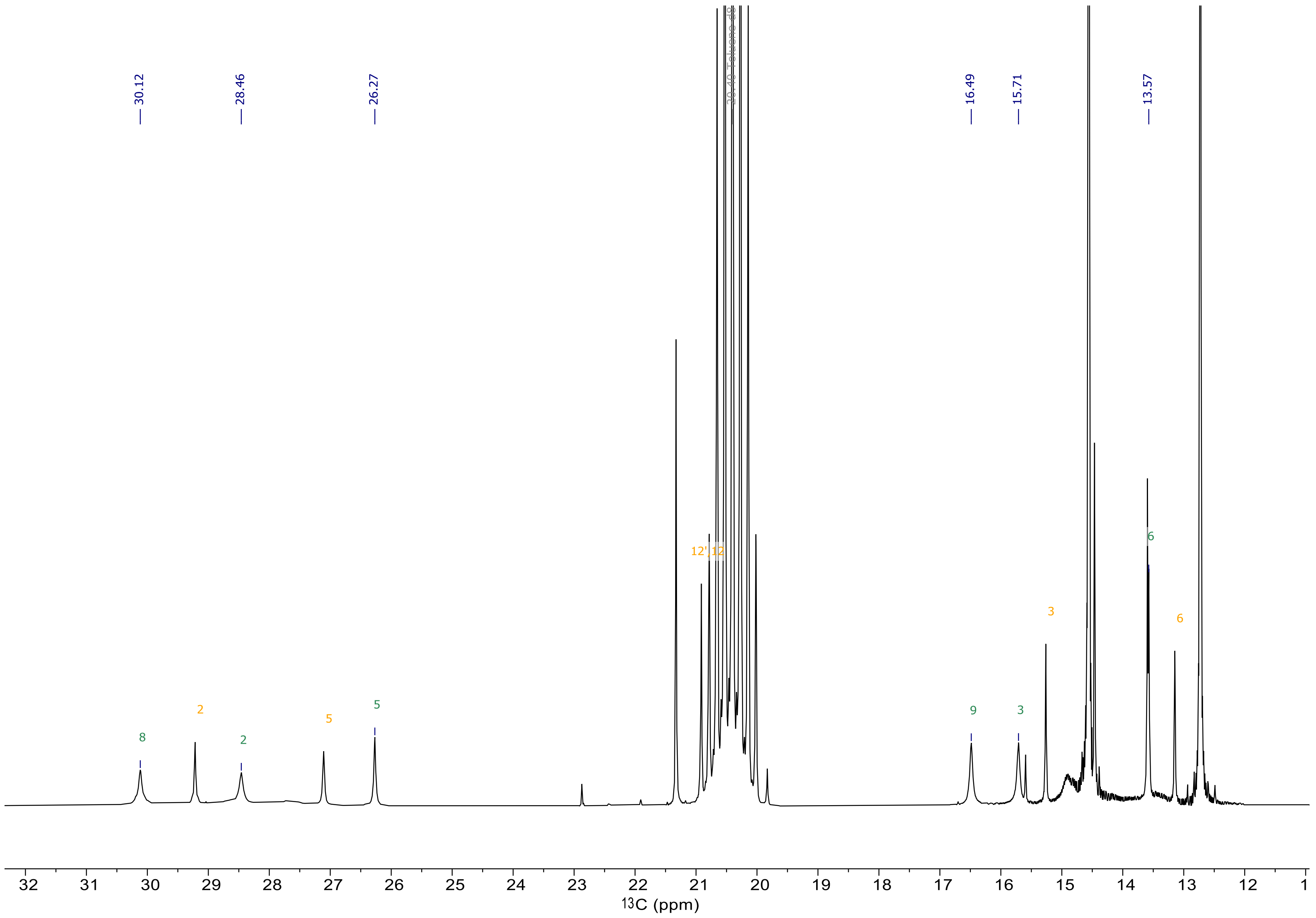

^{1}H-^{1}H COSY NMR of complex 18, $C_6D_5CD_3$, -20 °C

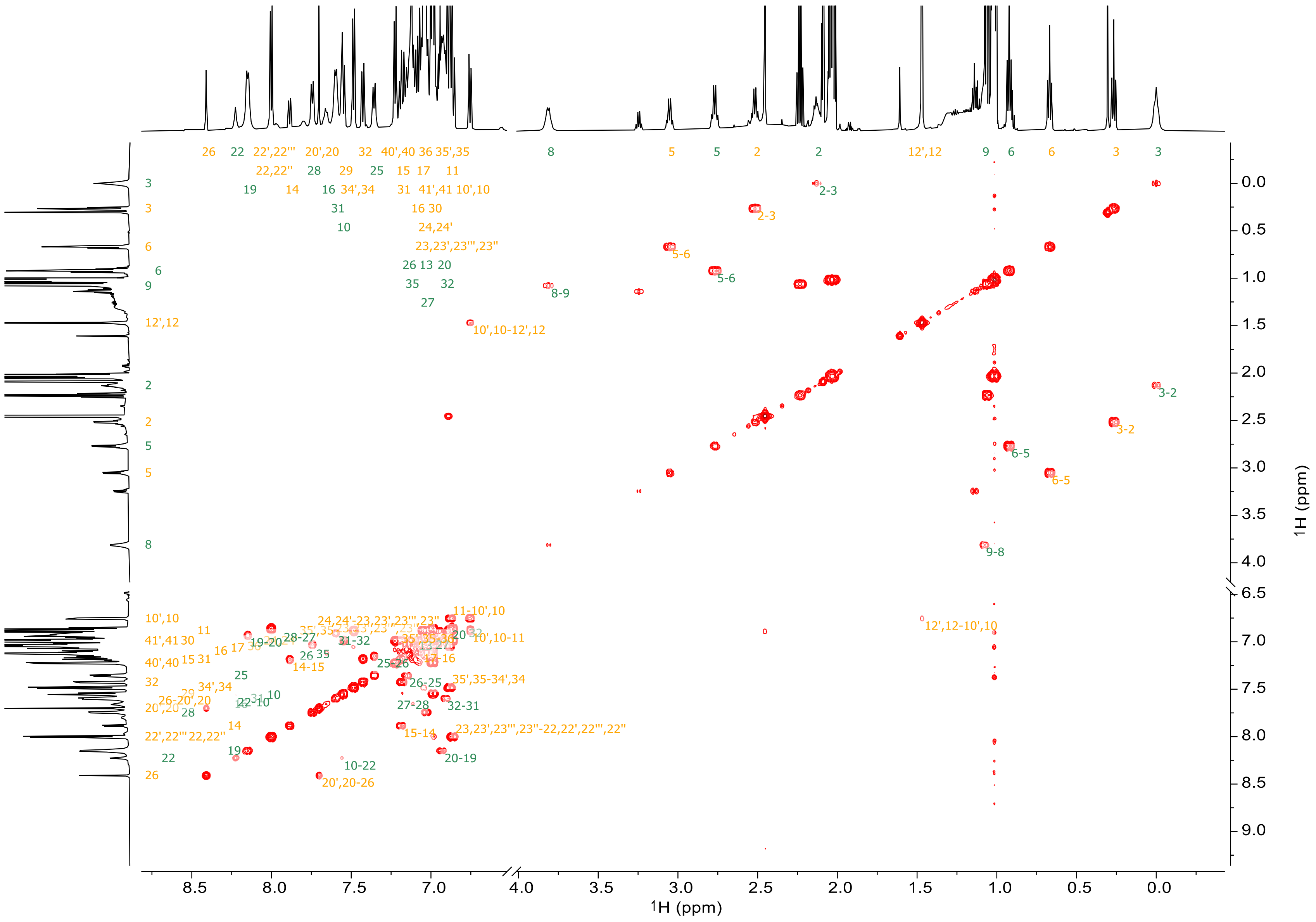

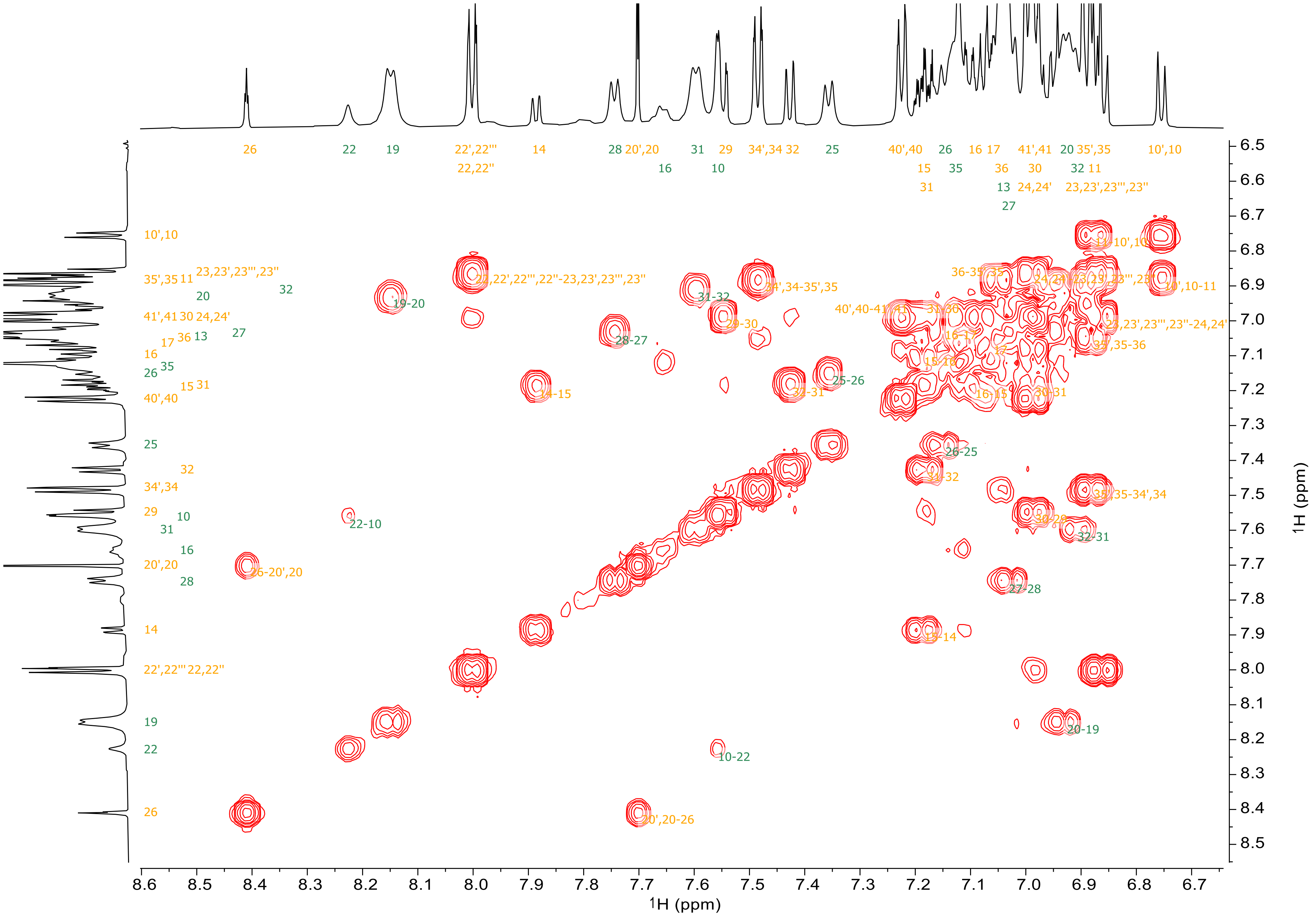
1H (ppm)
1H (ppm)

^{1}H-^{13}C edited HSQC NMR of complex 18, $C_6D_5CD_3$, -20 °C

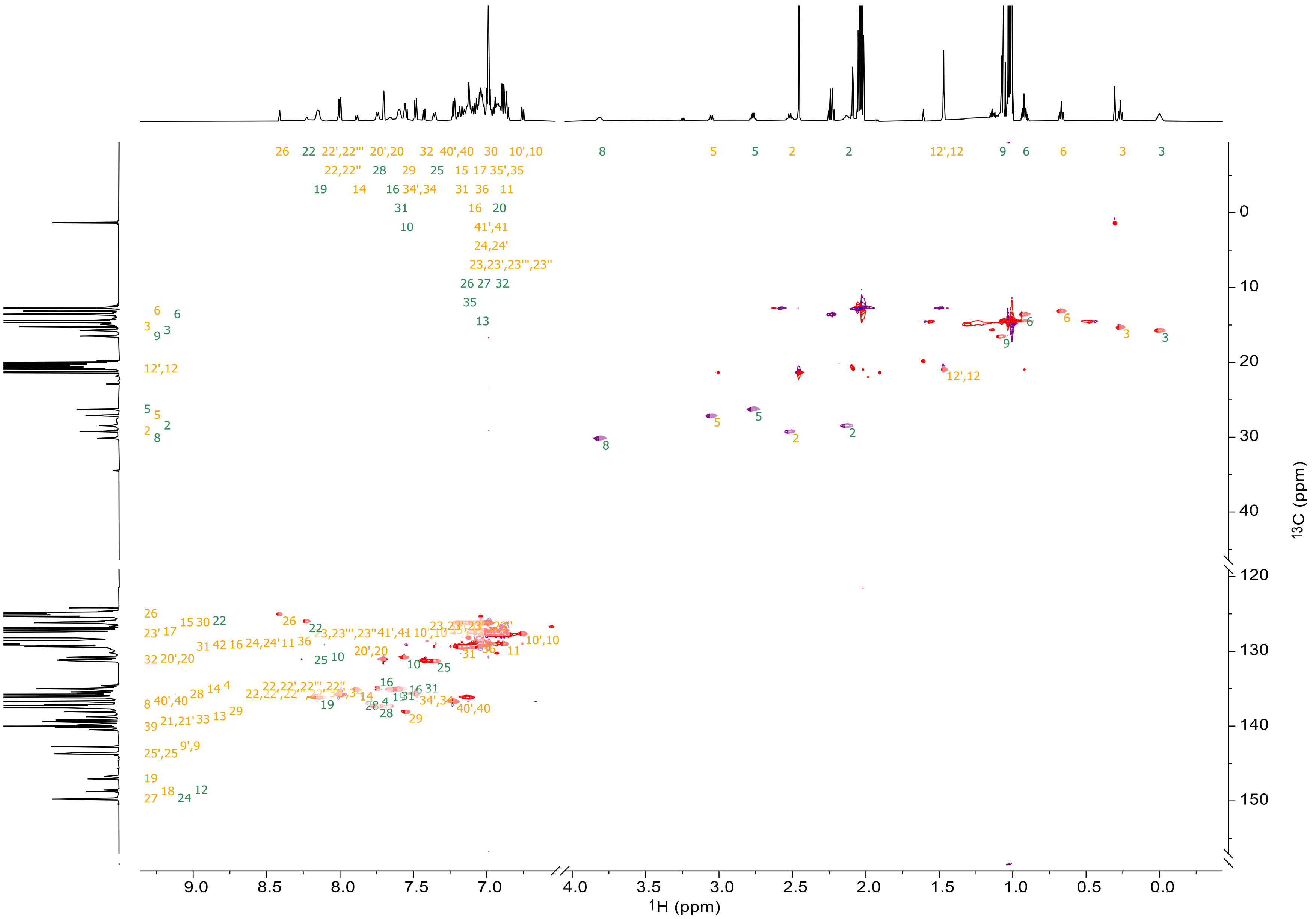

^{1}H-^{13}C edited HSQC NMR of complex 18, $C_6D_5CD_3$, -20 ºC

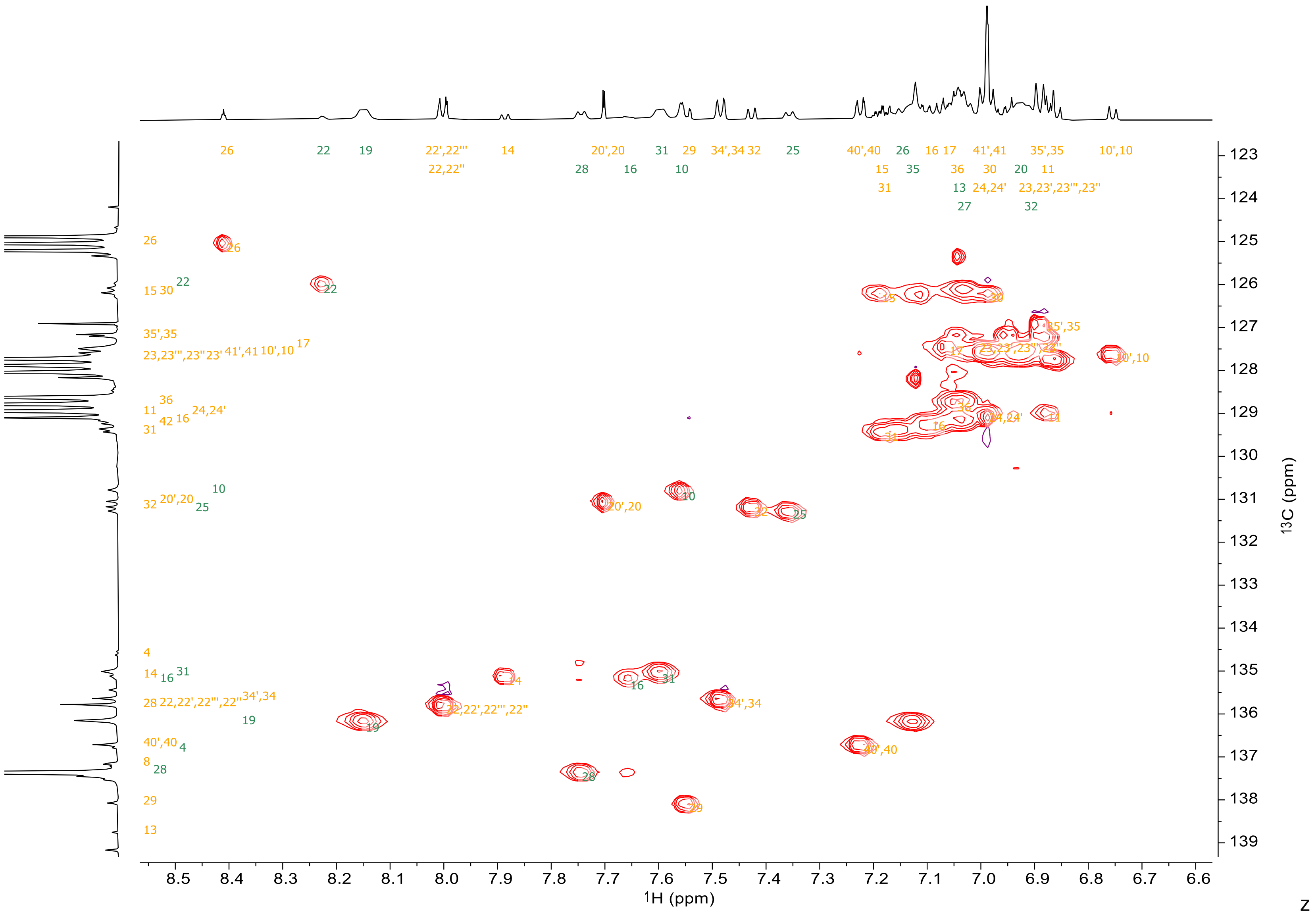

^{1}H-^{13}C HMBC NMR of complex 18, $C_6D_5CD_3$, -20 °C

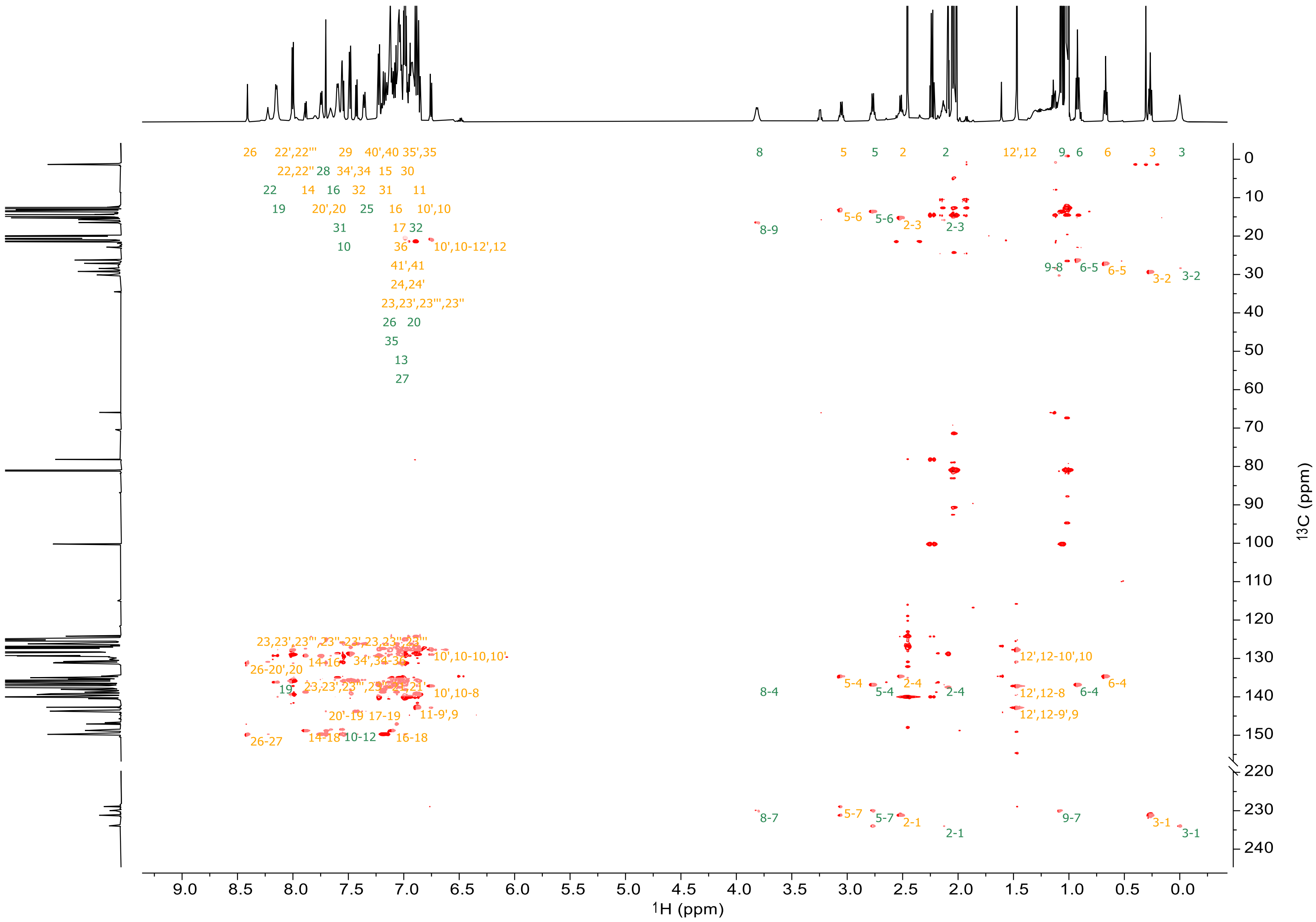

^{1}H-^{13}C HMBC NMR of complex 18, $C_6D_5CD_3$, -20 °C

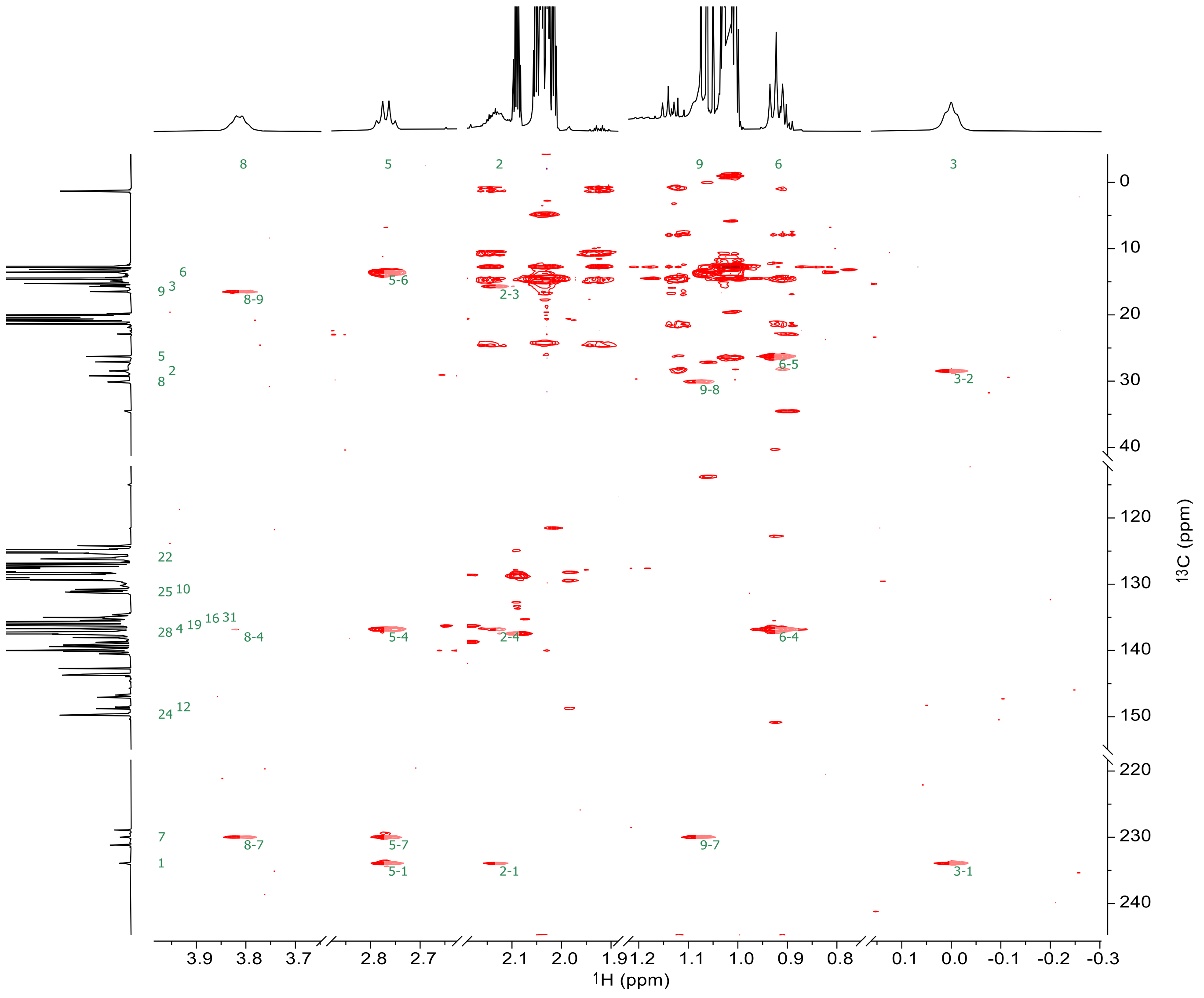

[1]H EASY ROESY of complex 18, $C_6D_5CD_3$, -20°C

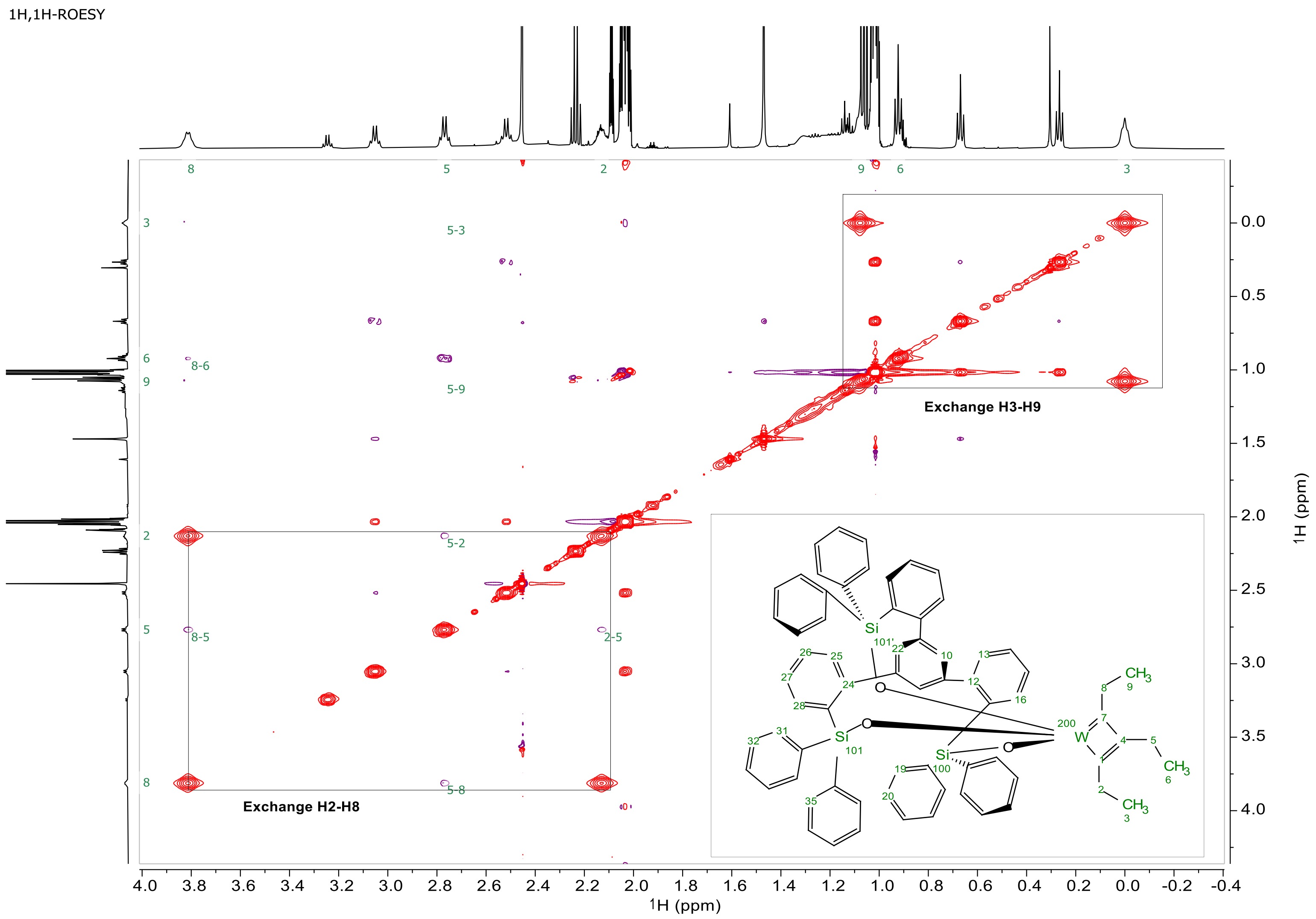

NMR assignments of complex 18, $C_6D_5CD_3$, -20°C

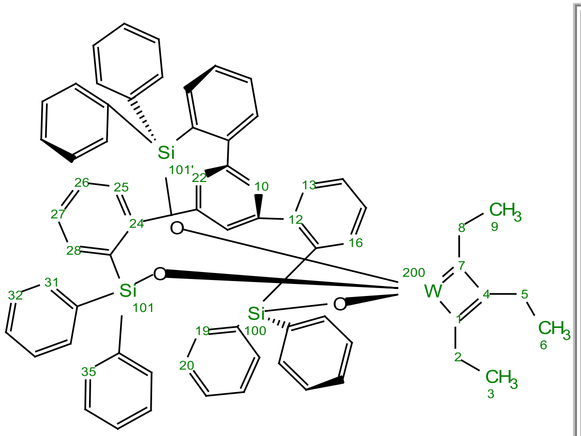

Atom	δ (ppm)	J	COSY	HSQC	HMBC	NOESY
1 C	233.93	89.20(200)			2, 3, 5	
2 C	28.46			2	3	
H2	2.13		3	2	1, 3, 4	5, 10, 22
3 C	15.71			3	2	
H3	0.00		2	3	1, 2	5, 19, 31, 35
4 C	136.79				2, 5, 6, 8	
5 C	26.27			5	6	
H2	2.77		6	5	1, 4, 6, 7	2, 3, 8, 9, 19, 31, 35
6 C	13.57			6	5	
H3	0.92		5	6	4, 5	8, 19, 31, 35
7 C	229.98	152.00(200)			5, 8, 9	
8 C	30.12			8	9	
H2	3.81		9	8	4, 7, 9	5, 6, 10, 19, 22, 31, 35
9 C	16.49			9	8	
H3	1.08		8	9	7, 8	5, 19, 31, 35
10 C	130.76			10		
H	7.56		22	10	12	2, 8
12 C	148.55				10	
13 C				13		
H	7.04			13		22
16 C	135.17			16		
H	7.66			16		
19 C	136.15			19	19	
H	8.15		20	19	19	3, 5, 6, 8, 9

Atom	δ (ppm)	J	COSY	HSQC	HMBC	NOESY
20 C						
H	6.93		19			
22 C	125.94			22		
H	8.23		10	22		2, 8, 13, 25
24 C	149.66					
25 C	131.19			25		
H	7.35		26	25		22
26 C						
H	7.15		25			
27 C						
H	7.04		28			
28 C	137.29			28		
H	7.75		27	28		
31 C	135.01			31		
H	7.60		32	31		3, 5, 6, 8, 9
32 C						
H	6.91		31			
35 C				35		
H	7.13			35		3, 5, 6, 8, 9
100 Si	-18.08					
101 Si	-20.71					
101' Si	-20.71					
200 W		152.00(7), 89.20(1)				

Remarks: Not all ^{13}C and ^{1}H NMR shifts could be assigned due to signal broadening and overlaps with complex 17. Where no isolated ^{1}H multiplet or ^{13}C signal was observable, shifts were extracted from the 2D data.

^{1}H NMR of Complex 8b, 600 MHz, $C_6D_5CD_3$, 25ºC

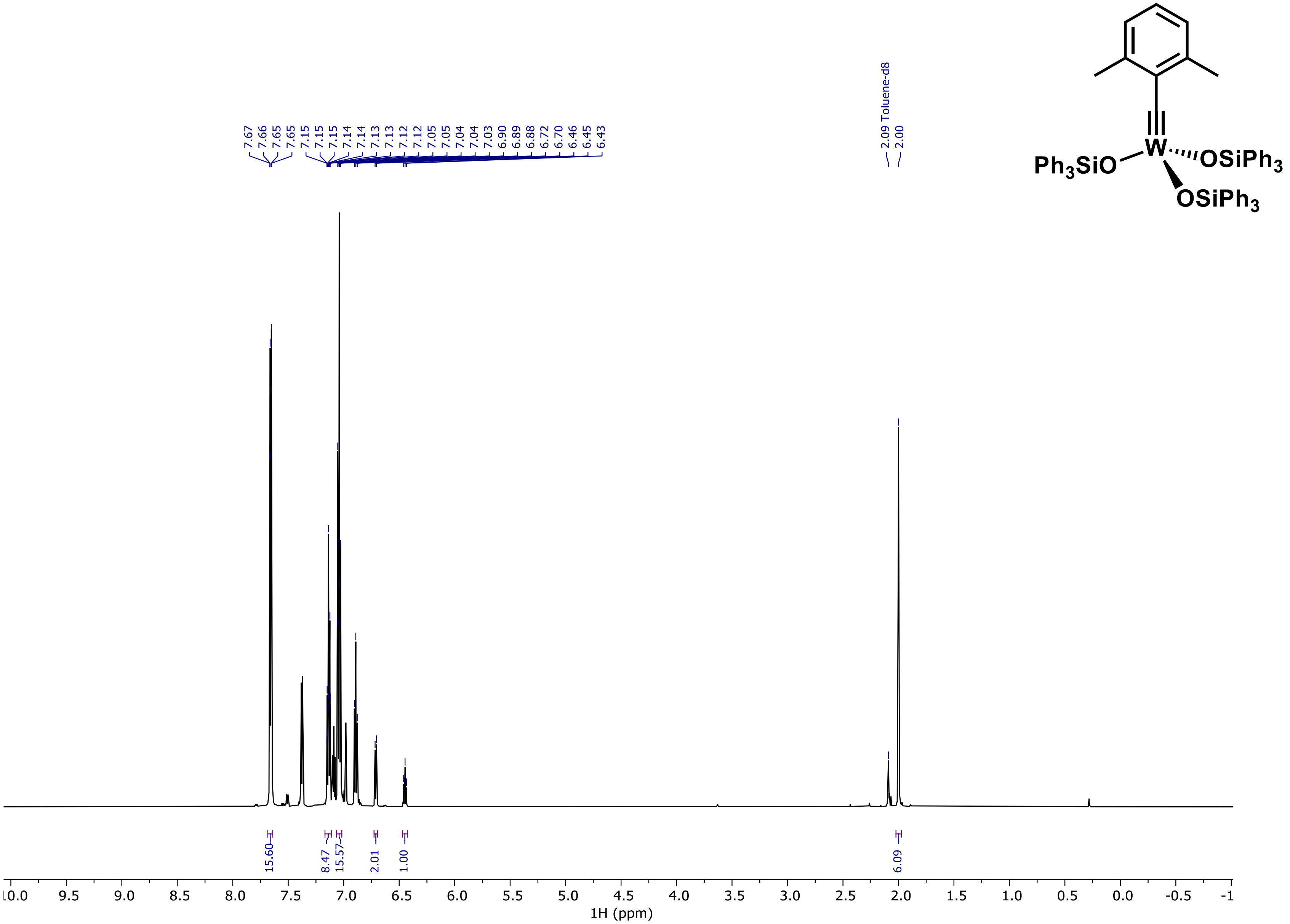

^{13}C NMR of Complex 8b, 151 MHz, $C_6D_5CD_3$, 25ºC

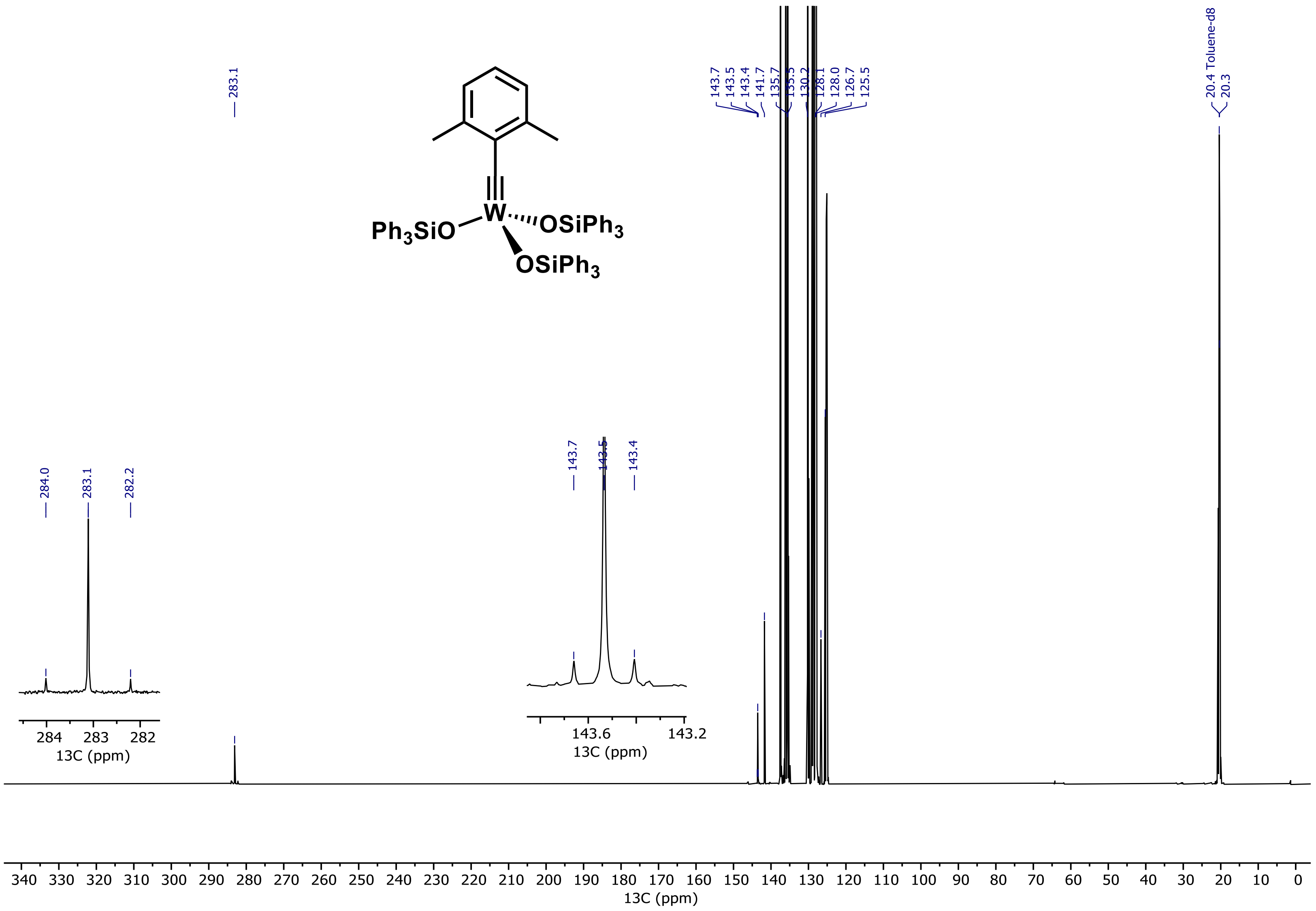

^{29}Si NMR of Complex 8b, 119 MHz, $C_6D_5CD_3$, 25ºC

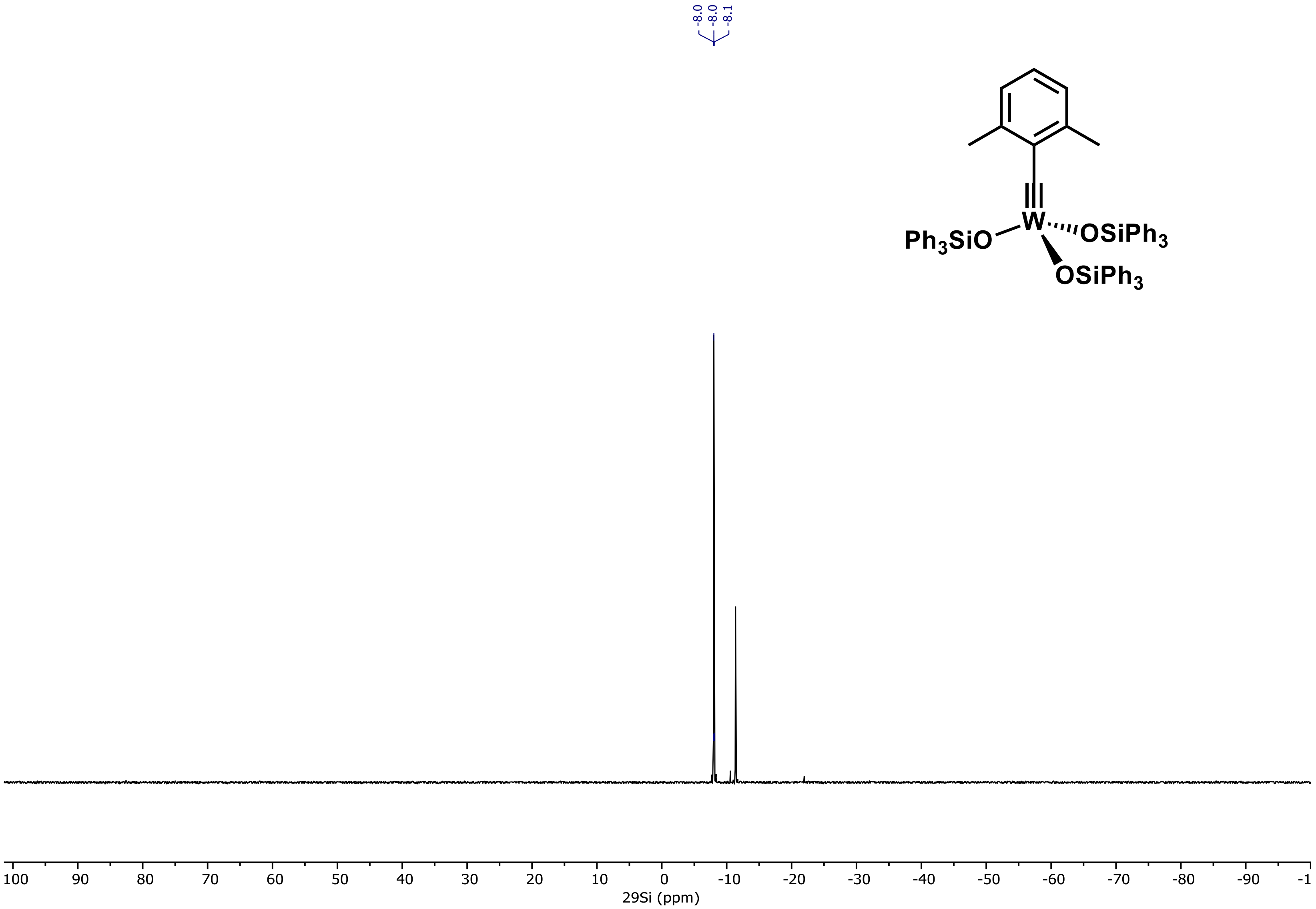

^{183}W NMR Projection created from 2D-HMBC Experiment of Complex 8b, 400 MHz, $C_6D_5CD_3$, 25ºC

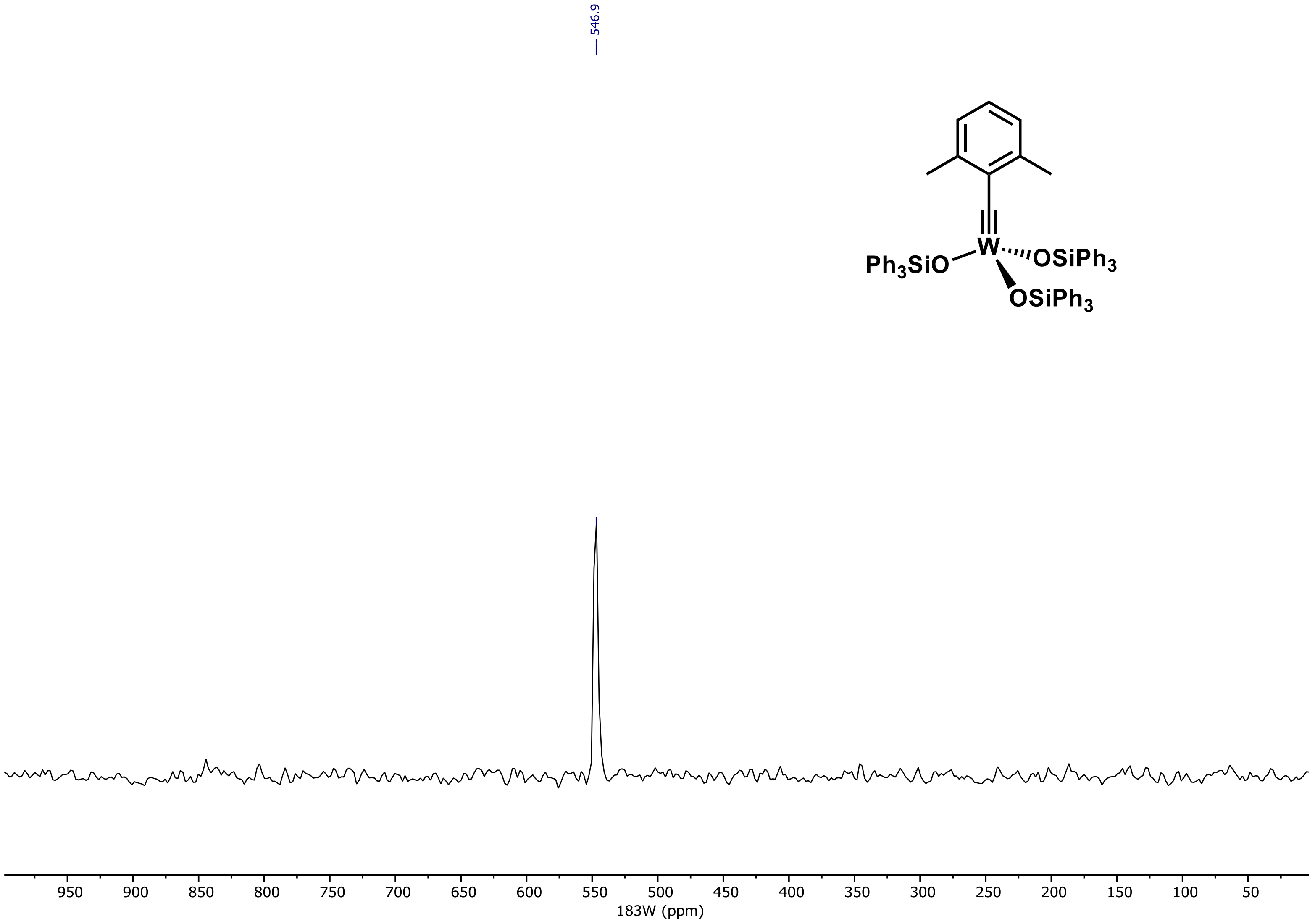

^{1}H NMR of Complex 19 in crude reaction mixture, 600 MHz, C_6D_6, 25ºC

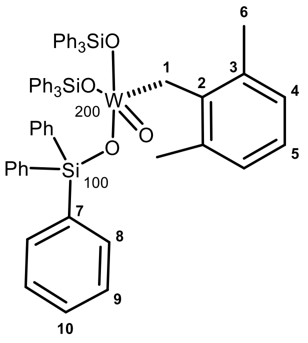

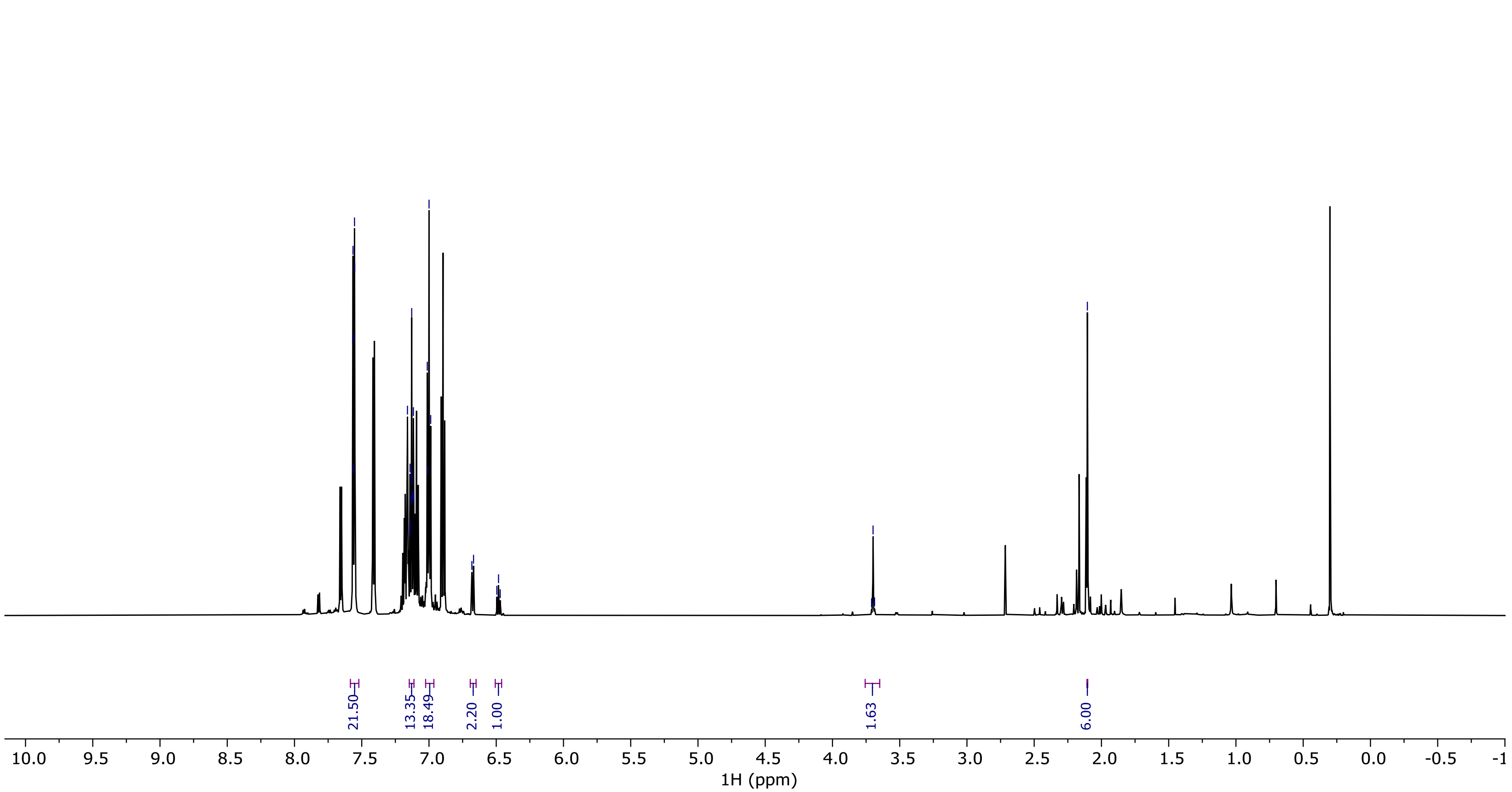

^{13}C NMR of Complex 19 in crude reaction mixture, 151 MHz, C_6D_6, 25ºC

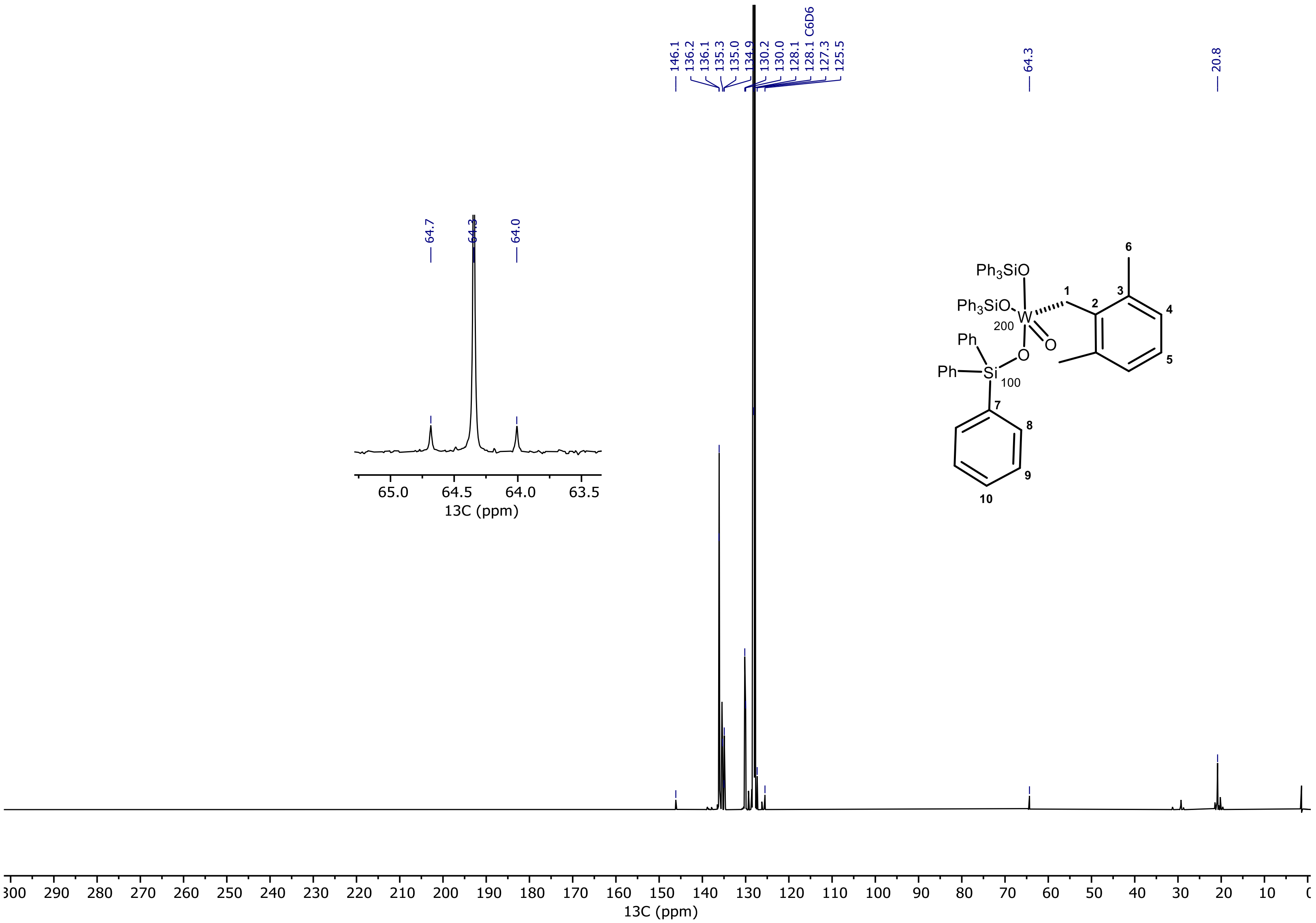

^{29}Si NMR of Complex 19 in crude reaction mixture, 151 MHz, C_6D_6, 25ºC

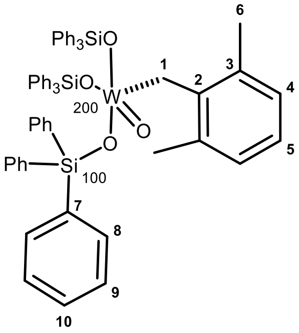

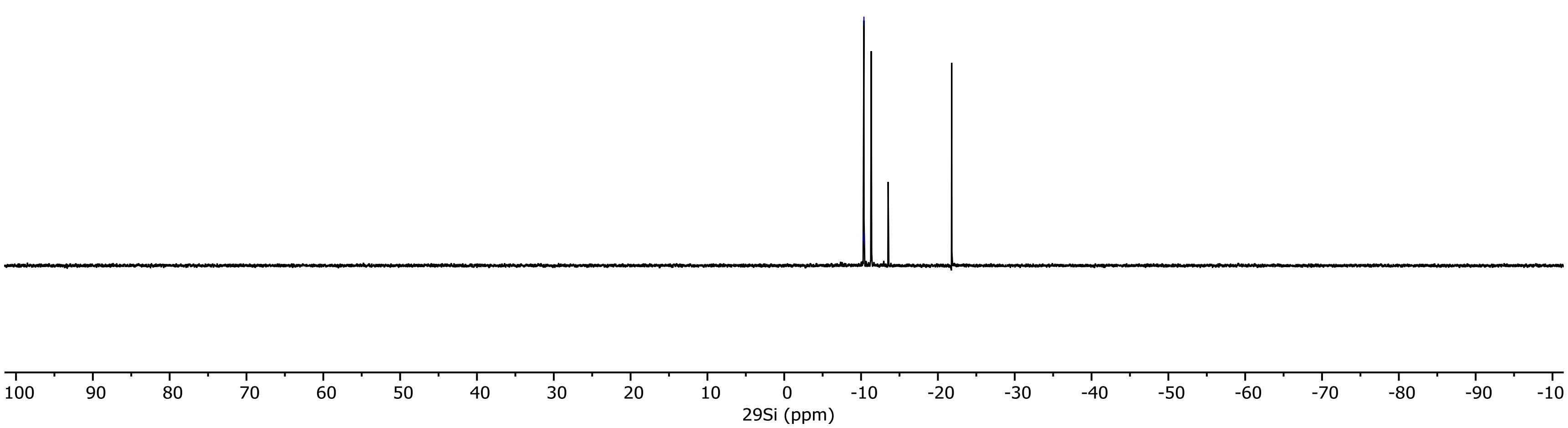

^{1}H-^{13}C edited HSQC NMR of complex 19 in crude reaction mixture, C_6D_6, 25 ºC

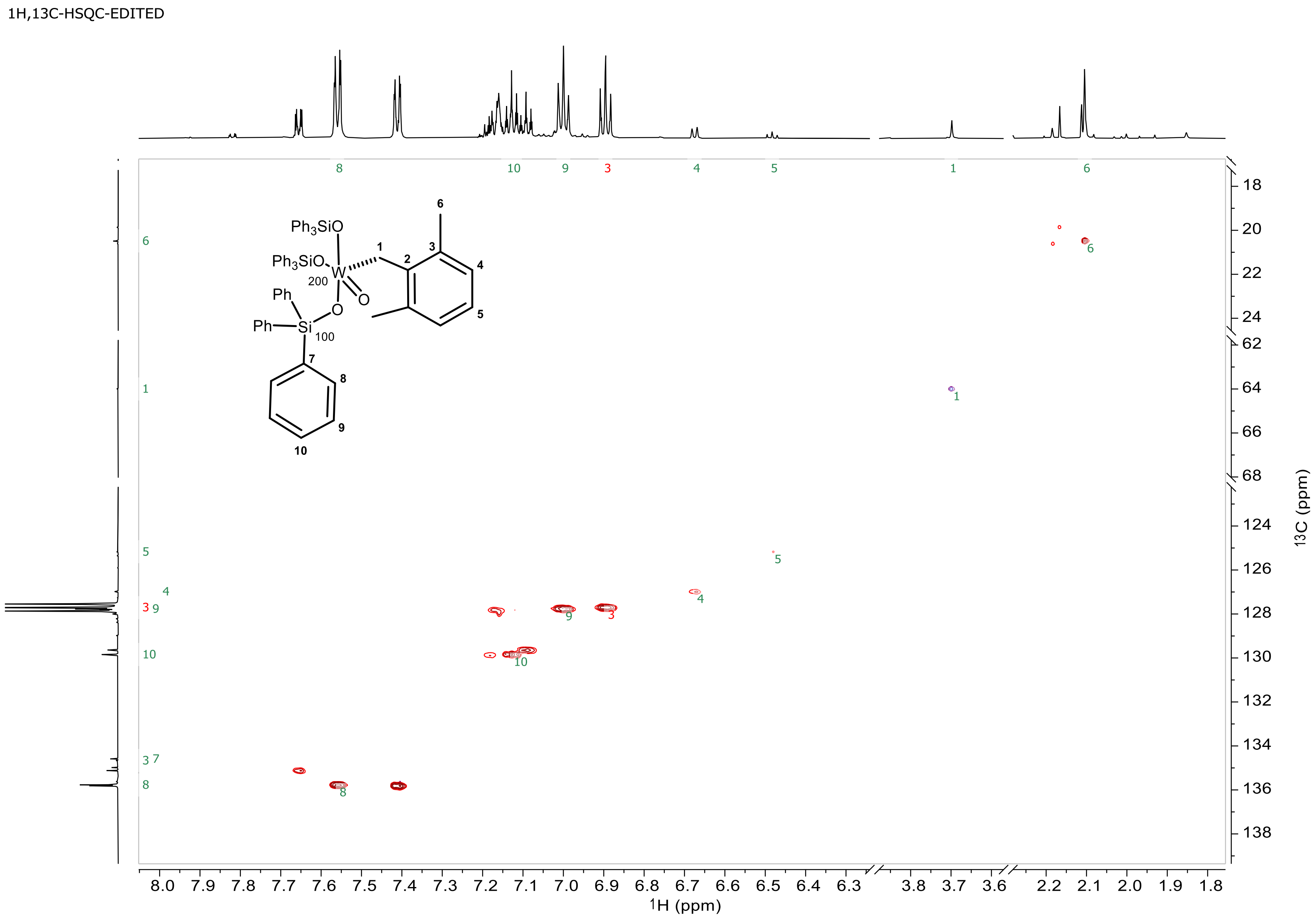

^{1}H-^{13}C HMBC NMR of complex 19 in crude reaction mixture, C_6D_6, 25 ºC

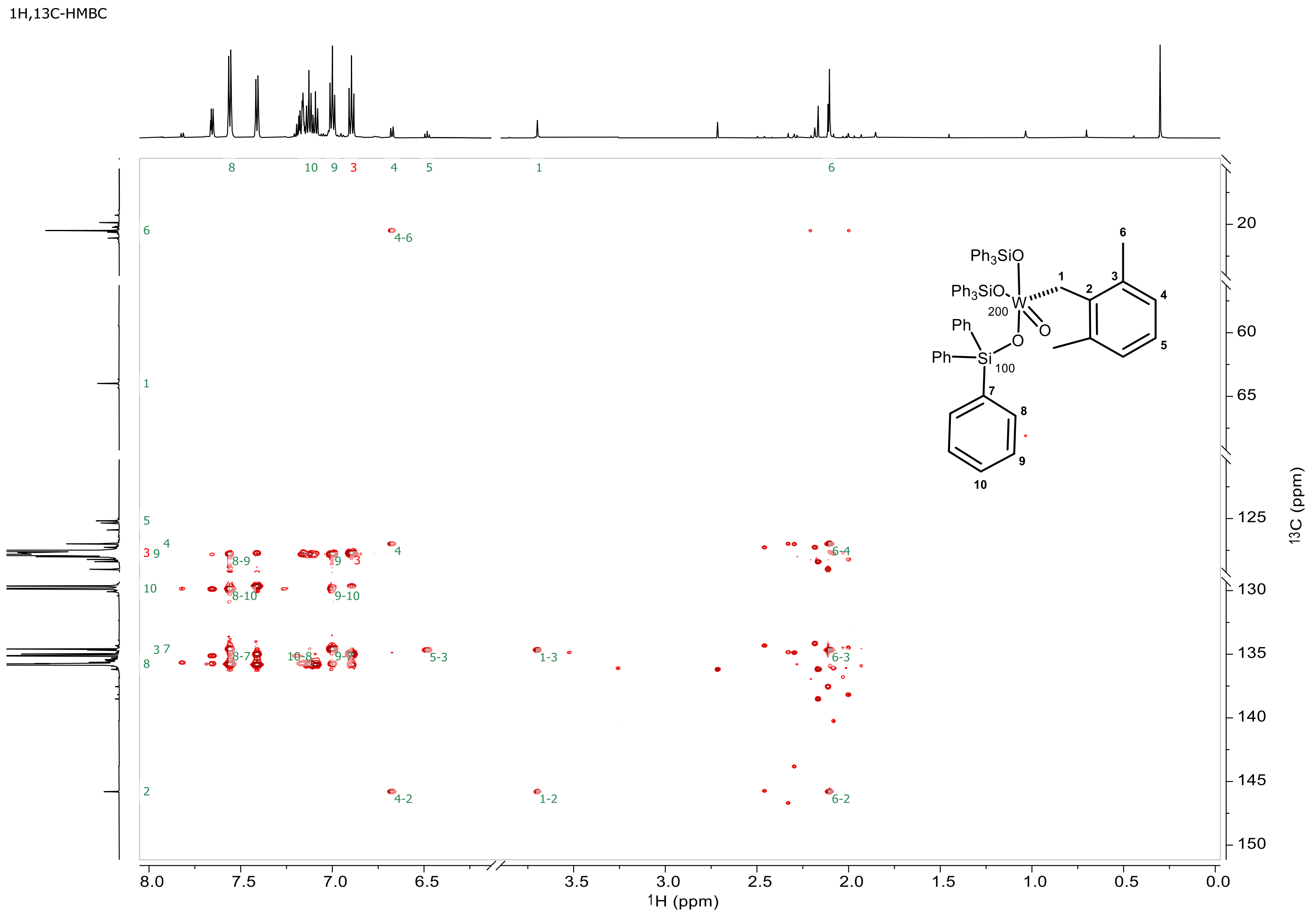

^{1}H-^{29}Si HMBC NMR of complex 19 in crude reaction mixture, C_6D_6, 25 ºC

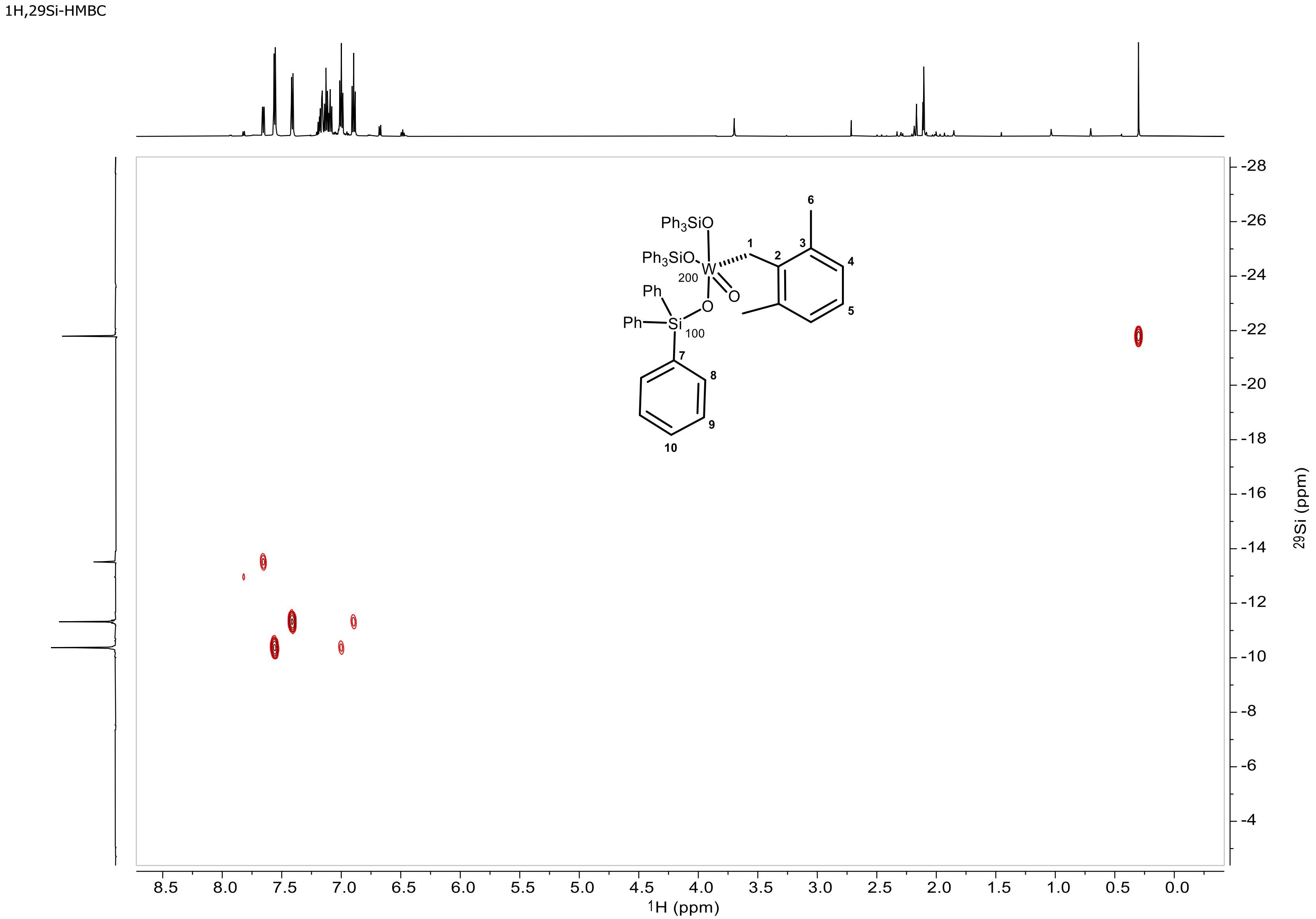

^{1}H-^{1}H COSY NMR of complex 19 in crude reaction mixture, C_6D_6, 25 °C

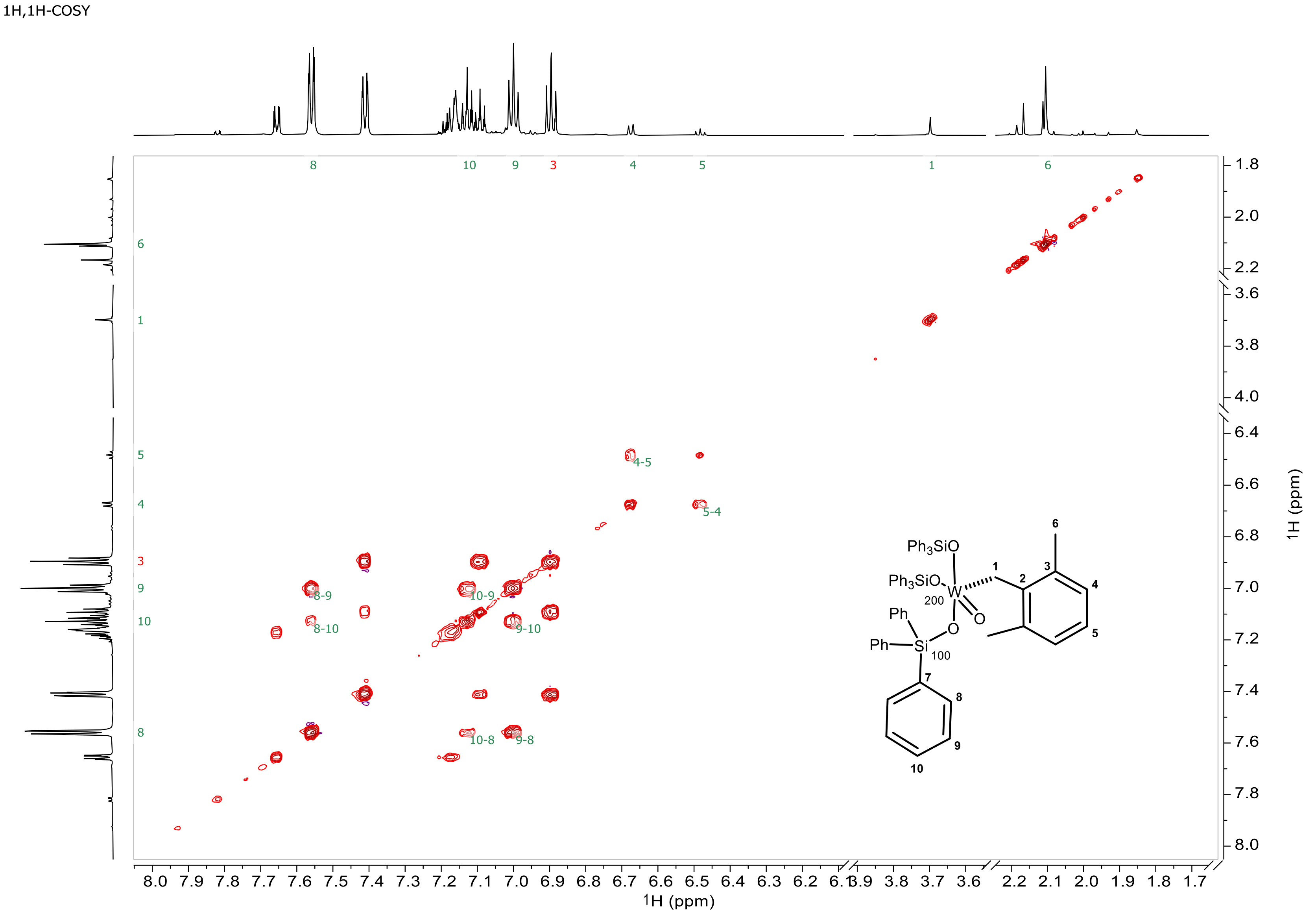

¹H EASY ROESY of complex 19 in crude reaction mixture, C_6D_6, 25°C

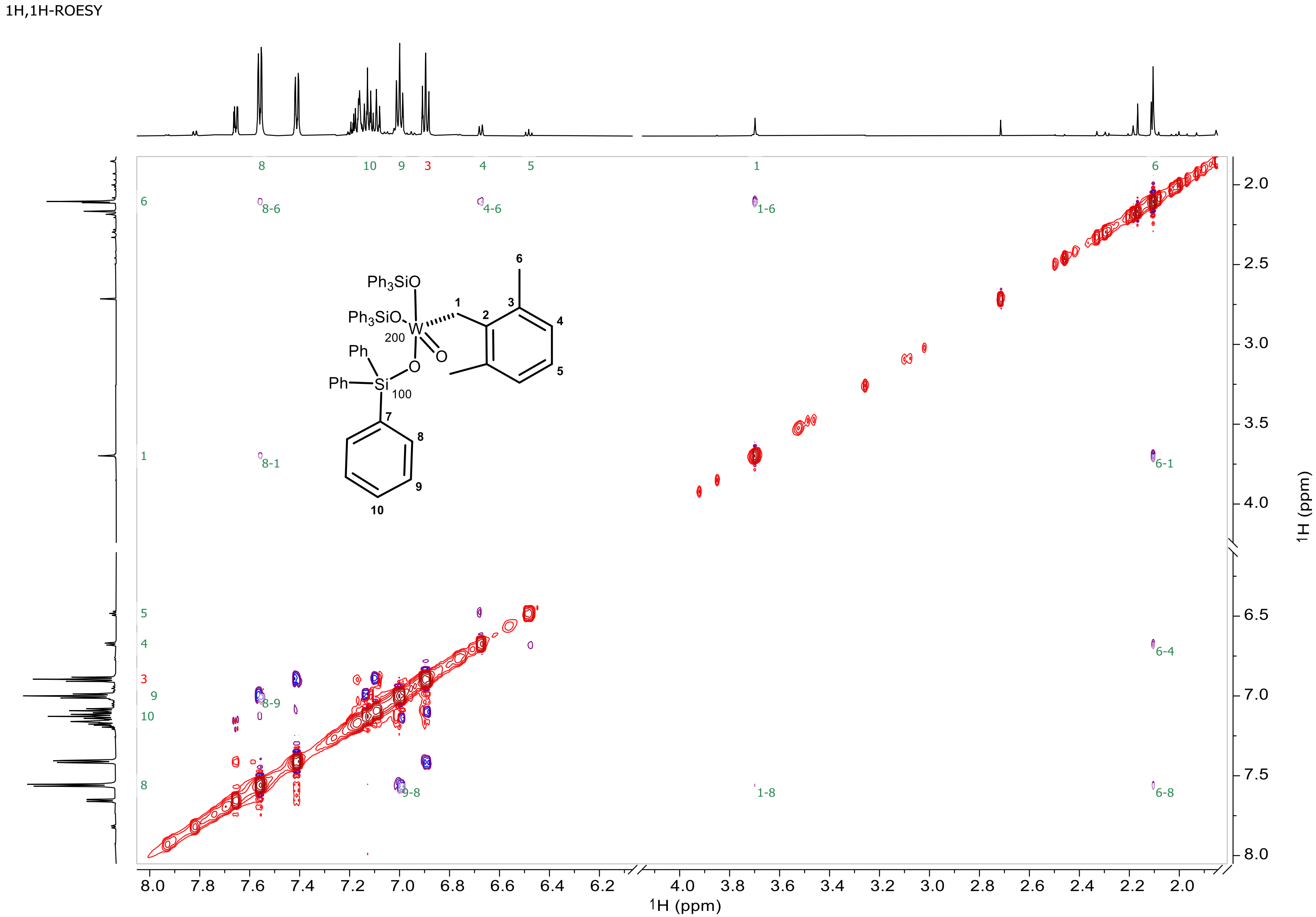

^{1}H ^{183}W NMR Experiment of Complex 19 in crude reaction mixture, C_6D_6, 25ºC

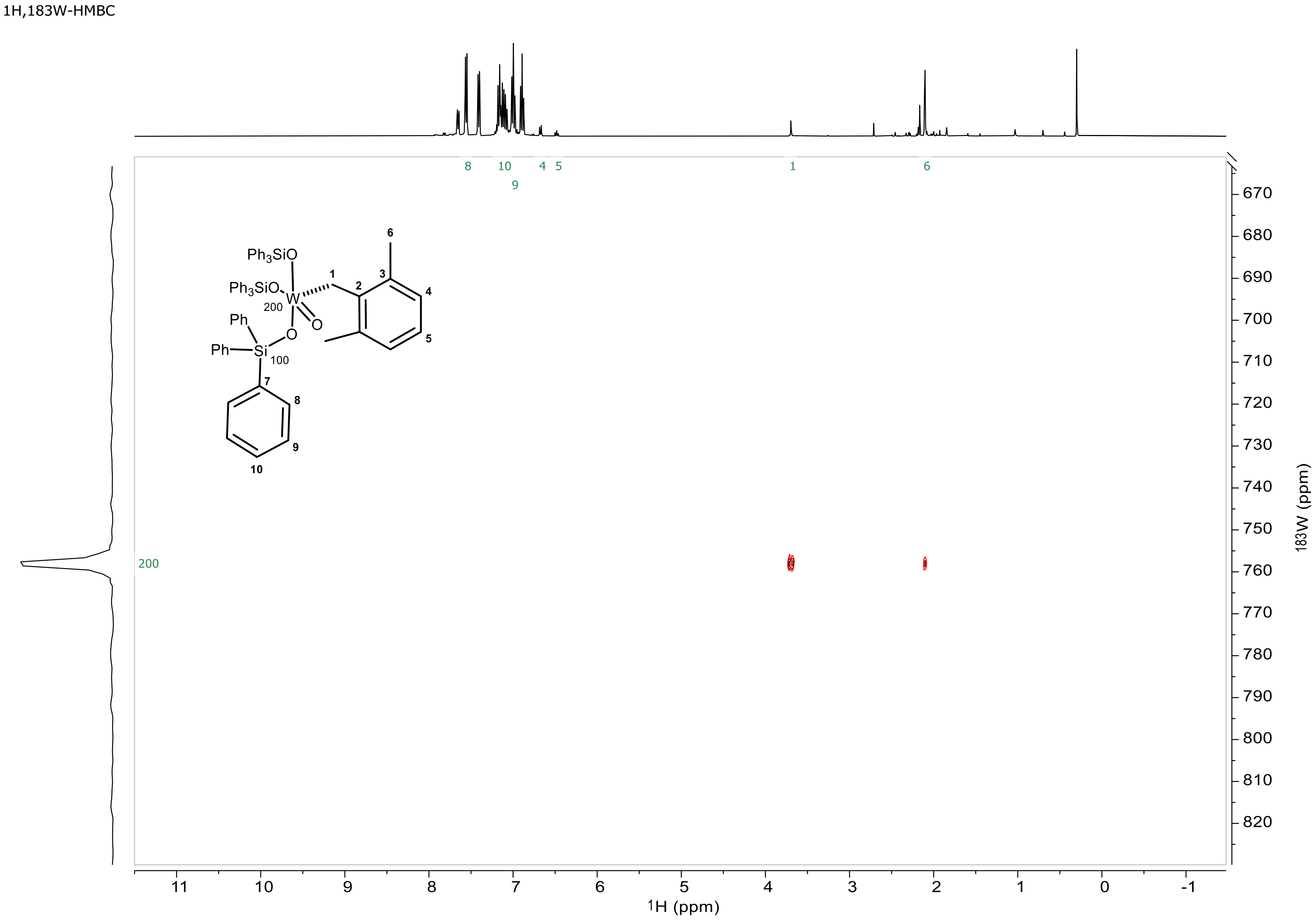

^{183}W NMR Projection created from 2D-HMBC Experiment of Complex 19, 17 MHz, C_6D_6, 25ºC

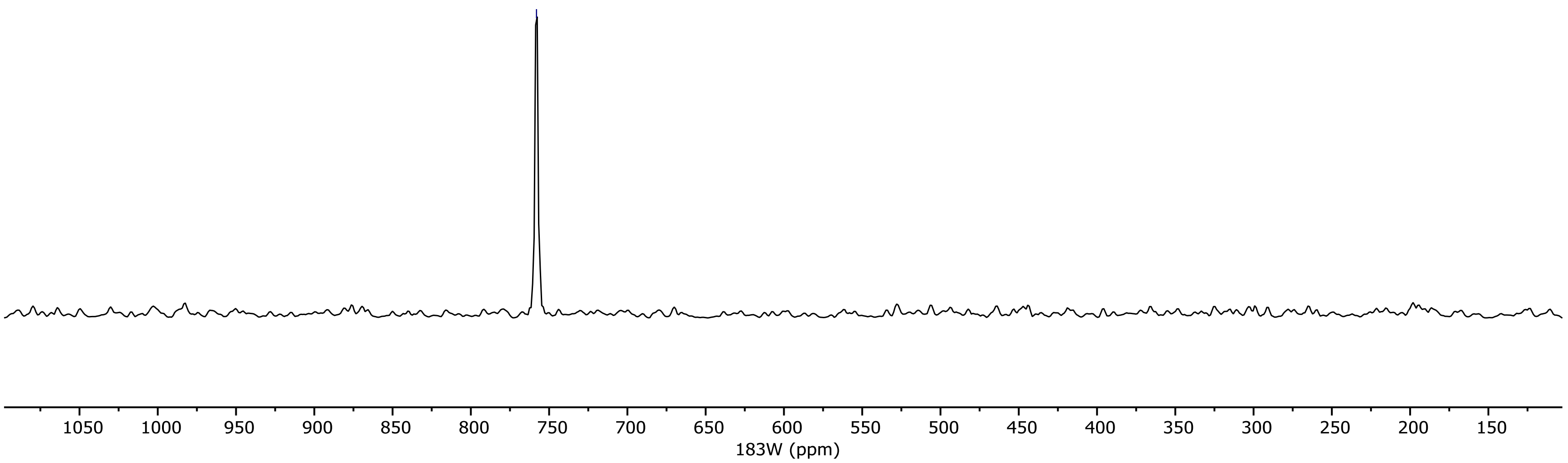

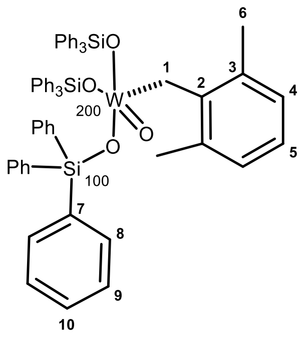

^{1}H NMR of Complex 10b, 600 MHz, $C_6D_5CD_3$, 25ºC

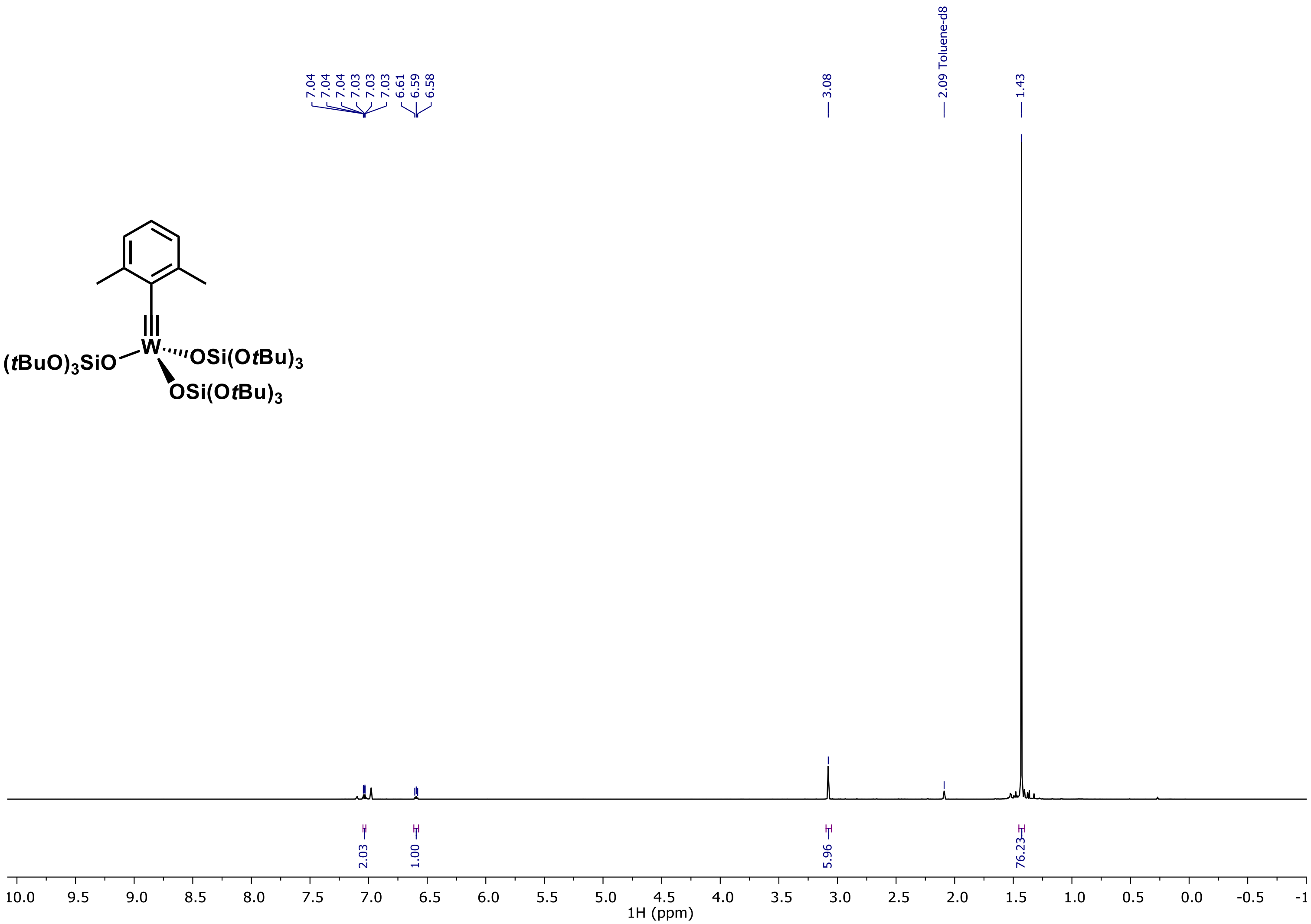

^{13}C NMR of Complex 10b, 151 MHz, $C_6D_5CD_3$, 25ºC

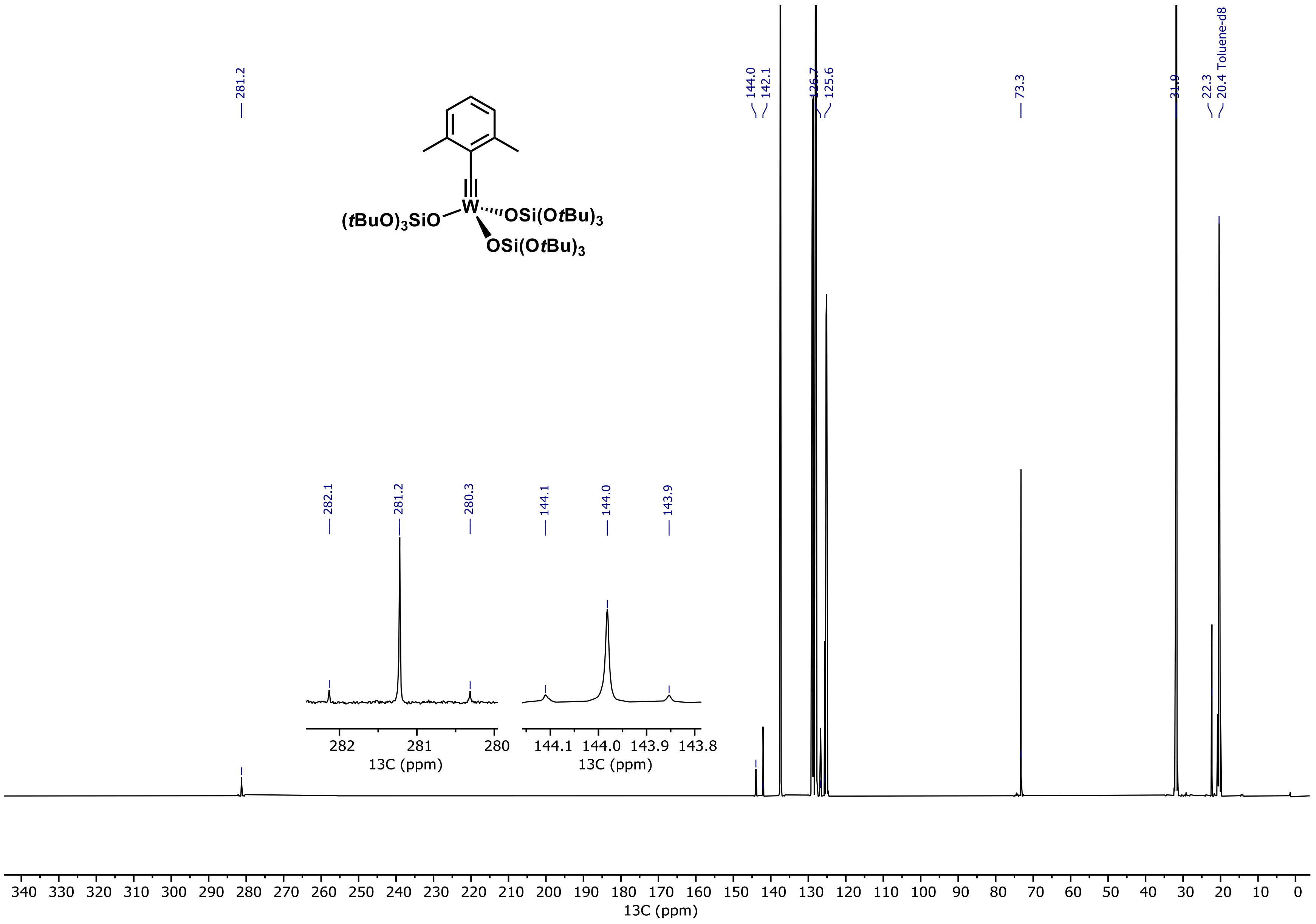

^{29}Si NMR of Complex 10b, 119 MHz, $C_6D_5CD_3$, 25ºC

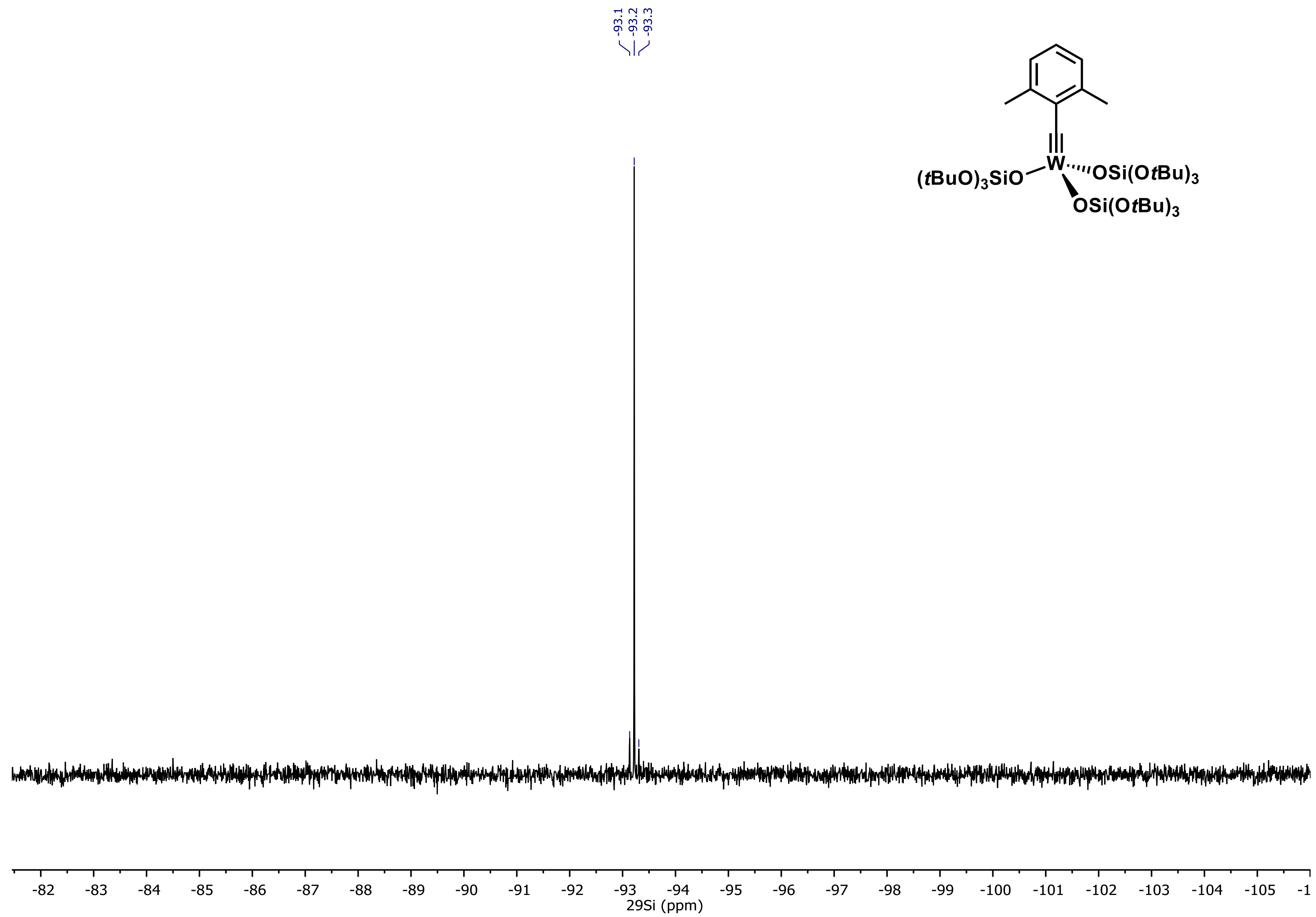

^{1}H-^{183}W HMBC NMR of Complex 10b, 400 MHz, $C_6D_5CD_3$, 25ºC

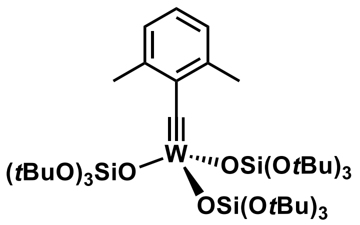

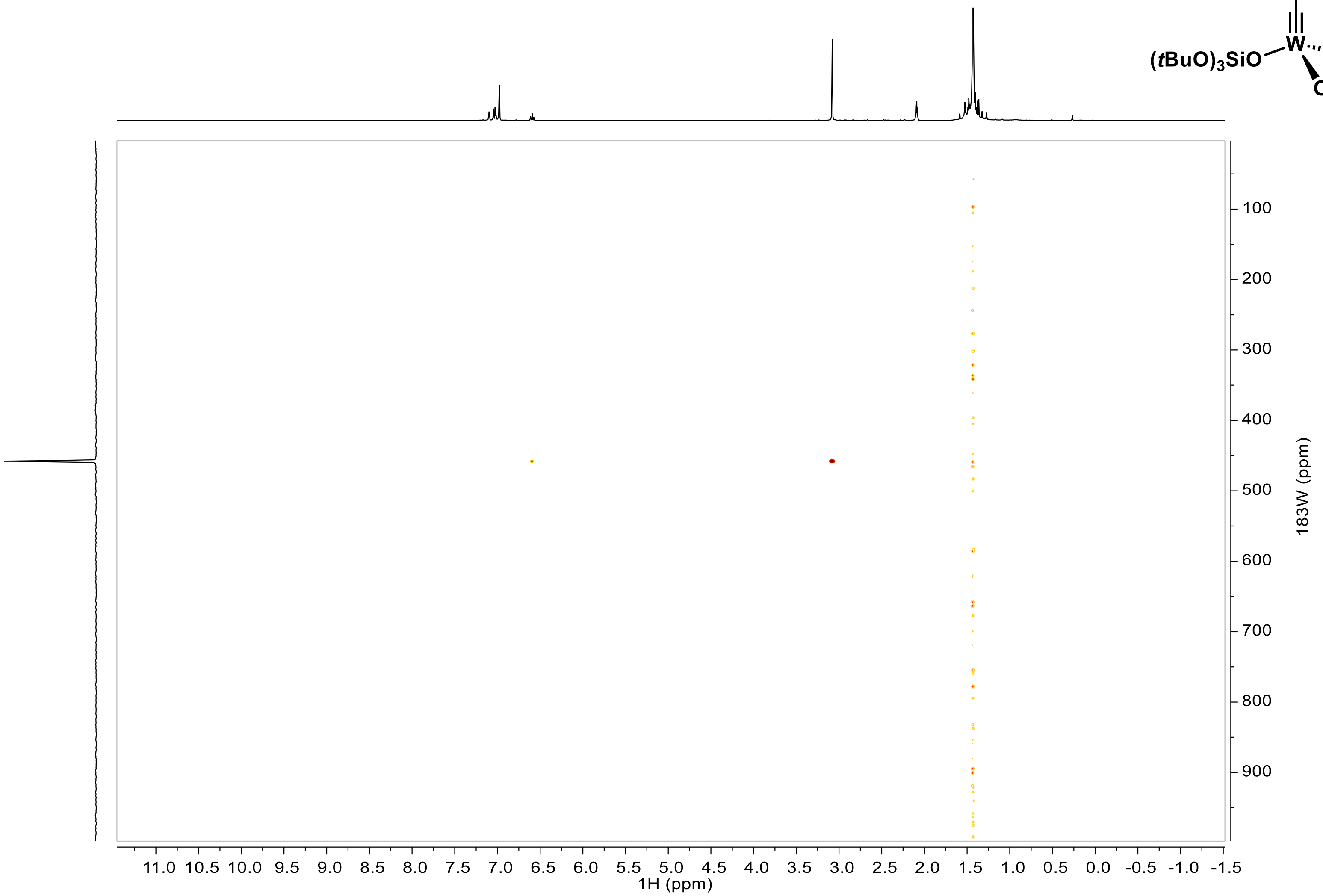

^{183}W NMR Projection created from 2D-HMBC Experiment of Complex 10b, 17 MHz, $C_6D_5CD_3$, 25ºC

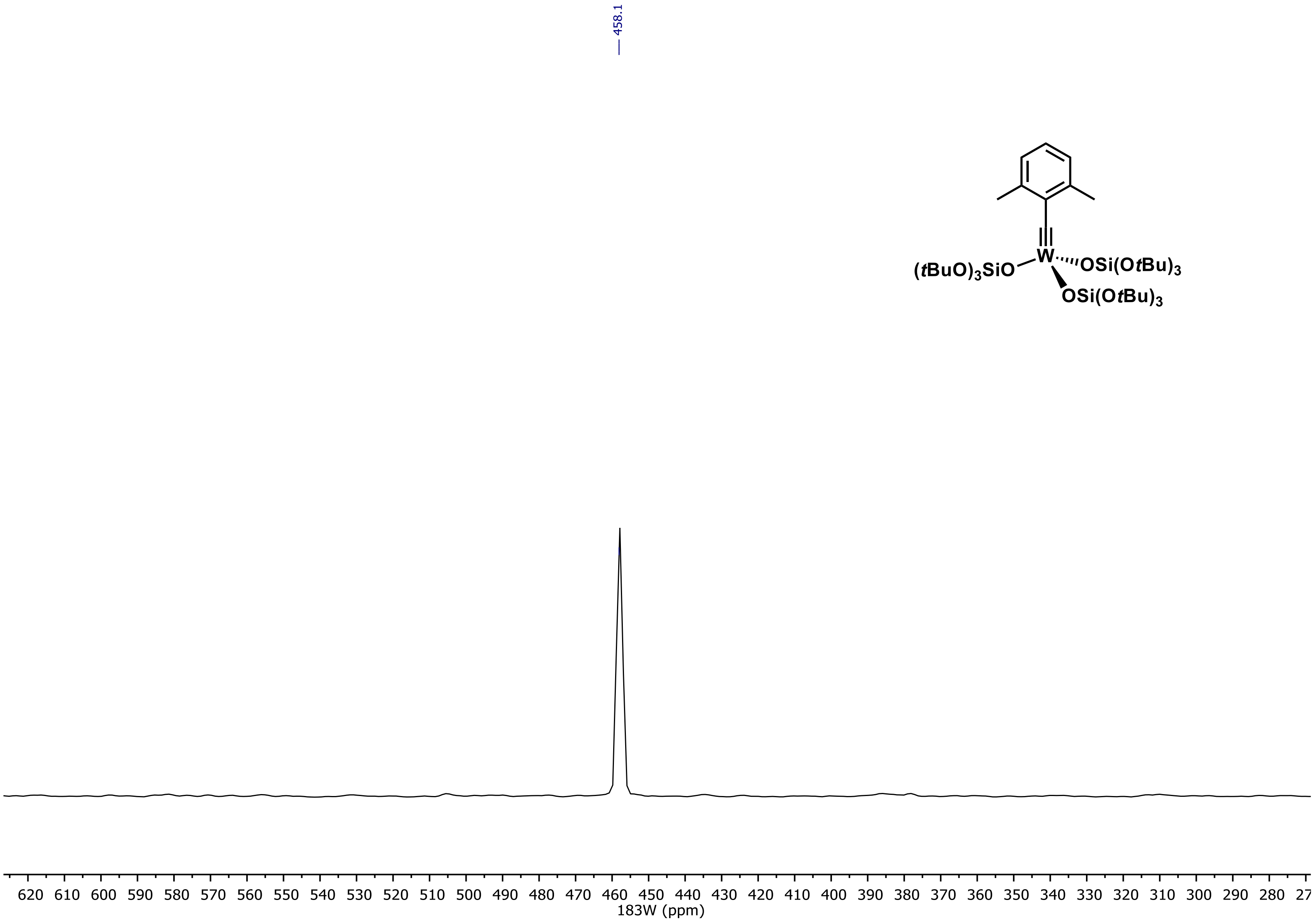

^{1}H NMR of Complex 23, 600 MHz, $C_6D_5CD_3$, 25ºC

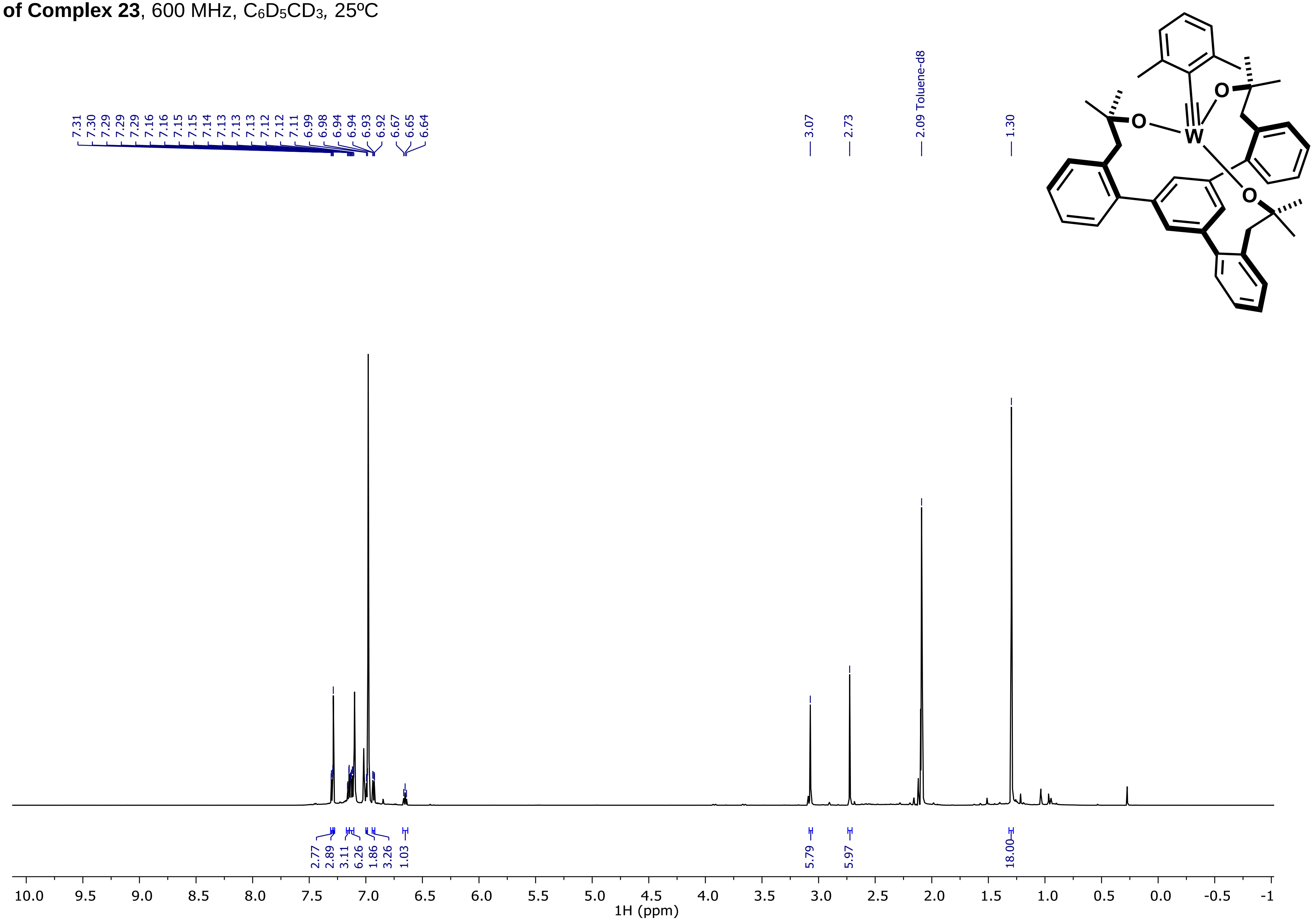

^{13}C NMR of Complex 23, 600 MHz, $C_6D_5CD_3$, 25ºC

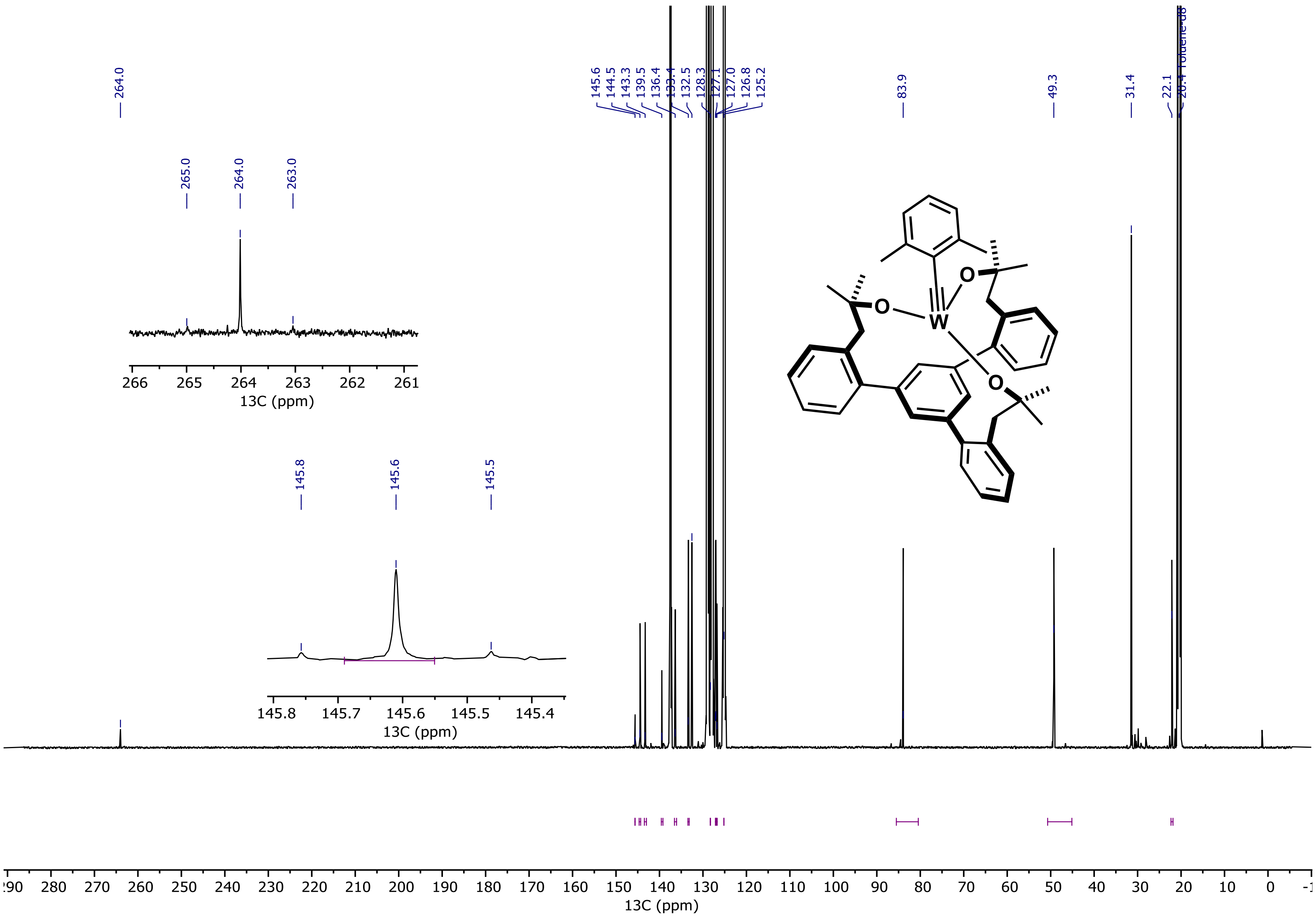

^{183}W NMR Projection created from 2D-HMBC Experiment of Complex 23, 17 MHz, $C_6D_5CD_3$, 25ºC

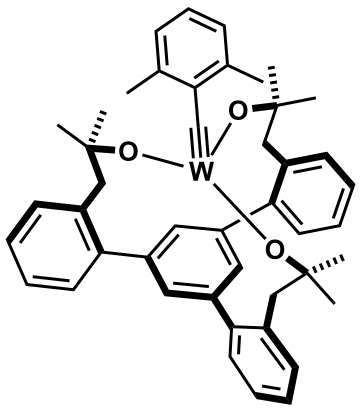

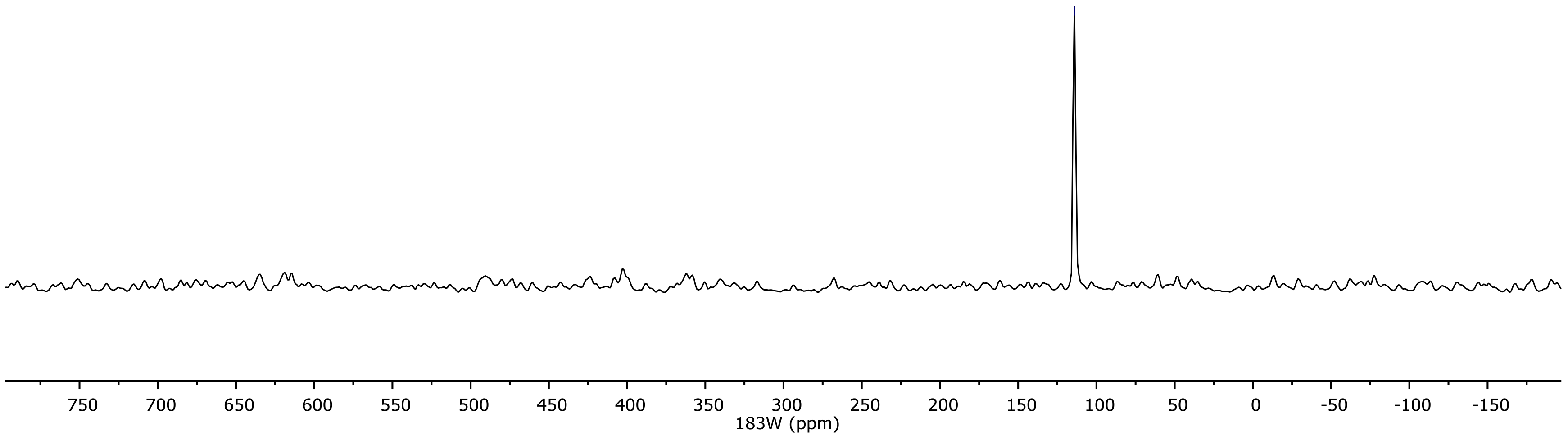

^{1}H NMR of 1,8-Dioxacyclotetradec-11-yne-2,7-dione (25), 400 MHz, $CDCl_3$, 25ºC

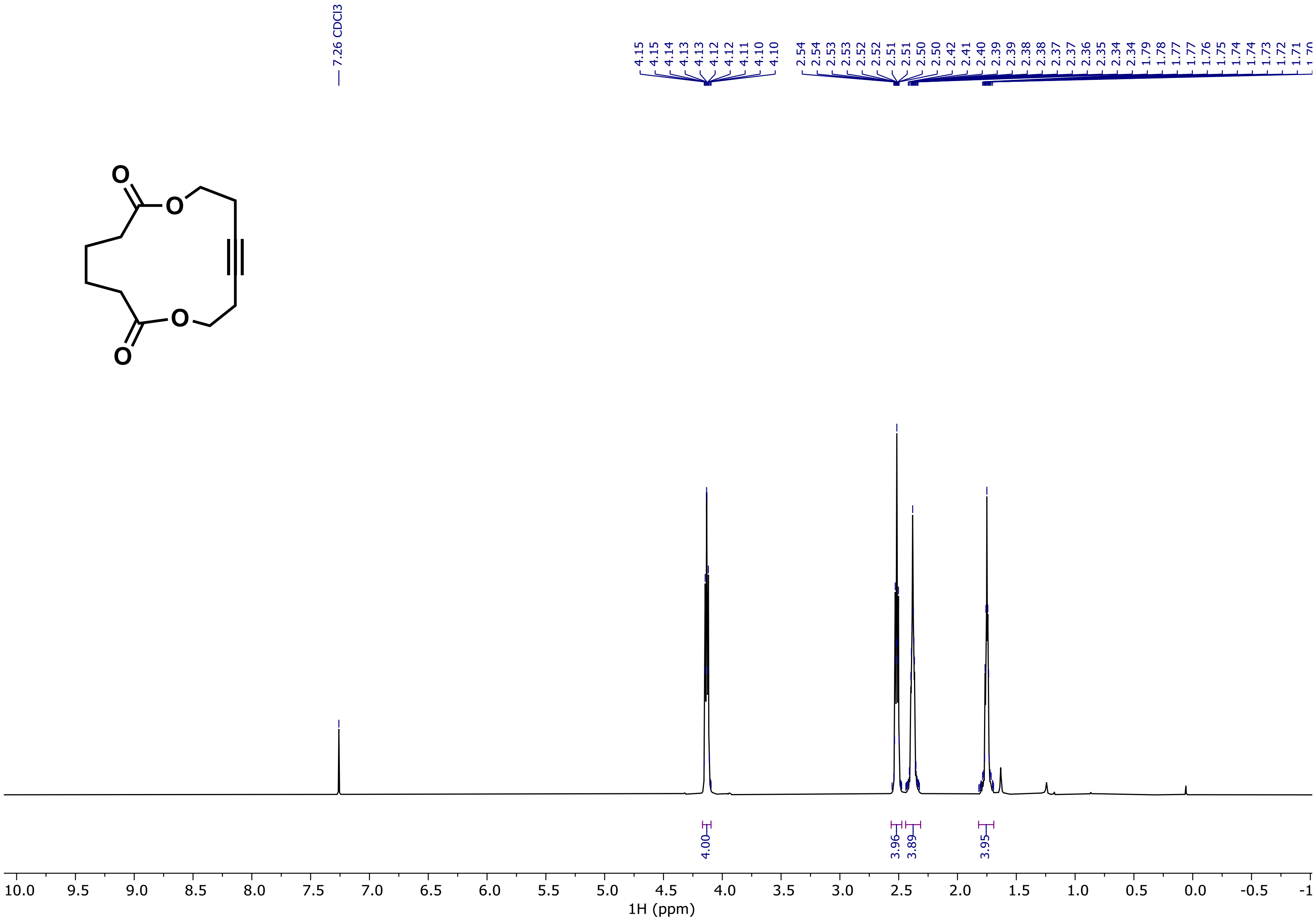

^{1}H NMR of compound 26, 400 MHz, $CDCl_3$, 25ºC

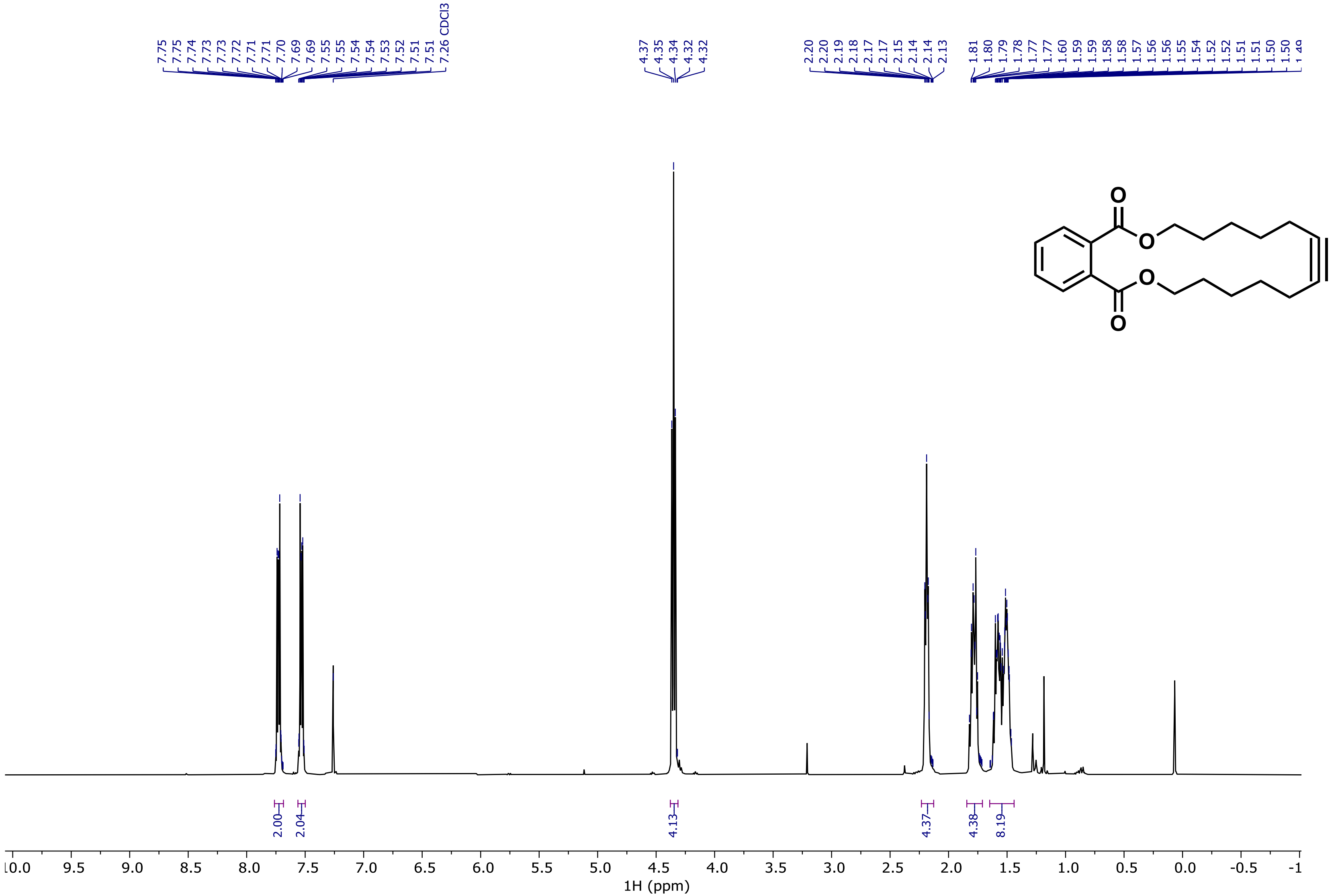

^{1}H NMR of compound 27, 400 MHz, $CDCl_3$, 25ºC

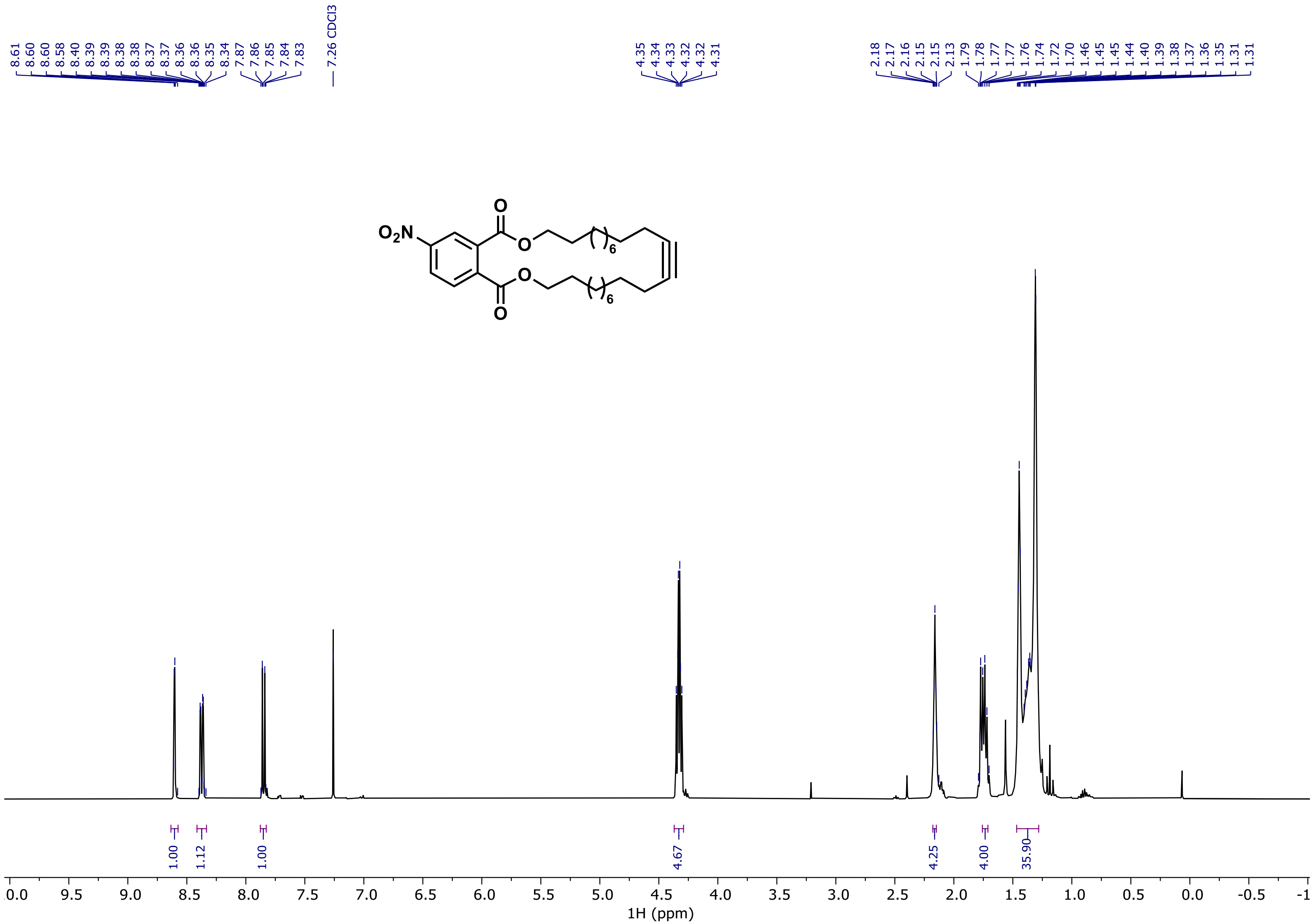

^{1}H NMR of compound 28, 400 MHz, $CDCl_3$, 25ºC

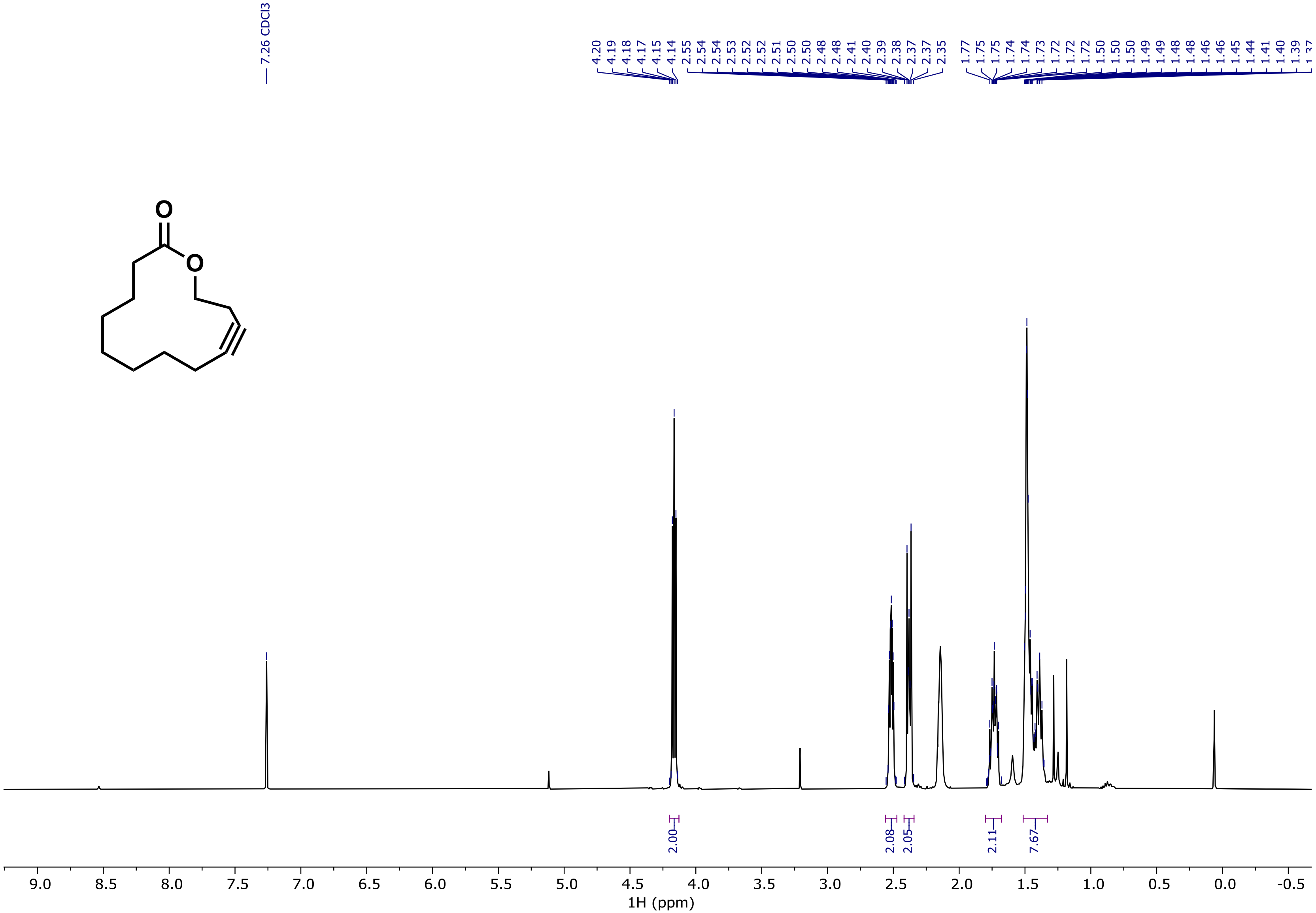

^{13}C NMR of compound 28, 400 MHz, $CDCl_3$, 25ºC

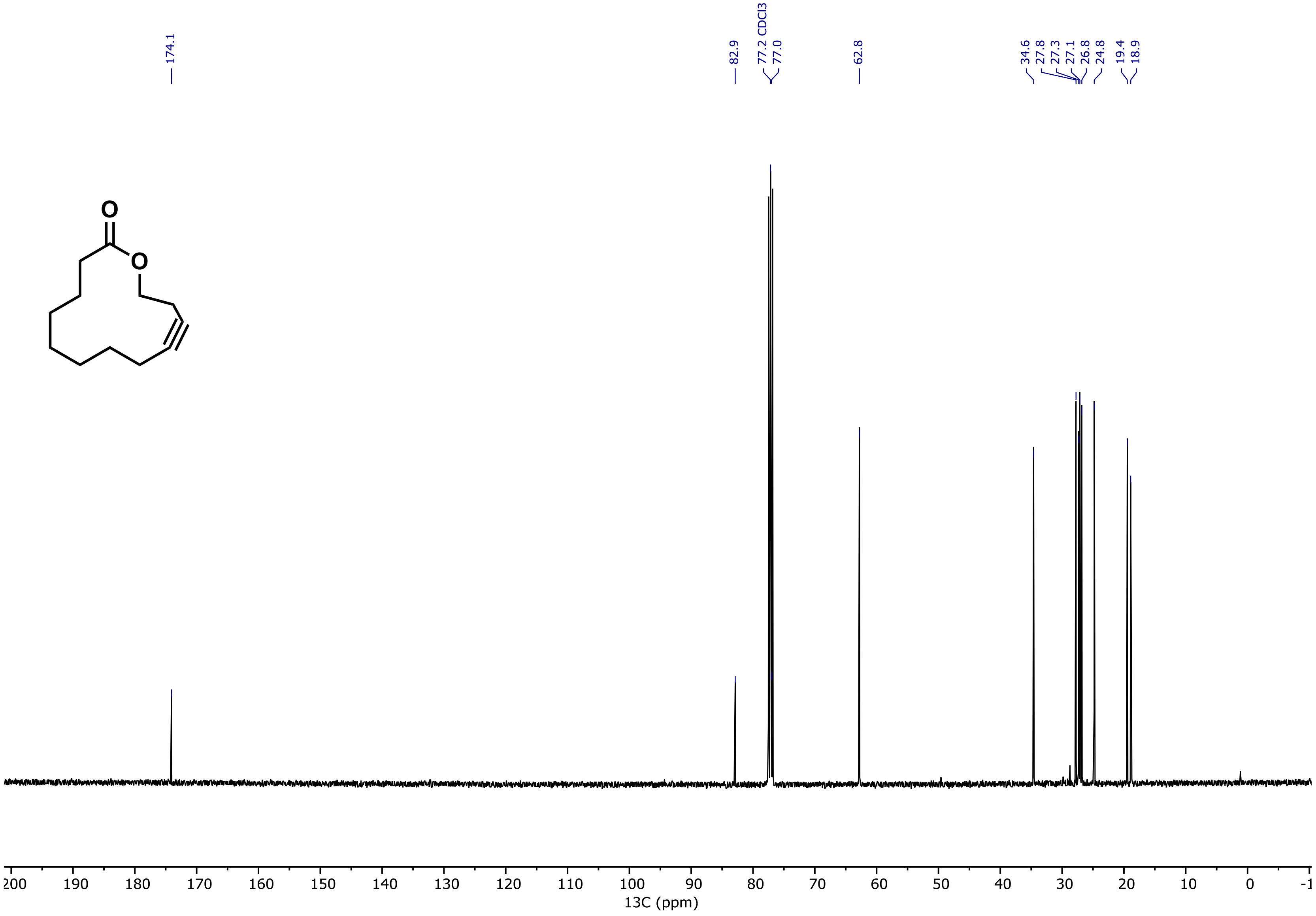

^{1}H NMR of compound 29, 400 MHz, $CDCl_3$, 25ºC

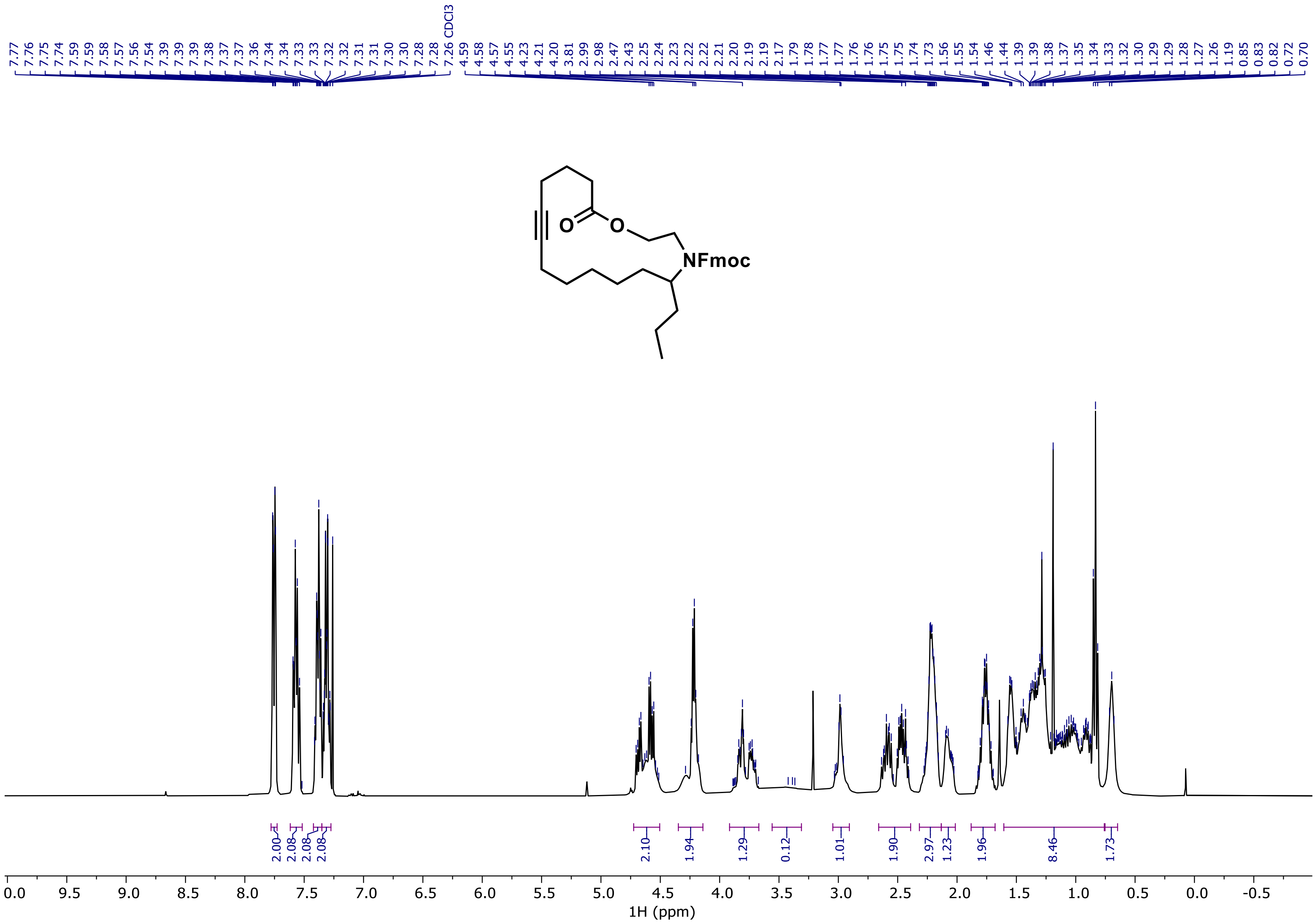

^{1}H NMR of compound 30, 400 MHz, $CDCl_3$, 25ºC

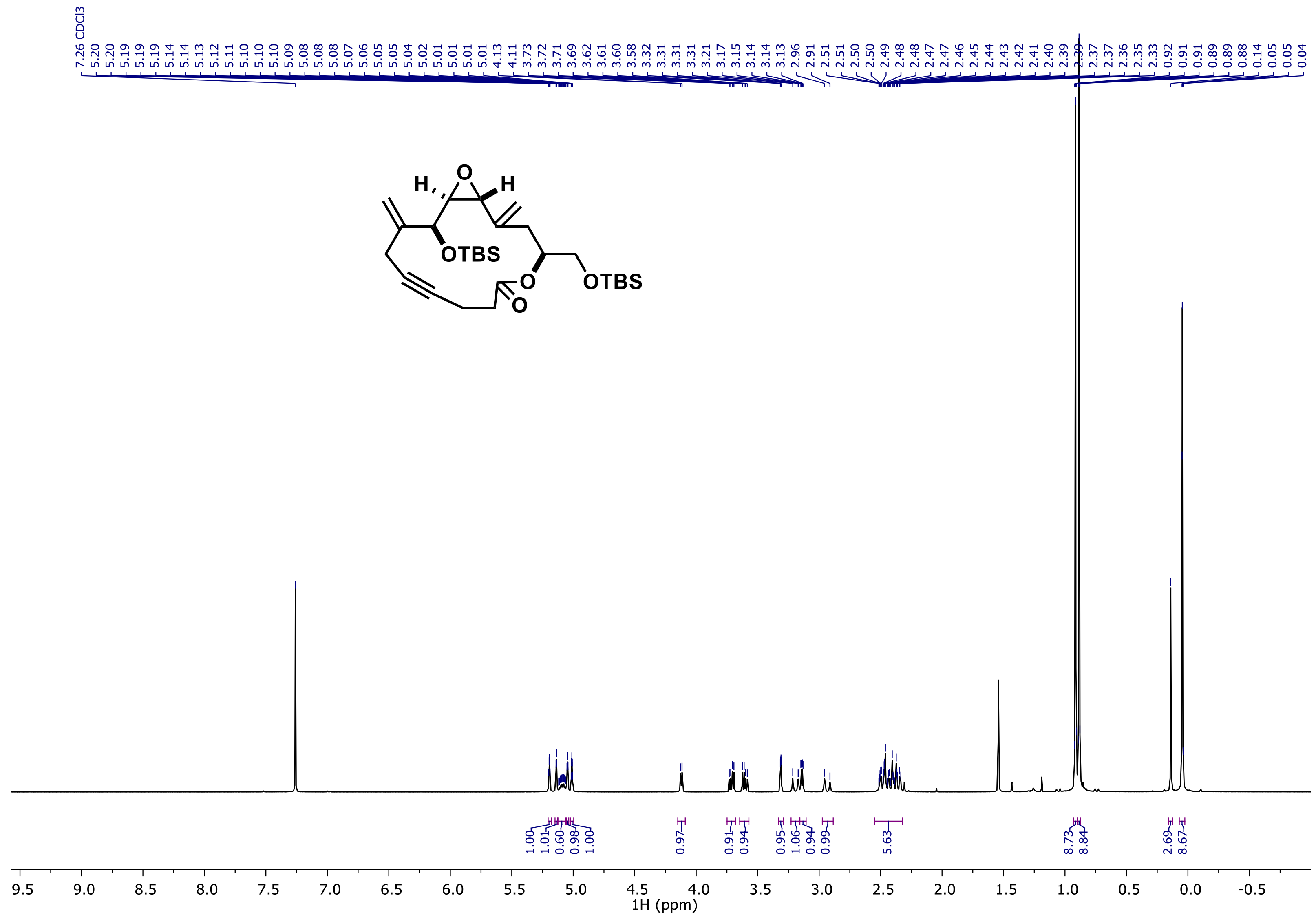

^{1}H NMR of compound 31, 400 MHz, $CDCl_3$, 25ºC

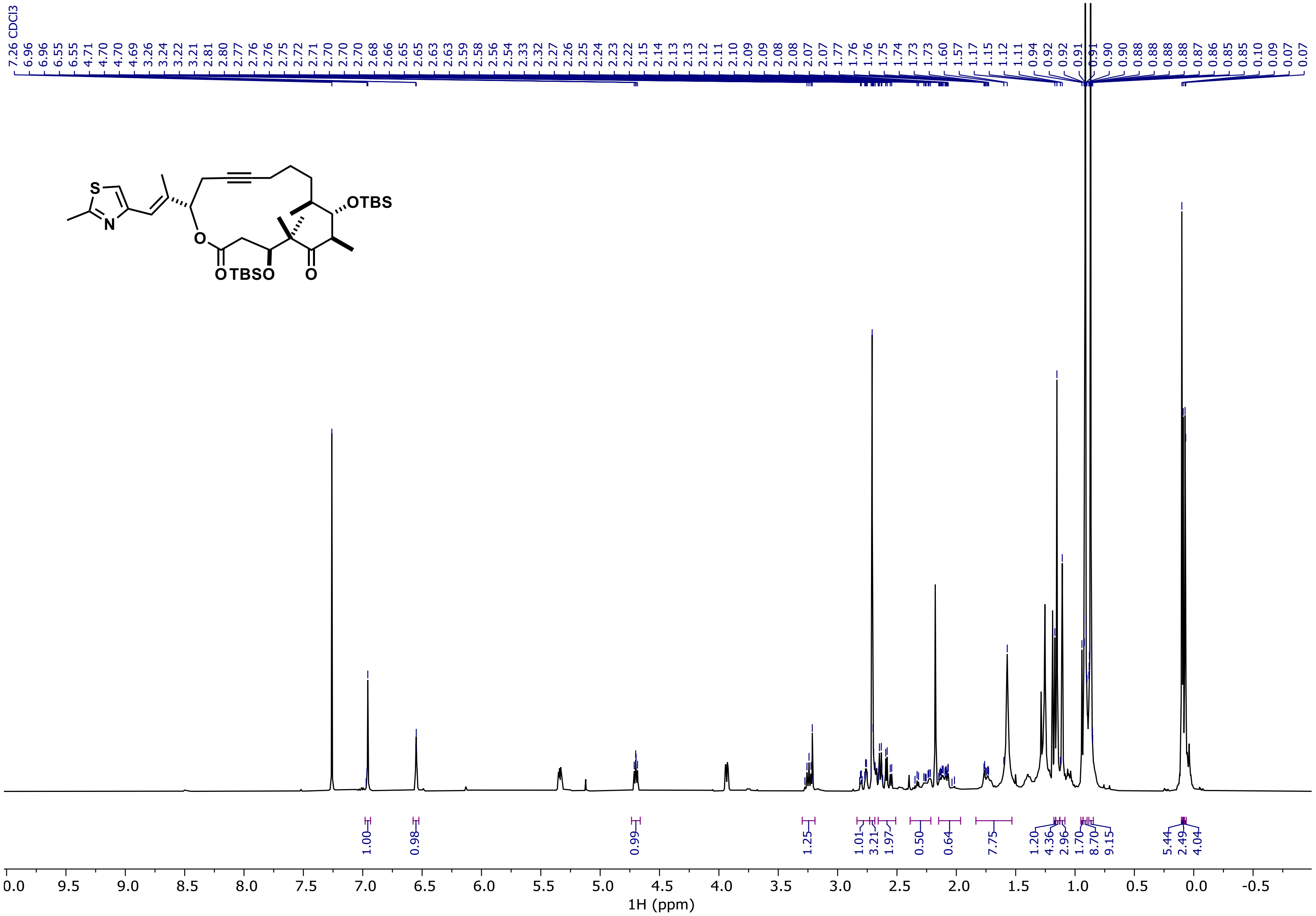

CRYSTALLOGRAPHIC INFORMATION

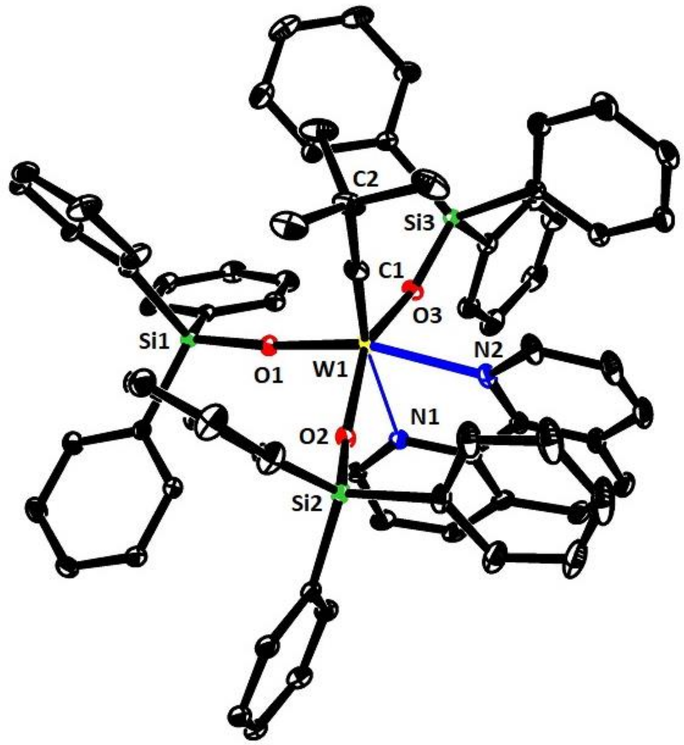

Figure S-1. Structure of [$(Ph_3SiO)_3W{\equiv}CCMe_3$]·(phen) [**8a**·(phen)] in the solid state

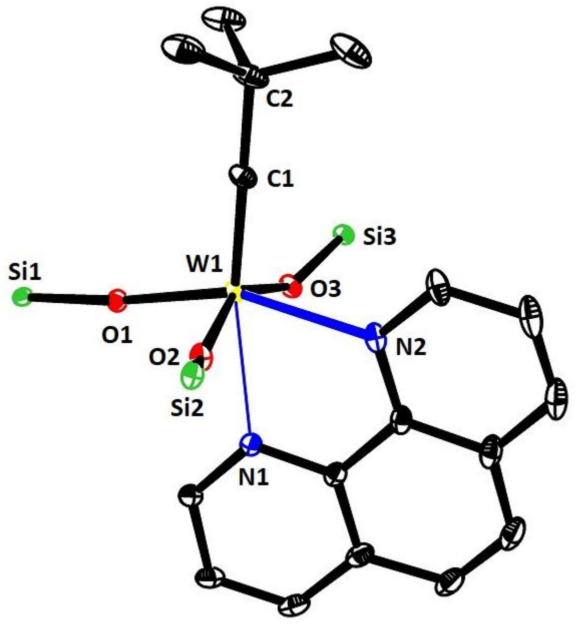

Figure S-2. Truncated structure showing the core of [$(Ph_3SiO)_3W{\equiv}CCMe_3$]·(phen) [**8a**·(phen)] in the solid state

X-ray Crystal Structure Analysis of Complex [8a·(phen)]: $C_{71}H_{62}N_2O_3Si_3W$, M_r = 1259.34 g mol^{-1}, orange prism, crystal size 0.30 x 0.26 x 0.09 mm, monoclinic, space group $P2_1/c$ [No. 14], a = 18.0841(14) Å, b = 19.4530(12) Å, c = 17.1683(10) Å, β = 102.996(7)°, V = 5884.9(7) Å^3, T = 100(2) K, Z = 4, D_{calc} = 1.421 g cm^{-3}, λ = 0.71073 Å, $\mu(Mo\text{-}K_\alpha)$ = 2.075 mm^{-1}, analytical absorption correction (T_{min} = 0.59, T_{max} = 0.84), Bruker-AXS Kappa Mach3 diffractometer with APEX-II detector and rotating anode X-ray source, 2.651 < θ < 33.055°, 156878 measured reflections, 22250 independent reflections, 19210 reflections with $I > 2\sigma(I)$, R_{int} = 0.0291. The structure was solved by dual space methods (*SHELXT*) and refined by full-matrix least-squares (*SHELXL*) against F^2 to R_1 = 0.022 [$I > 2\sigma(I)$], wR_2 = 0.048, 725 parameters, extinction coefficient = 0.00012(3), residual electron density 1.2 (0.63 Å from W1) / –1.3 (0.64 Å from W1) e · Å^{-3}. Some low-order reflections have been omitted from the final least-squares refinement. **CCDC-2013171**.

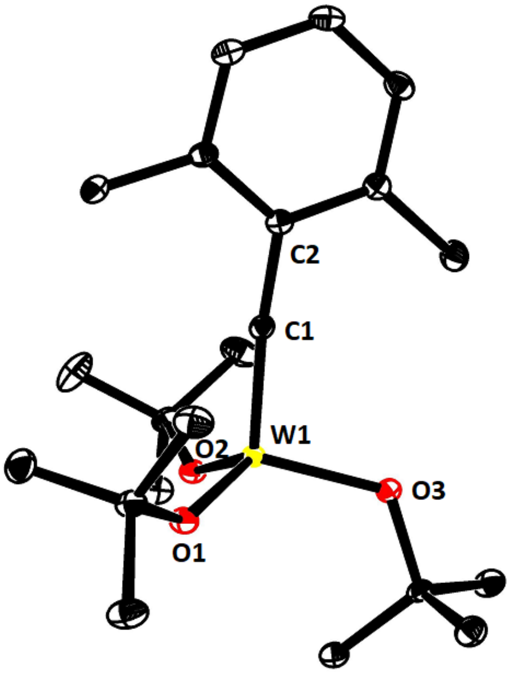

Figure S-3. Structure of [(*tert*-BuO)$_3$W≡CAr] (Ar = 2,6-dimethylphenyl) (**4b**) in the solid state

X-ray Crystal Structure Analysis of Complex 4b: $C_{21}H_{36}O_3W$, M_r = 520.35 g mol^{-1}, yellow plate, crystal size 0.17 x 0.16 x 0.08 mm, triclinic, space group $P\bar{1}$ [No. 2], a = 9.6983(9) Å, b = 10.9563(13) Å, c = 11.7039(13) Å, α = 98.969(5)°, β = 102.942(9)°, γ = 105.650(7)°, V = 1135.7(2) Å^3, T = 100(2) K, Z = 2, D_{calc} = 1.522 g cm^{-3}, λ = 0.71073 Å, $\mu(Mo\text{-}K_\alpha)$ = 5.100 mm^{-1}, analytical absorption correction (T_{min} = 0.44, T_{max} = 0.69), Bruker-AXS Kappa Mach3 diffractometer with APEX-II detector and IμS micro focus, 2.993 < θ < 27.500°, 24688 measured reflections, 5219 independent reflections, 5101 reflections with $I > 2\sigma(I)$, R_{int} = 0.0343. The structure was solved by dual space methods (*SHELXT*) and refined by full-matrix least-squares (*SHELXL*) against F^2 to R_1 = 0.014 [$I > 2\sigma(I)$], wR_2 = 0.037, 237 parameters, residual electron density 0.8 (0.90 Å from W1) / –1.2 (0.83 Å from W1) e · Å^{-3}. **CCDC- 2013167**.

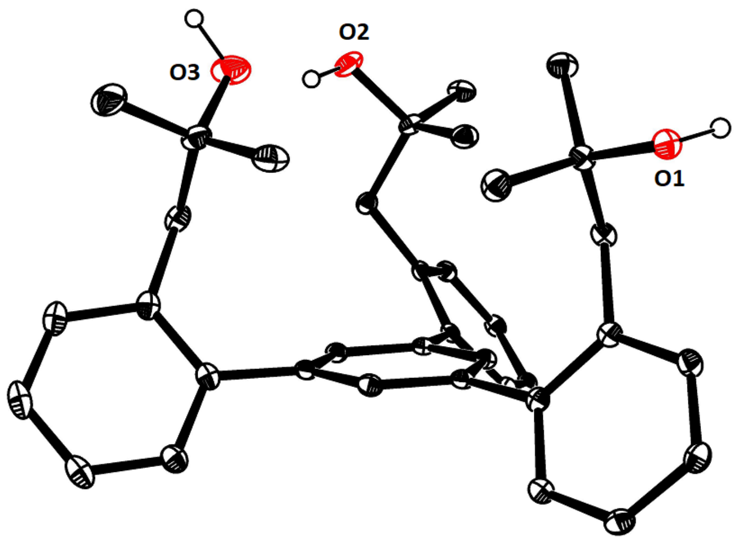

Figure S-4. Structure of the tripodal ligand **22** in the solid state; all hydrogen atoms, except the –OH protons, are omitted for clarity

X-ray Crystal Structure Analysis of Compound 22: $C_{72}H_{90}O_9$, M_r = 1099.43 g mol^{-1}, colorless plate, crystal size 0.4 x 0.23 x 0.12 mm, triclinic, space group $P\bar{1}$ [No. 2], a = 11.776(2) Å, b = 16.2767(16) Å, c = 17.1278(9) Å, α = 88.675(7)°, β = 72.901(7)°, γ = 89.130(10)°, V = 3137.0(6) Å^3, T = 100(2) K, Z = 2, D_{calc} = 1.164 g cm^{-3}, λ = 0.71073 Å, μ(*Mo-K*$_\alpha$) = 0.075 mm^{-1}, analytical absorption correction ($T_{\min}$ = 0.97, $T_{\max}$ = 0.99), Bruker-AXS Kappa Mach3 diffractometer with APEX-II detector and rotating anode X-ray source, 2.612 < θ < 36.065°, 152174 measured reflections, 29814 independent reflections, 21187 reflections with $I > 2\sigma(I)$, R_{int} = 0.0589. The structure was solved by dual space methods (*SHELXT*) and refined by full-matrix least-squares (*SHELXL*) against F^2 to R_1 = 0.045 [$I > 2\sigma(I)$], wR_2 = 0.128, 790 parameters, residual electron density 0.5 (0.67 Å from C48) / –0.3 (1.24 Å from C61) e · Å^{-3}. **CCDC-2013169**.

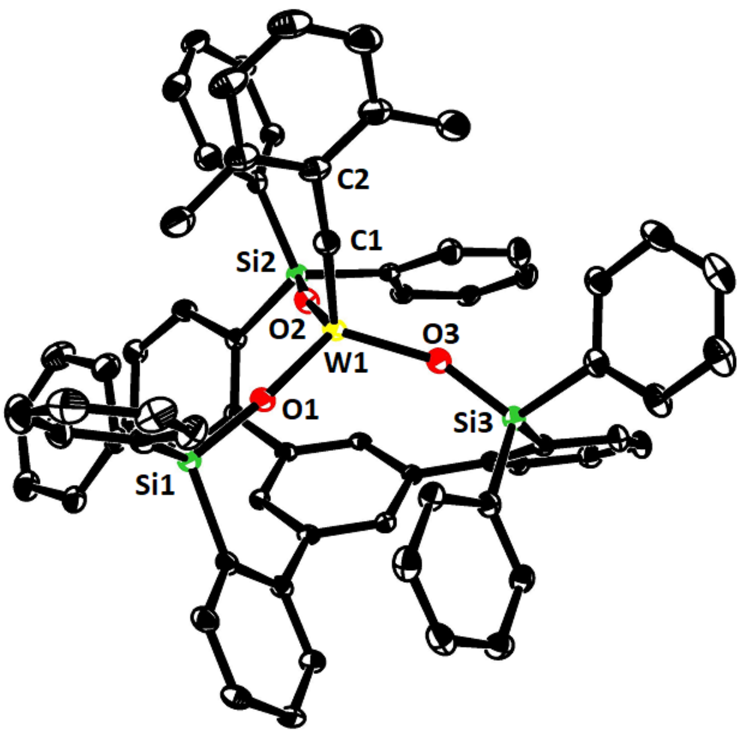

Figure S-5. Structure of the tungsten alkylidyne complex **16a** with a tripodal ligand framework in the solid state.

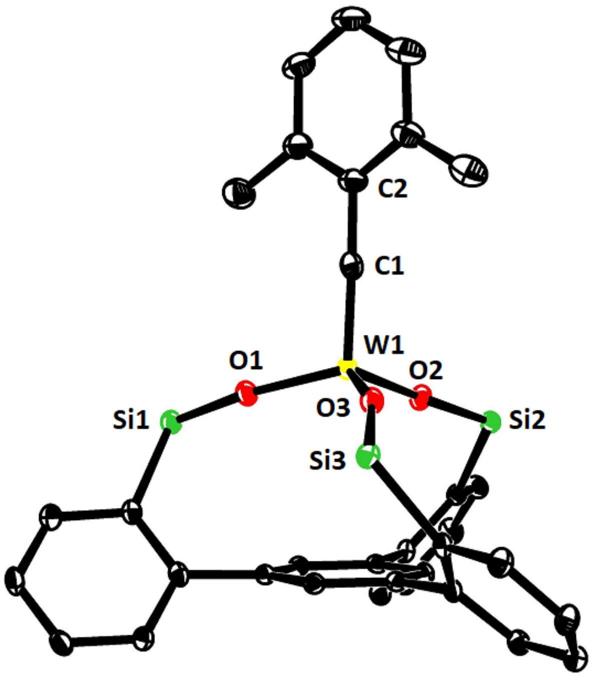

Figure S-6. Representation of the truncated structure of the tungsten alkylidyne complex **16a** with a tripodal ligand framework in the solid state, in which the lateral phenyl rings on the silicon atoms were removed for clarity

X-ray Crystal Structure Analysis of Complex 16a: $C_{69}H_{54}O_3Si_3W$, M_r = 1199.24 g mol^{-1}, yellow plate, crystal size 0.083 x 0.071 x 0.021 mm, triclinic, space group $P\bar{1}$ [No. 2], a = 13.9825(6) Å, b = 14.0803(6) Å, c = 16.5360(7) Å, α = 83.475(2)°, β = 72.892(2)°, γ = 61.767(2)°, V = 2740.1(2) Å^3, T = 100(2) K, Z = 1, D_{calc} = 1.454 g cm^{-3}, λ = 0.71073 Å, $\mu(Mo\text{-}K_\alpha)$ = 1.039 mm^{-1}, analytical absorption correction (T_{min} = 0.88, T_{max} = 0.97), Bruker-AXS Kappa Mach3 diffractometer with APEX-II detector and IµS micro focus, 1.289 < θ < 27.500°, 70950 measured reflections, 12597 independent reflections, 10259 reflections with $I > 2\sigma(I)$, R_{int} = 0.0645. The structure was solved by dual space methods (*SHELXT*) and refined by full-matrix least-squares (*SHELXL*) against F^2 to R_1 = 0.034 [$I > 2\sigma(I)$], wR_2 = 0.074, 687 parameters, residual electron density 2.6 (0.90 Å from W1) / –2.0 (0.83 Å from W1) e · Å^{-3}. **CCDC- 2013168**.

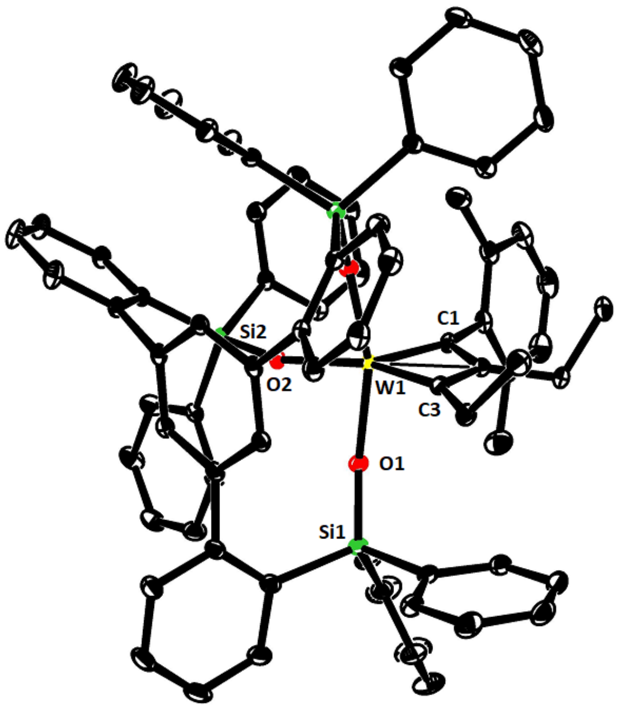

Figure S-7. Structure of the metallacyclobutadiene complex **17** with a tripodal ligand framework in the solid state

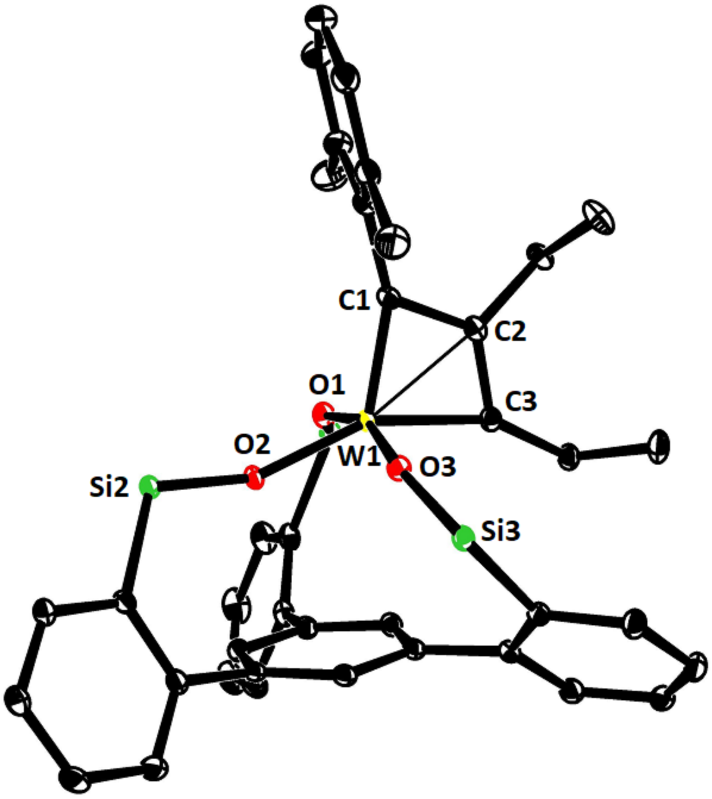

Figure S-8. Representation of the truncated structure of the metallacyclobutadiene complex **17** with a tripodal ligand framework in the solid state, in which the lateral phenyl rings on the silicon atoms were removed for clarity

X-ray Crystal Structure Analysis of Complex 17: $C_{79}H_{74}O_4Si_3W$, M_r = 1355.50 g mol^{-1}, red plate, crystal size 0.14 x 0.13 x 0.11 mm, triclinic, space group $P\bar{1}$ [No. 2], a = 12.785(3) Å, b = 13.8712(19) Å, c = 20.155(3) Å, α = 81.047(10)°, β = 86.292(17)°, γ = 87.448(14)°, V = 3521.2(10) Å^3, T = 100(2) K, Z = 2, D_{calc} = 1.278 g cm^{-3}, λ = 0.71073 Å, $\mu(Mo\text{-}K_\alpha)$ = 1.739 mm^{-1}, analytical absorption correction (T_{min} = 0.80, T_{max} = 0.85), Bruker-AXS Kappa Mach3 diffractometer with APEX-II detector and IµS micro focus, 2.711 < θ < 32.577°, 114388 measured reflections, 25571 independent reflections, 21228 reflections with $I > 2\sigma(I)$, R_{int} = 0.0492. The structure was solved by dual space methods (*SHELXT*) and refined by full-matrix least-squares (*SHELXL*) against F^2 to R_1 = 0.029 [$I > 2\sigma(I)$], wR_2 = 0.065, 790 parameters, a large solvent accessible void, containing disordered solvent, was masked in OLEX2, residual electron density 1.3 (1.32 Å from H14) / –1.6 (0.77 Å from W1) e · Å^{-3}. **CCDC-2014623**.

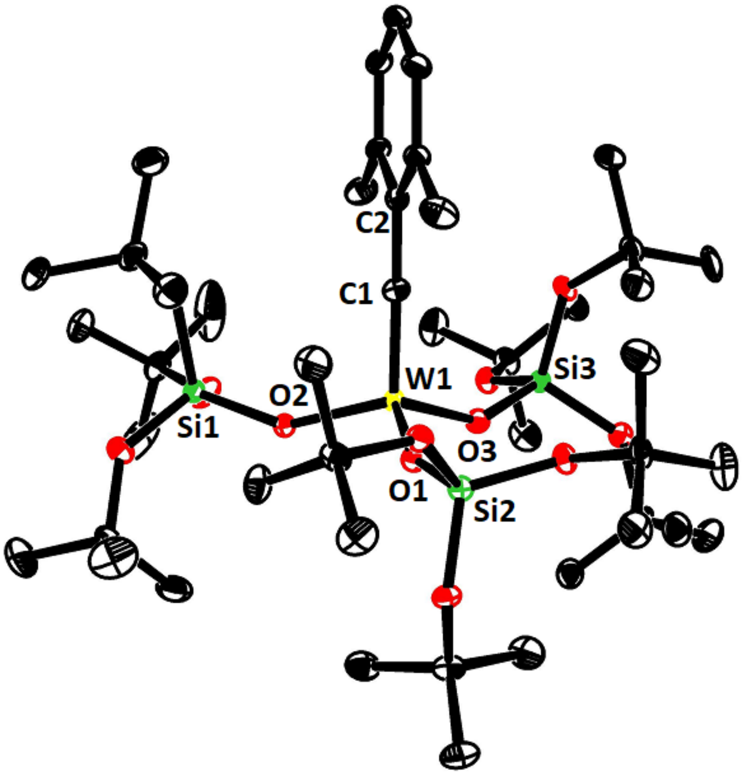

Figure S-9. Structure of [((*tert*-BuO)$_3$SiO)$_3$W≡CAr] (Ar = 2,6-dimethylphenyl) (**10b**) in the solid state

X-ray Crystal Structure Analysis of Complex 10b: $C_{45}H_{90}O_{12}Si_3W$, M_r = 1091.28 g mol^{-1}, yellow plate, crystal size 0.26 x 0.08 x 0.02 mm, orthorhombic, space group *P*ca2$_1$ [No. 29], a = 26.7662(17) Å, *b* = 9.6231(16) Å, *c* = 44.027(5) Å, *V* = 11340(2) Å^3, *T* = 100(2) K, *Z* = 8, D_{calc} = 1.278 g cm^{-3}, λ = 0.71073 Å, μ(*Mo*-K_α) = 2.151 mm^{-1}, analytical absorption correction (T_{min} = 0.02, T_{max} = 0.19), Bruker-AXS Kappa Mach3 diffractometer with APEX-II detector and rotating anode X-ray source, 2.607 < θ < 30.508°, 211778 measured reflections, 34108 independent reflections, 27986 reflections with $I > 2\sigma(I)$, R_{int} = 0.0830. The structure was solved by dual space methods (*SHELXT*) and refined by full-matrix least-squares (*SHELXL*) against F^2 to R_1 = 0.052 [$I > 2\sigma(I)$], wR_2 = 0.105, 1158 parameters, absolute structure parameter using Flack's method = 0.406(8), residual electron density 1.3 (0.78 Å from W1) / –2.7 (1.81Å from H40A) e · Å^{-3}. **CCDC-2013170**.

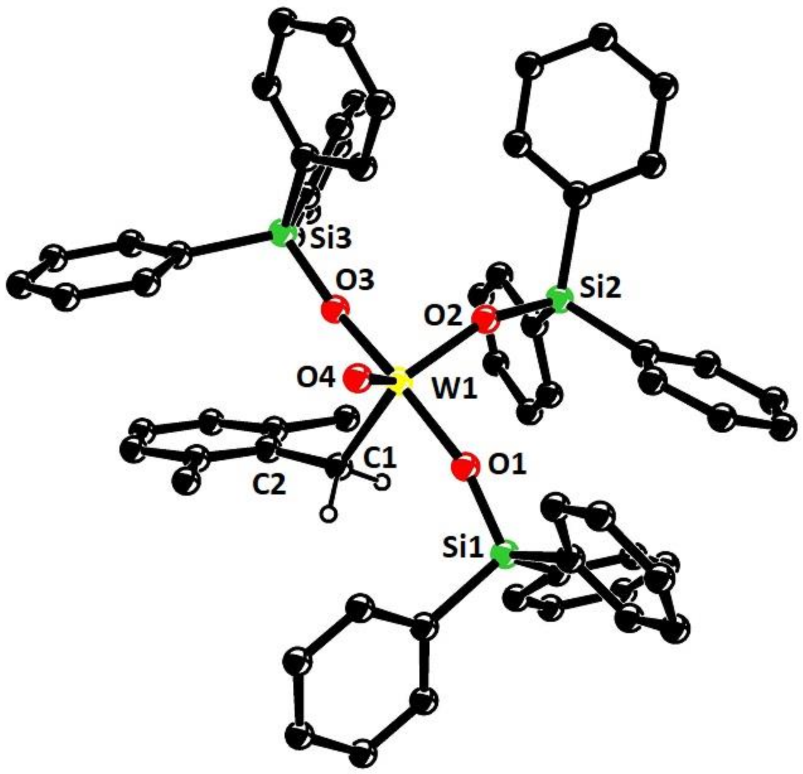

Figure S-10. Structure of the oxo benzyl complex **19** in the solid state

X-ray Crystal Structure Analysis of Complex 19: $C_{63}H_{56}O_4Si_3W$, M_r = 1145.19 g mol^{-1}, orange block, crystal size 0.16 x 0.10 x 0.10 mm, monoclinic, space group $P2_1/n$ [No. 14], a = 9.705(2) Å, b = 20.153(4) Å, c = 27.244(6) Å, β = 99.930(14)°, V = 5248.5(19) Å^3, T = 100(2) K, Z = 4, D_{calc} = 1.449 g cm^{-3}, λ = 0.71073 Å, μ(*Mo-K$_\alpha$*) = 2.318 mm^{-1}, analytical absorption correction (T_{min} = 0.77, T_{max} = 0.88), Bruker-AXS Kappa Mach3 diffractometer with APEX-II detector and rotating anode X-ray source, 2.838 < θ < 27.500°, 62702 measured reflections, 12028 independent reflections, 9770 reflections with $I > 2\sigma(I)$, R_{int} = 0.0551. The structure was solved by dual space methods (*SHELXT*) and refined by full-matrix least-squares (*SHELXL*) against F^2 to R_1 = 0.040 [$I > 2\sigma(I)$], wR_2 = 0.083, 650 parameters, absolute structure parameter using Flack's method = –0.03(2), residual electron density 2.1 (0.80 Å from W1) / –1.4 (0.66 Å from W1) e · Å^{-3}. **CCDC-2013172.**

GENERAL

Unless stated otherwise, all reactions were carried out under Ar in flame-dried glassware. The solvents used were purified by distillation over the drying agents indicated and were transferred under Ar: THF, Et_2O, 1,4-dioxane (Mg/anthracene), CH_2Cl_2, DME, MeCN (CaH_2), *n*-pentane, benzene, toluene (Na/K). Flash chromatography on silica gel (FC): Merck silica gel 60 (230–400 mesh).

All commercially available compounds (Fluka, Lancaster, Aldrich) were used as received, unless stated otherwise. Compounds **14**,[1] **15a**,[1] **S3**,[2] LiOSi(O*t*Bu)$_3$,[3] were prepared as described in the literature. The molecular sieves used in this investigation were dried for 24 h at 150 °C (sand bath) under vacuum prior to use and were stored and transferred under argon atmosphere.

IR: Spectrum One (Perkin-Elmer) spectrometer, wavenumbers ($\tilde{\nu}$) in cm^{-1}.
MS (EI): Finnigan MAT 8200 (70 eV), ESI-MS: ESQ3000 (Bruker), accurate mass determinations: Bruker APEX III FT-MS (7 T magnet) or Mat 95 (Finnigan). Elemental analysis: H. Kolbe, Mülheim/Ruhr.

NMR. Spectra were acquired on Bruker AvanceIII 300, 400, 500 MHz or an AvanceNeo 600 MHz NMR spectrometer in the solvents indicated; chemical shifts (δ) are given in ppm relative to TMS, coupling constants (*J*) in Hz. The solvent signals were used as references and the chemical shifts converted to the TMS scale ($CDCl_3$: $\delta_C \equiv 77.0$ ppm; residual $CHCl_3$ in $CDCl_3$: $\delta_H \equiv 7.26$ ppm; CD_2Cl_2: $\delta_C \equiv 53.8$ ppm; residual ^1H: $\delta_H \equiv 5.32$ ppm; [D_8]-toluene: $\delta_C \equiv 20.7$ ppm; residual $D_5C_6CD_2H$: $\delta_H = 2.09$ ppm). Chemical shifts were referenced indirectly to the ^1H chemical shift of the solvent.[4]

Diffusion coefficients were obtained from a double stimulated echo sequence with bipolar gradient pulses, convection compensation, longitudinal eddy current delay (LED) and three spoiler gradients (Bruker sequence: dstebpgp3s). The gradient pulse strength G was incremented from 2% to 98% of the maximum G_{max} with a squared gradient ramp in 60 steps. The diffusion time (Δ) used was 71 ms and the length of a gradient pulse gradient pulse (δ/2) of the encoding gradient was 1.3 ms. The maximum gradient strength G_{max} of the NMR probe (PA BBO 400S1 BBF-H-D-05 Z PLUS) was 53.5 $G\cdot cm^{-1}$. Diffusion coefficients were obtained by averaging three diffusion coefficients obtained from fitting the signal decay of three different resonance integrals to the Stejskal-Tanner equation (I) in the Bruker TOPSPIN T1T2 relaxation module.

$$I(G) = I_0 e^{-D(\gamma G\delta)^2(\Delta-\delta/3)} \quad \text{(I)}$$

Diffusion values were predicted using an EXCEL spreadsheet Stokes−Einstein Gierer-Wirtz Estimation (SEGWE) method.[5]

^{183}W NMR Spectroscopy. ^{183}W NMR shifts were extracted from the indirect dimension of a ^{1}H,^{183}W HMBC experiment. For the measurement, a standard Bruker BBFO probe (^{31}P-^{119}Ag) of a AVIIIHD 400 MHz NMR spectrometer could be tuned to the ^{183}W resonance frequency (16.67 MHz). The standard parameters for the experiment are shown in the following Table:

	F1	F2
**PULPROG**	hmbcgpndqf	
**Nuc1**	1H	183W
**TD**	2048	512
**Offset (ppm)**	5	500
**SW (ppm)**	12.98	997.4
**CNST13**	4	
**DS**	16	

	F1	F2
**NS**	4 (2)	
**GPNAM**	SMSQ10.100	
**GPZ1(%)**	70	
**GPZ2(%)**	30	
**GPZ3(%)**	44.17	
**NUS**	25%	
**Expt**	16 min 16 sec (8 min 8 s)	

The π/2 pulse was calibrated for a solution of Na_2WO_4 in D_2O (1 M) and had a typical length of 35 µs with a pulse power of 85 W. Data points in the indirect dimension (F1) were sparsely sampled with 25% NUS to reduce the measurement time. A second HMBC (10% NUS) with the same offset O2, but different SW in the indirect dimension was acquired to make sure that the observed peak is not folded. Raw data were Fourier-transformed with zero-filling and linear-prediction in the indirect dimension giving to a total matrix size of 1024 x 1024 datapoints. Unless denoted otherwise 1D ^{183}W spectra shown in the manuscript were generated from the 2D spectrum as a sum of all slices that contained a ^{183}W signal. The 1D ^{183}W spectrum of compound **4b** was acquired with a standard ineptrd sequence optimized for the same long range coupling (4 Hz) as the HMBC spectra. Data from AvanceNEO 600 MHz NMR spectrometer was acquired Bruker BBO CryoProbe, which significantly reduced the measurement time of most of the spectra, especially the 1D ^{13}C spectrum. A good SNR in the ^{13}C spectra was crucial to extract the ^{183}W-^{13}C coupling constants of the complexes.

EXPERIMENTAL DATA

Preparation of the Ligands

***CAUTION*: This and other experiments described below require pyrophoric materials (*tert*-butyllithium) which must be handled and disposed with great care! Likewise, quenching of the reactions can be dangerous. Especially when carried out on large scale, the use of either isopropanol in heptanes (2 M) or of dry CO_2 as quenching agent is recommended.**

(5'-(2-(Diethylsilyl)phenyl)-[1,1':3',1''-terphenyl]-2,2''-diyl)bis(diethylsilane) (S1)

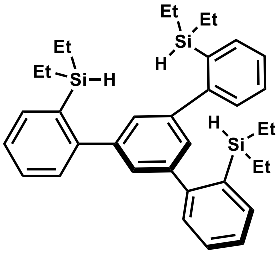

A two-necked, round-bottomed flask was equipped with a magnetic stir bar and a gas inlet connected to an argon-vacuum manifold. The flame-dried flask was filled with argon and charged with 1,3,4-tris-2'-bromophenylbenzene (**14**)[1] (2.00 g, 3.68 mmol) and Et_2O (77 mL). The resulting mixture was cooled to −125 °C. A solution of *tert*-butyllithium (14.2 mL, 22.6 mmol, 1.6 M in *n*-pentane) was added drophwise and the mixture was allowed to warm to ambient temperature. After stirring for 1 h stirring, the mixture was cooled to −125 °C and diethylsilane (2.86 mL, 22.1 mmol) was added dropwise. The mixture was then warmed to ambient temperature and stirring was continued overnight. The reaction was carefully quenched by adding water and the resulting mixture was transferred into a separation funnel. The organic phase was separated and the aqueous solution was extracted with ethyl acetate (3 x 50 mL). The combined organic layers were dried over $MgSO_4$, filtered and concentrated in vacuo. The residue was purified by flash chromatography on silica gel (hexanes/ethyl acetate, 10:1) to give the title compound as a colorless solid (1.9 g, 91%). ^{1}H NMR (400 MHz, CD_3Cl): δ = 7.58 – 7.54 (m, 3H), 7.42 – 7.36 (m, 3H), 7.35 – 7.28 (m, 6H), 7.22 (s, 3H), 4.13 (p, *J* = 3.4 Hz, 3H), 0.84 (t, *J* = 7.8 Hz, 18H), 0.60 (m, 12H). ^{13}C NMR (101 MHz, $CDCl_3$): δ = 149.4, 143.1, 136.0, 134.2, 129.6, 129.1, 128.9, 126.4, 8.5, 4.2. IR (film): ṽ 3051, 2952, 2872, 2102, 1583, 1558, 1460, 1409, 1378, 1260, 1230, 1124, 1098, 1063, 1006, 970, 891, 873, 736, 686, 640, 623, 607, 527, 458 cm^{-1}. HRMS-APPI (m/z): calcd. for $C_{36}H_{48}Si_3$ $[M]^{\cdot+}$, 564.30584; found, 564.30642.

Ligand 15b

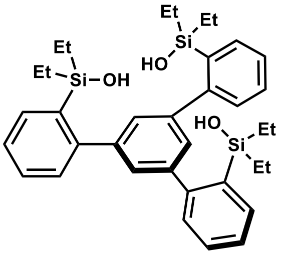

A one-neck round bottomed flask equipped with a stir bar was charged with silane **S1** (222 mg, 0.393 mmol) and CH_2Cl_2 (5 mL). The resulting mixture was cooled to 0 °C. *m*-Chloroperbenzoic acid (291 mg, 1.30 mmol, 77% *w/w*) was added portionwise and the reaction mixture was allowed to warm to ambient temperature. After 4 h, the mixture was carefully transferred to a separation funnel, diluted with CH_2Cl_2 (10 mL) and washed with sat. $NaHCO_3$ (4 x 15 mL) and brine (3 x 10 mL). The organic phase was then dried over $MgSO_4$, filtered and concentrated *in vacuo* to give ligand **15b** as a colorless solid (240 mg, 99%). ^{1}H NMR (400 MHz, $CDCl_3$): δ = 7.51 – 7.47 (m, 3H), 7.43 – 7.33 (m, 6H), 7.33 – 7.30 (m, 3H), 7.24 (s, 3H), 0.90 – 0.85 (m, 18H), 0.75 (m, 12H). ^{13}C NMR (101 MHz, $CDCl_3$): δ = 149.0, 144.1, 135.9, 134.6, 129.9, 128.9, 127.6, 126.4, 8.2, 6.9. ^{29}Si NMR (119 MHz, $CDCl_3$): δ = 7.7. IR (film): ṽ 3318, 3051, 2955, 2912, 2875, 1583, 1558, 1461, 1410, 1378, 1260, 1235, 1162, 1124, 1090, 1065, 1005, 959, 908, 888, 822, 760, 711, 615, 530, 512, 465 cm^{-1}. HRMS-ESI (m/z): calcd. for $C_{36}H_{47}O_3Si_3$ $[M-H]^-$, 611.28386; found, 611.28383.

(5'-(2-(Di-isobutylsilyl)phenyl)-[1,1':3',1''-terphenyl]-2,2''-diyl)bis(diisobutylsilane) (S2)

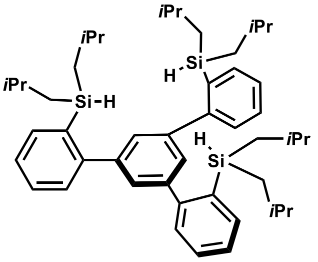

A two-necked, round-bottomed flask was equipped with a magnetic stir bar and a gas inlet connected to an argon-vacuum manifold. The flame-dried flask was filled with argon and charged with 1,3,4-tris-2'-bromophenylbenzene (**14**) (1.50 g, 2.76 mmol)[1] and Et_2O (58 mL). The resulting mixture was cooled to −125 °C. A solution of *tert*-butyllithium (8.94 mL, 17.0 mmol, 1.9 M in *n*-pentane) was added dropwise and the mixture was allowed to warm to ambient temperature. After stirring for 1 h at ambient temperature, the mixture was cooled to −125 °C and di-*iso*-butylchlorosilane (3.07 mL, 16.6 mmol) was added dropwise. The mixture was warmed to ambient temperature and stirring was continued overnight. The reaction was carefully quenched by adding water and the resulting mixture was transferred into a separation funnel. The organic phase was separated and the aqueous solution extracted with ethyl acetate (3 x 50 mL). The combined organic layers were dried over $MgSO_4$, filtered and concentrated in vacuo. The residue was purified by flash chromatography on silica gel (hexanes/*t*-butyl methyl ether, 99:1) and the product dried in high vacuum (70°C, 10^{-3} mbar, 3 h) to give silane **S2** as a colorless oil (1.56 g, 77%). ^{1}H NMR (600 MHz, CD_3Cl): δ = 7.64 (ddd, *J* = 7.4, 1.4, 0.6 Hz, 3H), 7.40 – 7.36 (m, 6H), 7.35 – 7.31 (m, 3H), 7.27 (s, 3H), 4.35 – 4.28 (m, 3H), 1.68 – 1.57 (m, 6H), 0.82 – 0.75 (m, 36H), 0.68 – 0.56 (m, 12H). ^{13}C NMR (151 MHz, $CDCl_3$): δ = 149.3, 143.0, 136.3, 135.1, 129.6, 129.1, 129.0, 126.4, 26.1, 25.7, 25.5, 24.3. ^{29}Si NMR (119 MHz, $CDCl_3$): δ = −14.4. IR (film): ṽ 3053, 2952, 2895, 2867, 2826, 2113, 1584, 1558, 1463, 1408, 1382, 1364, 1328, 1261, 1203, 1163, 1124, 1085, 1034, 950, 891, 849, 759, 740, 723, 637, 623, 527, 461, 417 cm^{-1}. HRMS-APPI (m/z): calcd. for $C_{48}H_{72}Si_3$ [M]$^+$, 732.49364; found, 732.49398.

Ligand 15c

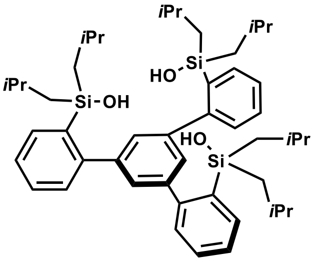

A two-neck round bottomed flask equipped with a stir bar was charged with silane **S2** (563 mg, 0.768 mmol) and CH_2Cl_2 (10 mL). The resulting mixture was cooled to 0 °C. *m*-Chloroperbenzoic acid (568 mg, 2.53 mmol, 77% w/w) was added in portions and the reaction mixture was allowed to warm to ambient temperature. After 4 h, the mixture was carefully transferred to a separation funnel, diluted with CH_2Cl_2 (10 mL) and washed with sat. $NaHCO_3$ (4 x 15 mL) and brine (3 x 10 mL). The organic phase was then dried over $MgSO_4$, filtered and concentrated *in vacuo* to give ligand **15c** as a colorless solid (584 mg, 97%). ^{1}H NMR (600 MHz, $CDCl_3$): δ = 7.54 – 7.51 (m, 3H), 7.40 – 7.36 (m, 3H), 7.34 – 7.32 (m, 6H), 7.27 (s, 3H), 1.75 (hept, *J* = 6.6 Hz, 6H), 0.80 (m, 36H), 0.78 – 0.69 (m, 12H). ^{13}C NMR (151 MHz, $CDCl_3$): δ = 148.5, 144.1, 137.4, 135.0, 129.7, 128.9, 127.9, 126.2, 28.5, 26.6, 26.3, 24.3. ^{29}Si NMR (119 MHz, $CDCl_3$): δ = 4.5. IR (film): ṽ 3449, 2951, 2924, 2894, 2866, 1584, 1463, 1435, 1409, 1381, 1364, 1328, 1219, 1163, 1123, 1088, 1064, 1033, 951, 908, 889, 830, 814, 759, 733, 667, 643, 622, 528, 487, 468 cm^{-1}. HRMS-ESI (m/z): calcd. for $C_{48}H_{72}O_3Si_3Na$ [M+Na]$^+$, 803.46815; found, 803.46891.

Ligand 22

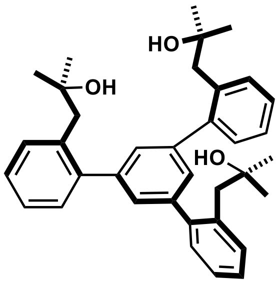

A two-necked, round-bottomed flask was equipped with a magnetic stir bar and a gas inlet connected to an argon-vacuum manifold. The flame-dried flask was filled with argon and charged with compound **14** (2.64 g, 4.86 mmol).[1] Et_2O (102 mL) was added and the resulting suspension was cooled to –125°C. A solution of *tert*-butyllithium (18.7 mL, 29.9 mmol, 1.6 M in *n*-pentane) was added over 10 min and the mixture was allowed to warm to ambient temperature once the addition was complete. After stirring for another 1 h, the heterogeneous dark brown mixture was again cooled to –125°C. 1,1-Dimethyloxirane (1.76 mL, 19.4 mmol) was added dropwise and the resulting mixture was allowed to warm to ambient temperature. After 8 h, the reaction was carefully quenched by adding water and the resulting mixture was transferred into a separation funnel. The organic phase was separated and the aqueous solution was extracted with ethyl acetate (4 x 50 mL). The combined organic layers were dried over $MgSO_4$, filtered and concentrated *in vacuo*. Hexanes (50 mL) was added to precipitate ligand **22** in the form of a white solid material (1.55 g, 61%). Colorless crystals suitable for single-crystal X-ray diffraction were grown from a concentrated CH_2Cl_2 solution (5 mL) layered with *n*-pentane (5 mL). ^{1}H NMR (400 MHz, $CDCl_3$): δ = 7.4 – 7.3 (m, 15H), 3.0 (s, 6H), 1.1 (s, 18H). ^{13}C NMR (101 MHz, $CDCl_3$): δ = 142.7, 142.6, 135.6, 131.8, 130.8, 129.9, 127.4, 126.8, 72.2, 45.9, 29.5. IR (film): $\tilde{\nu}$ 3404, 2971, 2929, 1591, 1488, 1465, 1412, 1364, 1302, 1202, 1138, 1083, 971, 901, 850, 757, 725, 639, 624, 613, 543, 513, 461 cm^{-1}. HRMS-ESI (m/z): calcd. for $C_{36}H_{41}O_3^-$ [M−H]$^-$, 521.30502; found, 521.30639.

Preparation of the Complexes

Complex S4

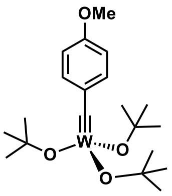

A 25 mL Schlenk flask was equipped with a magnetic stir bar and was flame dried under vacuum. The flask was filled with argon and W(≡CAr)Br_3(dme) (Ar = 4-methoxyphenyl) (**S3**)[2] (503 mg, 0.795 mmol) was dissolved in THF (9 mL). Then a solution of NaO*t*Bu (232 mg, 2.37 mmol) in THF (2 mL) was added dropwise at 23°C to the stirred solution. Stirring was continued for 14 h at ambient temperature before the solvent was removed *in vacuo* to obtain a dark brown solid. A second, flame dried 50 mL Schlenk flask was equipped with a magnetic stir bar and a Celite® (2 cm) packed argon frit. The dark brown solid was suspended in *n*-pentane (4 x 5 mL) and was filtered through the Celite® pad. The resulting filtrate was concentrated and the residue dried under vacuum (10^{-3} mbar) to give complex **S4** as a brown solid (376 mg, 91%) free of any residual THF. ^{1}H NMR (400 MHz, C_6D_6): δ = 7.31 – 7.25 (m, 2H), 6.87 – 6.72 (m, 2H), 3.31 (d, *J* = 1.0 Hz, 3H), 1.52 (d, *J* = 0.8 Hz, 27H). ^{13}C NMR (101 MHz, C_6D_6): δ = 257.2, 158.4, 142.2, 133.5, 113.2, 81.0, 54.8, 32.8. IR (film): $\tilde{\nu}$ 2969, 1580, 1505, 1468, 1359, 1277, 1239, 1166, 1042, 935, 826, 788, 590, 547, 481 cm^{-1}. HRMS-EI (m/z): calcd. for $C_{20}H_{34}WO_4^+$ [M]$^+$, 522.19670; found, 522.19707. Elemental analysis (%) calcd for $C_{20}H_{34}WO_4$: C 45.99, H 6.56; found: C 45.77, H 6.31.

Complex $[NMe_4]W(CO)_5(COAr)$ (Ar = 2,6-dimethlyphenyl) (S5)[6]

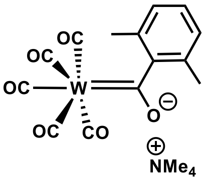

A two-neck round-bottom flask is equipped with a Teflon-coated magnetic stirring bar and an Argon-vacuum manifold. The flask is charged with 2-bromo-1,3-dimethylbenzene (3.02 g, 16.0 mmol) and diethyl ether (67 mL). The solution is cooled to −25°C and a solution of *tert*-butyllithium (21.0 mL, 33.6 mmol, 1.6 M in *n*-pentane) was added over 10 min. Once the addition is complete, stirring is continued for 1 h at −20°C.

A 250 mL, three-neck round-bottom flask is equipped with a Teflon-coated magnetic stirring bar, a reflux condenser, a pressure-equalizing dropping funnel fitted with a rubber stopper and an Argon-vacuum manifold. The flask is charged with $W(CO)_6$ (5.63 g, 16.0 mmol) and diethyl ether (51 mL) and the mixture is cooled to 0°C. The cold (−20 °C) solution of 2,6-dimethylphenyl lithium is added via the dropping funnel over 5 min to the suspension, causing an immediate color change to orange-brown and a slight exotherm. Once the addition is complete, the resulting orange-brown solution is stirred at ambient temperature for 2 h. The dropping funnel and the reflux condenser are replaced by a glass stopper and the solvent is removed at ambient temperature under reduced pressure. A solution of Me_4NBr (3.84 g, 24.4 mmol) in deionized water (20 mL) is added to the remaining dark yellow residue, causing the formation of an orange solid. The resulting suspension is vigorously stirred for 15 min at ambient temperature before the precipitate is filtered off in air, washed once with deionized water (5 mL), twice with cold (0 °C) diethyl ether (20 mL) and dried under high vacuum (10^{-3} mbar) to give complex **S5** as a pale orange microcrystalline solid (5.45 g, 64%). The complex is still contaminated with starting complex $W(CO_6)$, which can't be removed due to its low solubility, but turned out not to be problematic in the next step. ^{1}H NMR (600 MHz, CCl_2D_2): δ = 6.91 – 6.77 (m, 3H), 3.12 (s, 12H), 2.18 (s, 6H).^{13}C NMR (151 MHz, CCl_2D_2): δ = 298.1 (^1J-^{183}W-^{13}C = 82.4 Hz), 208.3 (^1J-^{183}W-^{13}C = 134.1 Hz), 203.5 (^1J-^{183}W-^{13}C = 127.9 Hz), 162.4 (^2J-^{183}W-^{13}C = 13.3 Hz), 128.3, 127.9, 125.0, 56.6 (^1J-^{14}N-^{13}C = 3.9 Hz), 18.8, 18.6. IR (film): $\tilde{\nu}$ 2028, 1859, 1552, 1519, 1485, 1438, 947, 849, 767, 693, 636, 596, 576, 534, 485, 440, 419 cm^{-1}. HRMS-ESI (m/z): calcd. for $C_{14}H_9O_6W^+$ $[M-C_4H_{12}N]^+$, 456.99139; found, 456.99199. Elemental analysis (%) calcd. for $C_{18}H_{21}NO_6W$: C 40.70, H 3.98, N 2.64; found: C 40.81, H 4.11, N 2.37.

Complex $[W(\equiv CAr)Br_3(dme)]$ (Ar = 2,6-dimethlyphenyl) (S6).

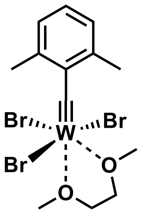

A 250 mL, three-neck round-bottom flask is equipped with a Teflon-coated magnetic stirring bar, a thermometer adapter, a rubber stopper and an Argon-vacuum manifold. The flask is charged with complex **S5** (5.82 g, 10.9 mmol) and CH_2Cl_2 (93 mL). The mixture is cooled to −78 °C and stirred for 15 min before a solution of oxalyl bromide (1.03 mL, 11.0 mmol) in CH_2Cl_2 (6 mL) is added dropwise over 10 min. Once the addition is complete, stirring is continued for 15 min at −78 °C. The mixture is then allowed to warm until a rapid color change to light brown is observed (at an internal temperature of ca. −40 °C). At this point, the reaction flask is quickly re-cooled to −78 °C.

A 1 L, three-neck jacketed cooling flask is equipped with a Teflon-coated magnetic stirring bar, a jacketed cooling filter frit (diameter: 4.5 cm; height: 20 cm; porosity: 4) containing a 3 cm-length pad of Celite®, a 20 mL pressure-equalizing dropping funnel connected to an Argon-vacuum manifold. The Celite® in the jacketed filter is slurried with CH_2Cl_2 and cooled to −78 °C using a cryostat. The 1 L, three-neck jacketed flask is loaded with 1, 2-dimethoxyethane (5.64 mL, 54.3 mmol) and cooled to −78 °C. The cold light

brownish mixture is filtered onto the Celite® packed filter frit, resulting in a bright yellow filtrate. Upon contact with DME in the 1 L receiving flask, a color change from yellow to dark red is observed. The mixture is stirred for 10 min before a solution of Br_2 (0.56 mL, 11.0 mmol) in CH_2Cl_2 (5 mL) is added dropwise over a period of 15 min via the dropping funnel. Once the addition is complete, the mixture is stirred for 15 min. The resulting brown-orange mixture is allowed to reach ambient temperature over the course of 1 h, during which time the color intensifies. Stirring is continued for 1 h at ambient temperature before the dropping funnel and the filter are replaced by glass stoppers. The reaction mixture is filtered through a pad of Celite® and the filtrate is concentrated at ambient temperature under reduced pressure to give complex **S6** as a green microcrystalline solid (6.28 g, 92%). ^{1}H NMR (600 MHz, C_6D_6): δ = 7.12 (dp, *J* = 7.7, 0.7 Hz, 2H), 6.15 (t, *J* = 7.6 Hz, 1H), 3.62 (d, *J* = 0.8 Hz, 6H), 3.47 (s, 3H), 3.38 (s, 3H), 2.96 – 2.92 (m, 2H), 2.89 – 2.85 (m, 2H). ^{13}C NMR (151 MHz, C_6D_6): δ = 337.4, 150.4, 136.9, 132.1, 125.2, 78.2, 75.5, 69.4, 60.2, 20.6. IR (film): $\tilde{\nu}$ 2935, 1445, 1080, 1030, 1010, 982, 855, 818, 775, 736, 422 cm^{-1}. Elemental analysis (%) calcd for $C_{13}H_{19}Br_3O_2W$: C 24.75, H 3.04, Br 38.00, W 29.14, O 5.07; found: C 24.63, H 3.00, Br 37.85, W 29.17.

Complex 4b

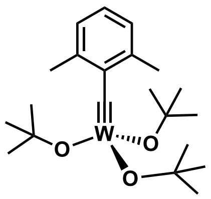

A 50 mL Schlenk flask was equipped with a magnetic stir bar and was flame dried under vaccum. The flask was filled with argon and complex **S6** (1.00 g, 1.60 mmol) was dissolved in THF (18 mL). Then a solution of NaO*t*Bu (467 mg, 4.76 mmol) in THF (4 mL) was added dropwise at 23°C to the stirred brown solution. Stirring was continued for 14 h at ambient temperature before the solvent was removed *in vacuo* to obtain a dark brown solid.
A second, flame dried 100 mL Schlenk flask was equipped with a magnetic stir bar and a Celite® (2 cm) packed argon frit. The dark brown solid was suspended in *n*-pentane (4 x 15 mL) and was filtered through the Celite® pad. The resulting filtrate was concentrated and dried under vacuum (10^{-3} mbar) to give complex **4b** as a brown solid (743 mg, 89%) free of any residual THF. Yellow crystals suitable for single-crystal X-ray diffraction were grown from a concentrated *n*-pentane solution (5 mL) stored at –85°C for one week. ^{1}H NMR (600 MHz, $C_6D_5CD_3$): δ = 7.01 (d, *J* = 7.5 Hz, 2H), 6.70 (t, *J* = 7.5 Hz, 1H), 2.87 (s, 6H), 1.40 (s, 27H). ^{13}C NMR (151 MHz, $C_6D_5CD_3$): δ = 271.3 (^{1}J-^{183}W-^{13}C = 297.3 Hz), 145.3 (^{2}J-^{183}W-^{13}C = 44.8 Hz), 140.7, 126.8, 125.9, 79.6, 32.1, 21.7. ^{183}W NMR (17 MHz, $C_6D_5CD_3$): δ = 166.0. IR (film): $\tilde{\nu}$ 2970, 2925, 1462, 1385, 1360, 1239, 1161, 1099, 1026, 944, 913, 895, 791, 780, 762, 734, 628, 597, 558, 512, 478 cm^{-1}. Elemental analysis (%) calcd for $C_{21}H_{36}WO_3$: C 48.47, H 6.97, W 35.33; found: C 47.99, H 7.02, W 34.94.

Complex 5b·THF

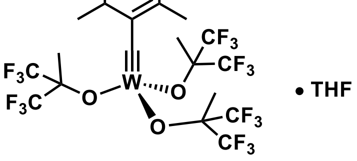

A 25 mL Schlenk flask was equipped with a magnetic stir bar and was flame dried under vaccum. The flask was filled with argon and charged with W(≡CAr)Cl_3(dme) (Ar = 2,6-$Me_2C_6H_3$) and THF (4 mL) to give a blue solution. A solution of NaOCMe$(CF_3)_3$ (153 mg, 0.814 mmol) in THF (2 mL) was then added dropwise at 23°C to give a pink suspension. Stirring was continued for 14 h at ambient temperature before the solvent was removed *in vacuo*. The solid material was extracted with *n*-pentane (4 x 1 mL) and the supernatant transferred via cannula into a second flame dried 10 mL Schlenk flask. The combined filtrates were concentrated and the residue was dried under vacuum (10^{-3} mbar) and co-evaporated with toluene (2 x 2 mL) to give complex **5b** as a THF adduct (215 mg, 93%). ^{1}H NMR (600 MHz, $C_6D_5CD_3$): δ = 6.90 (dp, *J* = 7.6, 0.7 Hz, 2H), 6.56 (tp, *J* = 7.6, 0.4 Hz, 1H),

3.80 (t, J = 6.3 Hz, 4H), 2.68 (s, 6H), 1.62 (s, 9H), 1.37 – 1.26 (m, 4H). ^{13}C NMR (151 MHz, $C_6D_5CD_3$): δ = 289.8 (^1J-^{183}W-^{13}C = 283.9 Hz), 144.7, 141.5 (^2J-^{183}W-^{13}C = 40.6 Hz), 129.6, 126.8, 124.7 (q, J = 288.6 Hz), 83.4 (hept, J = 29.0 Hz), 74.4, 25.6, 20.7, 18.5. ^{19}F NMR (565 MHz, $C_6D_5CD_3$): δ = –76.6. ^{183}W NMR (17 MHz, $C_6D_5CD_3$): δ = 395.7. HRMS-ESI (m/z): calcd. for $C_{21}H_{18}F_{18}O_3W^+$ [M-THF]$^+$: 844.04724; found, 844.04815.

Complex 16a

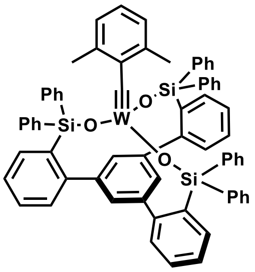

A 100 mL Schlenk flask was equipped with a magnetic stir bar and was flame dried under vacuum. The flask was filled with argon, charged with ligand **15a** (617 mg, 0.685 mmol) which was azeotropically dried with benzene (3 x 5 mL) to remove any residual water. Toluene (51 mL) was then added and the mixture vigorously stirred for 10 min to obtain a clear solution. A solution of complex **4b** (374 mg, 0.719 mmol) in toluene (10 mL) was added dropwise to the vigorously stirred mixture at 23°C. After 3 h stirring at ambient temperature, the solvent was removed *in vacuo* and the yellow/orange solid was washed with diethyl ether (4 x 5 mL) to give complex **16a** as a yellow/orange powder (646 mg, 79%). Yellow crystals suitable for single-crystal X-ray diffraction were grown from a concentrated Et_2O solution (5 mL) stored at –85°C for 1 week. ^{1}H NMR (600 MHz, $C_6D_5CD_3$): δ = 7.81 – 7.78 (m, 12H), 7.77 – 7.74 (m, 3H), 7.24 (s, 3H), 7.11 – 7.04 (m, 12H), 7.03 – 7.00 (m, 12H), 6.86 – 6.82 (m, 3H), 6.66 (m, 2H), 6.32 (t, J = 7.5 Hz, 1H), 2.07 (s, 6H). ^{13}C NMR (151 MHz, $C_6D_5CD_3$): δ = 287.4 (^1J-^{183}W-^{13}C = 270.1 Hz), 149.2, 144.1, 143.6 (^2J-^{183}W-^{13}C = 37.1 Hz), 141.2, 137.5, 136.8, 134.9, 134.5, 130.3, 130.2, 130.0, 129.0, 128.0, 126.6, 126.2, 125.1, 20.1. ^{29}Si NMR (79 MHz, $C_6D_5CD_3$): δ = −8.5 (^2J-^{183}W-^{29}Si = 19.4 Hz). ^{183}W NMR (17 MHz, $C_6D_5CD_3$): δ = 516.5. ^{1}H-DOSY NMR ($C_6D_5CD_3$): $D_{predicted.}$ = $5.37\cdot10^{-10}$ m^2/s^{-2}; $D_{exp.}$ = $5.79\cdot10^{-10}$ m^2/s^{-2}. IR (film): $\tilde{\nu}$ 1468, 1428, 1113, 1087, 1038, 1048, 998, 943, 900, 765, 735, 721, 696, 623, 576, 545, 508, 436, 409 cm^{-1}. HRMS-ESI (m/z): calcd. for $C_{69}H_{55}O_3Si_3W^+$ [M+H]$^+$, 1199.2963; found, 1199.2971. Elemental analysis (%) calcd. for $C_{69}H_{54}O_3Si_3W$: C 69.10, H 4.54, Si 7.03, W 15.33; found: C 68.89, H 4.53, Si 6.94, W 15.13.

Complex 16b

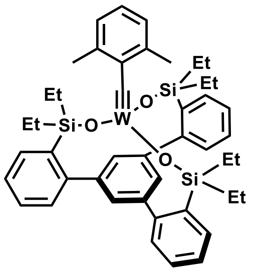

A 100 mL Schlenk flask was equipped with a magnetic stir bar and was flame dried under vacuum. The flask was filled with argon and charged with ligand **15b** (204 mg, 0.333 mmol), which was azeotropically dried with benzene (3 x 5 mL) to remove any residual water. Toluene (25 mL) was added and the mixture vigorously stirred for 10 min to obtain a clear solution before a solution of complex **4b** (173 mg, 0.333 mmol) in toluene (5 mL) was added dropwise with vigorous stirring. After 2 h stirring at ambient temperature, the solvent was removed *in vacuo* and the yellow/orange solid was extracted with *n*-pentane (4 x 5 mL). The solvent was removed *in vacuo* to give complex **16b** as a yellow/orange solid (298 mg, 98%). ^{1}H NMR (600 MHz, $C_6D_5CD_3$): δ = 7.41 (s, 3H), 7.40 – 7.34 (m, 3H), 7.25 – 7.20 (m, 3H), 7.20 – 7.13 (m, 6H), 7.01 (d, J = 7.5 Hz, 2H), 6.55 (t, J = 7.5 Hz, 1H), 2.83 (s, 6H), 1.00 – 0.91 (m, 18H), 0.98 – 0.83 (m, 12H). ^{13}C NMR (151 MHz, $C_6D_5CD_3$): δ = 281.5 (^1J-^{183}W-^{13}C = 272.5 Hz), 149.4, 144.7, 144.6 (^2J-^{183}W-^{13}C = 38.8 Hz), 140.5, 135.7, 134.6, 130.8, 129.2, 128.0, 126.7, 126.4, 125.9, 21.0, 9.2, 7.1. ^{29}Si NMR (119 MHz, $C_6D_5CD_3$): δ = 12.9. ^{183}W NMR (17 MHz, $C_6D_5CD_3$): δ = 460.0. ^{1}H-DOSY NMR ($C_6D_5CD_3$): $D_{predicted.}$ = $6.08\cdot10^{-10}$ m^2/s^{-2}; $D_{exp.}$ = $6.94\cdot10^{-10}$ m^2/s^{-2}. IR (film): $\tilde{\nu}$ 3049, 2953, 2910, 2873, 1583, 1557, 1459, 1408, 1377, 1260, 1232, 1123, 1090, 1046, 1027, 1005, 932, 881, 760,

712, 672, 623, 575, 553, 527, 508, 460 cm^{-1}. Elemental analysis (%) calcd. for $C_{45}H_{54}O_3Si_3W$: C 59.33, H 5.97, Si 9.25, W 20.18; found: C 58.87, H 5.83, Si 9.21, W 19.98.

Complex 16c

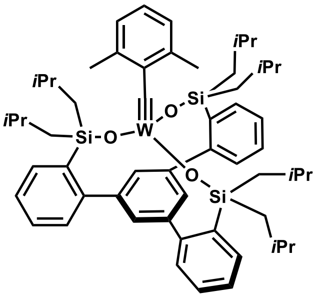

A 100 mL Schlenk flask was equipped with a magnetic stir bar and was flame dried under vacuum. The flask was filled with argon and charged with ligand **15c** (296 mg, 0.379 mmol), which was azeotropically dried with benzene (3 x 5 mL) to remove any residual water. Toluene (29 mL) was added and the mixture vigorously stirred for 10 min to obtain a clear solution before a solution of complex **4b** (199 mg, 0.382 mmol) in toluene (6 mL) was added to the vigorously stirred mixture. After 2 h stirring at ambient temperature, the solvent was removed *in vacuo* and the yellow/orange solid was extracted with *n*-pentane (4 x 5 mL). The solvent was removed *in vacuo* to give complex **16c** as a yellow/orange powder (407 mg, quant.). ^{1}H NMR (600 MHz, $C_6D_5CD_3$): δ = 7.55 – 7.49 (m, 3H), 7.47 (s, 3H), 7.26 – 7.21 (m, 3H), 7.20 – 7.15 (m, 6H), 6.99 (dm, *J* = 7.6 Hz, 2H), 6.53 (t, *J* = 7.5 Hz, 1H), 2.86 (s, 6H), 2.06 – 1.98 (m, *J* = 6.6 Hz, 6H), 1.08 – 0.98 (m, 12H), 0.88 (d, *J* = 6.6 Hz, 18H), 0.87 (d, *J* = 6.6 Hz, 18H). ^{13}C NMR (151 MHz, $C_6D_5CD_3$): δ = 281.9 (^{1}J-^{183}W-^{13}C = 272.8 Hz), 148.9, 144.7, 144.4 (^{2}J-^{183}W-^{13}C = 38.6 Hz), 140.5, 137.1, 135.2, 130.8, 129.2, 128.0, 126.5, 126.4, 125.8, 29.7, 26.9, 26.5, 24.5, 21.5. ^{29}Si NMR (119 MHz, $C_6D_5CD_3$): δ = 9.9. ^{183}W NMR (17 MHz, $C_6D_5CD_3$): δ = 475.2. IR (film): $\tilde{\nu}$ 2950, 2924, 2895, 2864, 1584, 1557, 1462, 1408, 1381, 1363, 1327, 1260, 1215, 1162, 1123, 1088, 1047, 1019, 925, 829, 758, 733, 661, 622, 576, 552, 505, 475, 408 cm^{-1}. HRMS-ESI (m/z): calcd. for $C_{57}H_{79}O_3Si_3W^+$ [M+H]$^+$, 1079.48409; found, 1079.48429. Elemental analysis (%) calcd. for $C_{57}H_{78}O_3Si_3W$: C 63.43, H 7.28, Si 7.81, W 17.03; found: C 63.59, H 7.31, Si 7.74, W 16.71.

Complex 16d

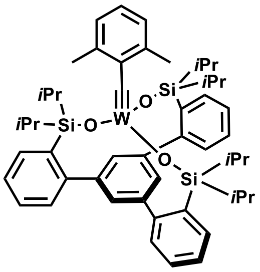

A 100 mL Schlenk flask was equipped with a magnetic stir bar and was flame dried under vacuum. The flask was filled with argon and charged with ligand **15d** (291 mg, 0.471 mmol), which was azeotropically dried with benzene (3 x 5 mL) to remove any residual water. Toluene (31 mL) was added and the mixture vigorously stirred for 10 min to obtain a clear solution, before a solution of complex **4b** (217 mg, 0.417mmol) in toluene (6 mL) was added dropwise to the vigorously stirred mixture. After 2 h stirring at ambient temperature, the solvent was removed *in vacuo* and the yellow/orange solid was extracted with *n*-pentane (4 x 5 mL). The solvent was removed *in vacuo* to give complex **16d** as yellow/orange powder (413 mg, 99%). ^{1}H NMR (600 MHz, $C_6D_5CD_3$): δ = 7.45 – 7.42 (m, 3H), 7.38 (s, 3H), 7.23 – 7.19 (m, 3H), 7.18 – 7.12 (m, 6H), 6.98 (d, *J* = 7.5 Hz, 2H), 6.53 (t, *J* = 7.5 Hz, 1H), 2.86 (s, 6H), 1.37 (hept, *J* = 7.5 Hz, 6H), 1.11 (d, *J* = 7.5 Hz, 18H), 1.10 (d, *J* = 7.4 Hz, 18H). ^{13}C NMR (151 MHz, $C_6D_5CD_3$): δ = 282.1 (^{1}J-^{183}W-^{13}C = 274.3 Hz), 149.7, 144.5 (^{2}J-^{183}W-^{13}C = 38.7 Hz), 144.3, 140.5, 134.4, 134.3, 131.2, 128.9, 127.7, 126.5, 126.3, 125.9, 21.5, 18.3, 17.9, 15.1. ^{29}Si NMR (119 MHz, $C_6D_5CD_3$): δ = 11.6 (^{2}J-^{183}W-^{29}Si = 8.1 Hz). ^{183}W NMR (17 MHz, $C_6D_5CD_3$): δ = 460.0. ^{1}H-DOSY NMR ($C_6D_5CD_3$): $D_{predicted.}$ = 5.84·10^{-10} m^2/s^{-2}; $D_{exp.}$ = 6.79·10^{-10} m^2/s^{-2}. IR (film): $\tilde{\nu}$ 3050, 2943, 2864, 1705, 1583, 1556, 1462, 1408, 1384, 1364, 1293, 1259, 1241, 1162, 1123, 1089, 1065, 1045, 1017, 1005, 924, 880, 759, 735, 722, 693, 666, 653, 626, 596, 577, 535, 513, 497, 466 cm^{-1}. HRMS-ESI (m/z): calcd. for $C_{51}H_{67}O_3Si_3W$ [M+H]$^+$ 995.39018; found, 995.39051. Elemental analysis (%) calcd. for $C_{51}H_{66}O_3Si_3W$: C 61.55, H 6.69, Si 8.47, W 18.47; found: C 58.85, H 6.89, Si 8.12, W 17.66.

Complex 8b

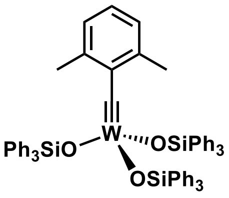

A 100 mL Schlenk flask was equipped with a magnetic stir bar and was flame dried under vacuum. The flask was filled with argon and charged with triphenylsilanol (510 mg, 1.81 mmol), which was azeotropically dried with benzene (3 x 5 mL) to remove any residual water. Toluene (45 mL) was added and the mixture vigorously stirred for 10 min to obtain a clear solution bwefore a solution of complex **4b** (345 mg, 0.663 mmol) in toluene (9 mL) was added dropwise to the vigorously stirred mixture. After stirring for 3 h at ambient temperature, the solvent was removed *in vacuo* to obtain a yellow/orange foam. Addition of diethyl ether (10 mL) gave a yellow solid and an orange-brown supernatant solution. The suspension was stored for 14 h at −85° before the orange-brown supernatant solution was removed by cannula filtration. The yellow solid was washed with *n*-pentane (3 x 5 mL) to give complex **8b** (305 mg, 41%) and an impurity (~28%), which could not be fully removed due to its similar solubility. ^{1}H NMR (600 MHz, $C_6D_5CD_3$): δ = 7.68 – 7.64 (m, 18H), 7.17 – 7.11 (m, 9H), 7.07 – 7.02 (m, 18H), 6.71 (d, *J* = 7.5 Hz, 2H), 6.45 (t, *J* = 7.5 Hz, 1H), 2.00 (s, 6H). ^{13}C NMR (151 MHz, $C_6D_5CD_3$): δ = 283.1 (^{1}J-^{183}W-^{13}C = 273.0 Hz), 143.5 (^{2}J-^{183}W-^{13}C = 38.1 Hz), 141.8, 135.7, 135.5, 130.2, 128.1, 126.7, 125.5, 20.3. ^{29}Si NMR (79 MHz, $C_6D_5CD_3$): δ = -8.03 (^{2}J-^{183}W-^{29}Si = 13.6 Hz). ^{183}W NMR (17 MHz, $C_6D_5CD_3$): δ = 548.0. HRMS-ESI (m/z): calcd. for $C_{63}H_{55}O_3Si_3W^+$ [M+H]$^+$ 1127.29628; found, 1127.29847.

Complex 19

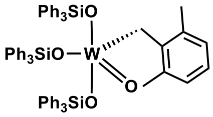

A 100 mL Schlenk flask was equipped with a magnetic stir bar and was flame dried under vacuum. The flask was filled with argon, charged with complex **4b** (269 mg, 0.518 mmol) and toluene (39 mL). A solution of triphenylsilanol (438 mg, 1.56 mmol) in toluene (8 mL) was added dropwise to the vigorously stirred mixture at 23°C. After stirring for 2.5 h at ambient temperature, water (9.3 µL, 0.518 mmol) was added and stirring was continued for 2 h before the solvent was removed *in vacuo*. The reaction mixture containing complex **19** was analyzed by NMR spectroscopy. Orange crystals suitable for single-crystal X-ray diffraction were grown from a concentrated Et_2O solution (5 mL) store at –18°C for one week. ^{1}H NMR (600 MHz, C_6D_6): δ = 7.58 – 7.52 (m, 18H), 7.15 – 7.11 (m, 9H), 7.03 – 6.96 (m, 18H), 6.68 (d, *J* = 7.5 Hz, 2H), 6.48 (t, *J* = 7.5 Hz, 1H), 3.70 (^{2}J-^{183}W-^{1}H = 13.3 Hz, s, 2H), 2.10 (s, 6H). ^{13}C NMR (151 MHz, C_6D_6): δ = 146.1, 136.1, 135.0, 134.9, 130.2, 128.1, 127.3, 125.5, 64.4 (^{1}J-^{183}W-^{13}C = 101.9 Hz), 20.8. ^{29}Si NMR (79 MHz, C_6D_6): δ = –0.4 (^{2}J-^{183}W-^{29}Si = 11.5 Hz). ^{183}W NMR (17 MHz, $C_6D_6CD_3$): δ = 758.1. HRMS: decomp.

Complex 10b

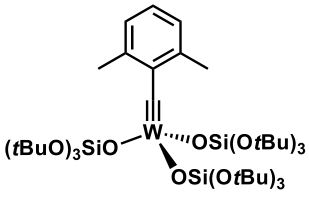

A 50 mL Schlenk flask was equipped with a magnetic stir bar and was flame dried under vacuum. The flask was filled with argon, charged with complex **7b** (757 mg, 1.20 mmol) and dissolved in CH_2Cl_2 (14 mL). Then a solution of LiOSi(O*t*Bu)$_3$ (973 mg, 3.60 mmol) in CH_2Cl_2 (7 mL) was added dropwise to the vigorously stirred green solution at 23°C, which turned instantly into a yellow suspension. After stirring for 14 h at ambient temperature, the solvent was removed *in vacuo* and the yellow/orange solid was extracted with *n*-pentane (4 x 5 mL). The solvent was removed *in vacuo* to give complex **10b** as yellow crystalline material (1.31 g, 99%). Yellow crystals suitable for single-crystal X-ray diffraction were obtained from a concentrated Et_2O solution (5 mL) upon slow evaporation of the solvent. ^{1}H NMR (600 MHz, $C_6D_5CD_3$): δ 7.05 – 7.02 (m, 2H), 6.59 (t, *J* = 7.5 Hz, 1H), 3.08 (s, 6H), 1.43 (s, 81H).

^{13}C NMR (151 MHz, $C_6D_5CD_3$): δ = 281.2 (^{1}J-^{183}W-^{13}C = 274.8 Hz), 144.0 (^{2}J-^{183}W-^{13}C = 38.7 Hz), 142.1, 126.7, 125.6, 73.3, 31.9, 22.3. ^{29}Si NMR (119 MHz, $C_6D_5CD_3$): δ = −93.2 (^{2}J-^{183}W-^{29}Si = 10.4 Hz). ^{183}W NMR (17 MHz, $C_6D_5CD_3$): δ = 458.1. ^{1}H-DOSY NMR ($C_6D_5CD_3$): $D_{predicted.}$ = 5.60·10^{-10} m^2/s^{-2}; $D_{exp.}$ = 7.216·10^{-10} m^2/s^{-2}. IR (film): $\tilde{\nu}$ 2972, 2930, 2872, 1470, 1389, 1364, 1241, 1188, 1053, 1026, 906, 829, 765, 734, 697, 649, 580, 510, 536, 470, 429 cm^{-1}. HRMS: decomp.; Elemental analysis (%) calcd. for $C_{45}H_{90}O_{12}Si_3W$: C 49.53, H 8.31, Si 7.72, W 16.85; found: C 48.89, H 8.41, Si 7.41, W 16.39.

Complex 23

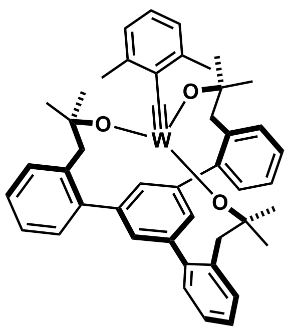

A 500 mL Schlenk flask was equipped with a magnetic stir bar and was flame dried under vacuum. The flask was filled with argon and charged with ligand **22** (1.03 g, 1.98 mmol), which was azeotropically dried with benzene (3 x 5 mL) to remove any residual water. Toluene (148 mL) was added and the mixture was vigorously stirred for 10 min to obtain a clear solution. A solution of complex **4b** (1.03 g, 1.98 mmol) in toluene (30 mL) was then added dropwise to the vigorously stirred mixture. After stirring for 2 h at ambient temperature, the solvent was removed *in vacuo* to give complex **23** as an orange powder (1.62 g, quant.). ^{1}H NMR (600 MHz, $C_6D_5CD_3$): δ 7.30 (dd, J = 7.4, 1.7 Hz, 3H), 7.29 (s, 3H), 7.15 (td, J = 7.4, 1.6 Hz, 3H), 7.12 (td, J = 7.3, 1.7 Hz, 3H), 6.99 (d, J = 7.5 Hz, 2H), 6.93 (dd, J = 7.4, 1.6 Hz, 3H), 6.65 (t, J = 7.5 Hz, 1H), 3.07 (s, 6H), 2.73 (s, 6H), 1.30 (s, 18H). ^{13}C NMR (151 MHz, $C_6D_5CD_3$): δ = 264.0 (^{1}J-^{183}W-^{13}C = 292.7 Hz), 145.6 (^{2}J-^{183}W-^{13}C = 44.2 Hz), 144.5, 143.3, 139.5, 136.4, 133.4, 132.6, 128.3, 127.1, 127.0, 126.8, 125.2, 83.9, 49.3, 31.4, 22.1. ^{183}W NMR (17 MHz, $C_6D_5CD_3$): δ = 114.2. IR (film): $\tilde{\nu}$ 3032, 2973, 2922, 1458, 1478, 1363, 1377, 1207, 1227, 1170, 1124, 1098, 972, 984, 999, 940, 896, 872, 786, 814, 757, 739, 675, 624, 639, 572, 590, 535, 558, 512, 472, 415 cm^{-1}. HRMS: decomp.; Elemental analysis (%) calcd. for $C_{45}H_{48}O_3W$: C 65.86, H 5.90, W 22.40; found C 65.63, H 5.93, W 22.19.

Complex 17

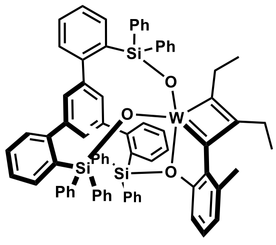

A 10 mL Schlenk flask was equipped with a magnetic stir bar and was flame dried under vacuum. The flask was filled with argon and charged with complex **16a** (32.3 mg, 0.027 mmol) and Et_2O (1 mL) to give a yellow suspension. Upon addition of 3-hexyne (10.7 mg, 14.8 µL) the color instantly changed to deep red. This solution was filtered via cannula and the filtrate was stored at –20°C for one week to obtain very sensitive, red crystals suitable for single-crystal X-ray diffraction.

NMR analysis: A 10 mL Schlenk flask was equipped with a magnetic stir bar and was flame dried under vacuum. The flask was filled with argon and charged with complex **16a** (29.3 mg, 0.025 mmol) and $C_6D_5CD_3$ (0.5 mL) to give a deep orange solution. This solution was transferred into a flame-dried *J. Young* NMR tube under Ar. Upon addition of 3-hexyne (13.9 µL, 0.122 mmol) the color instantly changed to deep red. The signals of the new complex were as follows: ^{1}H NMR (600 MHz, $C_6D_5CD_3$): δ = 8.41 (t, J = 1.7 Hz, 1H), 8.02 – 7.97 (m, 4H), 7.90 – 7.87 (m, 1H), 7.70 (d, J = 1.6 Hz, 2H), 7.57 – 7.52 (m, 2H), 7.51 – 7.46 (m, 4H), 7.43 (dt, J = 7.7, 1.0 Hz, 2H), 7.24 – 7.21 (m, 4H), 7.19 (td, J = 7.3, 1.3 Hz, 3H), 7.18 (td, J = 7.6, 1.5 Hz, 1H), 7.13 – 7.03 (m, 4H), 7.01 – 6.96 (m, 8H), 6.86 (s, 9H), 6.75 (d, J = 7.6 Hz, 2H), 3.05 (q, J = 7.3 Hz, 2H), 2.52 (q, J = 7.6 Hz, 2H), 1.47 (s, 6H), 0.67 (t, J = 7.6 Hz, 3H), 0.26 (t, J = 7.6 Hz, 3H). ^{13}C NMR (151

MHz, $C_6D_5CD_3$): δ = 231.2 (^1J-^{183}W-^{13}C = 86.8 Hz), 228.9 (^1J-^{183}W-^{13}C = 153.0 Hz), 149.8, 148.8, 147.0, 143.7, 142.7, 140.1, 139.4, 139.2, 138.7, 138.1, 137.2, 136.7, 135.8, 135.8, 135.6, 135.1, 134.6 (^2J-^{183}W-^{13}C = 23.0 Hz), 131.2, 131.0, 129.4, 129.2, 129.2, 129.1, 129.0, 128.7, 128.0, 127.6, 127.6, 127.4, 127.2, 126.2, 126.2, 125.3, 29.2, 27.1, 20.9, 15.3, 13.2. ^{29}Si NMR (119 MHz, $C_6D_5CD_3$): = –18.02 (^2J-^{183}W-^{29}Si = 15.6 Hz), –18.81 (^2J-^{183}W-^{29}Si = 16.6 Hz). A ^{183}W-NMR signal could not be detected; HRMS: decomp.; because of the sensitivity of the material, a correct elemental analysis has not been obtained.

Complex 18

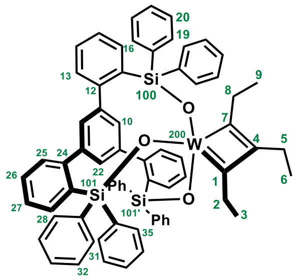

The NMR sample of complex **17** was stored for one week at ambient temperatures upon which time complex **18** was formed. Not all shifts of the new complex **18** in the ^{1}H and ^{13}C NMR NMR could be assigned due to signal broadening and overlaps with complex **17** (see attached spectra for more details).

Table S-1. Assigned NMR signals of complex **18**.

Atom	δ (ppm)	J	COSY	HSQC	NOESY	Atom	δ (ppm)	J	COSY	HSQC	NOESY
1 C	233.72	89.20(200)				20 C					
2 C	28.25			2		H	6.93		19		
H2	2.13		3	2	5, 10, 22	22 C	125.73			22	
3 C	15.5			3		H	8.23		10	22	2, 8, 13, 25
H3	0		2	3	5, 19, 31, 35	24 C	149.45				
4 C	136.58					25 C	130.98			25	
5 C	26.06			5		H	7.35		26	25	22
H2	2.77		6	5	2, 3, 8, 9, 19, 31, 35	26 C					
6 C	13.36			6		H	7.15		25		
H3	0.92		5	6	8, 19, 31, 35	27 C					
7 C	229.77	152.00(200)				H	7.04		28		
8 C	29.91			8		28 C	137.08			28	
H2	3.81		9	8	5, 6, 10, 19, 22, 31, 35	H	7.75		27	28	
9 C	16.28			9		31 C	134.8			31	
H3	1.08		8	9	5, 19, 31, 35	H	7.6		32	31	3, 5, 6, 8, 9
10 C	130.55			10		32 C					
H	7.56		22	10	2, 8	H	6.91		31		
12 C	148.34					35 C				35	
13 C				13		H	7.13			35	3, 5, 6, 8, 9
H	7.04			13	22	100 Si	-18.08				
16 C	134.96			16		101 Si	-20.71				
H	7.66			16		101' Si	-20.71				
19 C	135.94			19		200 W		152.00(7), 89.20(1)			
H	8.15		20	19	3, 5, 6, 8, 9						

Alkyne Metathesis Reactions

Homo-Metathesis. 1,2-Bis(4-methoxyphenyl)ethyne. 1-Methoxy-4-(prop-1-yn-1-yl)benzene (36.5 mg, 25.0 µmol) was added to a stirred suspension of complex **1a** (13.9 mg, 1.25 µmol, 5 mol%) and silanized, powdered molecular sieves 5Å (250 mg)[7] in toluene (1.25 mL) at 23 °C under argon atmosphere. The mixture was stirred for 2 h at this temperature before it was filtered through a short pad of Celite® which was carefully rinsed with *t*-butyl methyl ether (20 mL). The combined filtrates were evaporated and the title compound was obtained (85% NMR yield). ^{1}H NMR (400 MHz, $CDCl_3$): δ = 7.45 (d, *J* = 8.8 Hz, 3H), 6.87 (d, *J* = 8.8 Hz, 4H), 3.82 (s, 6H). The analytical and spectroscopic data are in agreement with those reported in the literature.[8]

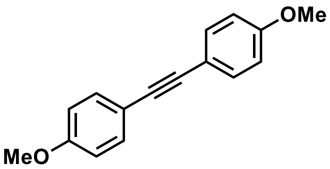

Representative Procedure for Ring Closing Alkyne Metathesis. 1,8-Dioxacyclotetradec-11-yne-2,7-dione (25). Di(pent-3-yn-1-yl) adipate (27.8 mg, 0.100 mmol) was added to a stirred solution of complex **1a** (4.1 mg, 0.5 µmol, 5 mol%) in toluene (50 mL) at 80 °C under argon atmosphere. The mixture was stirred for 4 h at this temperature before it was allowed to cool to ambient temperature and filtered through a short pad of Celite®, which was carefully rinsed with ethyl acetate (20 mL). The combined filtrates were evaporated and the residue was purified by flash chromatography on silica gel (hexanes/*t*-butyl methyl ether, 9:1 to 6:4) to give the title compound as a white solid (21.9 mg, 98%). ^{1}H NMR (400 MHz, $CDCl_3$): δ = 4.17 – 4.10 (m, 4H), 2.56 – 2.47 (m, 4H), 2.38 (td, *J* = 4.1, 1.8 Hz, 4H), 1.82 – 1.69 (m, 4H). The analytical and spectroscopic data are in agreement with those reported in the literature.[9]

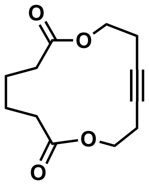

Compound 26

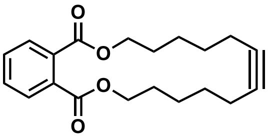

Prepared analogously; white solid (23.4 mg, 71%). ^{1}H NMR (400 MHz, $CDCl_3$): δ = 7.76 – 7.69 (m, 2H), 7.53 (dd, *J* = 5.8, 3.3 Hz, 2H), 4.35 (t, *J* = 6.1 Hz, 4H), 2.23 – 2.13 (m, 4H), 1.79 (dq, *J* = 9.0, 5.7 Hz, 4H), 1.65 – 1.44 (m, 8H). The analytical and spectroscopic data are in agreement with those reported in the literature.[1,9,10]

Compound 27

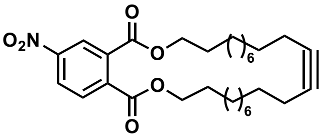

Prepared analogously; white solid (40.7 mg, 79%). ^{1}H NMR (400 MHz, $CDCl_3$): δ = 8.61 (d, *J* = 2.3 Hz, 1H), 8.38 (dd, *J* = 8.5, 2.3 Hz, 1H), 7.85 (d, *J* = 8.5 Hz, 1H), 4.33 (td, *J* = 7.0, 4.7 Hz, 4H), 2.17 (h, *J* = 2.1 Hz, 4H), 1.75 (h, *J* = 7.2 Hz, 4H), 1.48 – 1.28 (m, 28H). ^{13}C NMR (101 MHz, $CDCl_3$): δ = 166.5, 165.4, 148.9, 138.4, 133.4, 130.3, 126.0, 124.6, 80.7, 66.8, 29.8, 29.8, 29.7, 29.6, 29.5, 29.3, 29.3, 26.0, 25.9, 18.7. The analytical and spectroscopic data are in agreement with those reported in the literature.[1,9,10]

Compound 28

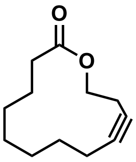

Prepared analogously; white solid (12.8 mg, 66%). ^{1}H NMR (400 MHz, $CDCl_3$): δ = 4.20 – 4.13 (m, 2H), 2.52 (tt, *J* = 5.3, 2.5 Hz, 2H), 2.42 – 2.34 (m, 2H), 1.80 – 1.68 (m, 2H), 1.51 – 1.33 (m, 8H). ^{13}C NMR (101 MHz, $CDCl_3$): δ = 174.1, 82.9, 77.0, 62.8, 34.6, 27.8, 27.3, 27.1, 26.8, 24.8, 19.5, 18.9. The analytical and spectroscopic data are in agreement with those

reported in the literature.[10]

Compound 29

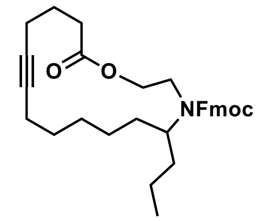

Prepared analogously; white solid (39.4 mg, 79%). ^{1}H NMR (rotamers, 400 MHz, $CDCl_3$): δ = 7.76 (dd, *J* = 7.7, 2.2 Hz, 2H), 7.63 – 7.53 (m, 2H), 7.45 – 7.27 (m, 4H), 4.73 – 4.49 (m, 2H), 4.22 (q, *J* = 5.2 Hz, 2H), 3.92 – 3.66 (m, 1H), 3.42 (s, 0H), 2.99 (s, 1H), 2.70 – 2.35 (m, 1H), 2.32 – 2.13 (m, 3H), 2.13 – 2.01 (m, 1H), 1.86 – 1.69 (m, 2H), 1.61 – 0.77 (m, 14H), 0.70 (s, 2H). ^{13}C NMR (rotamers, 101 MHz, $CDCl_3$): δ = 173.5, 173.4, 144.3, 144.2, 144.1, 141.6, 141.6, 127.8, 127.8, 127.7, 127.3, 127.2, 127.2, 127.1, 124.8, 124.7, 120.1, 120.1, 120.0, 120.0, 79.8, 79.6, 77.4, 66.6, 66.4, 63.6, 47.8, 47.5, 35.1, 34.8, 34.7, 32.6, 32.5, 28.6, 28.4, 26.9, 26.8, 23.7, 23.6, 19.7, 18.4, 17.9, 14.2, 14.0. The analytical and spectroscopic data are in agreement with those reported in the literature.[9, 10]

Compound 30

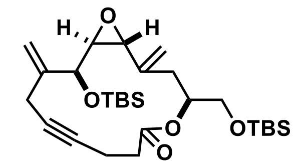

Prepared analogously; white solid (2.6 mg, 50%). ^{1}H NMR (400 MHz, $CDCl_3$): δ 5.19 (p, *J* = 1.1 Hz, 1H), 5.14 (d, *J* = 1.2 Hz, 1H), 5.12 – 5.06 (m, 1H), 5.05 (t, *J* = 1.5 Hz, 1H), 5.01 (p, *J* = 1.2 Hz, 1H), 4.12 (d, *J* = 4.8 Hz, 1H), 3.71 (dd, *J* = 10.4, 5.0 Hz, 1H), 3.60 (dd, *J* = 10.4, 5.7 Hz, 1H), 3.31 (dd, *J* = 2.1, 0.8 Hz, 1H), 3.19 (d, *J* = 17.6 Hz, 1H), 3.14 (dd, *J* = 4.8, 2.1 Hz, 1H), 2.93 (d, *J* = 17.6 Hz, 1H), 2.55 – 2.32 (m, 6H), 0.91 (s, 9H), 0.89 (s, 9H), 0.14 (s, 3H), 0.05 (d, *J* = 0.8 Hz, 9H). ^{13}C NMR (101 MHz, $CDCl_3$): δ = 171.7, 143.9, 141.2, 114.2, 114.1, 80.4, 78.8, 74.9, 72.6, 64.5, 62.7, 57.0, 34.3, 34.1, 26.0, 25.9, 22.7, 18.4, 18.4, 15.3, –4.7, –4.9, –5.3, –5.3. The analytical and spectroscopic data are in agreement with those reported in the literature.[11]

Compound 31

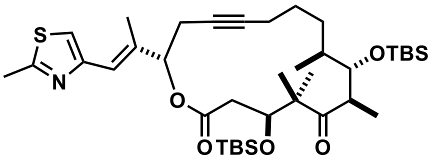

Prepared analogously; white solid (4.4 mg, 53%). ^{1}H NMR (400 MHz, $CDCl_3$): δ = 6.96 (s, 1H), 6.55 (s, 1H), 5.34 (dd, *J* = 7.3, 3.4 Hz, 1H), 4.70 (dd, *J* = 6.4, 4.8 Hz, 1H), 3.94 (dd, *J* = 6.6, 1.9 Hz, 1H), 3.31 – 3.12 (m, 1H), 2.83 – 2.74 (m, 1H), 2.71 (s, 3H), 2.71 – 2.53 (m, 3H), 2.18 (d, *J* = 1.3 Hz, 3H), 2.39 – 1.94 (m, 3H), 1.81 – 1.35 (m, 1H), 1.17 (s, 3H), 1.15 (s, 3H), 1.11 (s, 3H), 0.95 – 0.94 (m, 3H), 0.92 (s, 9H), 0.87 (s, 9H), 0.10 (s, 6H), 0.09 (s, 3H), 0.07 (d, *J* = 2.2 Hz, 6H). ^{13}C NMR (101 MHz, $CDCl_3$): δ = 216.7, 170.2, 164.9, 152.6, 137.0, 120.7, 116.9, 82.3, 78.2, 77.4, 76.5, 72.8, 54.7, 53.5, 44.6, 41.8, 39.1, 29.9, 26.4, 26.2, 26.1, 24.3, 21.2, 20.7, 19.4, 18.8, 18.7, 18.5, 17.1, 15.2, −3.1, −3.6, −3.9, −3.9. The analytical and spectroscopic data are in agreement with those reported in the literature.[12]

^{1}H, ^{183}W NMR HMBC Spectrum

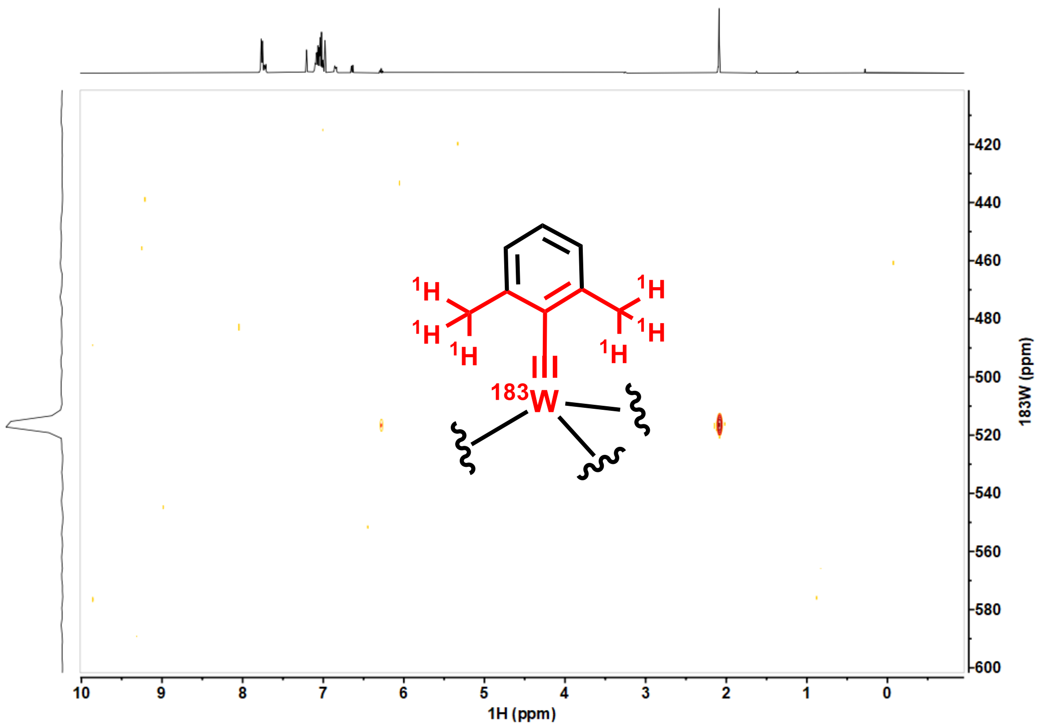

Figure S-11. ^{1}H, ^{183}W HMBC NMR spectrum of complex **16a.**

^{1}H, ^{183}W HMBC Projections: Folding Screening

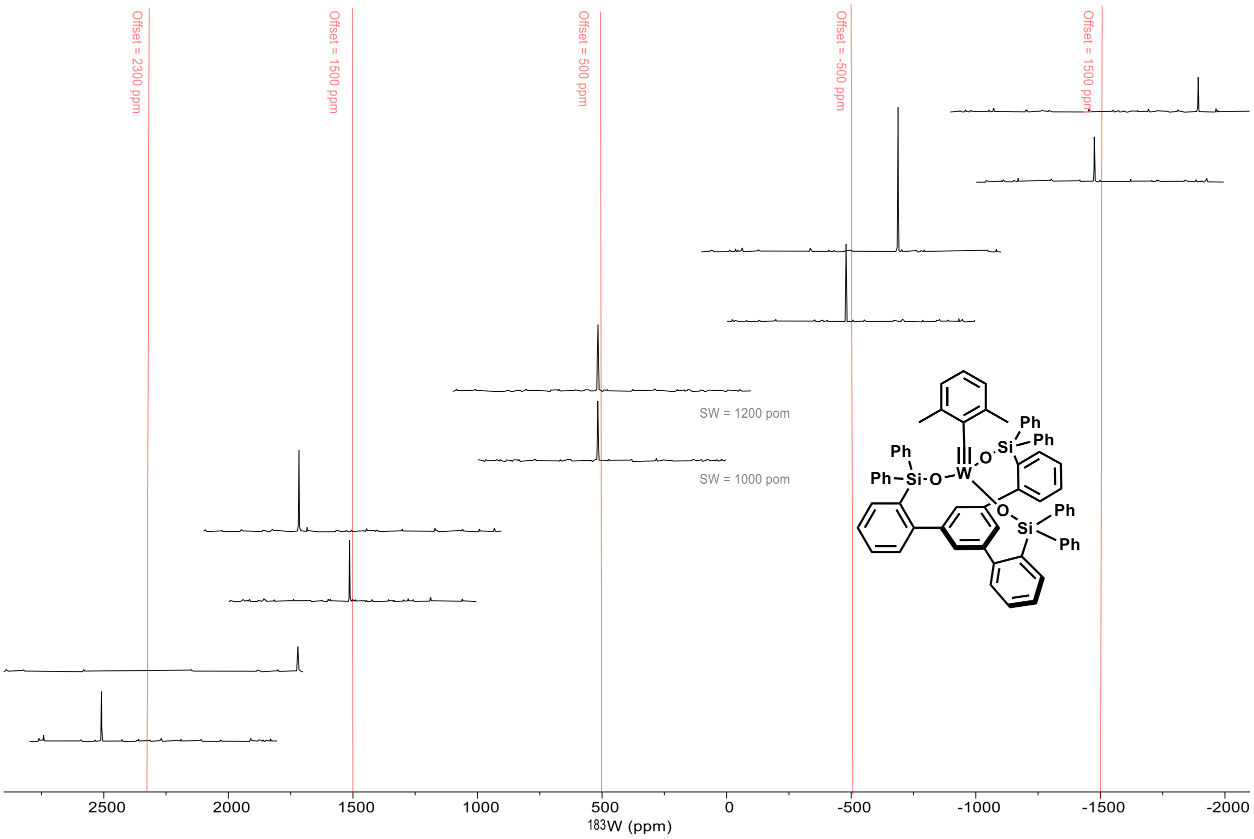

Figure S-12. Comparison of different ^{183}W projections extracted from ^{1}H, ^{183}W HMBC NMR spectra acquired with different offsets (vertical bars) and two different sweep widths of complex **16a**, 400 MHz, $C_6D_5CD_3$, 25ºC.

Comparison of 1D ^{183}W NMR Spectrum with 2D $^{1}H,^{183}W$ HMBC Projection

a) 1D INEPT ^{183}W NMR spectrum of **4b**

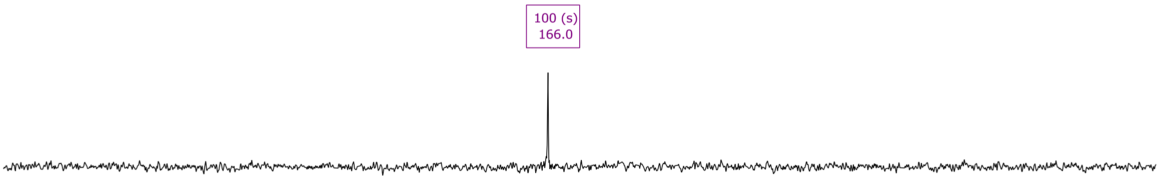

b) Projection of 2D ^{1}H, ^{183}W HMBC NMR spectrum of **4b**

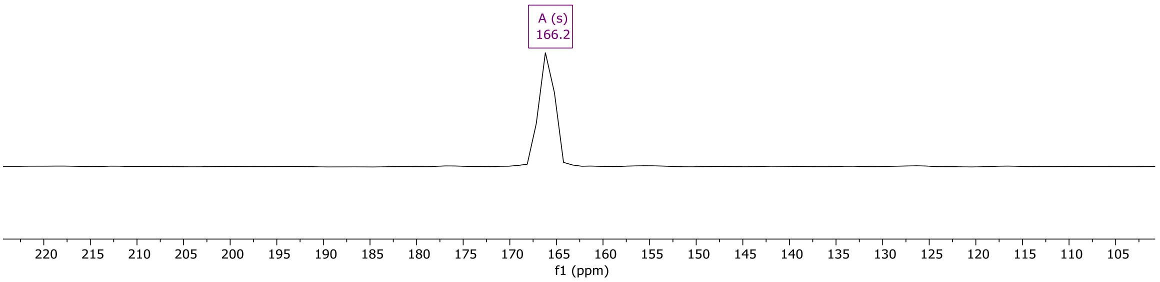

Figure S-13. Comparison of 1D INEPT ^{183}W NMR spectrum with 2D ^{1}H, ^{183}W HMBC projection: the shifts are identical within ±0.2 ppm.

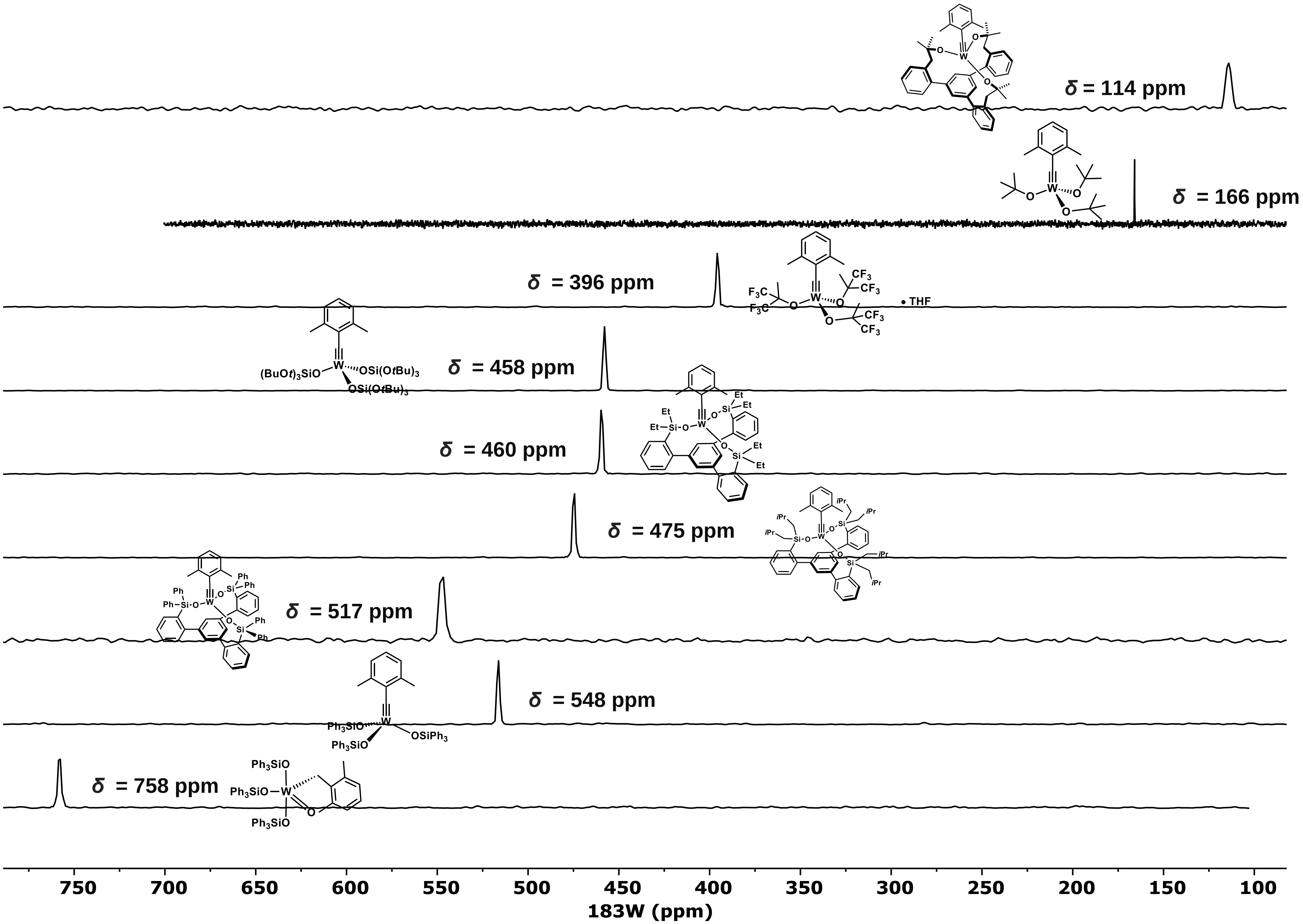

^{183}W NMR shifts of different tungsten complexes, as determined by ^{1}H,^{183}W HMBC experiments.

NMR Shift and Coupling Constants

Table S-2. ^{183}W-, ^{29}Si-, and ^{13}C-NMR shifts ([D_8]-toluene, 25°C) of the different complexes.

complex	δ (^{183}W) [ppm]	δ (^{29}Si) [ppm]	δ (^{13}C) [ppm]
**23**	114.2	–	264.0
**4b**	166	–	271.3
**5b·THF**	395.7	–	289.8
**10b**	458.1	–93.2	281.2
**16b**	460	12.9	281.5
**16d**	460	11.6	282.1
**16c**	475	9.9	281.9
**16a**	517	–8.5	287.4
**8b**	548	–8.0	283.1
**19**	758	–10.4	64.3

Table S-3. *J*-^{183}W-^{13}C-coupling constants ([D_8]-toluene, 25°C) of different complexes.

complex	^{1}J-^{183}W-^{13}C [Hz]	^{2}J-^{183}W-^{13}C [Hz]	δ (^{13}C) [ppm]
**23**	292.7	44.2	264.0
**4b**	297.3	44.8	271.3
**5b·THF**	283.9	40.6	289.8
**10b**	274.8	38.7	281.2
**16b**	272.5	38.8	281.5
**16d**	274.3	38.7	282.1
**16c**	272.8	38.6	281.9
**16a**	270.1	37.1	287.4
**8b**	273.0	38.1	283.1
**19**	101.9	not detected	64.3

Dilution Experiment and DOSY NMR:

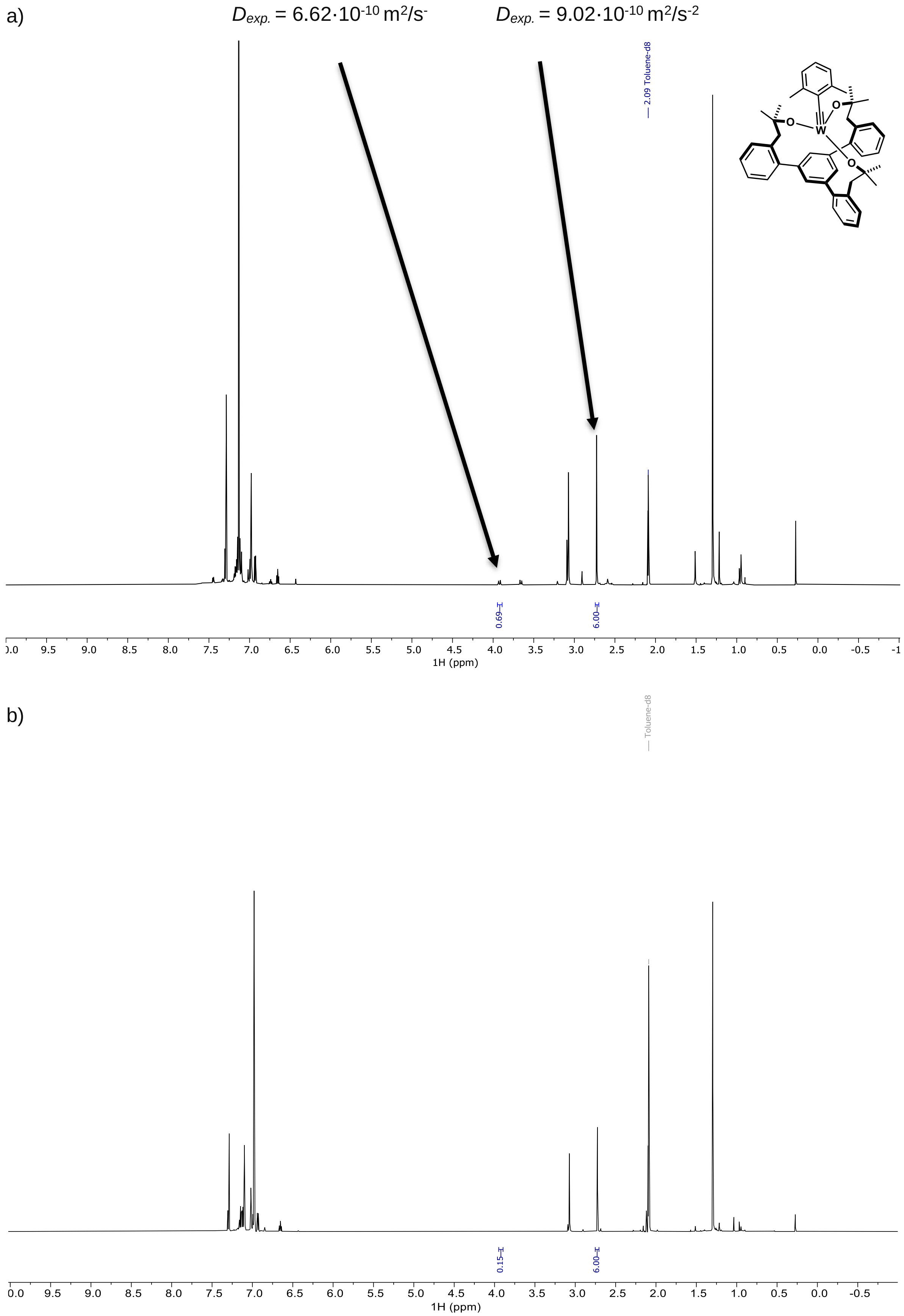

Figure S-14. ^{1}H-NMR spectra of tungsten alkylidyne complex **23** in [D$_8$]-toluene: a) at high concentration; b) at low concentration

Equilibration Kinetic Experiments

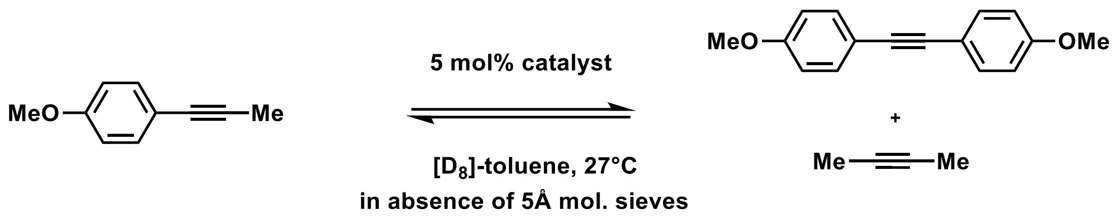

A flame dried *J. Young* NMR tube was filled with argon, charged with a stock solution of the complex (3.0 µmol, 0.5 mL, 5.0 µM in [D8]-toluene, 5 mol%) and a ^{1}H-NMR spectrum was recorded at 27°C. Then a solution of 1-methoxy-4-(prop-1yn-1-yl)benzene (100 µL, 60 µmol, 0.6 M in [D8]-toluene, 1.0 equiv.) was added and the conversion of this substrate to 1,3-bis(4-methoxyphenyl)-2-propa-1,2-diyne and 2-butyne was monitored by ^{1}H-NMR spectroscopy at 27°C. The data was corrected for relaxation effects as the relaxation time of 2-butyne is significantly longer due to its high symmetry and small size. Therefore two measurements were compared after the reaction: One with the standard data set and one with a longer delay (30s). Correction factor: 1-methoxy-4-(prop-1yn-1-yl)benzene = 1.023; 2-butyne = 1.127; 1,3-bis(4-methoxyphenyl)-2-propa-1,2-diyne = 1.028. Mass balance represents the sum of 2-butyne, 1-methoxy-4-(prop-1yn-1-yl)benzene and 1,3-bis(4-methoxyphenyl)-2-propa-1,2-diyne.

Benchmarking experiment in the molybdenum catalyst series:[1]

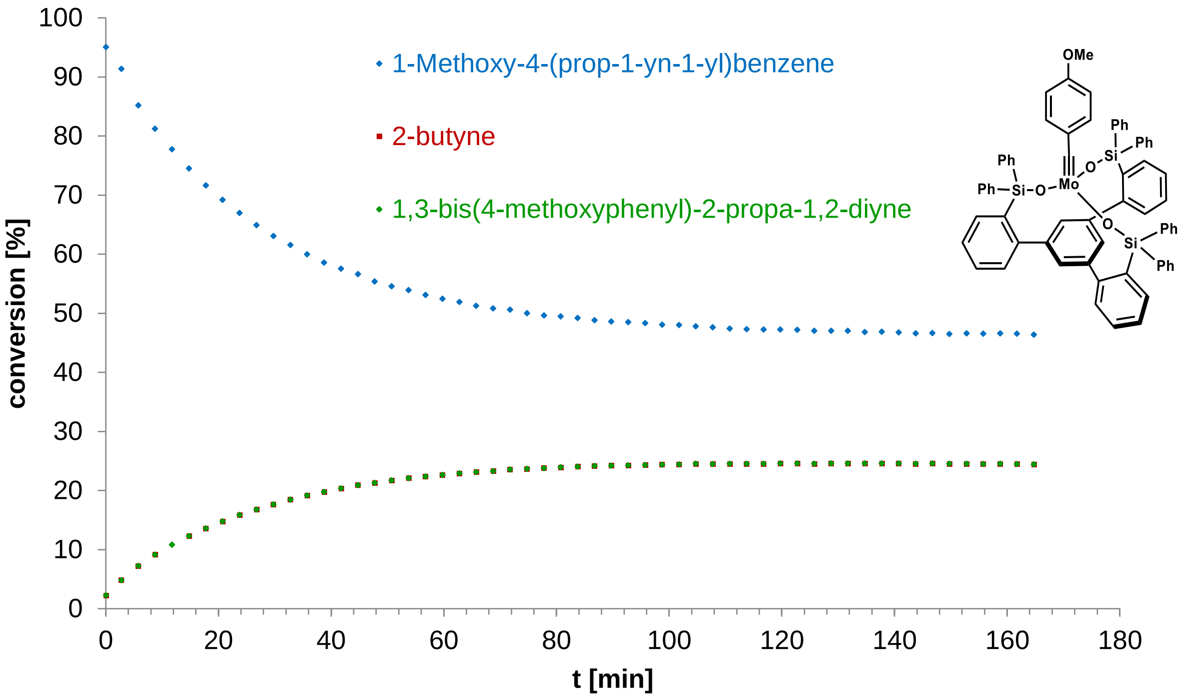

Figure S-15. Well-behaved consumption profile of 1-methoxy-4-(prop-1yn-1-yl)benzene and generation of 1,3-bis(4-methoxyphenyl)-2-propa-1,2-diyne and 2-butyne in [D8]-toluene, 27°C, 5 mol% molybdenum catalyst.[1]

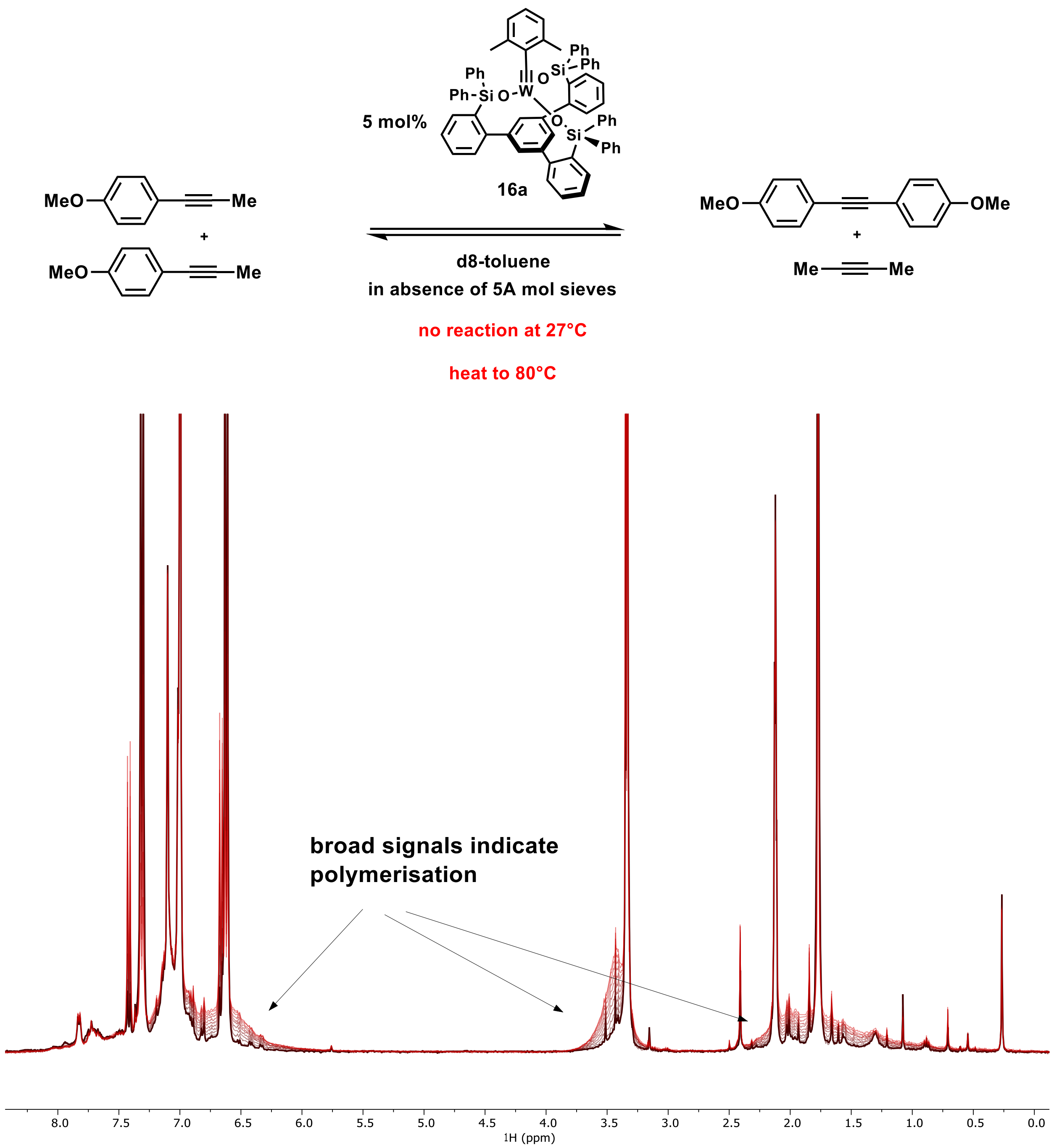

Figure S-16. Benchmarking of complex **16a** (5 mol%). Superimposed ^{1}H-NMR spectra: no consumption of 1-methoxy-4-(prop-1yn-1-yl)benzene and generation of 1,3-bis(4-methoxyphenyl)-2-propa-1,2-diyne and 2-butyne in [D_8]-toluene at 27°C; at 80°C significant polymerization was observed.

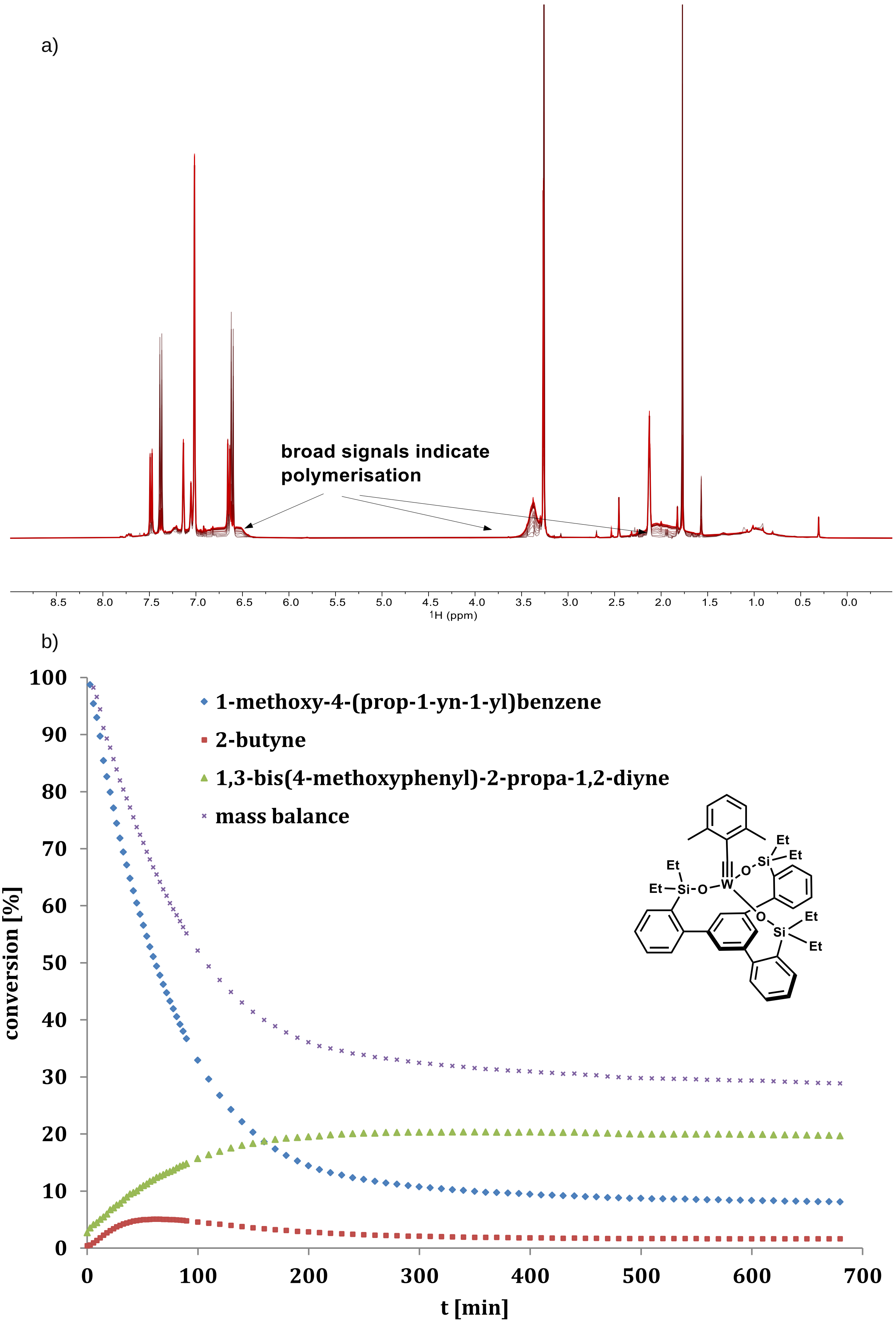

Figure S-17. Benchmarking of 5 mol% catalyst **16b**: a) Superimposed ^{1}H-NMR spectra b) Consumption of 1-methoxy-4-(prop-1yn-1-yl)benzene and generation of 1,3-bis(4-methoxyphenyl)-2-propa-1,2-diyne and 2-butyne in [D$_8$]-toluene, 27°C**.**

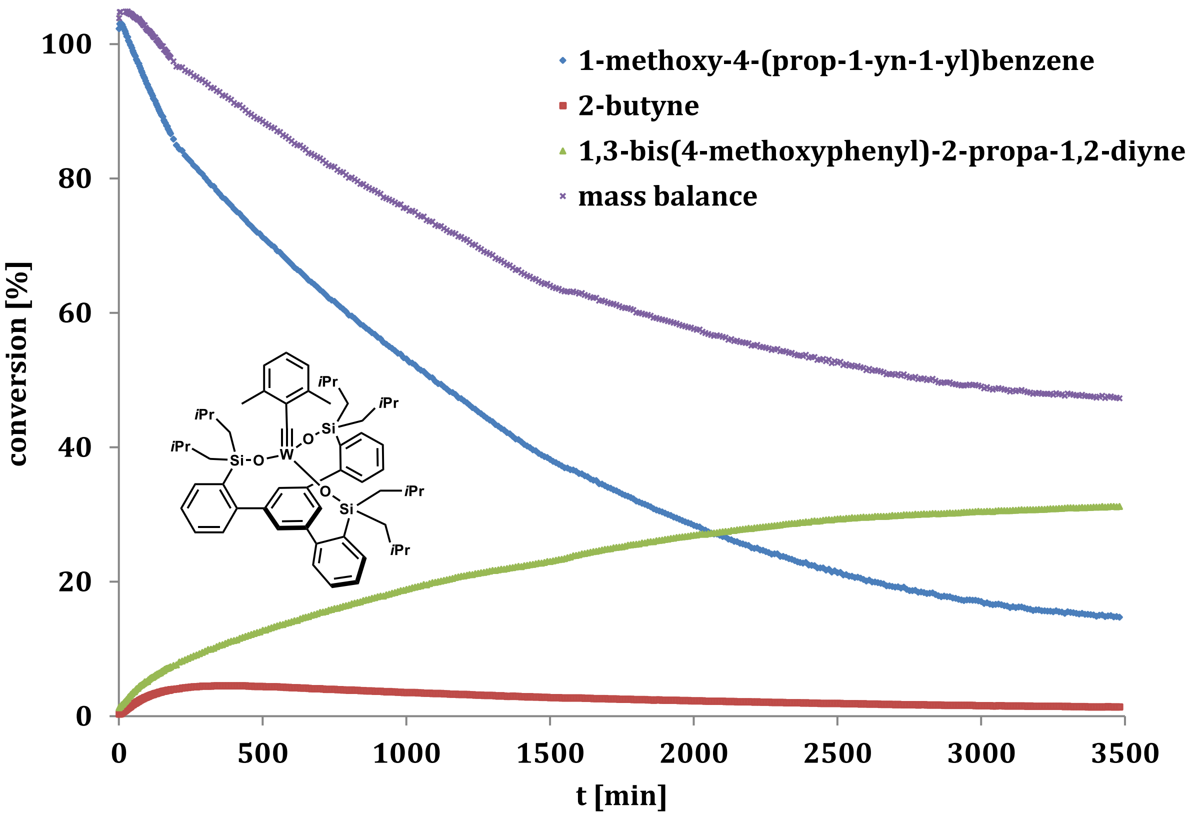

Figure S-18. Benchmarking of catalyst **16c** (5 mol%): Consumption of 1-methoxy-4-(prop-1yn-1-yl)benzene and generation of 1,3-bis(4-methoxyphenyl)-2-propa-1,2-diyne and 2-butyne in [D8]-toluene, 27°C,

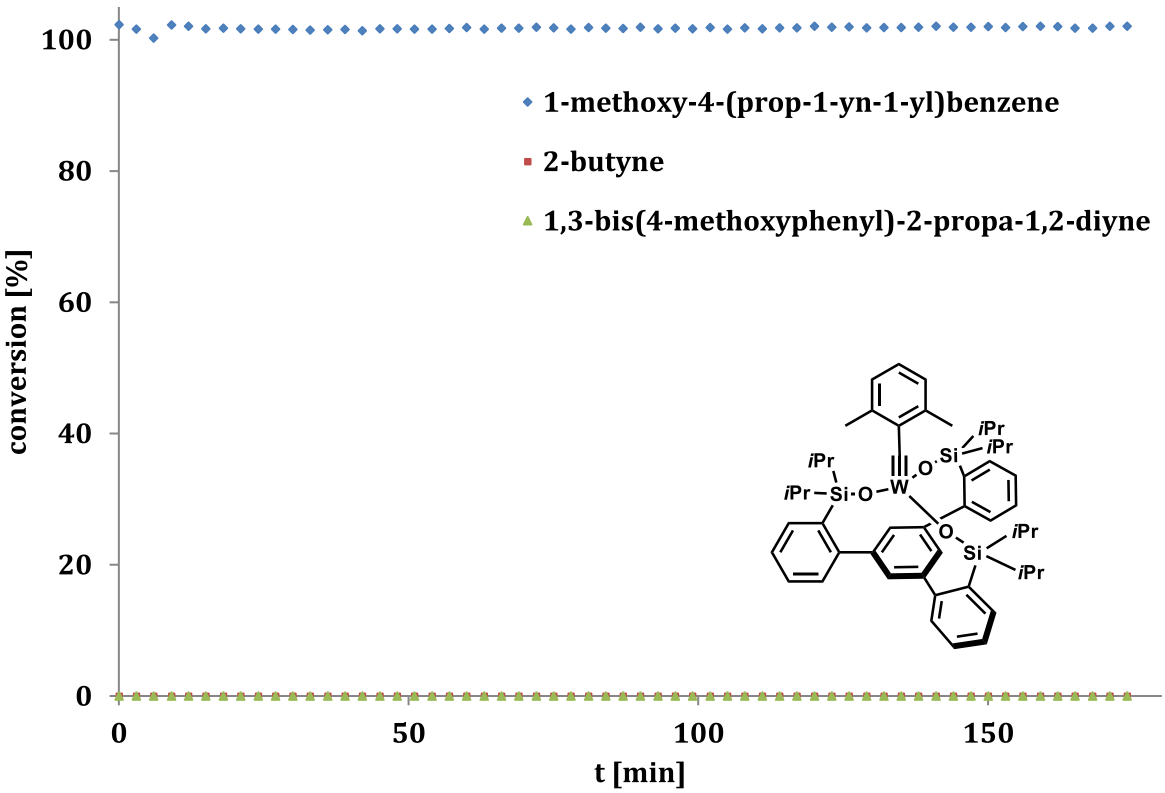

Figure S-19. Benchmarking of catalyst **16d** (5 mol%): Consumption of 1-methoxy-4-(prop-1yn-1-yl)benzene and generation of 1,3-bis(4-methoxyphenyl)-2-propa-1,2-diyne and 2-butyne in [D8]-toluene, 27°C

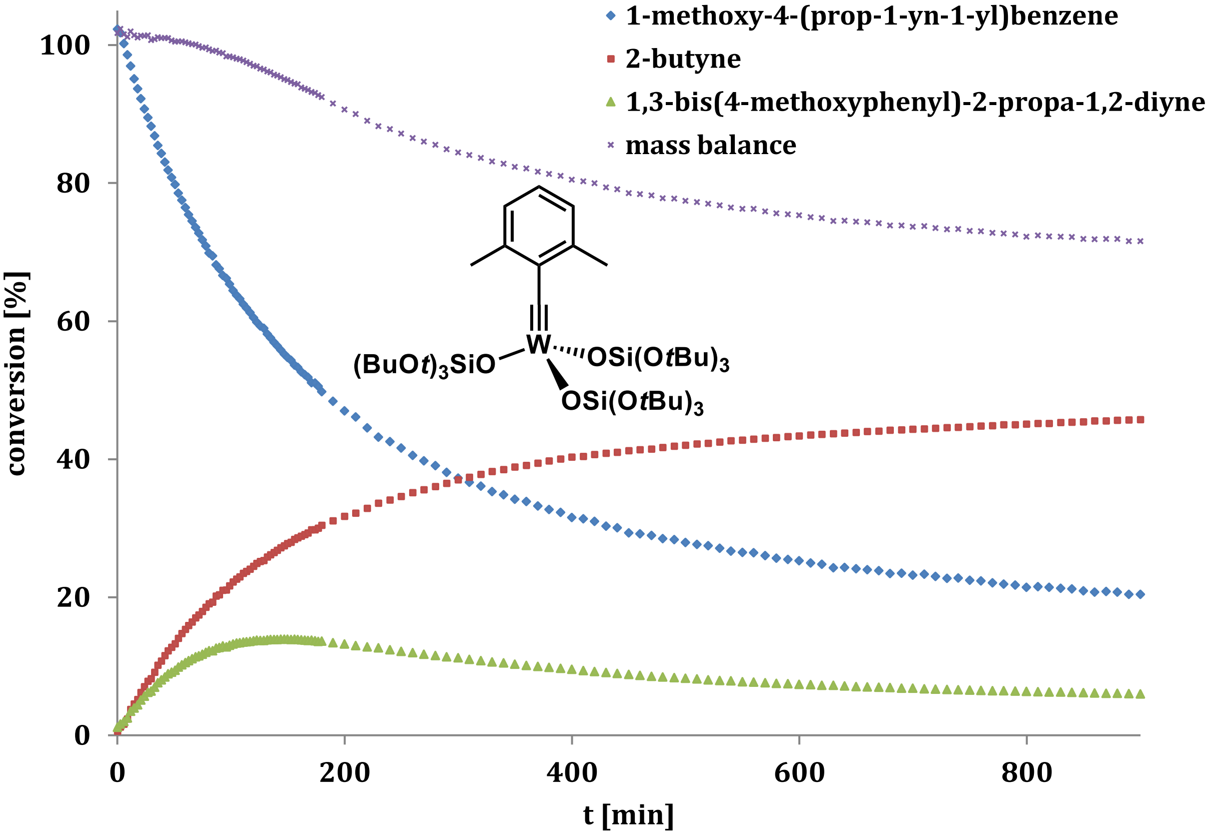

Figure S-20. Benchmarking of catalyst **10b** (5 mol%): Consumption of 1-methoxy-4-(prop-1yn-1-yl)benzene and generation of 1,3-bis(4-methoxyphenyl)-2-propa-1,2-diyne and 2-butyne in [D8]-toluene, 27°C

a)

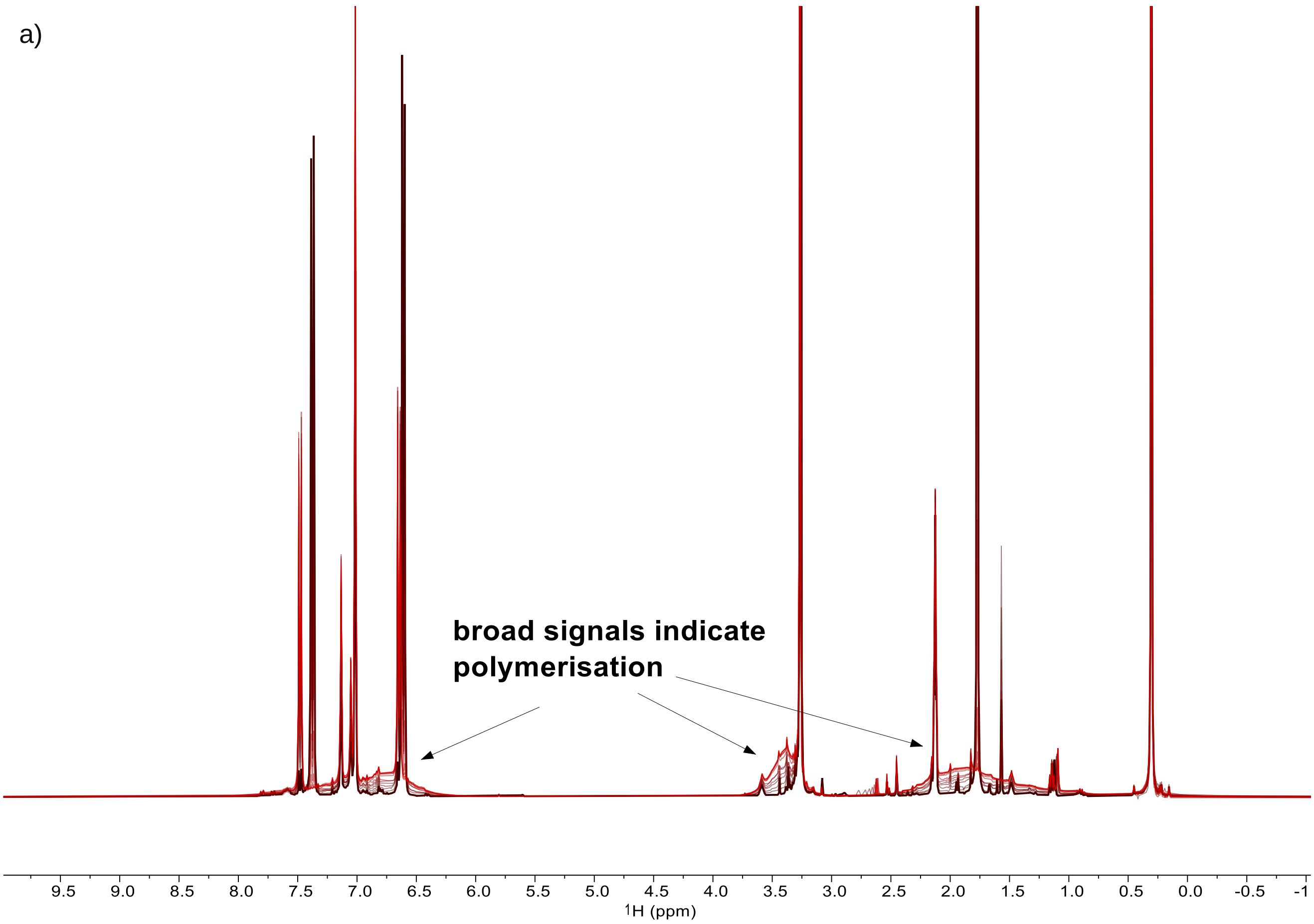

b)

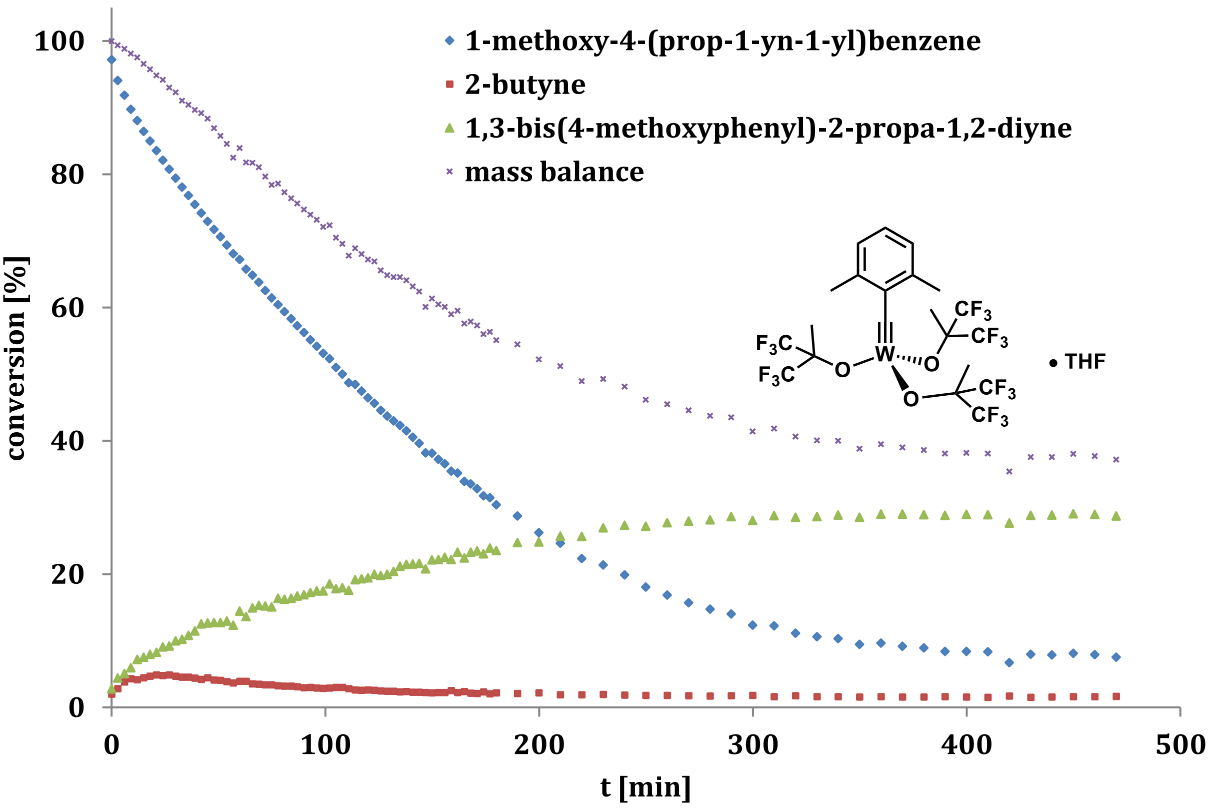

Figure S-21. Benchmarking of 5 mol% catalyst **5b**·THF: a) Superimposed ^{1}H-NMR spectra b) Consumption of 1-methoxy-4-(prop-1yn-1-yl)benzene and generation of 1,3-bis(4-methoxyphenyl)-2-propa-1,2-diyne and 2-butyne in [D$_8$]-toluene, 27°C**.**

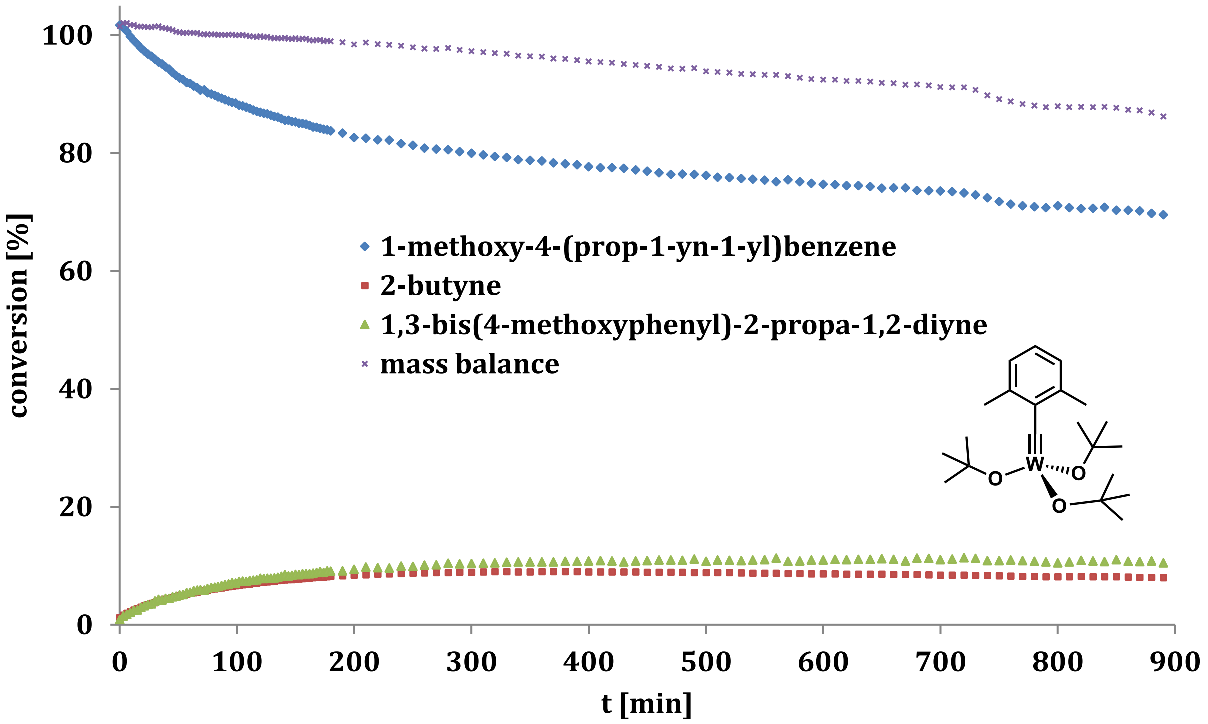

Figure S-22. Benchmarking of catalyst **4b** (5 mol%): Consumption of 1-methoxy-4-(prop-1yn-1-yl)benzene and generation of 1,3-bis(4-methoxyphenyl)-2-propa-1,2-diyne and 2-butyne in [D8]-toluene, 27°C

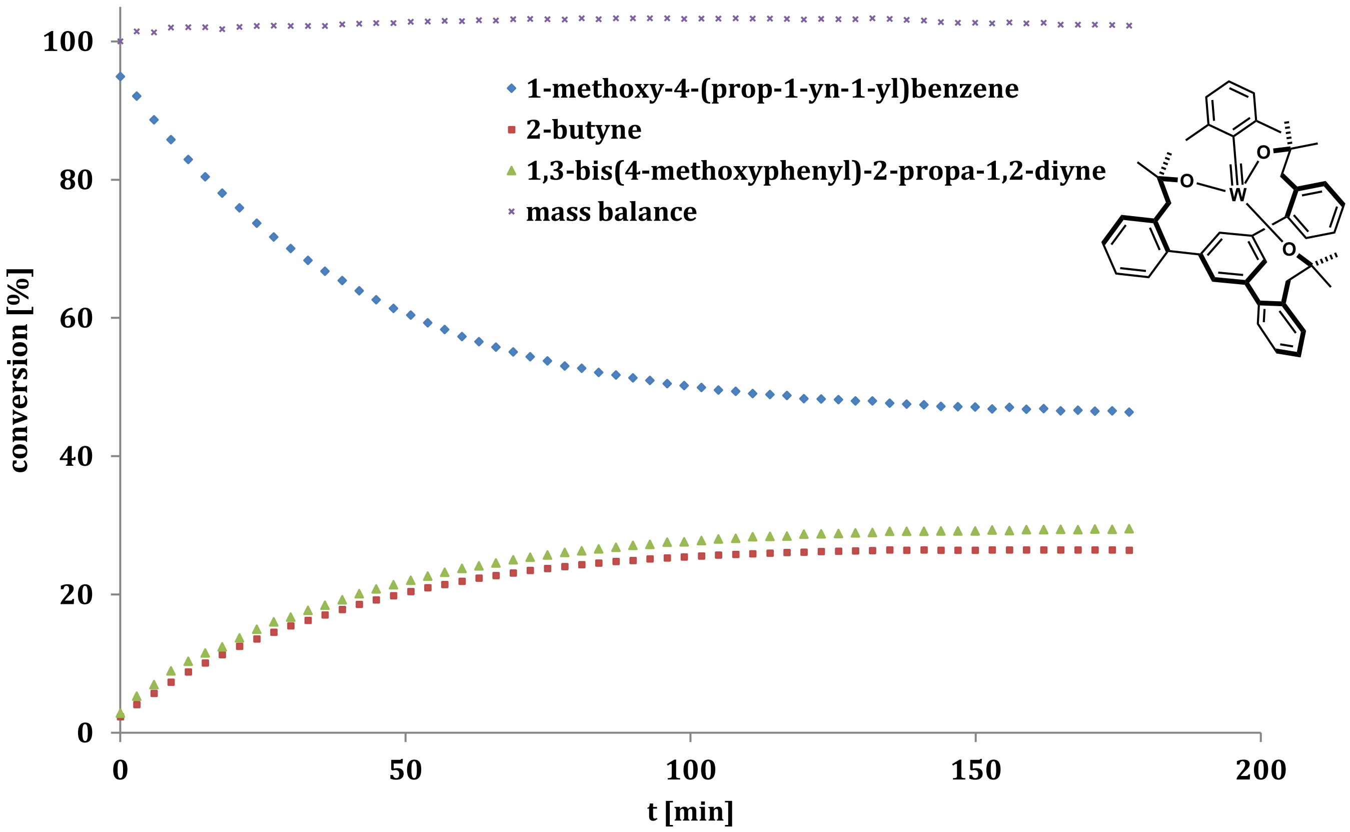

Figure S-23. Benchmarking of catalyst **23** (5 mol%): Consumption of 1-methoxy-4-(prop-1yn-1-yl)benzene and generation of 1,3-bis(4-methoxyphenyl)-2-propa-1,2-diyne and 2-butyne in [D8]-toluene, 27°C

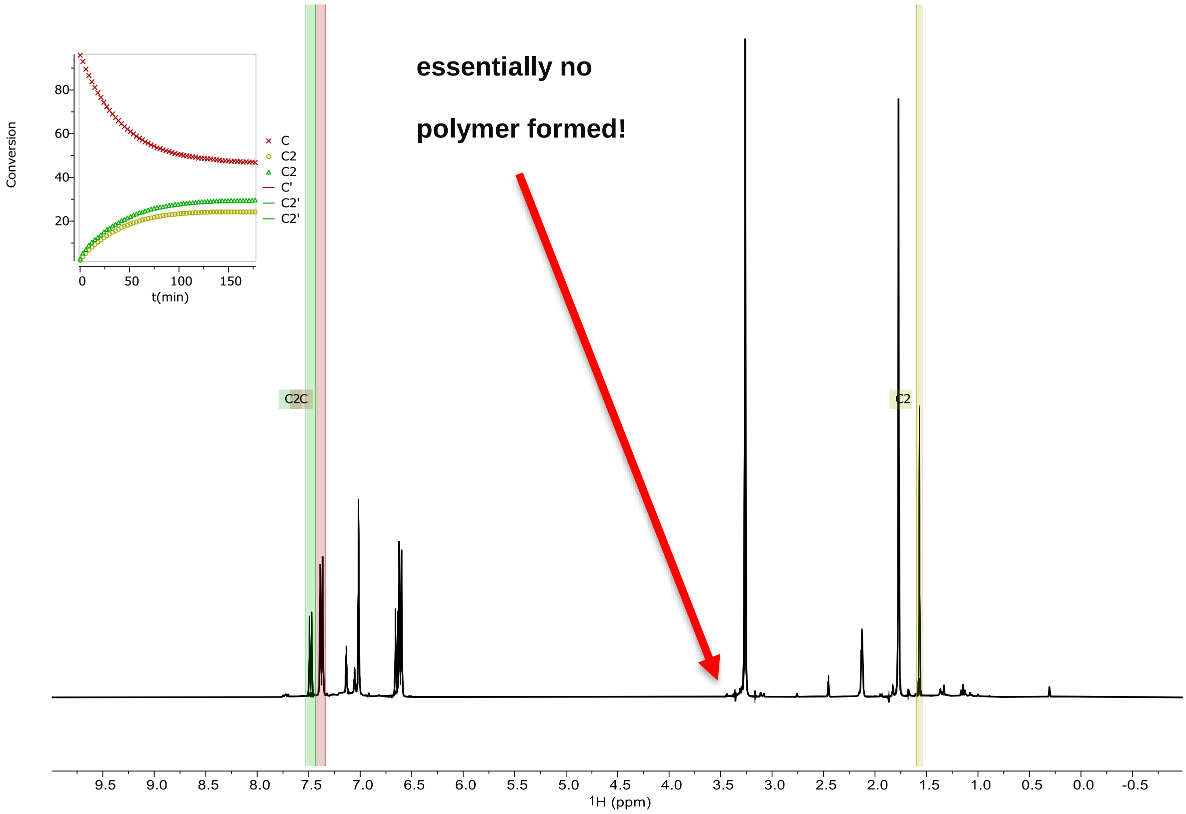

Figure S-24. Benchmarking of catalyst **23** (5 mol%): Superimposed ^{1}H-NMR spectra of the consumption profile in [D$_8$]-toluene, 27°C

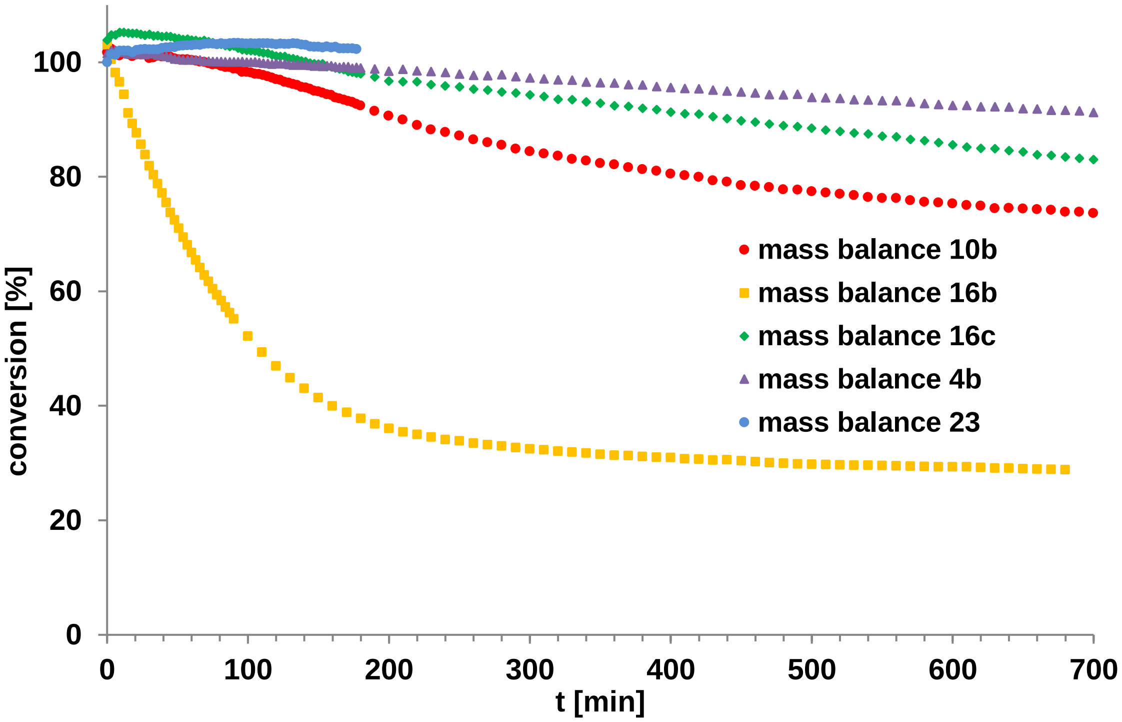

Figure S-25. Comparison of the mass balance during homo-metathesis of 1-methoxy-4-(prop-1yn-1-yl)benzene in [D$_8$]-toluene, 27°C (5 mol% of the respective tungsten catalyst).

Homo-Metathesis with Different Catalysts

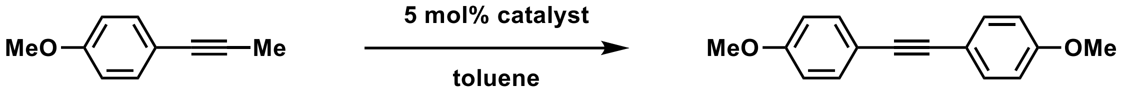

Table S-4. Homo-metathesis of 1-methoxy-4-(prop-1yn-1-yl)benzene in toluene (0.2 M), 5 mol% of different tungsten catalysts.

complex	additive	T (°C)	reaction time (h)	yield (% NMR)
**4b**	5Å mol. sieves	25	14	30
**8b**	5Å mol. sieves	25	14	no reaction
**16a**	5Å mol. sieves	25	14	no reaction
**16b**	5Å mol. sieves	25	14	56
**16c**	5Å mol. sieves	25	14	53
**16d**	5Å mol. sieves	25	14	no reaction
**10b**	5Å mol. sieves	25	14	53
**23**	5Å mol. sieves	25	14	69
**23**	–	25	4	76
**23**	silanized 5Å mol. sieves	25	2	85

Activation Parameters

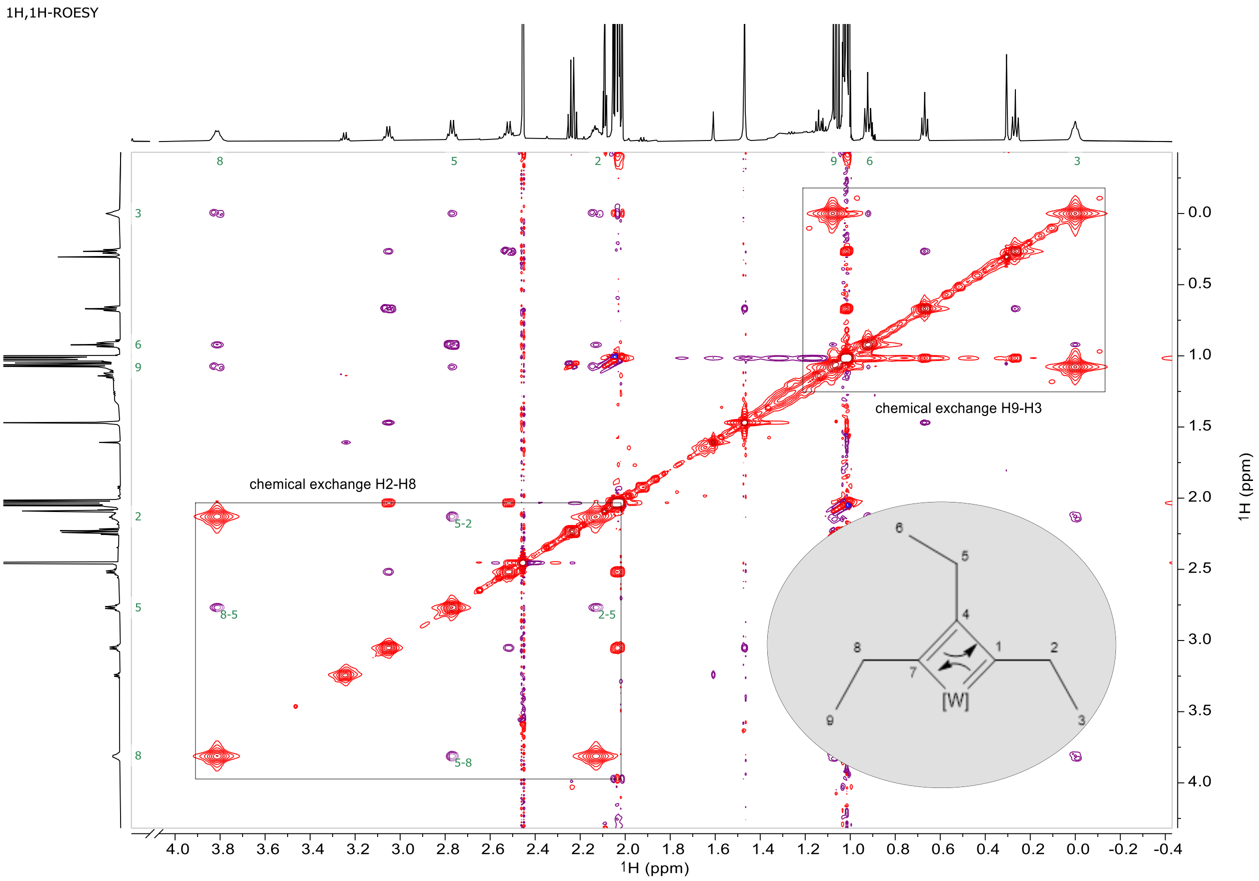

Figure S-26. Analysis of the 2D ROESY NMR experiment revealed a chemical exchange of two $C_\alpha/C_{\alpha'}$ ethyl groups in complex **18**.

^{1}H NMR signals of the four protons that are exchanging were simulated and fitted in the DNMR module of Bruker Topspin 4.09 at 5 different temperatures (243K, 253K, 263K, 273K, 283K) to obtain the exchange rate *k* between the groups at different temperatures. For the line-shape fitting a four site exchange model was used (H8->H2, H2->H8, H9->H3 and H3->H9). The results are shown in Table S-5.

Table S-5. Overview over all parameters used or obtained from lineshape fitting by DNMR. Values marked with an asterisk (*) were obtained from fitting. All other values were fixed.

T (K)	δ (ppm)				J_{H8H9} (Hz)	J_{H2H3} (Hz)	Intensity (a.u.)	lb (Hz)	*k** (hz)
	H8*	H2	H9	H3*					
243	3.8224	2.1031	1.1042	0.0099	7.342	7.501	2.04E+08	2.5	5.73634
253	3.8271	2.1432	1.0923	0.0139	7.342	7.501	6.37E+08	2.5	17.96200
263	3.8310	2.1876	1.0785	0.0189	7.342	7.501	4.22E+08	2.5	53.54910
273	3.8343	2.234	1.0485	0.0223	7.342	7.501	8.01E+08	2.5	150.36400
283	3.8293	2.3358	1.01	0.028	7.342	7.501	3.75E+07	2.5	370.35900

Line-Shape Analysis at 243K:

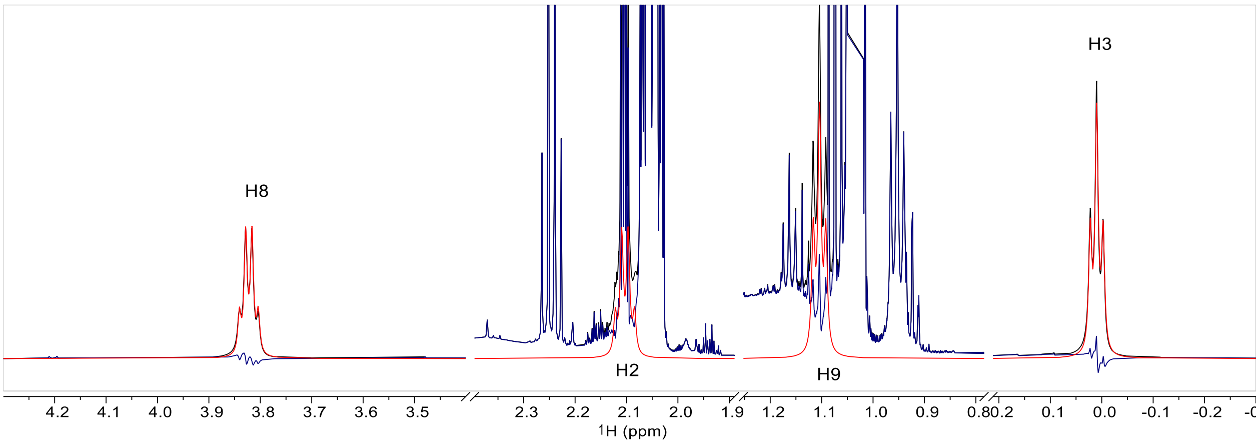

Figure S-27. Result from the line-shape simulation at 243K. Red: simulated spectrum obtained from DNMR after fitting; black: experimental spectrum; blue: difference between the experimental and the simulated spectrum.

Line-Shape Analysis at 253K:

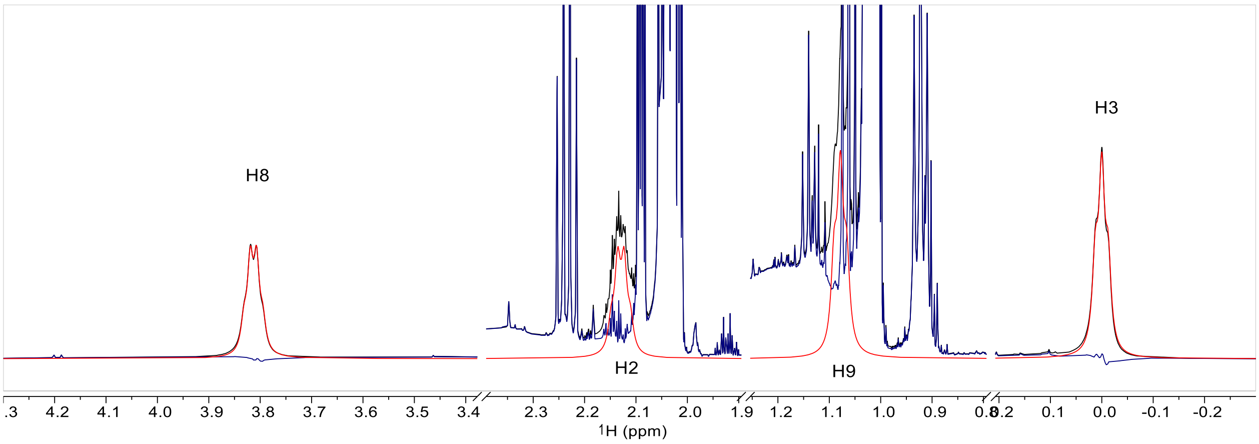

Figure S-28. Result from the line-shape simulation at 253K. Red: simulated spectrum obtained from DNMR after fitting; black: experimental spectrum; blue: difference between the experimental and the simulated spectrum.

Line-Shape Analysis at 263K:

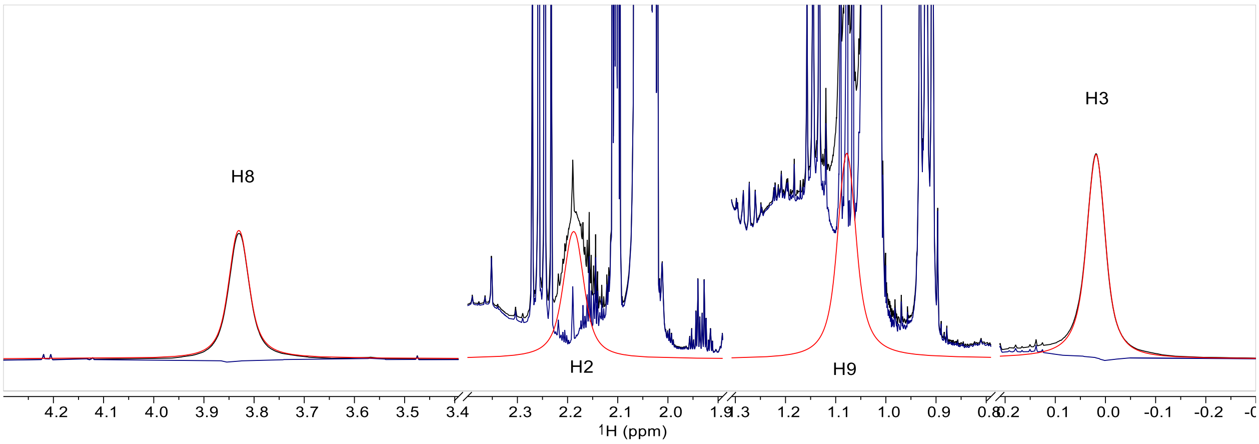

Figure S-29. Result from the line-shape simulation at 263K. Red: simulated spectrum obtained from DNMR after fitting; black: experimental spectrum; blue: difference between the experimental and the simulated spectrum.

Line-Shape analysis at 273K:

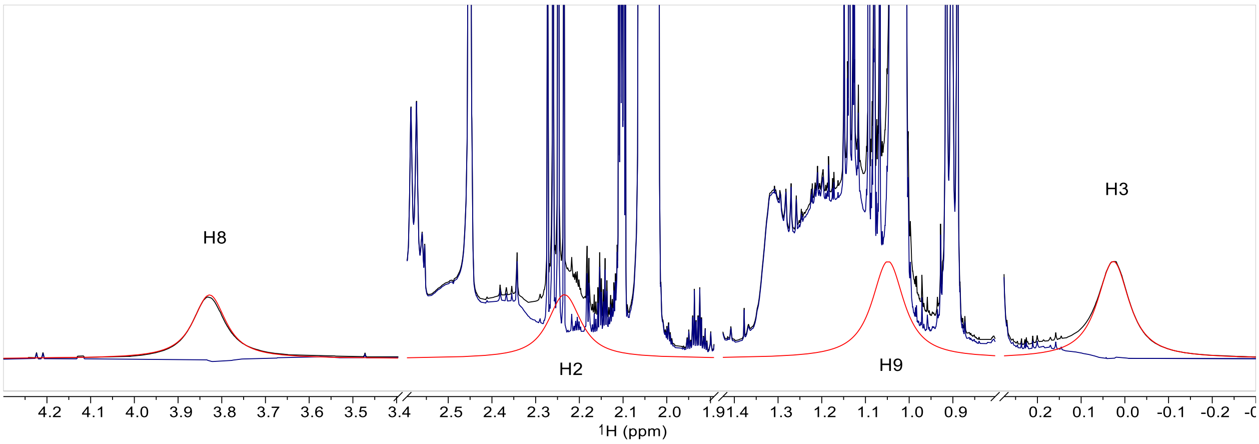

Figure S-30. Result from the line-shape simulation at 273K. Red: simulated spectrum obtained from DNMR after fitting; black: experimental spectrum; blue: difference between the experimental and the simulated spectrum.

Line-Shape Analysis at 283K:

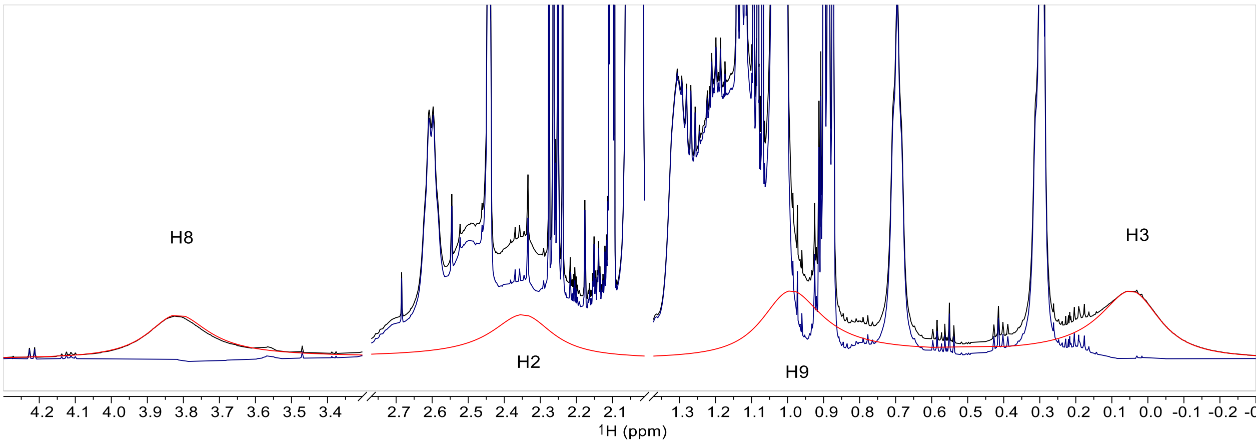

Figure S-31. Result from the line-shape simulation at 283K. Red: simulated spectrum obtained from DNMR after fitting; black: experimental spectrum; blue: difference between the experimental and the simulated spectrum.

Arrhenius Analysis

Table S-6. Physical constants used for Arrhenius and Eyring Analysis.

Name	value	unit
**R**	8.31446	J/(K*mol)
**h**	6.63E-34	J*s
**k_B**	1.38E-23	J/K

Table S-7. Parameters used for Arrhenius analysis.

T	k (hz)	1/(RT)	ln k
243	5.73634	0.000495	1.746821
253	17.962	0.000475	2.888258
263	53.5491	0.000457	3.980599
273	150.364	0.000441	5.013059
283	370.359	0.000425	5.914473

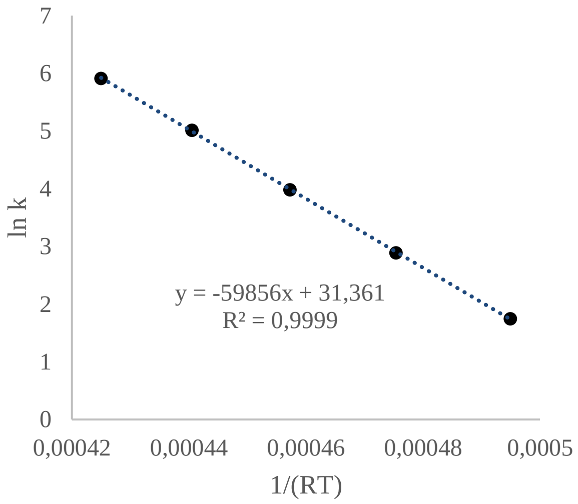

Figure S-32. Arrhenius plot obtained after ^{1}H lineshape analysis of the tautomerism observed in complex **18.**

Table S-8. Values from linear regression after plotting ln K against 1/RT.

	E_A	A
value	-59855.64	31.36073
std.dev	342.19696	0.157173
R^2	0.999902	0.018928
F	30595.517	3
ssreg	10.961848	0.001075

Table S-9. Activation energy obtained by Arrhenius analysis.

E_A	59.9	± 0.3 kJ/mol
	14.3	± 0.1 kcal/mol

Eyring Analysis

Table S-10. Parameters used for Eyring analysis.

**T(K)**	**1/*T***	**1/(RT)**	***k* (s-1)**	**(*k**h/(k_b*T))**	**ln ((*k**h/(k_b*T)))**
243.0	0.004115	4.949E-04	5.7363	1.13E-12	-27.5062
253.0	0.003953	4.754E-04	17.9620	3.41E-12	-26.4051
263.0	0.003802	4.573E-04	53.5491	9.77E-12	-25.3515
273.0	0.003663	4.406E-04	150.3640	2.64E-11	-24.3564
283.0	0.003534	4.250E-04	370.3590	6.28E-11	-23.491

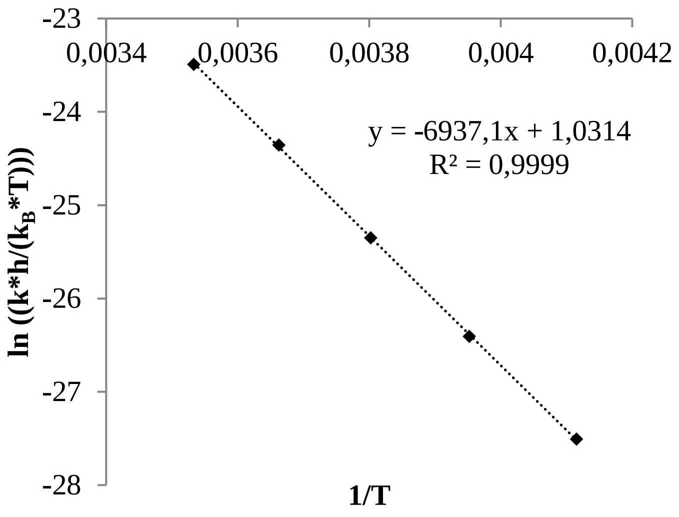

Figure S-33. Eyring plot obtained after ^{1}H lineshape analysis of the tautomerism observed in complex **18.**

Table S-11. Values obtained from linear regression after plotting ln ((*k**h/(k_b*T))) against 1/T

	-ΔH/R	ΔS/R
value	-6937.1100	1.0314
std.dev	40.6359	0.1552
R^2	0.9999	0.0187
F	29143.1447	3.0000
ssreg	10.1789	0.0010

Table S-12. Activation parameters obtained from the Eyring analysis:

ΔH	57.7	± 0.3 kJ/mol
**ΔH**	**13.8**	**± 0.1 kcal / mol**
ΔS	8.6	± 1.3 J/mol/K
**ΔS**	**2.05**	**± 0.31 cal / mol**
ΔG (25°C)	55.1	kJ/mol
**ΔG (25°C)**	**13.2**	**kcal / mol**

COMPUTATIONAL DETAILS

All geometry optimizations were performed with the Gaussian09 package[13] using the PBE0 functional.[14] W was represented by the quasi-relativistic effective core potential (RECP) from the Stuttgart group and the associated basis sets.[15-17] The remaining atoms (H, C, O, Si) were represented by a double-ζ Def2-SVP basis set.[18] Geometry optimizations were performed using the GD3 dispersion correction[19] and the SMD model[20] to account for the solvent (toluene)

NMR calculations were performed within the GIAO framework using ADF 2014[21] with the PBE0 functional and Slater-type basis sets of double-ζ quality (DZ). Relativistic effects were treated by the 2 component zeroth order regular approximation (ZORA).[22] Analysis of scalar-relativistic natural localized molecular orbitals were done with the NBO 6.0 program.[23] Calculated NMR shielding tensors were analyzed using these scalar-relativistic NLMO.[24,25] The 3D representation of the calculated shielding tensors were obtained as polar plots[26] of functions $\sum_{ij} r_i\, \sigma_{ij} r_j$.

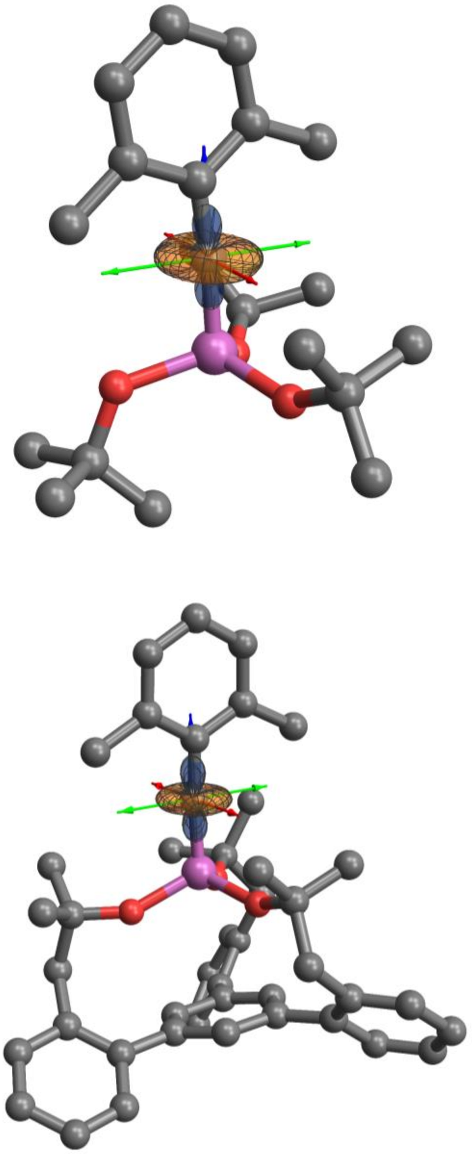

Figure S-34. Graphical representation of the shielding tensors of the Schrock complex **4b** (top) and the new complex **23** (bottom) with the tripodal ligand framework, which is more shielded in all three principle components.

Table S-13. Natural chemical shielding (NCS) analysis of alkylidyne carbon atom of complexes **16a**, **8b**, **10b**, **4b**, and **23**; all values are reported in ppm.

	σ_{xx}	σ_{dia}	σ_{para}	components of σ_{para}			
				σ(M-C)	σ(C-C')	π(M-C)	π(M-C')
**16a** (σ_{11})	-190	244	-434	-251	-144	-28	-15
**16a** (σ_{22})	-171	243	-414	-233	-119	-15	-33
**16a** (σ_{33})	144	305	-161	-6	-3	-45	-47
**8b** (σ_{11})	-186	251	-437	-252	-132	-35	-14
**8b** (σ_{22})	-169	247	-416	-231	-117	-15	-34
**8b** (σ_{33})	170	305	-135	-6	-3	-35	-38
**10b** (σ_{11})	-203	247	-450	-259	-133	-39	-14
**10b** (σ_{22})	-192	246	-438	-241	-126	-14	-38
**10b** (σ_{33})	184	306	-122	-5	-2	-35	-25
**4b** (σ_{11})	-220	248	-468	-257	-129	-57	-11
**4b** (σ_{22})	-183	248	-430	-234	-118	-22	-41
**4b** (σ_{33})	187	304	-117	-7	-2	-26	-25
**23** (σ_{11})	-191	247	-438	-254	-150	-27	-14
**23** (σ_{22})	-170	245	-415	-233	-120	-12	-36
**23** (σ_{33})	205	304	-100	-4	-1	-20	-24

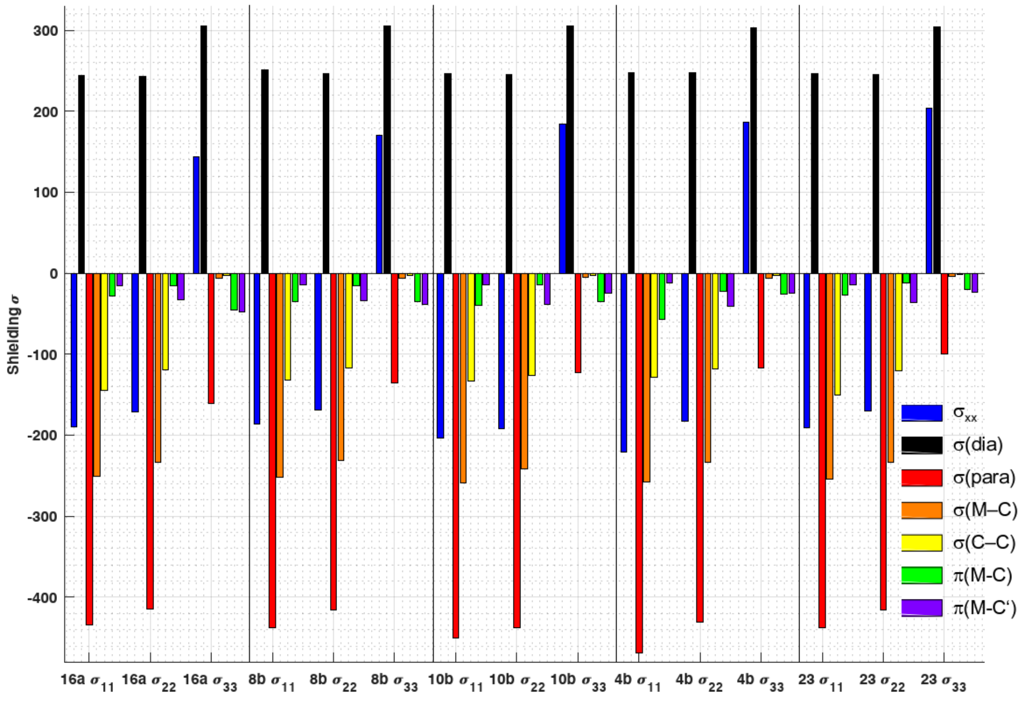

Figure S-35. Graphical representation of the data of the NCS analysis

Table S-14. Calculated energies (eigenvalues) of selected MOs

compound	MO	symmetry with respect to M–C axis	eigenvalue [Ha]
**4b**	LUMO+5	σ*(M–C)	0.828
**4b**	LUMO+1	π*(M–C)	-0.077
**4b**	LUMO	π*(M–C)	-0.643
**4b**	HOMO	π(M–C)	-5.466
**4b**	HOMO-1	π(M–C)	-5.930
**4b**	HOMO-6	σ(M–C)	-8.232
**23**	LUMO+13	σ*(M–C)	0.950
**23**	LUMO+7	π*(M–C)	0.051
**23**	LUMO+2	π*(M–C)	-0.514
**23**	HOMO	π(M–C)	-5.329
**23**	HOMO-1	π(M–C)	-5.886
**23**	HOMO-15	σ(M–C)	-8.342

REFERENCES

1. Hillenbrand, J.; Leutzsch, M.; Yiannakas, E.; Gordon, C. P.; Wille, C.; Nöthling, N.; Copéret, C.; Fürstner, A., *J. Am. Chem. Soc,* **2020,** *142*, 11279-11294.
2. Lackner, A. D.; Fürstner, A., *Angew. Chem*. **2015,** *127*, 13005-13009.
3. Jarupatrakorn, J.; Coles, M. P.; Tilley, T. D., *Chem. Mater.* **2005,** *17*, 1818-1828.
4. Harris, R. K.; Becker, E. D.; Cabral de Menezes, S. M.; Granger, P.; Hoffman, R. E.; Zilm, K. W., *Pure Appl. Chem.*, 2008; Vol. 80, p 59.
5. Evans, R.; Dal Poggetto, G.; Nilsson, M.; Morris, G. A., *Analyt.l Chem.* **2018,** *90*, 3987-3994.
6. Haberlag, B.; Freytag, M.; Daniliuc, C. G.; Jones, P. G.; Tamm, M., *Angew. Chem. Int. Ed*. **2012,** *51*, 13019-13022.
7. Han, X.; Wang, L.; Li, J.; Zhan, X.; Chen, J.; Yang, J., *Appl. Surf. Sci.* **2011,** *257*, 9525-9531.
8. Jin, G.; Zhang, X.; Cao, S., *Org. Lett.* **2013,** *15*, 3114-3117.
9. Heppekausen, J.; Stade, R.; Goddard, R.; Fürstner, A., *J. Am. Chem. Soc.* **2010,** *132*, 11045-11057.
10. Fürstner, A.; Guth, O.; Rumbo, A.; Seidel, G., *J. Am. Chem. Soc.* **1999,** *121*, 11108-11113.
11. Fürstner, A.; Flügge, S.; Larionov, O.; Takahashi, Y.; Kubota, T.; Kobayashi, J., *Chem. Eur. J.* **2009,** *15*, 4011-4029.
12. Fürstner, A.; Mathes, C.; Lehmann, C. W., *Chem. Eur. J.* **2001,** *7*, 5299-5317.
13. Frisch, M. J.; Trucks, G. W.; Schlegel, H. B.; Scuseria, G. E.; Robb, M. A.; Cheeseman, J. R.; Scalmani, G.; Barone, V.; Mennucci, B.; Petersson, G. A.; Nakatsuji, H.; Caricato, M.; Li, X.; Hratchian, H. P.; Izmaylov, A. F.; Bloino, J.; Zheng, G.; Sonnenberg, J. L.; Hada, M.; Ehara, M.; Toyota, K.; Fukuda, R.; Hasegawa, J.; Ishida, M.; Nakajima, T.; Honda, Y.; Kitao, O.; Nakai, H; Vreven, T.; Montgomery, J. A.; Peralta, Jr. J. E.; Ogliaro, F.; Bearpark, M.; Heyd, J. J.; Brothers, E.; Kudin, K. N.; Staroverov, V. N.; Kobayashi, R.; Normand, J.; Raghavachari, K.; Rendell, A.; Burant,

J. C.; Iyengar, S. S.; Tomasi, J.; Cossi, M.; Rega, N.; Millam, J. M.; Klene, M.; Knox, J. E.; Cross, J. B.; Bakken, V.; Adamo, C.; Jaramillo, J.; Gomperts, R.; Stratmann, R. E.; Yazyev, O.; Austin, A. J.; Cammi, R.; Pomelli, C.; Ochterski, J. W.; Martin, R. L.; Morokuma, K.; Zakrzewski, V. G.; Voth, G. A.; Salvador, P.; Dannenberg, J. J.; Dapprich, S.; Daniels, A. D.; Farkas, Ö.; Foresman, J. B.; Ortiz, J. V.; Cioslowski, J.; Fox, D. J. Gaussian 09 (Gaussian, Inc., Wallingford CT, 2009) Version D.01.

14. Adamo, C.; Barone, V. *J. Chem. Phys.* **1999**, *110*, 6158.
15. Dolg, M.; Wedig, U.; Stoll, H.; Preuss, H. *J. Chem. Phys.* **1987**, *86*, 866-872.
16. Andrae, D.; Haeussermann, U.; Dolg, M.; Stoll, H.; Preuss, H. *Theor. Chim. Acta* **1990**, *77*, 123-141.
17. Martin, J. M. L.; Sundermann, A. *J. Chem. Phys.* **2001**, *114*, 3408-3420.
18. Weigend, F.; Ahlrichs, R. *Phys. Chem. Chem. Phys.* **2005**, *7*, 3297-3305.
19. Grimme, S.; Antony, J.; Ehrlich, S.; Krieg, H. *J. Chem. Phys.* **2010**, *132*, 154104
20. Marenich, A. V.; Cramer, C. J.; Truhlar, D. G. *J. Phys. Chem. B* **2009**, *113*, 6378-6396.
21. te Velde, G.; Bickelhaupt, F. M.; Baerends, E. J.; Fonseca Guerra, C.; van Gisbergen, S. J. A.; Snijders, J. G.: Ziegler, T. *J. Comp. Chem.* **2001**, *22*, 931. Amsterdam Density Functional (ADF) Theoretical Chemistry Vrieje Universitet see http://www.scm.com (Version 2014).
22. van Lenthe, E.; van Leeuwen, R.; Baerends, E. J.; Snijders, J. G. *Int. J. Quant. Chem.* **1996**, *57*, 281-293.
23. Glendening, E. D.; Badenhoop, J. K.; Reed, A. E.; Carpenter, J. E.; Bohmann, J. A.; Morales, C. M.; Landis, C. R.; Weinhold, F. Theoretical Chemistry Institute, University of Wisconsin, Madison, WI, USA **2013**, http://nbo6.chem.wisc.edu/
24. Bohmann, J. A.; Weinhold, F.; Farrar, T. C. J. *J. Chem. Phys.* **1997**, *107*, 1173-1184.
25. Autschbach, J. *J. Chem. Phys.* **2008**, *128*, 164112.
26. Zurek, E.; Pickard, C. J.; Autschbach, J. *J. Phys. Chem. C.* **2008**, *112*, 11744-11750.
